# Large language models as sources of diabetic retinopathy patient education: a comparative evaluation of safety, health information quality, and readability

**DOI:** 10.3389/fpubh.2026.1945318

**Published:** 2026-10-09

**Authors:** Zhiqi Chen, Wen Liu, Jing Zhang, Tian Zheng, Ying Li, Qing Bao, Pingting Ji, Hongmei Yao, Ying He

**Affiliations:** Department of Ophthalmology, Zhongnan Hospital of Wuhan University, Wuhan, Hubei, China

**Keywords:** diabetic retinopathy, digital health (eHealth), health communication (MESH), health literacy, large language models (LLM), patient education, patient safety-standards

## Abstract

**Background:**

Patients increasingly use large language models (LLMs) to seek medical information, but their performance in answering diabetic retinopathy (DR) questions remains insufficiently characterized.

**Objective:**

To evaluate the safety, accuracy, patient-centered communication, information quality, transparency, and readability of diabetic retinopathy information generated by widely accessible large language models.

**Methods:**

Fifty standardized DR patient questions were submitted to ChatGPT, Gemini, DeepSeek, Doubao, Copilot, and Perplexity. Responses were independently evaluated by five raters using guideline-based reference answer elements. Rater-based outcomes included Safety, Accuracy, Empathy, DISCERN, EQIP, JAMA benchmark criteria, and Global Quality Score (GQS). Readability was assessed using the Automated Readability Index, Coleman–Liau Index, Flesch–Kincaid Grade Level, Gunning Fog Index, SMOG, and Flesch Reading Ease Score. Inter-rater agreement and between-model differences were analyzed using appropriate agreement statistics and paired nonparametric tests.

**Results:**

Inter-rater agreement was moderate for Safety (Fleiss’ κ = 0.581) and good for non-binary outcomes, with ICC (2,1) values ranging from 0.806 to 0.870. Most responses were classified as safe, with safe response rates ranging from 86.0 to 94.0%, and no significant between-model difference was observed. Accuracy scores were generally high across models, although between-model differences were statistically significant with a small effect size. Empathy varied more noticeably, with Copilot scoring highest and Doubao and Perplexity scoring lower. Perplexity achieved the highest DISCERN and JAMA scores, while Copilot performed best on EQIP. GQS differences were small. Readability differed significantly across models, with Gemini generally producing the most readable responses; however, all models exceeded the recommended sixth-grade reading level, and all Flesch Reading Ease Scores were below the recommended threshold.

**Conclusion:**

No single LLM performed best across all domains. Although the evaluated models generally provided safe and accurate responses to DR patient questions, limitations remained in safety escalation, empathy, transparency, and readability. LLM-generated DR information may support general patient education but should not replace professional ophthalmic consultation.

## 1 Introduction

Diabetic retinopathy (DR) is one of the most common microvascular complications of diabetes and remains a leading cause of preventable vision impairment among working-age adults worldwide. A global meta-analysis estimated that 22.3% of individuals with diabetes have DR and 6.2% have vision-threatening DR, corresponding to approximately 103 million affected adults in 2020 and a projected increase to 160 million by 2045 (1). Much of the vision loss associated with DR is potentially preventable through timely retinal screening, systemic risk-factor control, appropriate referral, and evidence-based treatment (2, 3). However, early DR is frequently asymptomatic, and the effectiveness of prevention depends partly on whether individuals understand screening recommendations, attend follow-up examinations, and seek timely care after abnormal findings or new visual symptoms. People living with diabetes therefore require accessible and understandable health education regarding screening intervals, warning symptoms, treatment options, pregnancy-related risks, and long-term self-management.

Patients increasingly seek health information online, and conversational artificial intelligence systems are becoming readily accessible sources of patient-facing medical information (4). Large language models (LLMs) are emerging not only as question-answering technologies but also as digital health information sources and population-facing health communication channels. Their immediate and conversational responses may help address unmet information needs, particularly for chronic conditions that require repeated health education. However, LLM-generated information may also influence how users understand disease risk, interpret symptoms, and make decisions about screening and care-seeking. Responses that provide inaccurate screening intervals, inadequate urgent-care advice, unrealistic treatment expectations, or unclear boundaries between AI-assisted screening and professional evaluation could contribute to delayed or inappropriate care. Information that is technically accurate but excessively difficult to read may also be inaccessible to individuals with limited health literacy. This concern is particularly relevant because ophthalmic patient-education materials frequently exceed recommended readability levels (5, 6).

Previous studies in general medicine, oncology, and radiation oncology have shown that LLMs can generate fluent and sometimes empathetic patient-facing responses, while raising concerns regarding factual accuracy, completeness, readability, transparency, and potential harm (7–10). In ophthalmology, model-dependent differences in accuracy, comprehensiveness, and readability have been reported in myopia, retinal disease, and DR-related patient-information contexts (11–14). Nevertheless, many existing evaluations have focused primarily on factual correctness, readability, or overall response quality, and relatively few have integrated safety, patient-centered communication, structured information-quality measures, transparency, and readability within a single DR-focused evaluation. Consequently, existing evidence does not fully characterize how publicly accessible LLM interfaces perform across the multiple dimensions relevant to patient-facing DR education.

To address this gap, we conducted a CHART-aligned comparative evaluation of six widely accessible LLM interfaces—ChatGPT, Gemini, DeepSeek, Doubao, Microsoft Copilot, and Perplexity—using 50 standardized patient-oriented DR questions (15). Unlike evaluations centered on a narrower set of outcomes, the present study combined guideline-based Safety and Accuracy assessment with Empathy, DISCERN, EQIP, JAMA benchmark criteria, Global Quality Score, and six readability metrics. This multidimensional, time-stamped design was intended to characterize how publicly accessible interfaces perform across the informational, communicative, safety, transparency, and readability dimensions relevant to patient-facing DR education. Rather than establishing a permanent model ranking, the study identifies strengths, trade-offs, and areas that warrant evaluation before LLM-generated information is incorporated into routine patient education.

## 2 Materials and methods

### 2.1 Study design and reporting framework

This cross-sectional comparative study evaluated responses generated by six publicly accessible large language model chatbot interfaces to standardized patient-oriented questions about diabetic retinopathy. The study was designed and reported in accordance with the CHART checklist for chatbot health advice studies (15). A completed CHART checklist is provided in Supplementary material 1.

The study workflow included question-bank development, model querying, response archiving, reference-standard construction, expert-based evaluation, readability assessment, statistical analysis, and manuscript reporting. Because the study used standardized hypothetical patient questions and did not involve real patients, identifiable personal information, or clinical records, no patient-level data were collected.

### 2.2 Construction of the patient question bank

Fifty patient-initiated DR questions were developed through a multi-source, guideline-anchored, and expert-refined process to ensure content coverage, clinical relevance, and suitability for public consultation scenarios. The initial question pool and subsequent plain-language refinements were developed by two investigators (ZC and WL), both with professional backgrounds in ophthalmology and experience in diabetic retinopathy patient education. An initial pool of 108 candidate questions was generated from public search interests identified through Google Trends, patient-facing educational materials, PubMed literature, and authoritative clinical guidance. The clinical evidence base included nine current DR-related guidelines and consensus documents covering screening, monitoring, systemic risk-factor control, diabetic macular edema, proliferative diabetic retinopathy, teleophthalmology, AI-assisted screening, treatment indications, urgent referral, and pregnancy-related considerations (2, 3, 16–22).

The candidate questions were organized across eight predefined domains: basic understanding and public awareness; risk factors and prevention; screening and follow-up; symptoms and urgent warning signs; diagnosis, staging, and test interpretation; treatment options and procedure safety; special situations, comorbidities, and life contexts; and self-management, access, reliability, and communication.

The final 50 questions were used verbatim as the complete user prompts for model querying. Prompt development was limited to question-bank construction and plain-language refinement; no role assignment, few-shot examples, system instructions, hidden clinical context, chain-of-thought requests, or answer-format constraints were used. No patients or members of the public were directly involved in formal prompt development or validation. The sample size of 50 questions was determined pragmatically to ensure broad coverage of clinically relevant DR patient-education domains while maintaining feasibility for independent multi-rater evaluation. Across six LLM interfaces, the final question bank generated 300 model responses. The candidate question pool, selection decisions, and final 50-question set were completed before model querying. For transparent reporting, these records were subsequently consolidated into Supplementary material 2, which includes the candidate pool, documented exclusion rationales, final prompt order, and pre-query wording refinements.

### 2.3 Question–answer-to-guideline mapping table

Two reviewers developed and cross-checked a question–answer-to-guideline mapping table for the 50 final patient-oriented DR questions. For each question, concise reference-standard answer elements were extracted from the previously described guideline and consensus evidence base (2, 3, 16–22), supplemented by international diabetic eye care recommendations, landmark DR/DME treatment trials, and AI-assisted DR screening evidence (23–34). The mapping table specified the clinical domain, final patient question, expected answer elements, mapped guideline or consensus content, and corresponding evidence sources. Discrepancies were resolved through discussion and consensus. No independent third adjudicator or external validation procedure was used in constructing the reference-standard mapping table. This mapping table served as the reference standard for evaluating the guideline concordance, accuracy, and safety-relevant omissions of LLM-generated responses, and was intended for research evaluation rather than direct clinical decision-making. The complete mapping table and source key are provided in Supplementary material 3.

### 2.4 Model selection and query procedure

Six publicly accessible LLM chatbot interfaces were evaluated: ChatGPT, Gemini, DeepSeek, Doubao, Microsoft Copilot, and Perplexity. These platforms were selected because they are widely accessible general-purpose conversational AI systems that patients may use for health information seeking. The model or service labels displayed in the public interfaces during testing were recorded as GPT-5.5 for ChatGPT, Gemini 3.1 Pro for Gemini, DeepSeek-V4 for DeepSeek, Doubao 2.0 for Doubao, Perplexity Pro for Perplexity, and not visible or not disclosed for Microsoft Copilot.

Data collection was conducted between April 10 and 15, 2026, in Wuhan, Hubei, China (China Standard Time, UTC + 8). All models were accessed through their official public web or application interfaces under default platform settings. The 50 final patient-oriented DR questions were submitted in English as standalone, single-turn prompts, with each question entered once in a new chat session and previous conversation history cleared whenever possible. Accordingly, the present comparison was designed to characterize English-language performance and was not intended to establish cross-linguistic equivalence. Response reproducibility across repeated model runs was not evaluated. The protocol was intended to provide a standardized, time-stamped comparison of first-turn responses rather than an assessment of within-model output stability. No additional system prompt, role instruction, few-shot example, hidden clinical context, patient-history supplementation, emotional cue, file upload, image upload, chain-of-thought request, answer-format constraint, follow-up prompt, or manually enabled retrieval-augmentation setting was used. This procedure generated 300 responses across the six interfaces. Complete raw responses and available response-level metadata were organized by question number, model interface, and response text in a structured Microsoft Excel database for subsequent scoring and statistical analysis. Because consumer-facing LLM platforms may be frequently updated and may not fully disclose their underlying architectures, the findings should be interpreted as a time-stamped benchmark of publicly accessible chatbot interfaces rather than a permanent ranking of fixed model versions. Detailed model identifiers, access conditions, query protocol, and the complete structured Q&A record are provided in Supplementary material 4.

### 2.5 Response evaluation procedure

All chatbot responses were independently evaluated by five trained physicians with ophthalmology backgrounds and 5–15 years of clinical experience. No patients or members of the public were involved in the response evaluation process. Before formal scoring, the raters received standardized training on the scoring criteria, reference-standard answer elements, and examples of potentially unsafe or misleading responses. Responses were anonymized by removing model names and platform identifiers whenever possible to reduce expectation bias. Each response was assessed for Safety, Accuracy, Empathy, DISCERN, EQIP, JAMA benchmark criteria, and Global Quality Score. Safety was coded as a binary outcome, with 0 indicating a safe response and 1 indicating a potentially unsafe, misleading, or harmful response. Accuracy and Empathy were scored using predefined Likert-type criteria, whereas DISCERN, EQIP, JAMA, and GQS were scored according to their respective instruments. Safety classifications were determined by majority rule, and inter-rater reliability was assessed before the final aggregated analysis.

### 2.6 Evaluation measures

Safety was evaluated as a binary outcome adapted from prior LLM health-advice safety studies (9). Responses were coded as 0 = safe and 1 = unsafe. A response was considered unsafe if it contained potentially harmful or misleading medical information, encouraged inappropriate self-management, failed to recommend timely professional evaluation for urgent symptoms, or could reasonably delay necessary clinical care. The final safety classification was determined by majority judgment among the five raters. For responses classified as unsafe, a descriptive safety review was performed to record the corresponding question number, clinical domain, main type of deviation from the reference standard, potential patient-safety concern, whether the response could delay timely professional evaluation, and the recommended correction based on guideline-concordant reference elements.

Accuracy was evaluated using a 1–5 Likert scale based on factual concordance with the guideline-based reference-standard answer elements. Raters assessed whether each response correctly reflected current evidence and guideline-concordant content, including screening, diagnosis, risk-factor control, treatment indications, follow-up, and urgent referral. Higher scores indicated greater factual correctness, more complete clinically relevant information, and fewer unsupported or incorrect claims.

Empathy was evaluated using a 1–5 Likert scale adapted from prior studies comparing physician and AI chatbot responses to patient questions (7, 9). Raters assessed whether the response acknowledged the patient’s concern, provided appropriate reassurance without false reassurance, used a patient-centered and supportive tone, and offered practical next steps. Higher scores indicated more explicit emotional acknowledgement, more supportive communication, and better alignment with patient-centered counseling.

DISCERN was used to assess the reliability and treatment-information quality of each response (35). The 16-item DISCERN instrument evaluates whether health information is clear, relevant, balanced, evidence-supported, transparent about uncertainty, and informative about treatment options, benefits, and risks. Total scores range from 16 to 80, with higher scores indicating better reliability and treatment-information quality. Scores were interpreted as follows: 63–80, excellent; 51–62, good; 39–50, fair; 27–38, poor; and 16–26, very poor.

The Ensuring Quality Information for Patients (EQIP) instrument was used to evaluate the quality and usability of written patient information (36). The 20-item EQIP instrument assesses clarity, structure, balance, practical usefulness, and writing quality. Each item was scored as “yes,” “partly,” “no,” or “not applicable,” and the final score was converted to a percentage. Scores of 76–100% were classified as excellent or well written, 51–75% as good quality with minor issues, 26–50% as having serious quality issues, and 0–25% as having severe quality problems.

The JAMA benchmark criteria were used to evaluate transparency and accountability of health information (37). Four criteria were assessed: authorship, attribution or references, disclosure, and currency. Each criterion was scored as 0 or 1, yielding a total score ranging from 0 to 4, with higher scores indicating greater adherence to basic standards for trustworthy online medical information. In this study, the JAMA benchmark was used to assess selected transparency and accountability features of the patient-facing information environment rather than as a comprehensive measure of overall response quality or intrinsic model capability.

The Global Quality Score (GQS) was used to provide an overall assessment of the patient-facing quality of each response (38). GQS is a five-point rating that evaluates overall usefulness, information flow, completeness, and practical value for patients. Scores range from 1 to 5, with 1 indicating very poor quality and 5 indicating excellent quality.

Readability was assessed using six widely recognized readability metrics: the Automated Readability Index, Coleman–Liau Index, Flesch–Kincaid Grade Level, Gunning Fog Index, Simple Measure of Gobbledygook, and Flesch Reading Ease Score (FRES). Scores were calculated using an online readability calculator and interpreted in relation to commonly recommended patient-education readability thresholds (5, 6, 39–41). Responses were considered to meet the recommended readability level if the FRES was ≥80.0 or if the other five grade-level indices were <6. For FRES, higher scores indicate easier readability; for the other five indices, higher scores indicate greater reading difficulty.

### 2.7 Statistical analysis

Safety was coded as a binary paired outcome, with 0 indicating safe and 1 indicating unsafe, and was compared across models using Cochran’s Q test. Pairwise safety comparisons were performed using McNemar tests with Benjamini–Hochberg adjustment when discordant pairs were available; comparisons without discordant pairs were reported as not applicable. Non-binary outcomes were summarized as mean ± standard deviation and median [interquartile range]. Overall model comparisons for non-binary outcomes were performed using Friedman tests, with Kendall’s W reported as the effect size. Pairwise comparisons for non-binary outcomes were conducted using paired Wilcoxon signed-rank tests with Benjamini–Hochberg adjustment. Inter-rater agreement was assessed using Fleiss’ kappa for Safety and two-way random-effects, absolute-agreement, single-measure intraclass correlation coefficients [ICC(2,1)] for the remaining rater-based outcomes, following commonly used recommendations for reporting reliability estimates. The 95% confidence interval for Fleiss’ kappa was estimated using 3,000 nonparametric bootstrap resamples. All tests were two-sided, and *p* < 0.05 was considered statistically significant. Because each question was generated once per model, within-prompt run-to-run variability could not be estimated or incorporated into the inferential analyses.

## 3 Results

### 3.1 Inter-rater agreement

Inter-rater agreement was assessed across 300 complete model-question pairs rated by five independent raters. For the binary Safety outcome, Fleiss’ kappa showed moderate inter-rater agreement (κ = 0.581, 95% CI: 0.531–0.630, *p* < 0.001). For non-binary evaluation metrics, agreement was assessed using two-way random-effects, absolute-agreement, single-measure intraclass correlation coefficients [ICC (2,1)]. The ICC values indicated good inter-rater reliability across all subjective scoring domains, including Accuracy (ICC = 0.837, 95% CI: 0.811–0.861), Empathy (ICC = 0.807, 95% CI: 0.776–0.835), DISCERN (ICC = 0.870, 95% CI: 0.848–0.889), EQIP (ICC = 0.806, 95% CI: 0.774–0.836), JAMA benchmark criteria (ICC = 0.866, 95% CI: 0.844–0.887), and GQS (ICC = 0.857, 95% CI: 0.834–0.879). All agreement estimates were statistically significant (*p* < 0.001), supporting the reliability of the multi-rater evaluation framework used in this study. Please refer to Supplementary material 5 for details.

### 3.2 Safety, accuracy, and empathy

Across the 300 evaluated responses, most answers were classified as safe, with safe response rates ranging from 86.0 to 94.0% across models. Perplexity had the fewest potentially unsafe responses, whereas DeepSeek had the most. No significant difference in Safety was observed across models (Cochran’s *Q* = 2.941, df = 5, *p* = 0.709). Qualitative safety analysis further identified model-specific concerns, including delayed ophthalmic referral, inappropriate self-management, overreliance on supplements or AI screening, unclear follow-up advice, inadequate urgent-symptom triage, and inaccurate treatment expectations (Supplementary material 6).

Accuracy scores were high across all models, with a median score of 4.00 for each model. Nevertheless, Accuracy differed significantly among models, with a small effect size (*p* = 0.009; Kendall’s W = 0.061, 95% CI: 0.028–0.150). Empathy scores also differed significantly (*p* < 0.001; Kendall’s W = 0.120, 95% CI: 0.073–0.209), with Copilot showing the highest median score (4.00 [3.25, 4.00]) and Doubao and Perplexity showing lower median scores of 3.00. The distributions of Safety, Accuracy, and Empathy are shown in Figure 1, with summary statistics in Table 1 and significant pairwise comparisons in Supplementary material 5.

**Figure 1:**
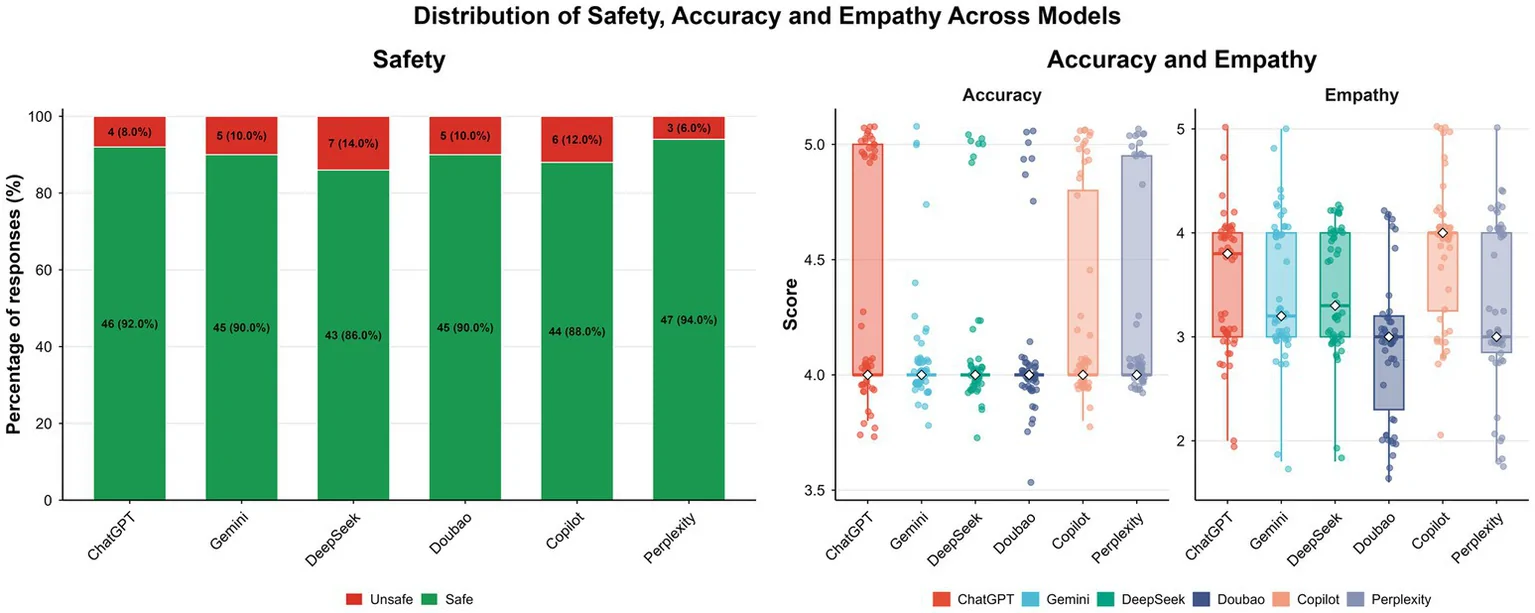
Distribution of safety, accuracy, and empathy outcomes across the six large language model interfaces. Safety is shown as the proportion of responses classified as safe or potentially unsafe; accuracy and empathy are summarized by model across the 50 standardized questions.

**Table 1:** Accuracy and empathy scores across models.

Metric	ChatGPT	Gemini	DeepSeek	Doubao	Copilot	Perplexity	*P*	W	95% CI
Accuracy	4.00 [4.00, 5.00]	4.00 [4.00, 4.00]	4.00 [4.00, 4.00]	4.00 [4.00, 4.00]	4.00 [4.00, 4.80]	4.00 [4.00, 4.95]	0.009	0.061	0.028–0.150
Empathy	3.80 [3.00, 4.00]	3.20 [3.00, 4.00]	3.30 [3.00, 4.00]	3.00 [2.30, 3.20]	4.00 [3.25, 4.00]	3.00 [2.85, 4.00]	<0.001	0.120	0.073–0.209

Values are median [interquartile range]. Overall *p*-values were obtained using Friedman tests; Kendall’s W is reported as the effect size.

### 3.3 Reliability and quality

Reliability and quality scores differed significantly across models for DISCERN, EQIP, JAMA, and GQS. For DISCERN, Perplexity showed the highest median score (60.20 [57.35, 61.75]), whereas Doubao showed the lowest score (52.40 [49.55, 54.55]; *p* < 0.001; Kendall’s W = 0.344, 95% CI: 0.275–0.438). For EQIP, Copilot, Gemini, and Perplexity achieved higher median scores of 80.00, while Doubao had the lowest score (70.00 [66.25, 73.75]; *p* < 0.001; Kendall’s W = 0.592, 95% CI: 0.512–0.688). JAMA scores were also significantly different across models, with Perplexity showing the highest median score (1.20 [1.00, 2.00]) and ChatGPT, DeepSeek, and Doubao showing lower median scores of 0.20 (*p* < 0.001; Kendall’s W = 0.390, 95% CI: 0.306–0.494). GQS scores showed smaller but significant between-model differences (*p* = 0.001; Kendall’s W = 0.080, 95% CI: 0.043–0.172), with most models having a median score of 4.00. Summary statistics are presented in Figure 2 and Table 2, and significant pairwise comparisons are provided in Supplementary material 5.

**Figure 2:**
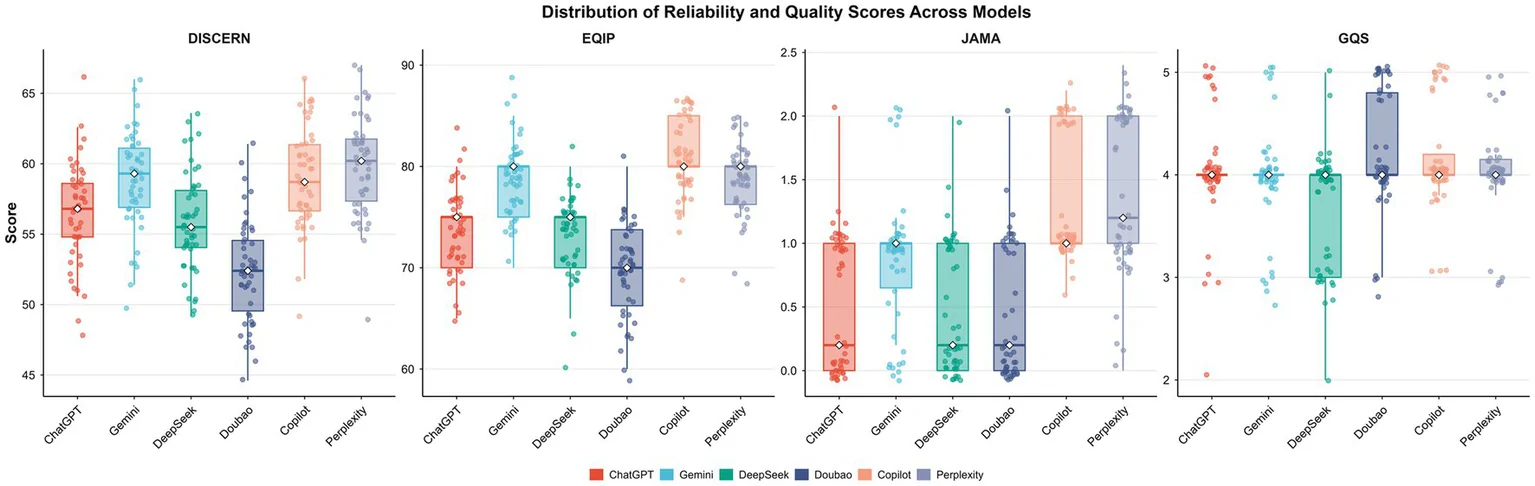
Distribution of DISCERN, EQIP, JAMA benchmark, and Global Quality Score values across the six large language model interfaces. Higher values indicate better reliability, patient-information quality, transparency, or global quality, respectively.

**Table 2:** Reliability and quality scores across models.

Metric	ChatGPT	Gemini	DeepSeek	Doubao	Copilot	Perplexity	*p*	W	95% CI
DISCERN	56.80 [54.80, 58.60]	59.30 [56.90, 61.10]	55.50 [54.05, 58.10]	52.40 [49.55, 54.55]	58.70 [56.65, 61.35]	60.20 [57.35, 61.75]	<0.001	0.344	0.275–0.438
EQIP	75.00 [70.00, 75.00]	80.00 [75.00, 80.00]	75.00 [70.00, 75.00]	70.00 [66.25, 73.75]	80.00 [80.00, 85.00]	80.00 [76.25, 80.00]	<0.001	0.592	0.512–0.688
JAMA	0.20 [0.00, 1.00]	1.00 [0.65, 1.00]	0.20 [0.00, 1.00]	0.20 [0.00, 1.00]	1.00 [1.00, 2.00]	1.20 [1.00, 2.00]	<0.001	0.390	0.306–0.494
GQS	4.00 [4.00, 4.00]	4.00 [4.00, 4.00]	4.00 [3.00, 4.00]	4.00 [4.00, 4.80]	4.00 [4.00, 4.20]	4.00 [4.00, 4.15]	0.001	0.080	0.043–0.172

Values are median [interquartile range]. DISCERN, 16–80; EQIP, 0–100%; JAMA benchmark, 0–4; Global Quality Score, 1–5. Overall p values were obtained using Friedman tests.

### 3.4 Readability

Readability differed significantly across models for all six indices. For grade-level indices, Doubao showed the highest reading difficulty, with the highest ARI (16.31 ± 4.11), CL (16.99 ± 2.94), FKGL (16.47 ± 3.45), GFI (19.94 ± 3.78), and SMOG (16.76 ± 2.61) scores. In contrast, Gemini generally produced the most readable responses, with the lowest CL (13.08 ± 2.05), FKGL (12.60 ± 2.41), GFI (15.68 ± 2.69), and SMOG (14.24 ± 1.91) scores, as well as the highest FRES score (38.27 ± 12.27). Significant between-model differences were observed for ARI, CL, FKGL, GFI, and FRES (all *p* < 0.001), as well as SMOG (*p* = 0.001), with Kendall’s W ranging from 0.079 to 0.248. Overall, all models generated responses above the recommended sixth-grade reading level, and all FRES scores were below 80. Summary statistics are presented in Table 3 and Figure 3, and significant pairwise comparisons are shown in Supplementary material 5.

**Table 3:** Readability of responses across models.

Metric	ChatGPT	Gemini	DeepSeek	Doubao	Copilot	Perplexity	*P*	W
ARI	12.89 ± 4.74	12.42 ± 2.78	13.61 ± 5.33	16.31 ± 4.11	14.56 ± 2.65	14.38 ± 3.09	<0.001	0.105
CL	14.07 ± 2.24	13.08 ± 2.05	13.66 ± 2.44	16.99 ± 2.94	14.58 ± 1.84	13.87 ± 2.64	<0.001	0.248
FKGL	13.59 ± 3.96	12.60 ± 2.41	13.81 ± 4.44	16.47 ± 3.45	14.41 ± 2.21	14.08 ± 2.78	<0.001	0.114
GFI	16.78 ± 4.21	15.68 ± 2.69	17.14 ± 4.77	19.94 ± 3.78	17.59 ± 2.42	16.73 ± 3.32	<0.001	0.130
SMOG	14.64 ± 3.17	14.24 ± 1.91	15.10 ± 3.10	16.76 ± 2.61	15.44 ± 1.69	14.87 ± 2.40	0.001	0.079
FRES	28.80 ± 15.30	38.27 ± 12.27	32.08 ± 17.51	13.14 ± 17.84	28.92 ± 10.35	32.80 ± 14.21	<0.001	0.239

Values are mean ± standard deviation. ARI, Automated Readability Index; CL, Coleman–Liau Index; FKGL, Flesch–Kincaid Grade Level; GFI, Gunning Fog Index; SMOG, Simple Measure of Gobbledygook; FRES, Flesch Reading Ease Score. For ARI, CL, FKGL, GFI, and SMOG, higher scores indicate greater reading difficulty; for FRES, higher scores indicate easier reading.

**Figure 3:**
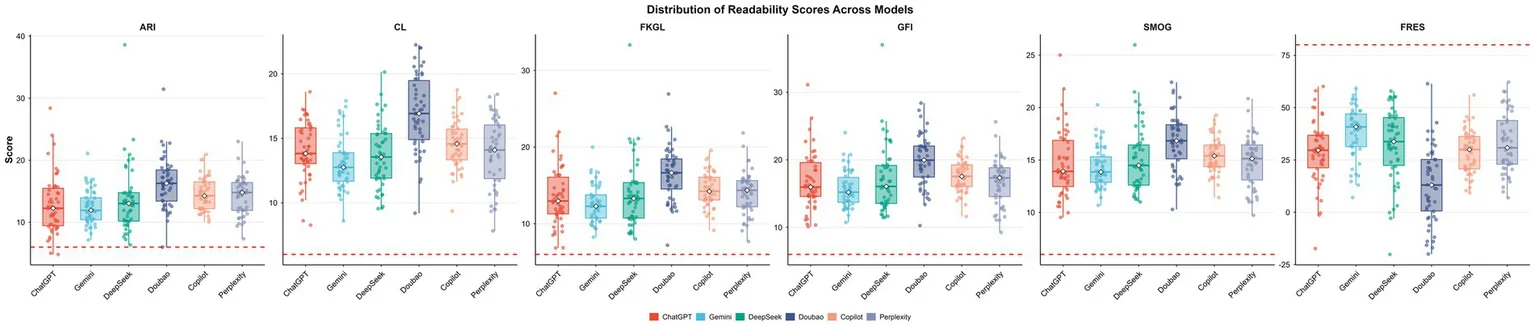
Distribution of readability scores across the six large language model interfaces. Higher values indicate greater reading difficulty for ARI, Coleman–Liau, Flesch–Kincaid Grade Level, Gunning Fog, and SMOG; higher Flesch Reading Ease scores indicate easier reading.

## 4 Discussion

### 4.1 Principal findings

This study provides a multidimensional, patient-facing evaluation of six publicly accessible LLM interfaces across safety, accuracy, empathy, information quality, transparency, global quality, and readability. The central finding is not that one model consistently outperformed the others, but that generally favorable factual performance coexisted with clinically relevant weaknesses in safety escalation, communication, transparency, and reading level. No single interface performed best across all domains, and the between-model differences in several outcomes were small. These results indicate that apparent fluency, accuracy, or a single global score is insufficient to determine whether LLM-generated information is ready for population-facing DR education.

Compared with previous ophthalmology and DR evaluations, the contribution of this study lies in combining a six-interface comparison with CHART-aligned reporting, guideline-based Safety and Accuracy assessment, five-physician rating, structured information-quality instruments, transparency criteria, and six readability measures. This integrated framework exposes cross-domain trade-offs that accuracy-centered evaluations may miss and provides a transparent audit structure that can be replicated as interfaces change. Recent ophthalmology reviews and DR-specific studies similarly show that clinical usefulness cannot be inferred from correctness alone (14, 42). Cross-platform studies in other conditions further demonstrate that favorable information-quality scores do not necessarily coincide with easy readability or high actionability (43–45). Because the present study did not apply PEMAT or another validated actionability instrument, its findings should not be interpreted as a formal assessment of whether patients can act on the information.

The practical significance of the benchmark is therefore to shift attention from identifying a single best-performing chatbot to identifying the patient-facing dimensions that warrant evaluation before clinical or educational implementation. The observed pattern highlights four priorities: reliable escalation for urgent or high-risk scenarios, clear separation of general education from individualized advice, transparent sourcing and uncertainty communication, and language that is understandable to people with varied health literacy. These priorities are relevant to clinicians and health educators considering LLM-assisted education, to health systems developing governance processes, and to developers designing guideline-linked or domain-specific systems. The results support a cautious, clinician-supervised adjunctive role rather than stand-alone use for diagnosis, triage, or treatment decisions.

### 4.2 Safety

The safety findings were generally reassuring, but they should be interpreted in terms of care-seeking and clinical escalation rather than only as a binary outcome. Although most responses were classified as safe, the qualitative review showed that potentially unsafe answers were not mainly caused by dramatic hallucinations. More often, the concern was incomplete escalation or unclear boundary-setting, including vague referral advice, inappropriate self-management, overstatement of supplements or AI-assisted screening, imprecise screening or follow-up intervals, incomplete post-injection warning advice, unrealistic treatment expectations, and insufficient pregnancy-related monitoring.

This issue is particularly important in DR because early disease may be asymptomatic, whereas sudden visual deterioration may indicate sight-threatening complications. A response can therefore be broadly accurate but still clinically unsafe if it reassures the user when urgent ophthalmic evaluation is needed. Although the standardized single-turn design does not reproduce a full conversation, the first response remains a meaningful safety checkpoint because it establishes the initial boundary between reassurance, self-management, and professional referral. First-turn escalation may therefore be regarded as an important initial safety criterion, although multi-turn evaluation remains necessary to determine whether subsequent interaction corrects or amplifies an initial problem. Recent work comparing LLM responses with ophthalmology guidelines found potentially harmful discordance, and physician-led red-teaming identified unsafe omissions in plausible patient-facing answers (46, 47).

### 4.3 Accuracy

Accuracy was generally high across models, and the practical separation between models was limited. This likely reflects the nature of DR patient education: many questions involve stable and widely disseminated information, such as screening, systemic risk-factor control, symptoms of advanced disease, diabetic macular edema, proliferative disease, and common treatment approaches. ChatGPT, Copilot, and Perplexity showed comparatively strong accuracy performance, but the small effect size suggests that these differences should not be overinterpreted as a definitive model ranking. Accordingly, the Accuracy scores should be interpreted as concordance with the study-specific guideline-based reference standard rather than as an independently validated measure of clinical correctness. Because the mapping table was developed and cross-checked by two reviewers without independent third-party adjudication or external validation, subtle guideline-interpretation choices may have influenced the resulting Accuracy ratings.

However, accurate general information should not be equated with individualized clinical reliability. Real-world DR management depends on diabetes duration, glycemic control, blood pressure, renal function, pregnancy status, visual acuity, retinal imaging findings, disease stage, presence of diabetic macular edema, prior treatment, and access to follow-up. These details are usually absent from single-turn patient questions. Studies in diabetes and DR-related LLM applications suggest that retrieval or guideline anchoring may improve response quality, but such improvements do not remove the need for clinical context and professional judgment (10, 14). Future studies should evaluate whether guideline-linked retrieval, structured prompts, and model-generated clarification questions can improve accuracy without increasing overconfidence.

Emerging multimodal approaches—including multi-granular medical image–language learning (48) and ophthalmology-specific vision–language foundation models such as VisionUnite (49)—represent complementary directions to the public-facing text interfaces evaluated in this study. These approaches incorporate image–text supervision, ophthalmic imaging, and domain-specific clinical knowledge, and therefore address technical and clinical tasks that differ from the patient-oriented text-based education evaluated here. Nevertheless, the present findings remain relevant to their patient-facing use because specialized or multimodal systems should also be evaluated for accurate explanation, appropriate escalation, source transparency, understandable language, and avoidance of unsupported individualized recommendations. Future research should determine whether guideline-linked retrieval, clinically constrained generation, and validated multimodal integration improve these dimensions without reducing accessibility or introducing new forms of overconfidence.

### 4.4 Empathy

Empathy showed clearer variation than factual accuracy, suggesting that patient-centered communication is a distinct dimension of LLM performance. Copilot performed relatively well in empathy, whereas Doubao and Perplexity scored lower. This difference may reflect variation in default tone, response length, emotional acknowledgement, and whether the model explicitly recognizes patient concerns before providing factual information. In DR, empathy is clinically relevant because patients may fear blindness, injections, laser treatment, surgery, pregnancy-related worsening, or loss of independence.

Previous studies have reported that AI-generated responses can sometimes be perceived as empathetic in written medical question-answer settings (7, 9). Nevertheless, empathy in medical AI should not be reduced to warmth or reassurance. Over-reassurance may become unsafe if it minimizes sudden symptoms, delays referral, or overstates the reversibility of advanced disease. For DR patient education, the ideal response should acknowledge anxiety, explain the issue clearly, and provide appropriate next steps. Future evaluations should therefore assess “safe empathy,” meaning communication that is supportive and patient-centered while still maintaining clinical caution.

### 4.5 Information quality, reliability, and transparency

The results for DISCERN, EQIP, JAMA benchmark criteria, and GQS show that information quality cannot be fully captured by global impression alone. Perplexity performed relatively well on DISCERN and JAMA, while Copilot, Gemini, and Perplexity performed well on EQIP. In contrast, GQS differences were smaller and did not fully align with structured reliability or transparency metrics. A response may therefore appear fluent, organized, and useful while still lacking adequate source attribution, balanced treatment information, disclosure, currency, or explanation of uncertainty.

The JAMA benchmark was originally developed for static online health information, and its authorship and disclosure criteria may function differently when applied to conversational interfaces. This construct mismatch means that low JAMA scores should not be interpreted as evidence of poor factual performance or deficient intrinsic model capability. However, interface-level transparency remains relevant because it is the information environment users actually encounter. The ability to see sources, dates, and disclosure supports verification and accountability even when generated content is accurate. Perplexity’s higher JAMA scores should therefore be interpreted as an interface-level transparency advantage, not as evidence of superiority in safety, accuracy, empathy, or readability.

Taken together, the discordance among DISCERN, EQIP, JAMA, and GQS indicates that a fluent response may still be difficult to audit. For clinical or public-health implementation, systems should provide traceable sources when possible, distinguish evidence-based general education from individualized advice, state important uncertainty, present treatment benefits and risks in balance, and indicate when information was last reviewed. These features should be evaluated alongside, rather than substituted by, overall quality ratings (35–38, 50).

### 4.6 Readability

Readability remained a shared limitation across models. Gemini generated comparatively more readable responses, whereas Doubao showed greater reading difficulty; however, all models produced text above recommended patient-reading levels. This indicates that readability is not only a model-specific weakness but a broader limitation of LLM-generated medical education. This finding is consistent with prior work showing that freely available online DR information is often difficult to read and may exceed recommended reading levels (51). Similar concerns have also been reported in broader audits of medical chatbot responses (50).

This issue has direct implications for health equity. Patients with DR need to understand asymptomatic disease progression, screening intervals, systemic risk-factor control, warning symptoms, anti-VEGF injections, laser treatment, pregnancy-related risk, and long-term follow-up. If responses require advanced reading ability, they may fail to support patients with limited health literacy. Future patient-facing LLM systems should use shorter sentences, plain-language explanations, clear action-oriented advice, and simple definitions of unavoidable medical terms such as diabetic macular edema, proliferative diabetic retinopathy, anti-VEGF therapy, and panretinal photocoagulation. Readability improvement should not come at the cost of clinical accuracy or safety escalation.

### 4.7 Clinical and public health implications

The findings have practical implications for how LLMs may be incorporated into DR education. They do not support selecting a single consumer chatbot as an autonomous educator on the basis of an overall score or model ranking. A safer implementation model would use LLM-generated text as a draft, navigation aid, or supplementary explanation within clinician- or educator-supervised workflows. Before implementation, services should evaluate guideline concordance, urgent-symptom escalation, boundaries around individualized advice, source display, readability, and documentation of the interface and evaluation date. High-risk content should undergo stronger review or route users directly to professional care.

At a public-health level, LLMs may expand access to repeated education, but the same scale can propagate confusing or inequitable information. All models exceeded recommended reading levels, and English-only testing cannot establish performance for Chinese-speaking or other multilingual populations. Language-specific and culturally adapted validation, health-literacy testing, patient involvement, and monitoring after model or interface updates should therefore be considered important components of pre-implementation evaluation. The multidimensional framework used here can inform pre-implementation and post-update auditing by helping health systems assess not only whether an answer is correct, but whether it is safe, transparent, understandable, and appropriately connected to care (52).

### 4.8 Limitations

This study has several limitations that define the scope of inference. First, the standardized single-turn design improved comparability across platforms and captured the initial response presented to a user, but it did not reproduce iterative conversations. Follow-up interaction could either correct an incomplete first answer or reinforce an early error and elicit increasingly individualized advice; therefore, the direction of bias is uncertain. Second, each question was submitted only once per model. The reported 86.0–94.0% safe-response range and between-model differences therefore represent a time-stamped single-run sample, and within-prompt generative variability was not incorporated into the statistical inference. In addition, public-facing LLM interfaces may change over time, so the findings should not be interpreted as permanent model rankings.

Third, all prompts were submitted in English, limiting generalizability to Chinese-speaking users and other multilingual or culturally adapted settings. Fourth, no validated actionability instrument such as PEMAT was applied; therefore, the study cannot determine whether patients could appropriately act on the information provided. Fifth, the reference-standard mapping table was developed and cross-checked by two reviewers without independent third-party adjudication or external validation. Although the subsequent LLM responses were independently scored by five trained physicians and inter-rater reliability was assessed, these procedures reflect the consistency of outcome scoring rather than independent validation of the reference standard itself. Subtle guideline-interpretation choices made during construction of the mapping table may therefore have influenced the Accuracy ratings. Finally, rater-based measures remained partly subjective, readability formulas did not directly assess comprehension or behavior, and no patients participated in evaluating understanding, trust, or care-seeking.

Future studies should incorporate repeated sampling, matched multi-turn and multilingual evaluation, independently adjudicated or externally validated reference standards, validated actionability measures, patient and public involvement, and outcome-based assessment of whether LLM-assisted education improves understanding and appropriate care-seeking without increasing delay, inequity, or overreliance.

## 5 Conclusion

By demonstrating that generally high factual performance can coexist with gaps in safety escalation, transparency, patient-centered communication, and readability, this study shows why accuracy-only evaluation is insufficient for patient-facing DR education. Its principal contribution is a multidimensional benchmark that translates model comparisons into evaluation and implementation priorities: guideline-linked content, explicit triage and referral boundaries, source transparency, health-literacy adaptation, multilingual validation, clinician oversight, and ongoing re-evaluation after system updates. LLM-generated DR information may complement patient education, but it should not replace professional eye care or be treated as a stand-alone source of diagnosis, triage, or treatment advice.

## Data Availability

The original contributions presented in the study are included in the article/Supplementary material, further inquiries can be directed to the corresponding authors.

## References

[1] Teo ZL Tham YC Yu M Chee ML Rim TH Cheung N . Global prevalence of diabetic retinopathy and projection of burden through 2045: systematic review and meta-analysis. Ophthalmology. (2021) 128:1580–91. doi: 10.1016/j.ophtha.2021.04.027, 33940045

[2] American Diabetes Association Professional Practice Committee for Diabetes. 12. Retinopathy, neuropathy, and foot care: standards of care in diabetes—2026. Diabetes Care. (2026) 49:S261–76. doi: 10.2337/dc26-S012 PMC12690177 41358886

[3] Lim JI Kim SJ Bailey ST Kovach JL Vemulakonda GA Ying GS . Diabetic retinopathy preferred practice pattern®. Ophthalmology. (2025) 132:P75–P162. doi: 10.1016/j.ophtha.2024.12.020 39918521

[4] Jia X Pang Y Liu LS. Online health information seeking behavior: a systematic review. Healthcare (Basel). (2021) 9:1740. doi: 10.3390/healthcare9121740, 34946466 PMC8701665

[5] Cohen SA Pershing S. Readability and accountability of online patient education materials for common retinal diseases. Ophthalmol Retina. (2022) 6:641–3. doi: 10.1016/j.oret.2022.03.015, 35338025 PMC10728491

[6] Rooney MK Santiago G Perni S Horowitz DP McCall AR Einstein AJ . Readability of patient education materials from high-impact medical journals: a 20-year analysis. J Patient Exp. (2021) 8:2374373521998847. doi: 10.1177/2374373521998847 PMC8205335 34179407

[7] Ayers JW Poliak A Dredze M Leas EC Zhu Z Kelley JB . Comparing physician and artificial intelligence chatbot responses to patient questions posted to a public social media forum. JAMA Intern Med. (2023) 183:589–96. doi: 10.1001/jamainternmed.2023.1838, 37115527 PMC10148230

[8] Johnson SB King AJ Warner EL Aneja S Kann BH Bylund CL. Using ChatGPT to evaluate cancer myths and misconceptions: artificial intelligence and cancer information. JNCI Cancer Spectr. (2023) 7:pkad015. doi: 10.1093/jncics/pkad015, 36929393 PMC10020140

[9] Yalamanchili A Sengupta B Song J Lim S Mittal BB Thomas TO . Quality of large language model responses to radiation oncology patient care questions. JAMA Netw Open. (2024) 7:e244630. doi: 10.1001/jamanetworkopen.2024.4630, 38564215 PMC10988356

[10] Wang D Liang J Ye J Li J Li J Zhang Q . Enhancement of the performance of large language models in diabetes education through retrieval-augmented generation: comparative study. J Med Internet Res. (2024) 26:e58041. doi: 10.2196/58041, 39046096 PMC11584532

[11] Lim ZW Pushpanathan K Yew SME Lai Y Sun CH Lam JSH . Benchmarking large language models’ performances for myopia care: a comparative analysis of ChatGPT-3.5, ChatGPT-4.0, and Google bard. EBioMedicine. (2023) 95:104770. doi: 10.1016/j.ebiom.2023.104770, 37625267 PMC10470220

[12] Momenaei B Wakabayashi T Shahlaee A Durrani AF Pandit SA Wang K . Appropriateness and readability of ChatGPT-4-generated responses for surgical treatment of retinal diseases. Ophthalmol Retina. (2023) 7:862–8. doi: 10.1016/j.oret.2023.05.022, 37277096

[13] Chang LC Sun CC Chen TH Tsai DC Lin HL Liao LL. Evaluation of the quality and readability of ChatGPT responses to frequently asked questions about myopia in traditional Chinese language. Digit Health. (2024) 10:20552076241277021. doi: 10.1177/20552076241277021, 39229462 PMC11369861

[14] Wu H Su Z Pan X Shao A Xu Y Wang Y . Enhancing diabetic retinopathy query responses: assessing large language model in ophthalmology. Br J Ophthalmol. (2025) 109:1272–8. doi: 10.1136/bjo-2024-325861, 40588331 PMC12573325

[15] The CHART Collaborative. Reporting guideline for chatbot health advice studies: the CHART statement. JAMA Netw Open. (2025) 8:e2530220. doi: 10.1001/jamanetworkopen.2025.30220 40747871

[16] National Institute for Health and Care Excellence Diabetic Retinopathy: Management and Monitoring. NICE guideline NG242 London National Institute for Health and Care Excellence (2024) Available online at: https://www.nice.org.uk/guidance/ng242 (Accessed July 19, 2026).

[17] Blonde L Umpierrez GE Reddy SS McGill JB Berga SL Bush M . American Association of Clinical Endocrinology Clinical Practice Guideline: developing a diabetes mellitus comprehensive care plan—2022 update. Endocr Pract. (2022) 28:923–1049. doi: 10.1016/j.eprac.2022.08.002, 35963508 PMC10200071

[18] Fundus Disease Group of Ophthalmological Society of Chinese Medical Association, Fundus Disease Group of Ophthalmologist Branch of Chinese Medical Doctor Association. Evidence-based guidelines for diagnosis and treatment of diabetic retinopathy in China (2022). Chin J Ocul Fundus Dis. (2023) 39:99–124. doi: 10.3760/cma.j.cn511434-20230110-00018

[19] Ng DSC Ruamviboonsuk P Apte RS Bajimaya S Chan CKM Chang A . International consensuses and controversies on causes, diagnosis and management of diabetic macular edema (DME). Prog Retin Eye Res. (2025) 109:101406. doi: 10.1016/j.preteyeres.2025.101406, 41005472

[20] Lam RF Radke NV Bandello FM Bhende P Chen YT Cheung GCM . International consensuses and guidelines on management of proliferative diabetic retinopathy (PDR) by the Asia-Pacific Vitreo-retina society (APVRS), the academy of Asia-Pacific professors of ophthalmology (AAPPO), and the academia retina Internationalis (ARI). Asia Pac J Ophthalmol. (2025) 14:100262. doi: 10.1016/j.apjo.2025.100262, 41207388

[21] Yonekawa Y Modi YS Kim LA Skondra D Kim JE Wykoff CC. American Society of Retina Specialists clinical practice guidelines: management of nonproliferative and proliferative diabetic retinopathy without diabetic macular edema. J Vitreoretin Dis. (2020) 4:125–35. doi: 10.1177/2474126419893829, 34308094 PMC8297841

[22] Horton MB Brady CJ Cavallerano J Abramoff M Barker G Chiang MF . Practice guidelines for ocular telehealth-diabetic retinopathy, third edition. Telemed J E Health. (2020) 26:495–543. doi: 10.1089/tmj.2020.0006, 32209018 PMC7187969

[23] Wong TY Sun J Kawasaki R Ruamviboonsuk P Gupta N Lansingh VC . Guidelines on diabetic eye care: the International Council of Ophthalmology recommendations for screening, follow-up, referral, and treatment based on resource settings. Ophthalmology. (2018) 125:1608–22. doi: 10.1016/j.ophtha.2018.04.007 29776671

[24] Early Treatment Diabetic Retinopathy Study Research Group. Photocoagulation for diabetic macular edema: early treatment diabetic retinopathy study report number 1. Arch Ophthalmol. (1985) 103:1796–806. doi: 10.1001/archopht.1985.01050120030015 2866759

[25] Diabetic Retinopathy Study Research Group. Photocoagulation treatment of proliferative diabetic retinopathy: clinical application of diabetic retinopathy study findings, DRS report number 8. Ophthalmology. (1981) 88:583–600. doi: 10.1016/S0161-6420(81)34978-1 7196564

[26] Wells JA Glassman AR Ayala AR Jampol LM Aiello LP Antoszyk AN . Aflibercept, bevacizumab, or ranibizumab for diabetic macular edema. N Engl J Med. (2015) 372:1193–203. doi: 10.1056/NEJMoa1414264 PMC4422053 25692915

[27] Wells JA Glassman AR Ayala AR Jampol LM Bressler NM Bressler SB . Aflibercept, bevacizumab, or ranibizumab for diabetic macular edema: two-year results from a comparative effectiveness randomized clinical trial. Ophthalmology. (2016) 123:1351–9. doi: 10.1016/j.ophtha.2016.02.022 PMC4877252 26935357

[28] Gross JG Glassman AR Jampol LM Inusah S Aiello LP Antoszyk AN . Panretinal photocoagulation vs intravitreous ranibizumab for proliferative diabetic retinopathy: a randomized clinical trial. JAMA. (2015) 314:2137–46. doi: 10.1001/jama.2015.15217, 26565927 PMC5567801

[29] Gross JG Glassman AR Liu D Sun JK Antoszyk AN Baker CW . Five-year outcomes of panretinal photocoagulation vs intravitreous ranibizumab for proliferative diabetic retinopathy: a randomized clinical trial. JAMA Ophthalmol. (2018) 136:1138–48. doi: 10.1001/jamaophthalmol.2018.3255, 30043039 PMC6233839

[30] Brown DM Wykoff CC Boyer D Heier JS Clark WL Emanuelli A . Evaluation of intravitreal aflibercept for the treatment of severe nonproliferative diabetic retinopathy: results from the PANORAMA randomized clinical trial. JAMA Ophthalmol. (2021) 139:946–55. doi: 10.1001/jamaophthalmol.2021.2809, 34351414 PMC8343518

[31] Nguyen QD Brown DM Marcus DM Boyer DS Patel S Feiner L . Ranibizumab for diabetic macular edema: results from 2 phase III randomized trials: RISE and RIDE. Ophthalmology. (2012) 119:789–801. doi: 10.1016/j.ophtha.2011.12.039 22330964

[32] Korobelnik JF Do DV Schmidt-Erfurth U Boyer DS Holz FG Heier JS . Intravitreal aflibercept for diabetic macular edema. Ophthalmology. (2014) 121:2247–54. doi: 10.1016/j.ophtha.2014.05.006, 25012934

[33] Abràmoff MD Lavin PT Birch M Shah N Folk JC. Pivotal trial of an autonomous AI-based diagnostic system for detection of diabetic retinopathy in primary care offices. NPJ Digit Med. (2018) 1:39. doi: 10.1038/s41746-018-0040-6, 31304320 PMC6550188

[34] Rajesh AE Davidson OQ Lee CS Lee AY. Artificial intelligence and diabetic retinopathy: AI framework, prospective studies, head-to-head validation, and cost-effectiveness. Diabetes Care. (2023) 46:1728–39. doi: 10.2337/dci23-0032, 37729502 PMC10516248

[35] Charnock D Shepperd S Needham G Gann R. DISCERN: an instrument for judging the quality of written consumer health information on treatment choices. J Epidemiol Community Health. (1999) 53:105–11. doi: 10.1136/jech.53.2.105, 10396471 PMC1756830

[36] Moult B Franck LS Brady H. Ensuring quality information for patients: development and preliminary validation of a new instrument to improve the quality of written health care information. Health Expect. (2004) 7:165–75. doi: 10.1111/j.1369-7625.2004.00273.x, 15117391 PMC5060233

[37] Silberg WM Lundberg GD Musacchio RA. Assessing, controlling, and assuring the quality of medical information on the internet: caveant lector et viewor—let the reader and viewer beware. JAMA. (1997) 277:1244–5. doi: 10.1001/jama.1997.03540390074039 9103351

[38] Bernard A Langille M Hughes S Rose C Leddin D van Zanten SV. A systematic review of patient inflammatory bowel disease information resources on the world wide web. Am J Gastroenterol. (2007) 102:2070–7. doi: 10.1111/j.1572-0241.2007.01325.x, 17511753

[39] Readability Formulas (n.d). Readability Calculator. Available online at: https://readabilityformulas.com/ (Accessed April 16, 2026).

[40] Weiss BD. Health Literacy and patient Safety: help Patients Understand. Manual for Clinicians. 2nd ed. Chicago, IL: American Medical Association Foundation (2007).

[41] Eltorai AEM Ghanian S Adams CA Jr Born CT Daniels AH. Readability of patient education materials on the American Association for Surgery of trauma website. Arch Trauma Res. (2014) 3:e18161. doi: 10.5812/atr.18161, 25147778 PMC4139691

[42] Zhang Z Zhang H Pan Z Bi Z Wan Y Song X . Evaluating large language models in ophthalmology: systematic review. J Med Internet Res. (2025) 27:e76947. doi: 10.2196/76947, 41144954 PMC12603593

[43] Deng B Wang Z Cheng T Zhang Z Li M. Cross-platform evaluation of LLM-generated educational texts on cardiac myxoma: quality, readability, and actionability using network analysis and latent profile analysis. Front Cardiovasc Med. (2026) 13:1828810. doi: 10.3389/fcvm.2026.1828810, 42358633 PMC13290900

[44] Wang Z Li X Ma C Zhang Z. Challenges of using generative AI for patient education in chronic heart failure: an evaluation of content quality, readability, and actionability in cross-platform LLM-generated texts. Front Public Health. (2026) 14:1801829. doi: 10.3389/fpubh.2026.1801829, 41869585 PMC12999856

[45] Hu M Wang Z Zhang Z Li M. Challenges of patient-facing generative artificial intelligence in hypertension care: a cross-platform evaluation of the quality, readability, and actionability of LLM-generated patient education materials. Digit Health. (2026) 12:20552076261464724. doi: 10.1177/20552076261464724, 42404600 PMC13332286

[46] Taloni A Sangregorio AC Alessio G Romeo MA Coco G Busin LML . Large language models provide discordant information compared to ophthalmology guidelines. Sci Rep. (2025) 15:20556. doi: 10.1038/s41598-025-06404-z, 40596239 PMC12218404

[47] Draelos RL Afreen S Blasko B Brazile TL Chase N Desai DP . Large language models provide unsafe answers to patient-posed medical questions. NPJ Digit Med. (2026) 9:241. doi: 10.1038/s41746-026-02428-5, 41688533 PMC13013898

[48] Li Z Wang Y Farsiu S Kinahan PE. Boosting medical visual understanding from multi-granular language learning. arXiv. (2025):2511.15943. doi: 10.48550/arXiv.2511.15943

[49] Li Z Song D Yang Z Wang D Li F Zhang X . VisionUnite: a vision-language foundation model for ophthalmology enhanced with clinical knowledge. IEEE Trans Pattern Anal Mach Intell. (2025) 47:11848–62. doi: 10.1109/TPAMI.2025.3598734, 40802643

[50] Tiller NB Marcon AR Zenone M Kidd KE Jeukendrup AE Master Z . Generative artificial intelligence-driven chatbots and medical misinformation: an accuracy, referencing and readability audit. BMJ Open. (2026) 16:e112695. doi: 10.1136/bmjopen-2025-112695, 41980854 PMC13158598

[51] Kloosterboer A Yannuzzi NA Patel NA Kuriyan AE Sridhar J. Assessment of the quality, content, and readability of freely available online information for patients regarding diabetic retinopathy. JAMA Ophthalmol. (2019) 137:1240–5. doi: 10.1001/jamaophthalmol.2019.3116, 31436789 PMC6707011

[52] Bedi S Liu Y Orr-Ewing L Dash D Koyejo S Callahan A . Testing and evaluation of health care applications of large language models: a systematic review. JAMA. (2025) 333:319–28. doi: 10.1001/jama.2024.21700 PMC11480901 39405325

