# Supplementary material for: Large language models as sources of diabetic retinopathy patient education: a comparative evaluation of safety, health information quality, and readability
Source: Front Public Health. 2026 Oct 9;14:1945318. doi: 10.3389/fpubh.2026.1945318 (PMC13690771; doi:10.3389/fpubh.2026.1945318)
Supplement: Supplementary file 1 [file Supplementary_file_1.docx]

**Supplementary Material 1. Completed CHART Checklist**

| **Heading** | **No** | **CHART checklist item** | **Page No / location** |
| --- | --- | --- | --- |
| **Title and abstract** | | | |
| Title | 1a | State that the study is assessing one or more generative AI-driven chatbots for clinical evidence or health advice. | p. 1 (Title) |
| Abstract/summary | 1b | Apply a structured format, if applicable. | p. 1 (Abstract) |
| **Introduction** | | | |
| Background | 2a | State the scientific background, rationale, and healthcare context for evaluating the generative AI-driven chatbot(s), referencing relevant literature when applicable. | p. 2 (Introduction) |
|  | 2b | State the aims and research questions including the target audience, intervention, comparator(s), and outcome(s). | p. 2 (Introduction, final paragraph) |
| **Methods** | | | |
| Model identifiers | 3a | State the name and version identifier(s) of the generative AI model(s) and chatbot(s) under evaluation, as well as their date of release or last update. | p. 3, Section 2.4; SM4 Table S4-1 (release/update dates not visible in the public interfaces) |
|  | 3b | State whether the generative AI model(s) and chatbot(s) are open-source or closed-source/proprietary. | SM4 Table S4-1 |
| Model details | 4a | State whether the generative AI model was a base model or a novel base model, tuned model, or fine-tuned model. | SM4 Table S4-1 (model architecture/tuning status not disclosed in the public interfaces) |
|  | 4b | If a base model is used, cite its development in sufficient detail to identify the model. | N/A; SM4 Table S4-1 |
|  | 4c | If a novel base model, tuned model, or fine-tuned model is used, describe the pre- and/or post-implementation/deployment data and parameters. | N/A; SM4 Table S4-1 |
| Prompt engineering | 5a | Describe the evolution of study prompt development. | pp. 2-3, Section 2.2; SM2 |
|  | 5ai | Describe the sources of prompts. | pp. 2-3, Section 2.2; SM2 |
|  | 5aii | State the number and characteristics of the individual(s) involved in prompt engineering. | p. 3, Section 2.2 |
|  | 5aiii | Provide details of any patient and public involvement during prompt engineering. | p. 3, Section 2.2 |
|  | 5b | Provide study prompts. | p. 3, Section 2.2; SM2 Table S2-3; SM4 Section S4-4 |
| Query strategy | 6a | State route of access to generative AI model. | p. 3, Section 2.4; SM4 Table S4-1 |
|  | 6b | State the date(s) and location(s) of queries for the generative AI-driven chatbot(s), including the day, month, and year as well as city and country. | p. 3, Section 2.4 |
|  | 6c | Describe whether prompts were input into separate chat session(s). | p. 3, Section 2.4; SM4 Table S4-2 |
|  | 6d | Provide all generative AI-driven chatbot output/responses. | p. 3, Section 2.4; SM4 Section S4-4 |
| Performance evaluation | 7a | Define the ground truth or reference standard used to define successful generative AI-driven chatbot performance. | p. 3, Section 2.3; SM3 |
|  | 7b | Describe the process undertaken for generative AI-driven chatbot performance evaluation. | pp. 3-4, Sections 2.5-2.6 |
|  | 7bi | State the number and characteristics of team members involved in performance evaluation. | p. 3, Section 2.5 |
|  | 7bii | Provide details of any patients and public involvement during the evaluation process. | p. 3, Section 2.5 |
|  | 7biii | State whether evaluators were blinded to the identity of the generative AI-driven chatbot(s) under assessment. | p. 3, Section 2.5 |
| Sample size | 8 | Report how the sample size was determined. | p. 3, Section 2.2 |
| Data analysis | 9a | Describe statistical analysis methods, including any evaluation of reproducibility of generative AI-driven chatbot responses. | p. 4, Section 2.7; SM5 (response reproducibility across repeated model runs was not evaluated) |
|  | 9ai | Report the measures used for performance evaluation. | p. 4, Section 2.6 |
| **Results** | | | |
|  | 10a | Report the performance evaluation undertaken, including the alignment between generative AI-driven chatbot output and ground truth or reference standard using quantitative or mixed methods approaches as applicable. | pp. 4-6, Sections 3.1-3.4; SM5 |
|  | 10b | For responses deviating from the ground truth or reference standard, state the nature of the difference(s). | p. 5, Section 3.2; SM6 |
|  | 10c | Report the evaluation for potentially harmful, biased, or misleading responses. | p. 5, Section 3.2; SM6 |
| **Discussion** | | | |
|  | 11a | Interpret study findings in the context of relevant evidence. | pp. 5-9, Discussion |
|  | 11b | Describe the strengths and limitations of the study. | pp. 5-9, Discussion; Section 4.8 (Limitations) |
|  | 11c | Describe the potential implications for practice, education, policy, regulation, and research. | pp. 7-9, Discussion; p. 9 (Conclusion) |
| **Open science** | | | |
| Disclosures | 12a | Report any relevant conflicts of interest for all authors. | p. 9 (Conflict of interest) |
| Funding | 12b | Report sources of funding and their role in the conduct and reporting of the study. | p. 9 (Funding) |
| Ethics | 12c | Describe the process undertaken for ethical approval. | p. 9 (Ethics statement) |
|  | 12ci | Describe the measures taken to safeguard data privacy of patient health information, as applicable. | p. 2, Section 2.1; p. 9 (Ethics statement) |
|  | 12cii | State whether permission/licensing was obtained for the use of original, copyrighted data. | p. 9 (Ethics statement); SM4 (raw model outputs shared only where permitted by platform terms; formal licensing status not separately reported) |
| Protocol | 12d | Provide a study protocol. | No separate protocol; pp. 2-4 (Methods); SM2-SM5 |
| Data availability | 12e | State where study data, code repository, and model parameters can be accessed. | p. 9 (Data availability statement); SM4; data and code available from the corresponding author upon reasonable request |
