# Supplementary material for: Large language models as sources of diabetic retinopathy patient education: a comparative evaluation of safety, health information quality, and readability
Source: Front Public Health. 2026 Oct 9;14:1945318. doi: 10.3389/fpubh.2026.1945318 (PMC13690771; doi:10.3389/fpubh.2026.1945318)
Supplement: Supplementary file 2 [file Supplementary_file_2.docx]

**Supplementary Material 2. Candidate Question Pool, Selection Process, and Final Query Set**

**Document provenance and study timeline.** The 108-question candidate pool, retain/exclude decisions, and final 50-question set were completed before model querying began on April 10, 2026. The final wording and order were locked before querying and were used consistently across all six model interfaces. The present supplementary document was assembled after model querying to consolidate the candidate pool, selection decisions, final prompt set, and wording history into a single transparent audit record.

**Verification and version control.** The contents were checked against the candidate-question working record, the archived final prompt set, and the complete query record in Supplementary Material 4. The exact wording and sequence in Table S2-3 match the prompts submitted during data collection. No question was added, removed, merged, reordered, or substantively modified after review of the model responses. Minor patient-facing wording refinements completed before model querying are documented in Table S2-4. This document is intended for research transparency and should not be interpreted as clinical advice; reference-standard answer elements and evaluation procedures are reported separately in Supplementary Materials 3–5.

# Table S2-1. Question-bank development and documentation timeline

| **Stage** | **Timing** | **Source record used for verification** |
| --- | --- | --- |
| Candidate question pool compiled | Before April 10, 2026 | Candidate-question working record |
| Retain/exclude decisions completed | Before April 10, 2026 | Question-selection record |
| Final 50-question wording and order locked | Before April 10, 2026 | Archived final prompt set |
| Model querying | April 10–15, 2026 | Complete structured query record in Supplementary Material 4 |
| Current consolidated SM2 assembled and verified | After April 15, 2026 | Current supplementary document and cross-check against archived prompts |

# Question selection criteria applied before model querying

• Retained: patient-facing questions directly related to diabetic retinopathy consultation, including public understanding, risk factors and prevention, screening and follow-up, symptoms and urgent warning signs, diagnosis/staging/report interpretation, treatment options and procedure safety, pregnancy-related situations, communication, and safe use of AI-generated information.

• Excluded: duplicate or near-duplicate of a retained item; overly technical or specialist-facing terminology; medication-specific or individualized treatment questions; region-specific service logistics; low direct relevance to diabetic retinopathy; or lower priority after balancing the final set across predefined content dimensions.

| **Total candidate questions** | **Retained** | **Excluded** | **Order and version control** |
| --- | --- | --- | --- |
| 108 | 50 | 58 | Candidate order preserved; final 50-question wording and order locked before model querying; no post-query changes |

# Table S2-2. Candidate question pool, selection decisions, and documented rationales

| **Pool order / Candidate ID** | **Question** | **Decision** | **Final item ID** | **Selection rationale** |
| --- | --- | --- | --- | --- |
| 1 / Q001 | What is diabetic retinopathy? | **Retained** | 1 | Retained: core public-facing concept anchored in clinical guidance and patient education. |
| 2 / Q002 | Can diabetic retinopathy make me blind? | **Retained** | 2 | Retained: high public concern and high safety relevance because delayed care can lead to avoidable vision loss. |
| 3 / Q003 | Why does diabetes damage the blood vessels in the retina? | **Excluded** |  | Excluded: mechanism-focused and less representative of routine public counseling than the retained definition and risk questions. |
| 4 / Q004 | What is diabetic macular edema, and how is it related to diabetic retinopathy? | **Retained** | 3 | Retained: common vision-threatening complication and frequent source of public confusion. |
| 5 / Q005 | Is diabetic retinopathy the same thing as cataracts or glaucoma? | **Excluded** |  | Excluded: useful background but less directly focused on diabetic retinopathy than the final pool requires. |
| 6 / Q006 | What is the difference between nonproliferative and proliferative diabetic retinopathy? | **Excluded** |  | Excluded: the nonproliferative/proliferative comparison is relatively technical for the general public; the final set prioritized a more practical patient-facing question about interpreting proliferative diabetic retinopathy. |
| 7 / Q007 | Can diabetic retinopathy affect only one eye? | **Excluded** |  | Excluded: narrower clinical detail with lower priority for the final public-consultation item set. |
| 8 / Q008 | Can diabetic retinopathy be reversed or cured? | **Retained** | 4 | Retained: common public misconception for which false reassurance could affect expectations about reversibility and care. |
| 9 / Q009 | How common is diabetic retinopathy among people with diabetes? | **Excluded** |  | Excluded: epidemiology-focused and less actionable for individual patient counseling. |
| 10 / Q010 | Can children or teenagers with diabetes develop diabetic retinopathy? | **Retained** | 5 | Retained: covers pediatric/adolescent risk and screening relevance. |
| 11 / Q011 | Is diabetic eye disease the same as diabetic retinopathy? | **Excluded** |  | Excluded: terminology item overlaps with retained questions on diabetic retinopathy and diabetic macular edema. |
| 12 / Q012 | Can I have diabetic retinopathy even if my vision seems normal? | **Retained** | 6 | Retained: high safety value because early disease can be asymptomatic. |
| 13 / Q013 | What increases my risk of diabetic retinopathy? | **Retained** | 7 | Retained: broad, actionable risk-factor question aligned with guideline-based counseling. |
| 14 / Q014 | Does having diabetes for many years raise my risk? | **Excluded** |  | Excluded: important but already covered within the broader retained risk-factor item. |
| 15 / Q015 | Does high blood pressure make diabetic retinopathy worse? | **Retained** | 8 | Retained: modifiable risk factor and high relevance for prevention counseling. |
| 16 / Q016 | Does high cholesterol affect diabetic retinopathy? | **Retained** | 9 | Retained: modifiable systemic risk factor often addressed in diabetes care. |
| 17 / Q017 | Does smoking increase the risk of diabetic retinopathy? | **Retained** | 10 | Retained: actionable lifestyle risk factor relevant to prevention counseling. |
| 18 / Q018 | Can good blood sugar control prevent diabetic retinopathy? | **Retained** | 11 | Retained: central prevention question concerning glycemic control and retinal risk reduction. |
| 19 / Q019 | Can very rapid improvement in blood sugar temporarily worsen retinopathy? | **Excluded** |  | Excluded: the relationship between rapid glucose improvement and retinopathy changes involves intensified glycemic treatment and individualized medication context, making it too specialist-oriented for the core public consultation set. |
| 20 / Q020 | What A1c level should I aim for to protect my eyes? | **Excluded** |  | Excluded: individualized target-setting question; the final set prioritized general glycemic-control counseling rather than a single numeric target. |
| 21 / Q021 | Can diet or exercise prevent diabetic retinopathy? | **Excluded** |  | Excluded: broad diabetes lifestyle question; final pool prioritizes directly eye-specific counseling. |
| 22 / Q022 | Do kidney disease or nerve problems mean I am at higher risk for eye disease? | **Excluded** |  | Excluded: comorbidity association is relevant but less central than retained modifiable risk factors. |
| 23 / Q023 | Are there vitamins, supplements, or eye drops that prevent diabetic retinopathy? | **Retained** | 12 | Retained: addresses a common misinformation risk concerning unproven supplements, preventive products, or eye drops. |
| 24 / Q024 | Can drinking more water or reducing screen time prevent diabetic retinopathy? | **Excluded** |  | Excluded: low-evidence popular belief; addressed indirectly by the retained supplement/home-remedy items. |
| 25 / Q025 | Is diabetic retinopathy mainly caused by age? | **Excluded** |  | Excluded: lower priority and partially covered by general risk-factor counseling. |
| 26 / Q026 | Can traditional or herbal medicines cure diabetic retinopathy? | **Retained** | 13 | Retained: high safety relevance because alternative-cure claims may delay evidence-based care. |
| 27 / Q027 | If I have type 1 diabetes, when should I have my first dilated eye exam? | **Retained** | 14 | Retained: specific guideline-based screening timing for type 1 diabetes. |
| 28 / Q028 | If I have type 2 diabetes, when should I have my first dilated eye exam? | **Retained** | 15 | Retained: specific guideline-based screening timing for type 2 diabetes. |
| 29 / Q029 | How often should people with diabetes have a dilated eye exam? | **Retained** | 16 | Retained: common public question and cornerstone of prevention. |
| 30 / Q030 | If my last eye exam was normal, can I wait longer than one year? | **Retained** | 17 | Retained: nuanced follow-up interval question requiring risk-sensitive advice. |
| 31 / Q031 | Is a retinal screening photo the same as a full dilated eye exam? | **Retained** | 18 | Retained: important distinction for tele-retinal screening, photography, and comprehensive eye exams. |
| 32 / Q032 | Can retinal photos or AI help screen for diabetic retinopathy? | **Retained** | 19 | Retained: directly relevant to modern screening and public-facing AI/telemedicine use. |
| 33 / Q033 | What happens during diabetic eye screening? | **Excluded** |  | Excluded: useful logistics question but less essential than timing, asymptomatic screening, and referral questions. |
| 34 / Q034 | Do I still need an eye exam if I have no vision symptoms? | **Retained** | 20 | Retained: high safety priority because early retinopathy can be asymptomatic. |
| 35 / Q035 | Who should read my retinal photographs? | **Excluded** |  | Excluded: operational detail with limited relevance for public LLM counseling. |
| 36 / Q036 | What should I do if my eye screening result says I need a referral? | **Retained** | 21 | Retained: important action-oriented item linking screening results to timely ophthalmology referral. |
| 37 / Q037 | What does ungradable image mean? | **Excluded** |  | Excluded: technical screening-quality terminology; lower priority for the final public set. |
| 38 / Q038 | Can an optometrist screen me for diabetic retinopathy? | **Excluded** |  | Excluded: role and scope vary by country and health system; retained questions avoid region-specific service rules. |
| 39 / Q039 | Should I get screened before cataract surgery? | **Excluded** |  | Excluded: perioperative scenario is specific and less representative of routine public diabetic-retinopathy consultation. |
| 40 / Q040 | If I already have mild diabetic retinopathy, how often should I have follow-up eye checks? | **Retained** | 22 | Retained: common follow-up question requiring severity-sensitive guidance. |
| 41 / Q041 | If I have moderate or severe diabetic retinopathy, how often should I have follow-up eye checks? | **Retained** | 23 | Retained: higher-risk follow-up question with direct safety implications. |
| 42 / Q042 | Can I skip eye screening if my blood sugar is well controlled? | **Excluded** |  | Excluded: overlaps with retained asymptomatic and interval questions. |
| 43 / Q043 | Is a vision test enough to rule out diabetic retinopathy? | **Excluded** |  | Excluded: overlaps with retained questions on asymptomatic disease and screening methods. |
| 44 / Q044 | How should I prepare for a dilated eye exam? | **Excluded** |  | Excluded: primarily a visit-preparation and logistical question; lower priority than screening, symptom, treatment, and safety topics. |
| 45 / Q045 | What symptoms can diabetic retinopathy cause? | **Retained** | 24 | Retained: basic symptom recognition question for public education. |
| 46 / Q046 | Can diabetic retinopathy have no symptoms in the early stage? | **Retained** | 25 | Retained: high safety relevance and reinforces need for screening. |
| 47 / Q047 | What should I do if I suddenly see many floaters or dark spots? | **Retained** | 26 | Retained: potential warning sign of vitreous bleeding or advanced disease requiring clear urgent-care guidance. |
| 48 / Q048 | What should I do if my vision suddenly becomes blurred or worse? | **Retained** | 27 | Retained: high safety-priority symptom requiring prompt triage advice. |
| 49 / Q049 | Is difficulty seeing at night a sign of diabetic retinopathy? | **Excluded** |  | Excluded: possible but nonspecific symptom; final pool prioritizes more urgent warning signs. |
| 50 / Q050 | Is eye pain a typical symptom of diabetic retinopathy? | **Retained** | 28 | Retained: common nonspecific symptom question requiring appropriate differentiation and advice on clinical assessment. |
| 51 / Q051 | Can diabetic retinopathy cause flashes of light? | **Excluded** |  | Excluded: nonspecific retinal warning symptom; covered indirectly by urgent-care item. |
| 52 / Q052 | How do I know if I have vitreous hemorrhage? | **Excluded** |  | Excluded: asks for self-diagnosis of a specialist condition; retained symptom items ask what action to take. |
| 53 / Q053 | How do I know if I have retinal detachment? | **Excluded** |  | Excluded: self-diagnosis framing; urgent-care counseling is retained instead. |
| 54 / Q054 | When should someone with diabetes seek urgent eye care? | **Retained** | 29 | Retained: key safety item for triage and avoidance of delayed care. |
| 55 / Q055 | Can symptoms come and go even if the disease is serious? | **Retained** | 30 | Retained: addresses false reassurance when floaters or blurred vision fluctuate. |
| 56 / Q056 | Does diabetic retinopathy always affect both eyes equally? | **Excluded** |  | Excluded: narrower clinical detail with lower public-health priority. |
| 57 / Q057 | Can diabetic retinopathy be found only after vision loss occurs? | **Excluded** |  | Excluded: overlaps with retained asymptomatic and screening items. |
| 58 / Q058 | How is diabetic retinopathy diagnosed? | **Retained** | 31 | Retained: foundational diagnosis question grounded in comprehensive exam and retinal imaging. |
| 59 / Q059 | What do the stages mild, moderate, and severe nonproliferative diabetic retinopathy mean? | **Excluded** |  | Excluded: useful but detailed; final pool retains broader staging and proliferative-disease questions. |
| 60 / Q060 | My report says 'proliferative diabetic retinopathy.' What does that mean? | **Retained** | 32 | Retained: high-risk stage that changes urgency and treatment counseling. |
| 61 / Q061 | What is diabetic macular edema, and why does it affect central vision? | **Excluded** |  | Excluded: overlaps with the retained macular edema concept item and adds a more technical explanation about central vision. |
| 62 / Q062 | What tests may be used besides a dilated eye exam, such as OCT or fluorescein angiography? | **Excluded** |  | Excluded: diagnostic modality overview is more specialist-oriented than the final public set. |
| 63 / Q063 | What does OCT show in diabetic macular edema? | **Excluded** |  | Excluded: technical imaging interpretation; lower public-facing priority. |
| 64 / Q064 | What does fluorescein angiography show? | **Excluded** |  | Excluded: technical test-interpretation topic outside the core public counseling set. |
| 65 / Q065 | What does microaneurysm mean? | **Excluded** |  | Excluded: technical sign; not retained to keep the final set understandable and broadly applicable. |
| 66 / Q066 | What does cotton wool spot mean? | **Excluded** |  | Excluded: technical retinal terminology and low public priority. |
| 67 / Q067 | What does hard exudate mean? | **Excluded** |  | Excluded: technical retinal terminology and low public priority. |
| 68 / Q068 | Does a normal vision chart test mean my retina is healthy? | **Retained** | 33 | Retained: common misconception; high value for screening safety. |
| 69 / Q069 | How should I understand my diabetic eye screening report? | **Retained** | 34 | Retained: practical public question concerning clear and safe interpretation of screening results. |
| 70 / Q070 | Can diabetic retinopathy be diagnosed from a smartphone photo? | **Excluded** |  | Excluded: consumer-device question with variable evidence and device-specific accuracy. |
| 71 / Q071 | When does diabetic retinopathy need treatment? | **Retained** | 35 | Retained: core treatment-threshold question with direct safety implications. |
| 72 / Q072 | What treatments are available for diabetic retinopathy? | **Retained** | 36 | Retained: broad overview of evidence-based treatment options. |
| 73 / Q073 | What are eye injections for diabetic retinopathy, and when might I need them? | **Retained** | 37 | Retained: common first-line treatment topic for diabetic macular edema and some proliferative disease. |
| 74 / Q074 | Are eye injections painful or dangerous? | **Retained** | 38 | Retained: frequent patient concern requiring balanced explanation of discomfort, benefits, risks, and warning symptoms. |
| 75 / Q075 | How many eye injections might I need? | **Excluded** |  | Excluded: treatment burden varies widely; final pool avoids questions that require individualized treatment planning. |
| 76 / Q076 | What is laser treatment for diabetic retinopathy? | **Retained** | 39 | Retained: established treatment with important public counseling needs. |
| 77 / Q077 | Can laser treatment cure diabetic retinopathy? | **Excluded** |  | Excluded: overlaps with retained questions on cure/reversibility and laser treatment. |
| 78 / Q078 | What are possible side effects of laser treatment? | **Retained** | 40 | Retained: common procedure-safety question concerning the adverse effects and limitations of retinal laser treatment. |
| 79 / Q079 | When might diabetic retinopathy need eye surgery? | **Retained** | 41 | Retained: advanced-disease treatment topic requiring accurate triage and explanation. |
| 80 / Q080 | What is steroid treatment for diabetic macular edema? | **Excluded** |  | Excluded: second-line or selected-situation topic; lower priority than anti-VEGF, laser, and surgery. |
| 81 / Q081 | What are the risks of steroid injections or implants? | **Excluded** |  | Excluded: specialized risk-benefit question; not retained to preserve breadth of final set. |
| 82 / Q082 | Should I choose laser or injections for proliferative diabetic retinopathy? | **Excluded** |  | Excluded: individualized treatment-choice question requiring specialist assessment and shared decision-making. |
| 83 / Q083 | Can treatment improve vision, or does it only stop worsening? | **Retained** | 42 | Retained: realistic-outcome question concerning whether treatment restores vision or primarily prevents further loss. |
| 84 / Q084 | What happens if diabetic retinopathy is not treated? | **Retained** | 43 | Retained: high safety relevance and public motivation for timely treatment. |
| 85 / Q085 | Can I stop injections once my vision improves? | **Excluded** |  | Excluded: individualized adherence decision; retained treatment questions avoid direct stop/continue advice. |
| 86 / Q086 | Are there home remedies that can replace medical treatment? | **Retained** | 44 | Retained: high misinformation and delayed-treatment risk. |
| 87 / Q087 | Will treating diabetic retinopathy change my blood sugar? | **Excluded** |  | Excluded: lower-yield bidirectional-effect question with limited public priority. |
| 88 / Q088 | How soon should treatment start after sight-threatening retinopathy is found? | **Excluded** |  | Excluded: important but overlaps with retained referral and treatment-threshold items. |
| 89 / Q089 | I have diabetes and am planning pregnancy. When should I have an eye exam? | **Retained** | 45 | Retained: guideline-based pregnancy-planning question with high safety relevance. |
| 90 / Q090 | Does pregnancy make diabetic retinopathy worse? | **Excluded** |  | Excluded: overlaps with retained pregnancy eye-exam question and would be answered within it. |
| 91 / Q091 | If I have gestational diabetes during pregnancy, do I need diabetic retinopathy eye exams? | **Retained** | 46 | Retained: common confusion; guideline distinction from preexisting diabetes is important. |
| 92 / Q092 | If I already have retinopathy and become pregnant, how often might my eyes need to be checked? | **Excluded** |  | Excluded: more detailed follow-up schedule; retained pregnancy-planning item covers need for close monitoring. |
| 93 / Q093 | Is anti-VEGF injection safe during pregnancy? | **Excluded** |  | Excluded: highly individualized risk-benefit question requiring specialist and obstetric input. |
| 94 / Q094 | Can semaglutide or other GLP-1 receptor agonists worsen diabetic retinopathy? | **Excluded** |  | Excluded: this item involves a specific medication class, such as semaglutide or GLP-1 receptor agonists, and specialist drug-safety judgment beyond the core scope of general public diabetic retinopathy consultation. |
| 95 / Q095 | Should I stop Ozempic, Wegovy, or Mounjaro because of diabetic retinopathy concerns? | **Excluded** |  | Excluded: direct medication stop/start advice involving brand-name drugs and individualized risk-benefit judgment; not appropriate for the general public diabetic retinopathy question set. |
| 96 / Q096 | Can cataract surgery worsen diabetic retinopathy or macular edema? | **Excluded** |  | Excluded: specific surgical scenario and less central to public DR counseling. |
| 97 / Q097 | Can diabetic retinopathy affect driving? | **Excluded** |  | Excluded: important but highly jurisdiction-dependent; not retained for an internationally applicable set. |
| 98 / Q098 | Can I fly or travel if I have diabetic retinopathy or bleeding in the eye? | **Excluded** |  | Excluded: specific lifestyle scenario with variable advice depending on diagnosis and treatment status. |
| 99 / Q099 | Does high blood pressure medicine affect diabetic retinopathy? | **Excluded** |  | Excluded: medication-specific question outside the direct retinopathy counseling scope. |
| 100 / Q100 | Are older adults with diabetes managed differently for eye screening? | **Excluded** |  | Excluded: age-specific management varies with health status and local practice; lower priority for final set. |
| 101 / Q101 | What questions should I ask my eye doctor after being diagnosed with diabetic retinopathy? | **Retained** | 47 | Retained: practical doctor-patient communication question reflecting real public needs after diagnosis. |
| 102 / Q102 | How can I understand whether online information about diabetic retinopathy is trustworthy? | **Excluded** |  | Excluded: broad online health-information appraisal question; lower priority than diabetic-retinopathy-specific screening, symptom, treatment, and care-seeking topics. |
| 103 / Q103 | How can I explain diabetic retinopathy to my family? | **Excluded** |  | Excluded: communication-support question with lower priority than directly actionable diabetic-retinopathy care topics. |
| 104 / Q104 | What should I do if I cannot afford eye exams or injections? | **Excluded** |  | Excluded: important access issue but heavily dependent on country, insurance, and local assistance programs. |
| 105 / Q105 | I am scared of losing my vision from diabetic retinopathy. What should I do? | **Retained** | 48 | Retained: common fear of vision loss requiring supportive, non-misleading, and action-oriented communication. |
| 106 / Q106 | How can I remember appointments and improve treatment adherence? | **Excluded** |  | Excluded: adherence-support question is useful but less central than retained clinical and safety items. |
| 107 / Q107 | Can an AI chatbot replace advice from my eye doctor? | **Retained** | 49 | Retained: directly addresses the appropriate role and limitations of AI-generated eye-health information. |
| 108 / Q108 | What information about my diabetes and eyesight should I bring to an eye appointment? | **Retained** | 50 | Retained: practical appointment-preparation question supporting organized communication with eye-care professionals. |

# Table S2-3. Final 50-question set used for model querying, in exact prompt order

| **Final No.** | **Candidate ID** | **Dimension** | **Final query wording** |
| --- | --- | --- | --- |
| 1 | Q001 | Basic understanding and public awareness | What is diabetic retinopathy? |
| 2 | Q002 | Basic understanding and public awareness | Can diabetic retinopathy make me blind? |
| 3 | Q004 | Basic understanding and public awareness | What is diabetic macular edema, and how is it related to diabetic retinopathy? |
| 4 | Q008 | Basic understanding and public awareness | Can diabetic retinopathy be reversed or cured? |
| 5 | Q010 | Basic understanding and public awareness | Can children or teenagers with diabetes develop diabetic retinopathy? |
| 6 | Q012 | Basic understanding and public awareness | Can I have diabetic retinopathy even if my vision seems normal? |
| 7 | Q013 | Risk factors and prevention | What increases my risk of diabetic retinopathy? |
| 8 | Q015 | Risk factors and prevention | Does high blood pressure make diabetic retinopathy worse? |
| 9 | Q016 | Risk factors and prevention | Does high cholesterol affect diabetic retinopathy? |
| 10 | Q017 | Risk factors and prevention | Does smoking increase the risk of diabetic retinopathy? |
| 11 | Q018 | Risk factors and prevention | Can good blood sugar control prevent diabetic retinopathy? |
| 12 | Q023 | Risk factors and prevention | Are there vitamins, supplements, or eye drops that prevent diabetic retinopathy? |
| 13 | Q026 | Risk factors and prevention | Can traditional or herbal medicines cure diabetic retinopathy? |
| 14 | Q027 | Screening and follow-up | If I have type 1 diabetes, when should I have my first dilated eye exam? |
| 15 | Q028 | Screening and follow-up | If I have type 2 diabetes, when should I have my first dilated eye exam? |
| 16 | Q029 | Screening and follow-up | How often should people with diabetes have a dilated eye exam? |
| 17 | Q030 | Screening and follow-up | If my last eye exam was normal, can I wait longer than one year? |
| 18 | Q031 | Screening and follow-up | Is a retinal screening photo the same as a full dilated eye exam? |
| 19 | Q032 | Screening and follow-up | Can retinal photos or AI help screen for diabetic retinopathy? |
| 20 | Q034 | Screening and follow-up | Do I still need an eye exam if I have no vision symptoms? |
| 21 | Q036 | Screening and follow-up | What should I do if my eye screening result says I need a referral? |
| 22 | Q040 | Screening and follow-up | If I already have mild diabetic retinopathy, how often should I have follow-up eye checks? |
| 23 | Q041 | Screening and follow-up | If I have moderate or severe diabetic retinopathy, how often should I have follow-up eye checks? |
| 24 | Q045 | Symptoms and urgent warning signs | What symptoms can diabetic retinopathy cause? |
| 25 | Q046 | Symptoms and urgent warning signs | Can diabetic retinopathy have no symptoms in the early stage? |
| 26 | Q047 | Symptoms and urgent warning signs | What should I do if I suddenly see many floaters or dark spots? |
| 27 | Q048 | Symptoms and urgent warning signs | What should I do if my vision suddenly becomes blurred or worse? |
| 28 | Q050 | Symptoms and urgent warning signs | Is eye pain a typical symptom of diabetic retinopathy? |
| 29 | Q054 | Symptoms and urgent warning signs | When should someone with diabetes seek urgent eye care? |
| 30 | Q055 | Symptoms and urgent warning signs | Can symptoms come and go even if the disease is serious? |
| 31 | Q058 | Diagnosis, staging, and test interpretation | How is diabetic retinopathy diagnosed? |
| 32 | Q060 | Diagnosis, staging, and test interpretation | My report says 'proliferative diabetic retinopathy.' What does that mean? |
| 33 | Q068 | Diagnosis, staging, and test interpretation | Does a normal vision chart test mean my retina is healthy? |
| 34 | Q069 | Diagnosis, staging, and test interpretation | How should I understand my diabetic eye screening report? |
| 35 | Q071 | Treatment options and procedure safety | When does diabetic retinopathy need treatment? |
| 36 | Q072 | Treatment options and procedure safety | What treatments are available for diabetic retinopathy? |
| 37 | Q073 | Treatment options and procedure safety | What are eye injections for diabetic retinopathy, and when might I need them? |
| 38 | Q074 | Treatment options and procedure safety | Are eye injections painful or dangerous? |
| 39 | Q076 | Treatment options and procedure safety | What is laser treatment for diabetic retinopathy? |
| 40 | Q078 | Treatment options and procedure safety | What are possible side effects of laser treatment? |
| 41 | Q079 | Treatment options and procedure safety | When might diabetic retinopathy need eye surgery? |
| 42 | Q083 | Treatment options and procedure safety | Can treatment improve vision, or does it only stop worsening? |
| 43 | Q084 | Treatment options and procedure safety | What happens if diabetic retinopathy is not treated? |
| 44 | Q086 | Treatment options and procedure safety | Are there home remedies that can replace medical treatment? |
| 45 | Q089 | Special situations, comorbidities, and life contexts | I have diabetes and am planning pregnancy. When should I have an eye exam? |
| 46 | Q091 | Special situations, comorbidities, and life contexts | If I have gestational diabetes during pregnancy, do I need diabetic retinopathy eye exams? |
| 47 | Q101 | Self-management, access, reliability, and communication | What questions should I ask my eye doctor after being diagnosed with diabetic retinopathy? |
| 48 | Q105 | Self-management, access, reliability, and communication | I am scared of losing my vision from diabetic retinopathy. What should I do? |
| 49 | Q107 | Self-management, access, reliability, and communication | Can an AI chatbot replace advice from my eye doctor? |
| 50 | Q108 | Self-management, access, reliability, and communication | What information about my diabetes and eyesight should I bring to an eye appointment? |

*Note: The wording and sequence shown in this table reproduce the prompts submitted to all six model interfaces. The complete archived responses are provided in Supplementary Material 4.*

# Table S2-4. Differences between candidate-pool wording and final query wording

| **Final No.** | **Candidate ID** | **Candidate-pool wording** | **Final query wording** | **Reason for pre-query refinement** |
| --- | --- | --- | --- | --- |
| 3 | Q004 | What is macular edema, and how is it related to diabetic retinopathy? | What is diabetic macular edema, and how is it related to diabetic retinopathy? | Adds the word 'diabetic' to match the disease context and reduce ambiguity. |
| 14 | Q027 | When should a person with type 1 diabetes have their first dilated eye exam? | If I have type 1 diabetes, when should I have my first dilated eye exam? | Changes third-person wording to a direct patient question. |
| 15 | Q028 | When should a person with type 2 diabetes have their first dilated eye exam? | If I have type 2 diabetes, when should I have my first dilated eye exam? | Changes third-person wording to a direct patient question. |
| 18 | Q031 | Can a retinal screening photo replace a full dilated eye exam? | Is a retinal screening photo the same as a full dilated eye exam? | Uses neutral wording that better reflects a public question about whether the two tests are equivalent. |
| 19 | Q032 | Can retinal photos or artificial intelligence help screen for diabetic retinopathy? | Can retinal photos or AI help screen for diabetic retinopathy? | Uses the commonly understood abbreviation 'AI' and shortens the question. |
| 21 | Q036 | What should I do if my screening result says I need referral? | What should I do if my eye screening result says I need a referral? | Clarifies that the referral comes from an eye-screening result. |
| 34 | Q069 | How should I understand my retinal screening report? | How should I understand my diabetic eye screening report? | Uses the patient-facing phrase 'diabetic eye screening report' instead of the more technical 'retinal screening report'. |
| 46 | Q091 | Do people with gestational diabetes need diabetic retinopathy eye exams during pregnancy? | If I have gestational diabetes during pregnancy, do I need diabetic retinopathy eye exams? | Changes third-person wording to a direct patient question. |
| 50 | Q108 | What information about my diabetes and vision should I bring to an eye appointment? | What information about my diabetes and eyesight should I bring to an eye appointment? | Uses 'eyesight' for a more patient-facing expression while preserving the meaning. |

*Note: All refinements shown in this table were completed before the final prompt set was locked and before model querying. The “Final query wording” column reproduces the wording actually submitted to the model interfaces.*
