# Supplementary material for: Large language models as sources of diabetic retinopathy patient education: a comparative evaluation of safety, health information quality, and readability
Source: Front Public Health. 2026 Oct 9;14:1945318. doi: 10.3389/fpubh.2026.1945318 (PMC13690771; doi:10.3389/fpubh.2026.1945318)
Supplement: Supplementary file 3 [file Supplementary_file_3.docx]

**Supplementary Material 3. Question–Answer-to-Guideline Mapping Table**

Diabetic Retinopathy LLM Study | Reference-standard answer elements for public consultation questions

Purpose: This document maps each of the 50 final public-facing diabetic retinopathy questions to concise guideline-aligned answer elements and the corresponding guideline or consensus basis. It is intended for evaluation of LLM responses, not for direct clinical decision-making.

Evidence coverage note: The evidence base was finalized using 21 verified guideline, consensus, teleophthalmology, AI-screening, and landmark clinical-trial references.

**Source Key**

| **Code** | **Verified guideline / consensus / supporting evidence source** |
| --- | --- |
| **G1** | American Diabetes Association Professional Practice Committee for Diabetes. 12. Retinopathy, Neuropathy, and Foot Care: Standards of Care in Diabetes-2026. Diabetes Care. 2026;49(Suppl 1):S261-S276. |
| **G2** | Lim JI, Kim SJ, Bailey ST, Kovach JL, Vemulakonda GA, Ying GS, Flaxel CJ; American Academy of Ophthalmology Preferred Practice Pattern Retina/Vitreous Committee. Diabetic Retinopathy Preferred Practice Pattern®. Ophthalmology. 2025;132(4):P75-P162. |
| **G3** | National Institute for Health and Care Excellence. Diabetic retinopathy: management and monitoring. NICE Guideline NG242. Published 13 August 2024. |
| **G4** | Blonde L, Umpierrez GE, Reddy SS, McGill JB, Berga SL, Bush M, et al. American Association of Clinical Endocrinology Clinical Practice Guideline: Developing a Diabetes Mellitus Comprehensive Care Plan-2022 Update. Endocr Pract. 2022;28(10):923-1049. |
| **G5** | Fundus Disease Group of Ophthalmological Society of Chinese Medical Association; Fundus Disease Group of Ophthalmologist Branch of Chinese Medical Doctor Association. Evidence-based guidelines for diagnosis and treatment of diabetic retinopathy in China (2022). Chin J Ocul Fundus Dis. 2023;39(2):99-124. |
| **G6** | Ng DSC, Ruamviboonsuk P, Apte RS, Bajimaya S, Chan CKM, Chang A, et al. International consensuses and controversies on causes, diagnosis and management of diabetic macular edema (DME). Prog Retin Eye Res. 2025;109:101406. |
| **G7** | Lam RF, Radke NV, et al. International consensuses and guidelines on management of proliferative diabetic retinopathy (PDR) by the Asia-Pacific Vitreo-retina Society (APVRS), the Academy of Asia-Pacific Professors of Ophthalmology (AAPPO) and the Academia Retina Internationalis (ARI). Asia Pac J Ophthalmol (Phila). 2025;14(6):100262. |
| **G8** | Yonekawa Y, Modi YS, Kim LA, Skondra D, Kim JE, Wykoff CC. American Society of Retina Specialists Clinical Practice Guidelines: Management of Nonproliferative and Proliferative Diabetic Retinopathy Without Diabetic Macular Edema. J Vitreoretin Dis. 2020;4(2):125-135. |
| **G9** | Horton MB, Brady CJ, Cavallerano J, Abramoff MD, Barker GP, Chiang MF, et al. Practice Guidelines for Ocular Telehealth-Diabetic Retinopathy, Third Edition. Telemed J E Health. 2020;26(4):495-543. |
| **G10** | Wong TY, Sun J, Kawasaki R, Ruamviboonsuk P, Gupta N, Lansingh VC, et al. Guidelines on Diabetic Eye Care: The International Council of Ophthalmology Recommendations for Screening, Follow-up, Referral, and Treatment Based on Resource Settings. Ophthalmology. 2018;125(10):1608-1622. |
| **G11** | Early Treatment Diabetic Retinopathy Study Research Group. Photocoagulation for diabetic macular edema: Early Treatment Diabetic Retinopathy Study Report Number 1. Arch Ophthalmol. 1985;103(12):1796-1806. |
| **G12** | Diabetic Retinopathy Study Research Group. Photocoagulation treatment of proliferative diabetic retinopathy: clinical application of Diabetic Retinopathy Study findings, DRS Report Number 8. Ophthalmology. 1981;88(7):583-600. |
| **G13** | Wells JA, Glassman AR, Ayala AR, Jampol LM, Aiello LP, Antoszyk AN, et al.; Diabetic Retinopathy Clinical Research Network. Aflibercept, bevacizumab, or ranibizumab for diabetic macular edema. N Engl J Med. 2015;372(13):1193-1203. |
| **G14** | Wells JA, Glassman AR, Ayala AR, Jampol LM, Bressler NM, Bressler SB, et al.; Diabetic Retinopathy Clinical Research Network. Aflibercept, bevacizumab, or ranibizumab for diabetic macular edema: two-year results from a comparative effectiveness randomized clinical trial. Ophthalmology. 2016;123(6):1351-1359. |
| **G15** | Gross JG, Glassman AR, Jampol LM, Inusah S, Aiello LP, Antoszyk AN, et al.; Writing Committee for the Diabetic Retinopathy Clinical Research Network. Panretinal photocoagulation vs intravitreous ranibizumab for proliferative diabetic retinopathy: a randomized clinical trial. JAMA. 2015;314(20):2137-2146. |
| **G16** | Gross JG, Glassman AR, Liu D, Sun JK, Antoszyk AN, Baker CW, et al.; Diabetic Retinopathy Clinical Research Network. Five-year outcomes of panretinal photocoagulation vs intravitreous ranibizumab for proliferative diabetic retinopathy: a randomized clinical trial. JAMA Ophthalmol. 2018;136(10):1138-1148. |
| **G17** | Brown DM, Wykoff CC, Boyer D, Heier JS, Clark WL, Emanuelli A, et al. Evaluation of intravitreal aflibercept for the treatment of severe nonproliferative diabetic retinopathy: results from the PANORAMA randomized clinical trial. JAMA Ophthalmol. 2021;139(9):946-955. |
| **G18** | Nguyen QD, Brown DM, Marcus DM, Boyer DS, Patel S, Feiner L, et al.; RISE and RIDE Research Group. Ranibizumab for diabetic macular edema: results from 2 phase III randomized trials: RISE and RIDE. Ophthalmology. 2012;119(4):789-801. |
| **G19** | Korobelnik JF, Do DV, Schmidt-Erfurth U, Boyer DS, Holz FG, Heier JS, et al. Intravitreal aflibercept for diabetic macular edema. Ophthalmology. 2014;121(11):2247-2254. |
| **G20** | Abramoff MD, Lavin PT, Birch M, Shah N, Folk JC. Pivotal trial of an autonomous AI-based diagnostic system for detection of diabetic retinopathy in primary care offices. NPJ Digit Med. 2018;1:39. |
| **G21** | Rajesh AE, Davidson OQ, Lee CS, Lee AY. Artificial Intelligence and Diabetic Retinopathy: AI Framework, Prospective Studies, Head-to-head Validation, and Cost-effectiveness. Diabetes Care. 2023;46(10):1728-1739. |

**Basic understanding and public awareness**

| **No.** | **Dimension** | **Question** | **Reference-standard answer elements** | **Mapped guideline / consensus content** | **Sources** |
| --- | --- | --- | --- | --- | --- |
| 1 | Basic understanding and public awareness | **What is diabetic retinopathy?** | Define DR as a diabetes-related neurovascular/microvascular retinal complication. Mention early microaneurysms, hemorrhage/ischemia, possible neovascularization in PDR, possible DME at any stage, and the risk of preventable vision loss. | The guideline definition describes DR as a highly diabetes-specific neurovascular complication, with microaneurysms as early changes, hemorrhage/ischemia, PDR when neovascularization occurs, and DME from retinal vascular leakage. | **G1; G2; G5** |
| 2 | Basic understanding and public awareness | **Can diabetic retinopathy make me blind?** | Answer yes, but avoid fatalism. Explain that untreated DR, DME, PDR, vitreous hemorrhage, tractional retinal detachment, and neovascular glaucoma can cause severe vision loss or blindness; screening and treatment greatly reduce risk. | Standards and retina guidelines identify DR as a leading cause of blindness and emphasize prompt diagnosis, referral, laser/anti-VEGF treatment, and monitoring to prevent vision loss. | **G1; G2; G3; G7** |
| 3 | Basic understanding and public awareness | **What is diabetic macular edema, and how is it related to diabetic retinopathy?** | Explain that DME is fluid/exudate accumulation and retinal thickening in the macula, causing central vision impairment. State that DME is a complication of diabetic retinal vessel leakage and may occur at different DR stages. | Guidelines and consensus documents define DME as macular thickening/fluid due to capillary leakage/blood-retinal barrier breakdown and recognize it as a major form of vision-threatening DR. | **G1; G3; G5; G6** |
| 4 | Basic understanding and public awareness | **Can diabetic retinopathy be reversed or cured?** | State that DR is usually not permanently cured; early disease may improve or stabilize with glucose/BP/lipid control, while advanced structural damage is often irreversible. Treatments aim to preserve or improve vision and prevent progression. | Guidelines emphasize lifelong systemic risk-factor control, early detection, and timely treatment; current therapies can prevent and sometimes improve vision but do not erase all permanent retinal damage. | **G1; G2; G3; G6** |
| 5 | Basic understanding and public awareness | **Can children or teenagers with diabetes develop diabetic retinopathy?** | Answer yes. Explain risk is related to diabetes duration, glycemic control, puberty, and type of diabetes. Type 1 youth are generally screened after several years of disease; type 2 youth should be screened at or soon after diagnosis. | ADA and diabetes-care guidance state that children and adolescents with type 1 or type 2 diabetes are at risk for DR and need screening according to diabetes type and duration. | **G1; G4** |
| 6 | Basic understanding and public awareness | **Can I have diabetic retinopathy even if my vision seems normal?** | Answer yes. Early DR and even vision-threatening disease may be asymptomatic; normal vision does not rule out retinal changes. Emphasize regular dilated examination or validated screening. | Guidelines explicitly note that people with vision-threatening retinopathy may be asymptomatic, making prompt screening and diagnosis essential. | **G1; G2; G9** |

**Risk factors and prevention**

| **No.** | **Dimension** | **Question** | **Reference-standard answer elements** | **Mapped guideline / consensus content** | **Sources** |
| --- | --- | --- | --- | --- | --- |
| 7 | Risk factors and prevention | **What increases my risk of diabetic retinopathy?** | List major risk factors: longer diabetes duration, chronic hyperglycemia, hypertension, dyslipidemia, nephropathy, pregnancy/preexisting diabetes, and poor access/adherence to screening. Avoid implying that age alone is the main cause. | ADA, AAO, AACE, and China guidelines identify diabetes duration and chronic hyperglycemia as central risks and also cite hypertension, dyslipidemia, nephropathy, and pregnancy-related progression. | **G1; G2; G4; G5** |
| 8 | Risk factors and prevention | **Does high blood pressure make diabetic retinopathy worse?** | Answer yes. High blood pressure can worsen retinal microvascular injury and progression. Encourage BP management through the diabetes/primary-care team and eye follow-up; do not suggest self-adjusting medications. | ADA recommends reaching BP goals to reduce risk/progression. NICE states that BP management in people with hypertension can reduce NPDR progression. | **G1; G3; G4** |
| 9 | Risk factors and prevention | **Does high cholesterol affect diabetic retinopathy?** | Answer yes, as part of a broader risk profile. Explain that dyslipidemia is associated with DR/DME and exudates; lipid goals and appropriate lipid-lowering therapy should be managed by clinicians. | ADA recommends lipid-goal strategies for DR risk/progression, and NICE discusses fenofibrate/statin evidence for progression risk in selected contexts. | **G1; G3; G4** |
| 10 | Risk factors and prevention | **Does smoking increase the risk of diabetic retinopathy?** | State that smoking is harmful for vascular and diabetes outcomes and may worsen eye-health risk, while evidence for DR-specific risk may vary. Recommend cessation support and regular eye screening. | AAO and diabetes-care guidance include smoking in risk assessment and overall diabetes complication prevention; cessation is consistent with comprehensive diabetes care. | **G2; G4; G5** |
| 11 | Risk factors and prevention | **Can good blood sugar control prevent diabetic retinopathy?** | Answer yes, it substantially reduces risk and slows progression, but it is not a complete guarantee. Note that very rapid A1c reduction in high-risk patients may require eye assessment/monitoring. | ADA recommends glycemic-goal strategies to reduce risk/progression; NICE advises notifying ophthalmology when a treatment may cause a rapid, substantial HbA1c drop. | **G1; G3; G4** |
| 12 | Risk factors and prevention | **Are there vitamins, supplements, or eye drops that prevent diabetic retinopathy?** | State that no vitamin, supplement, or eye drop is proven to prevent DR on its own or replace standard care. Supplements may correct deficiencies but should not substitute glucose/BP/lipid control and screening. | AACE states that nutritional supplements have modest or neutral effects and should not be used for diabetes/CV-risk management until scientifically proven; DR guidelines prioritize systemic control and screening. | **G1; G4; G2** |
| 13 | Risk factors and prevention | **Can traditional or herbal medicines cure diabetic retinopathy?** | State clearly that traditional or herbal medicines have not been proven to cure DR and should not delay or replace evidence-based care. Mention possible interactions and the need to discuss use with clinicians. | Guidelines support evidence-based systemic management, screening, laser, anti-VEGF, corticosteroid in selected DME, and vitrectomy when indicated; alternative cures are not standard DR treatments. | **G1; G3; G4; G5; G6** |

**Screening and follow-up**

| **No.** | **Dimension** | **Question** | **Reference-standard answer elements** | **Mapped guideline / consensus content** | **Sources** |
| --- | --- | --- | --- | --- | --- |
| 14 | Screening and follow-up | **If I have type 1 diabetes, when should I have my first dilated eye exam?** | For type 1 diabetes, initial dilated comprehensive eye examination is generally 5 years after onset/diagnosis in adults; pediatric timing depends on age/duration. Earlier assessment if symptoms or pregnancy planning. | ADA screening recommendation: adults with type 1 diabetes should have initial dilated comprehensive eye examination 5 years after onset. | **G1; G2; G4** |
| 15 | Screening and follow-up | **If I have type 2 diabetes, when should I have my first dilated eye exam?** | For type 2 diabetes, initial dilated comprehensive eye examination should occur at the time of diagnosis because diabetes may have been present for years before detection. | ADA screening recommendation: people with type 2 diabetes should have initial dilated comprehensive eye examination at diagnosis. | **G1; G2; G4** |
| 16 | Screening and follow-up | **How often should people with diabetes have a dilated eye exam?** | Generally recommend annual dilated retinal examination for people with diabetes; intervals may lengthen to 1-2 years after normal exams and good control, or shorten if DR/DME is present or progressing. | ADA recommends at least annual exams when DR is present; every 1-2 years may be considered after normal exams and goal-range glycemic indicators. | **G1; G2** |
| 17 | Screening and follow-up | **If my last eye exam was normal, can I wait longer than one year?** | Answer conditionally yes. If one or more exams show no DR and glycemic indicators are at goal, 1-2 year screening may be considered; do not advise skipping follow-up without clinician guidance. | ADA allows 1-2 year screening after normal examinations under good control, but recommends individualized shorter intervals when risk factors or any DR/DME exist. | **G1; G2** |
| 18 | Screening and follow-up | **Is a retinal screening photo the same as a full dilated eye exam?** | State that a retinal screening photo is useful but not the same as a full dilated comprehensive eye exam. Abnormal, ungradable, or high-risk results require referral pathways and in-person evaluation. | ADA states retinal photography/AI can be appropriate screening, but photographs are not a substitute for dilated comprehensive eye exams when indicated; telehealth guidelines require validation and referral systems. | **G1; G8; G9; G10** |
| 19 | Screening and follow-up | **Can retinal photos or AI help screen for diabetic retinopathy?** | Answer yes. Retinal photos and validated AI can improve DR screening access and triage, but results must be reliable, documented, and linked to timely referral for comprehensive eye examination when abnormal or ungradable. | ADA endorses retinal photography with remote reading or FDA-approved AI screening strategies if referral pathways exist; ocular telehealth guidelines address validation, image quality, personnel, documentation, and communication. | **G1; G9; G10; G20; G21** |
| 20 | Screening and follow-up | **Do I still need an eye exam if I have no vision symptoms?** | Answer yes. No symptoms does not rule out DR; routine screening is needed because early and even vision-threatening disease may be silent. | ADA explains that identifying diabetes-related eye disease is important because vision-threatening retinopathy may be asymptomatic and timely intervention can prevent vision loss. | **G1; G2** |
| 21 | Screening and follow-up | **What should I do if my eye screening result says I need a referral?** | Advise arranging the recommended comprehensive eye examination or ophthalmology referral within the stated timeframe. Urgent symptoms require same-day care. The answer should not minimize referral findings. | ADA recommends prompt referral for DME, moderate or worse NPDR, or any PDR; telehealth and screening guidelines require timely referral for abnormal or inconclusive screening results. | **G1; G2; G9; G10** |
| 22 | Screening and follow-up | **If I already have mild diabetic retinopathy, how often should I have follow-up eye checks?** | For mild DR, follow-up is usually at least annually and may be 6-12 months depending on risk, metabolic control, and clinician assessment. Avoid fixed universal intervals without context. | ADA states subsequent examinations are generally repeated annually for no or mild retinopathy, with more frequent exams if risk factors, progression, or DME are present. | **G1; G2; G3** |
| 23 | Screening and follow-up | **If I have moderate or severe diabetic retinopathy, how often should I have follow-up eye checks?** | For moderate NPDR, monitoring may be every 6-12 months; for severe/very severe NPDR, every 3-6 months is commonly considered, with retina/ophthalmology oversight and faster review if progression or symptoms occur. | NICE recommends hospital eye services consider 6-12 months for moderate NPDR and 3-6 months for severe or very severe NPDR; ADA requires more frequent ophthalmologist exams for progressing or sight-threatening retinopathy. | **G3; G1; G2** |

**Symptoms and urgent warning signs**

| **No.** | **Dimension** | **Question** | **Reference-standard answer elements** | **Mapped guideline / consensus content** | **Sources** |
| --- | --- | --- | --- | --- | --- |
| 24 | Symptoms and urgent warning signs | **What symptoms can diabetic retinopathy cause?** | List symptoms: blurred/fluctuating vision, floaters, dark spots, poor night vision, color/contrast change, distorted vision, and sudden vision loss. State that symptoms often appear late. | DR guideline descriptions include asymptomatic early disease and later complications such as vitreous hemorrhage, DME, retinal detachment, and vision loss. | **G1; G2; G5** |
| 25 | Symptoms and urgent warning signs | **Can diabetic retinopathy have no symptoms in the early stage?** | Answer yes. Early NPDR may have no symptoms, which is why screening is recommended even when vision feels normal. | Guidelines emphasize that vision-threatening retinopathy can be asymptomatic and that screening enables timely intervention before vision loss. | **G1; G2** |
| 26 | Symptoms and urgent warning signs | **What should I do if I suddenly see many floaters or dark spots?** | Recommend urgent/same-day eye evaluation for sudden many floaters or dark spots, especially with flashes, curtain/shadow, or vision loss. Explain concern for vitreous hemorrhage or retinal detachment/tear. | PDR can cause vitreous hemorrhage and tractional retinal detachment; guidelines stress prompt referral and treatment for sight-threatening disease. | **G1; G2; G7** |
| 27 | Symptoms and urgent warning signs | **What should I do if my vision suddenly becomes blurred or worse?** | Recommend prompt/urgent evaluation for sudden blurred or worsening vision, especially if sudden, severe, unilateral, with floaters/flashes/curtain, eye pain, neurologic symptoms, or vision loss. | Guidelines describe DME, vitreous hemorrhage, retinal detachment, and PDR as vision-threatening complications requiring timely ophthalmic assessment and treatment. | **G1; G2; G3** |
| 28 | Symptoms and urgent warning signs | **Is eye pain a typical symptom of diabetic retinopathy?** | State that eye pain is not a typical early DR symptom. Pain may suggest other serious ocular problems, including neovascular glaucoma, acute glaucoma, inflammation, or infection, and warrants urgent evaluation when severe or sudden. | DR is often asymptomatic or presents with visual symptoms; advanced neovascular complications can involve glaucoma and require eye-specialist care. | **G1; G2; G7** |
| 29 | Symptoms and urgent warning signs | **When should someone with diabetes seek urgent eye care?** | Urgent eye care is warranted for sudden vision loss, new shower of floaters, flashes, curtain/shadow, large dark spot, severe persistent blur, or eye pain/redness/headache/nausea. Advise not to wait. | Guidelines identify PDR, DME, vitreous hemorrhage, and retinal detachment as sight-threatening; prompt referral/treatment prevents irreversible vision loss. | **G1; G2; G7** |
| 30 | Symptoms and urgent warning signs | **Can symptoms come and go even if the disease is serious?** | Answer yes. Intermittent or fluctuating visual symptoms can still occur with serious disease; coming and going should not be used to rule out DR/DME. Encourage examination if symptoms are new, recurrent, or worsening. | DR and DME can be asymptomatic, fluctuating, or progressive; guideline-based safety messaging prioritizes screening and timely evaluation over symptom reassurance. | **G1; G2; G6** |

**Diagnosis, staging, and test interpretation**

| **No.** | **Dimension** | **Question** | **Reference-standard answer elements** | **Mapped guideline / consensus content** | **Sources** |
| --- | --- | --- | --- | --- | --- |
| 31 | Diagnosis, staging, and test interpretation | **How is diabetic retinopathy diagnosed?** | Diagnosis should include history and dilated retinal examination; retinal photography can screen; OCT assesses macular edema; fluorescein angiography/OCT angiography may help evaluate leakage/ischemia when needed. | AAO and China guidelines describe clinical examination plus ancillary imaging; DME consensus emphasizes OCT for objective DME assessment while noting structure-function limitations. | **G2; G5; G6; G9** |
| 32 | Diagnosis, staging, and test interpretation | **My report says 'proliferative diabetic retinopathy.' What does that mean?** | Explain PDR as advanced DR with retinal neovascularization due to ischemia/VEGF. It can bleed or cause tractional retinal detachment/neovascular glaucoma, so it requires retina-specialist management. | ADA and PDR consensus define PDR by neovascularization and emphasize complications such as vitreous hemorrhage, tractional retinal detachment, and irreversible vision loss without prompt intervention. | **G1; G7; G8** |
| 33 | Diagnosis, staging, and test interpretation | **Does a normal vision chart test mean my retina is healthy?** | Answer no. A normal vision chart only measures central visual acuity and does not inspect the retina; early DR/DME or peripheral disease can exist despite good acuity. | Guidelines emphasize that retinal disease may be asymptomatic and requires validated screening/dilated examination or retinal imaging rather than visual acuity alone. | **G1; G2; G9** |
| 34 | Diagnosis, staging, and test interpretation | **How should I understand my diabetic eye screening report?** | Explain report components: image quality/gradability, DR severity, DME/maculopathy status, whether referral is needed, follow-up interval, and urgent symptoms. Encourage asking the eye professional to explain the result. | Telehealth guidelines emphasize communication, documentation, image quality, and referral pathways. ADA recommends referral for DME, moderate or worse NPDR, or any PDR. | **G1; G9; G2** |

**Treatment options and procedure safety**

| **No.** | **Dimension** | **Question** | **Reference-standard answer elements** | **Mapped guideline / consensus content** | **Sources** |
| --- | --- | --- | --- | --- | --- |
| 35 | Treatment options and procedure safety | **When does diabetic retinopathy need treatment?** | Treatment is needed for vision-threatening disease such as DME affecting vision, PDR/high-risk PDR, and sometimes severe NPDR; mild disease often needs systemic control and monitoring rather than immediate ocular treatment. | ADA recommends prompt referral for DME, moderate or worse NPDR, or any PDR; PRP is indicated for high-risk PDR and anti-VEGF is first-line for most center-involved DME with impaired vision. | **G1; G2; G3; G7; G8; G15; G16** |
| 36 | Treatment options and procedure safety | **What treatments are available for diabetic retinopathy?** | Available treatments include systemic glucose/BP/lipid optimization, anti-VEGF injections, laser photocoagulation, intravitreal corticosteroids in selected DME, and vitrectomy for advanced complications. | ADA, NICE, DME consensus, and PDR consensus summarize anti-VEGF, PRP/focal-grid laser, steroids in selected cases, and vitrectomy for advanced DR/DME/PDR. | **G1; G3; G6; G7; G11; G12; G13; G18; G19** |
| 37 | Treatment options and procedure safety | **What are eye injections for diabetic retinopathy, and when might I need them?** | Explain intravitreal injections: anti-VEGF drugs reduce leakage, DME, and abnormal neovascularization; they are first-line for most center-involved DME with impaired acuity and an alternative to PRP for some PDR. Steroids are selected cases. | ADA recommends anti-VEGF as first-line for most center-involved DME impairing visual acuity and as a reasonable alternative to PRP for some PDR; DME consensus discusses individualized anti-VEGF/steroid strategies. | **G1; G3; G6; G7; G13; G14; G18; G19** |
| 38 | Treatment options and procedure safety | **Are eye injections painful or dangerous?** | State that injections are usually done with antisepsis and anesthesia and are generally safe, but rare serious risks include infection/endophthalmitis, retinal tear/detachment, bleeding, pressure rise, and inflammation; urgent pain/vision loss after injection needs care. | Guidelines discuss intravitreal anti-VEGF and steroid treatments as standard options while requiring informed decision-making and reporting/monitoring of adverse events. | **G1; G2; G3; G6; G13; G14** |
| 39 | Treatment options and procedure safety | **What is laser treatment for diabetic retinopathy?** | Explain PRP and focal/grid laser. PRP treats PDR by reducing drive for abnormal new vessels; focal/grid laser treats selected DME/leakage. It usually prevents worsening more than restores already lost vision. | ADA states PRP reduces vision loss in high-risk PDR and in some severe NPDR; focal/grid laser remains a reasonable treatment for persistent DME or when anti-VEGF is unsuitable. | **G1; G3; G7; G8; G11; G12; G15; G16** |
| 40 | Treatment options and procedure safety | **What are possible side effects of laser treatment?** | Mention possible side effects: temporary discomfort/light sensitivity/blur, inflammation, reduced night or peripheral vision after PRP, macular edema worsening, scarring, and rare vision loss. Risk-benefit discussion is required. | Laser guidelines support PRP/focal-grid laser for selected indications but recognize treatment effects and individualized decision-making, especially where central vision or field loss matters. | **G1; G2; G3; G7; G11; G12** |
| 41 | Treatment options and procedure safety | **When might diabetic retinopathy need eye surgery?** | Eye surgery, especially vitrectomy, may be needed for non-clearing vitreous hemorrhage, tractional retinal detachment threatening the macula, severe fibrovascular proliferation, or selected tractional/refractory DME cases. | NICE and PDR consensus include vitrectomy for advanced PDR complications; ADA lists tractional retinal detachment and vitreous hemorrhage as major complications of DR/PDR. | **G1; G2; G3; G7** |
| 42 | Treatment options and procedure safety | **Can treatment improve vision, or does it only stop worsening?** | Treatment can do both. Anti-VEGF for DME may improve vision, and PRP/laser can reduce severe vision-loss risk; advanced retinal cell loss or scarring may limit recovery. | ADA notes current therapies can prevent vision loss and help improve vision for many; DME consensus reports anti-VEGF superiority over laser for improving vision in DME trials. | **G1; G3; G6; G13; G14; G18; G19** |
| 43 | Treatment options and procedure safety | **What happens if diabetic retinopathy is not treated?** | Untreated DR may progress from NPDR to PDR and DME, leading to vitreous hemorrhage, tractional retinal detachment, neovascular glaucoma, irreversible vision loss, or blindness. | ADA and PDR consensus describe neovascularization, vitreous hemorrhage, tractional retinal detachment, and irreversible vision loss as complications of untreated or advanced PDR. | **G1; G2; G7; G12; G15; G16** |
| 44 | Treatment options and procedure safety | **Are there home remedies that can replace medical treatment?** | State no: home remedies cannot replace medical treatment for DR/DME/PDR. Lifestyle measures support diabetes control but do not substitute eye exams, laser, injections, surgery, or retina follow-up when indicated. | Guidelines identify evidence-based ocular treatments and systemic risk-factor control; AACE cautions against relying on unproven supplements for diabetes management. | **G1; G3; G4; G5** |

**Special situations, comorbidities, and life contexts**

| **No.** | **Dimension** | **Question** | **Reference-standard answer elements** | **Mapped guideline / consensus content** | **Sources** |
| --- | --- | --- | --- | --- | --- |
| 45 | Special situations, comorbidities, and life contexts | **I have diabetes and am planning pregnancy. When should I have an eye exam?** | For preexisting type 1 or type 2 diabetes, arrange a dilated eye exam before pregnancy or in the first trimester, and monitor every trimester and up to 1 year postpartum as indicated by retinopathy severity. | ADA recommends counseling individuals with preexisting diabetes about pregnancy-related DR risk and eye examination before pregnancy/first trimester with trimester and postpartum follow-up as indicated. | **G1; G4; G2** |
| 46 | Special situations, comorbidities, and life contexts | **If I have gestational diabetes during pregnancy, do I need diabetic retinopathy eye exams?** | If it is gestational diabetes only, routine DR eye exams solely for gestational diabetes are generally not required. Confirm that diabetes was not preexisting or undiagnosed type 2 diabetes; assess if symptoms or other risk factors exist. | ADA states individuals who develop gestational diabetes mellitus do not require eye examinations during pregnancy because they do not appear to be at increased risk of developing DR during pregnancy. | **G1** |

**Self-management, access, reliability, and communication**

| **No.** | **Dimension** | **Question** | **Reference-standard answer elements** | **Mapped guideline / consensus content** | **Sources** |
| --- | --- | --- | --- | --- | --- |
| 47 | Self-management, access, reliability, and communication | **What questions should I ask my eye doctor after being diagnosed with diabetic retinopathy?** | Questions should cover stage/severity, DME status, test results, treatment options/risks, follow-up interval, urgent symptoms, systemic targets, pregnancy considerations, and who to contact if vision changes occur. | Guidelines emphasize informed shared decision-making, communication between eye and diabetes-care teams, access to HbA1c/BP/renal information, and tailored follow-up/treatment. | **G1; G2; G3; G9** |
| 48 | Self-management, access, reliability, and communication | **I am scared of losing my vision from diabetic retinopathy. What should I do?** | Use empathic reassurance: fear is understandable, but blindness is often preventable with screening, glucose/BP/lipid control, timely treatment, and urgent care for sudden symptoms. Mention vision rehabilitation/support if vision loss has occurred. | ADA recommends systemic risk-factor optimization, timely screening/referral/treatment, and counseling/referral for vision rehabilitation and educational resources when diabetes-related vision loss occurs. | **G1; G2; G3** |
| 49 | Self-management, access, reliability, and communication | **Can an AI chatbot replace advice from my eye doctor?** | Answer no. AI chatbots can provide general education and may support triage, while validated AI retinal systems can screen images. They cannot perform a full exam, interpret all tests personally, or replace an eye doctor's individualized advice. | ADA and telehealth guidelines support validated AI/retinal-photo screening only with documentation and referral pathways; DME consensus calls for rigorous appraisal of AI reliability and generalizability before implementation. | **G1; G6; G9; G20; G21** |
| 50 | Self-management, access, reliability, and communication | **What information about my diabetes and eyesight should I bring to an eye appointment?** | Bring diabetes duration/type, recent HbA1c, blood pressure, lipid and kidney results if available, medications, pregnancy status/plans, symptoms and timing, prior eye reports/images/treatments, glasses/contacts, and questions. | NICE recommends ophthalmologists have access to HbA1c and BP and consider DR stage, HbA1c, renal function, and BP when deciding interventions and follow-up; guidelines emphasize team communication and documentation. | **G3; G1; G2; G9** |

**Updated Reference List**

Verification note: The final 50 questions were not modified. This section only finalizes the supporting evidence base. Bibliographic details were checked against PubMed, official guideline pages, or journal pages where available. References are listed in Vancouver style and may be renumbered according to target journal requirements.

1. American Diabetes Association Professional Practice Committee for Diabetes. 12. Retinopathy, Neuropathy, and Foot Care: Standards of Care in Diabetes-2026. Diabetes Care. 2026;49(Suppl 1):S261-S276. doi:10.2337/dc26-S012.

2. Lim JI, Kim SJ, Bailey ST, Kovach JL, Vemulakonda GA, Ying GS, Flaxel CJ; American Academy of Ophthalmology Preferred Practice Pattern Retina/Vitreous Committee. Diabetic Retinopathy Preferred Practice Pattern®. Ophthalmology. 2025;132(4):P75-P162. doi:10.1016/j.ophtha.2024.12.020.

3. National Institute for Health and Care Excellence. Diabetic retinopathy: management and monitoring. NICE Guideline NG242. Published 13 August 2024. Available from: https://www.nice.org.uk/guidance/ng242.

4. Blonde L, Umpierrez GE, Reddy SS, McGill JB, Berga SL, Bush M, et al. American Association of Clinical Endocrinology Clinical Practice Guideline: Developing a Diabetes Mellitus Comprehensive Care Plan-2022 Update. Endocr Pract. 2022;28(10):923-1049. doi:10.1016/j.eprac.2022.08.002.

5. Fundus Disease Group of Ophthalmological Society of Chinese Medical Association; Fundus Disease Group of Ophthalmologist Branch of Chinese Medical Doctor Association. Evidence-based guidelines for diagnosis and treatment of diabetic retinopathy in China (2022). Chin J Ocul Fundus Dis. 2023;39(2):99-124. doi:10.3760/cma.j.cn511434-20230110-00018.

6. Ng DSC, Ruamviboonsuk P, Apte RS, Bajimaya S, Chan CKM, Chang A, et al. International consensuses and controversies on causes, diagnosis and management of diabetic macular edema (DME). Prog Retin Eye Res. 2025;109:101406. doi:10.1016/j.preteyeres.2025.101406.

7. Lam RF, Radke NV, et al. International consensuses and guidelines on management of proliferative diabetic retinopathy (PDR) by the Asia-Pacific Vitreo-retina Society (APVRS), the Academy of Asia-Pacific Professors of Ophthalmology (AAPPO) and the Academia Retina Internationalis (ARI). Asia Pac J Ophthalmol (Phila). 2025;14(6):100262. doi:10.1016/j.apjo.2025.100262.

8. Yonekawa Y, Modi YS, Kim LA, Skondra D, Kim JE, Wykoff CC. American Society of Retina Specialists Clinical Practice Guidelines: Management of Nonproliferative and Proliferative Diabetic Retinopathy Without Diabetic Macular Edema. J Vitreoretin Dis. 2020;4(2):125-135. doi:10.1177/2474126419893829.

9. Horton MB, Brady CJ, Cavallerano J, Abramoff MD, Barker GP, Chiang MF, et al. Practice Guidelines for Ocular Telehealth-Diabetic Retinopathy, Third Edition. Telemed J E Health. 2020;26(4):495-543. doi:10.1089/tmj.2020.0006.

10. Wong TY, Sun J, Kawasaki R, Ruamviboonsuk P, Gupta N, Lansingh VC, et al. Guidelines on Diabetic Eye Care: The International Council of Ophthalmology Recommendations for Screening, Follow-up, Referral, and Treatment Based on Resource Settings. Ophthalmology. 2018;125(10):1608-1622. doi:10.1016/j.ophtha.2018.04.007.

11. Early Treatment Diabetic Retinopathy Study Research Group. Photocoagulation for diabetic macular edema: Early Treatment Diabetic Retinopathy Study Report Number 1. Arch Ophthalmol. 1985;103(12):1796-1806.

12. Diabetic Retinopathy Study Research Group. Photocoagulation treatment of proliferative diabetic retinopathy: clinical application of Diabetic Retinopathy Study findings, DRS Report Number 8. Ophthalmology. 1981;88(7):583-600.

13. Wells JA, Glassman AR, Ayala AR, Jampol LM, Aiello LP, Antoszyk AN, et al.; Diabetic Retinopathy Clinical Research Network. Aflibercept, bevacizumab, or ranibizumab for diabetic macular edema. N Engl J Med. 2015;372(13):1193-1203. doi:10.1056/NEJMoa1414264.

14. Wells JA, Glassman AR, Ayala AR, Jampol LM, Bressler NM, Bressler SB, et al.; Diabetic Retinopathy Clinical Research Network. Aflibercept, bevacizumab, or ranibizumab for diabetic macular edema: two-year results from a comparative effectiveness randomized clinical trial. Ophthalmology. 2016;123(6):1351-1359. doi:10.1016/j.ophtha.2016.02.022.

15. Gross JG, Glassman AR, Jampol LM, Inusah S, Aiello LP, Antoszyk AN, et al.; Writing Committee for the Diabetic Retinopathy Clinical Research Network. Panretinal photocoagulation vs intravitreous ranibizumab for proliferative diabetic retinopathy: a randomized clinical trial. JAMA. 2015;314(20):2137-2146. doi:10.1001/jama.2015.15217.

16. Gross JG, Glassman AR, Liu D, Sun JK, Antoszyk AN, Baker CW, et al.; Diabetic Retinopathy Clinical Research Network. Five-year outcomes of panretinal photocoagulation vs intravitreous ranibizumab for proliferative diabetic retinopathy: a randomized clinical trial. JAMA Ophthalmol. 2018;136(10):1138-1148. doi:10.1001/jamaophthalmol.2018.3255.

17. Brown DM, Wykoff CC, Boyer D, Heier JS, Clark WL, Emanuelli A, et al. Evaluation of intravitreal aflibercept for the treatment of severe nonproliferative diabetic retinopathy: results from the PANORAMA randomized clinical trial. JAMA Ophthalmol. 2021;139(9):946-955. doi:10.1001/jamaophthalmol.2021.2809.

18. Nguyen QD, Brown DM, Marcus DM, Boyer DS, Patel S, Feiner L, et al.; RISE and RIDE Research Group. Ranibizumab for diabetic macular edema: results from 2 phase III randomized trials: RISE and RIDE. Ophthalmology. 2012;119(4):789-801. doi:10.1016/j.ophtha.2011.12.039.

19. Korobelnik JF, Do DV, Schmidt-Erfurth U, Boyer DS, Holz FG, Heier JS, et al. Intravitreal aflibercept for diabetic macular edema. Ophthalmology. 2014;121(11):2247-2254. doi:10.1016/j.ophtha.2014.05.006.

20. Abramoff MD, Lavin PT, Birch M, Shah N, Folk JC. Pivotal trial of an autonomous AI-based diagnostic system for detection of diabetic retinopathy in primary care offices. NPJ Digit Med. 2018;1:39. doi:10.1038/s41746-018-0040-6.

21. Rajesh AE, Davidson OQ, Lee CS, Lee AY. Artificial Intelligence and Diabetic Retinopathy: AI Framework, Prospective Studies, Head-to-head Validation, and Cost-effectiveness. Diabetes Care. 2023;46(10):1728-1739. doi:10.2337/dci23-0032.
