# Supplementary material for: Large language models as sources of diabetic retinopathy patient education: a comparative evaluation of safety, health information quality, and readability
Source: Front Public Health. 2026 Oct 9;14:1945318. doi: 10.3389/fpubh.2026.1945318 (PMC13690771; doi:10.3389/fpubh.2026.1945318)
Supplement: Supplementary file 4 [file Supplementary_file_4.docx]

**Supplementary Material 4. Model Identifiers, Access Conditions, Query Protocol,**

**and Complete Q&A Record**

**Purpose.** This supplementary material documents the model labels, public-interface disclosure status, access conditions, prompting procedure, and complete archived responses used in the comparative evaluation of six large language model chatbot interfaces for diabetic retinopathy patient questions. Information on model identifiers and access conditions was recorded as displayed in the public user interface during the April 2026 data-collection period. The complete Q&A record presents the 50 final patient-oriented questions and the corresponding responses generated by each model interface.

**Table S4-1. Model identifiers and public-interface disclosure status**

| **Variable** | **ChatGPT** | **Gemini** | **DeepSeek** | **Doubao** | **Microsoft Copilot** | **Perplexity** |
| --- | --- | --- | --- | --- | --- | --- |
| **Platform name** | ChatGPT | Gemini | DeepSeek | Doubao | Microsoft Copilot | Perplexity |
| **Provider/vendor** | OpenAI | Google | DeepSeek | ByteDance | Microsoft | Perplexity AI |
| **Displayed model/service label in interface** | GPT-5.5 | Gemini 3.1 Pro | DeepSeek-V4 | Doubao 2.0 | Not visible / not disclosed | Perplexity Pro |
| **Underlying model disclosed by interface** | Displayed as GPT-5.5; technical details not fully disclosed | Displayed as Gemini 3.1 Pro; technical details not fully disclosed | Displayed as DeepSeek-V4; service configuration not fully disclosed | Displayed as Doubao 2.0; service configuration not fully disclosed | Not disclosed in the user interface | Perplexity Pro service mode; underlying model routing not fully disclosed |
| **Release date visible to users** | Not visible in the user interface at testing | Not visible in the user interface at testing | Not visible in the user interface at testing | Not visible in the user interface at testing | Not visible in the user interface at testing | Not visible in the user interface at testing |
| **Last update date visible to users** | Not visible in the user interface at testing | Not visible in the user interface at testing | Not visible in the user interface at testing | Not visible in the user interface at testing | Not visible in the user interface at testing | Not visible in the user interface at testing |
| **Open-source / open-weight / proprietary status** | Proprietary / closed-source public interface | Proprietary / closed-source public interface | Public chatbot interface; model/service configuration not fully disclosed | Proprietary / closed-source public interface | Proprietary / closed-source public interface | Proprietary public interface; model routing not fully disclosed |
| **Base, tuned, or fine-tuned status disclosed** | Not disclosed in the user interface | Not disclosed in the user interface | Not disclosed in the user interface | Not disclosed in the user interface | Not disclosed in the user interface | Not disclosed in the user interface |
| **Access route** | Official public web interface or app | Official public web interface or app | Official public web interface or app | Official public web interface or app | Official public web interface or app | Official public web interface or app |
| **Access URL or app name** | Official public interface/app; exact URL or app build not archived | Official public interface/app; exact URL or app build not archived | Official public interface/app; exact URL or app build not archived | Official public interface/app; exact URL or app build not archived | Official public interface/app; exact URL or app build not archived | Official public interface/app; exact URL or app build not archived |
| **Account type used** | Account tier not separately archived | Account tier not separately archived | Account tier not separately archived | Account tier not separately archived | Account tier not separately archived | Account tier not separately archived |

**Table S4-2. Query environment and prompting conditions**

| **Variable** | **Recorded value** |
| --- | --- |
| **Query date range** | April 10-15, 2026 |
| **Query location** | Wuhan, Hubei, China |
| **Time zone** | China Standard Time (UTC+8) |
| **Device and operating system** | Not archived in the study metadata |
| **Browser or app version** | Not archived in the study metadata |
| **Network environment** | Not archived in the study metadata |
| **Language of queries** | English |
| **Search / browsing / web-access mode** | Default platform setting; no additional browsing setting was manually enabled |
| **Citation or source-link mode** | Default platform setting; source links or citations were not specifically requested in the prompt |
| **File upload used** | No |
| **Image upload used** | No |
| **Additional system prompt used** | No |
| **Role instruction used** | No |
| **Hidden clinical context supplied** | No |
| **Follow-up prompting used** | No |
| **New chat session used for each question** | Yes |
| **Previous conversation history cleared when possible** | Yes |
| **Platform settings** | Default settings |
| **Prompt content** | The 50 final patient-oriented diabetic retinopathy questions were submitted verbatim as single-turn prompts |
| **Number of questions submitted per model** | 50 |
| **Number of responses analyzed per model** | 50 |
| **Total number of model responses analyzed** | 300 |

**Table S4-3. Response collection by model**

| **Model interface** | **Displayed model/service label** | **Questions submitted** | **Responses analyzed** | **Model-specific note** |
| --- | --- | --- | --- | --- |
| **ChatGPT** | GPT-5.5 | 50 | 50 | Findings should be interpreted as a time-stamped benchmark of the public interface. |
| **Gemini** | Gemini 3.1 Pro | 50 | 50 | Findings should be interpreted as a time-stamped benchmark of the public interface. |
| **DeepSeek** | DeepSeek-V4 | 50 | 50 | Findings should be interpreted as a time-stamped benchmark of the public interface. |
| **Doubao** | Doubao 2.0 | 50 | 50 | Findings should be interpreted as a time-stamped benchmark of the public interface. |
| **Microsoft Copilot** | Not visible / not disclosed | 50 | 50 | The user interface did not display an explicit model version at the time of testing. |
| **Perplexity** | Perplexity Pro | 50 | 50 | Perplexity Pro was recorded as the service mode; underlying model routing was not fully disclosed. |

**Section S4-4. Complete Structured Q&A Record**

The following section presents the complete archived responses for the 50 final patient-oriented diabetic retinopathy questions. For each question, responses are listed by model interface in the order archived during data collection. The text is presented as the research record used for subsequent evaluation and does not represent clinical advice.

**Q1. What is diabetic retinopathy?**

**model: ChatGPT**

*answer:*

Diabetic retinopathy is a complication of Diabetes Mellitus that affects the eyes-specifically the retina, the light-sensitive tissue at the back of the eye.

What happens?

Over time, high blood sugar damages the tiny blood vessels in the retina. These vessels can:

Leak fluid or blood

Become blocked

Grow abnormally (in advanced stages)

This damage interferes with normal vision and can eventually lead to blindness if untreated.

Stages of diabetic retinopathy

1. Early stage (Non-proliferative)

Small bulges (microaneurysms) form in blood vessels

Blood or fluid may leak into the retina

May cause swelling (macular edema)

Often no noticeable symptoms at first

2. Advanced stage (Proliferative)

New, fragile blood vessels grow abnormally

These can bleed into the eye (vitreous hemorrhage)

Scar tissue may form and pull on the retina

Can lead to retinal detachment and severe vision loss

Symptoms

Early on, there may be no symptoms. As it progresses:

Blurred or fluctuating vision

Dark spots or floaters

Poor night vision

Vision loss

Risk factors

Long duration of diabetes

Poor blood sugar control

High blood pressure

High cholesterol

Pregnancy (in people with diabetes)

Treatment & prevention

Good blood sugar control is the most important prevention

Regular eye exams (even if vision seems fine)

Treatments include:

Laser therapy

Injections into the eye

Surgery (in advanced cases)

Key takeaway

Diabetic retinopathy is a common but preventable cause of blindness in people with diabetes-early detection and control of blood sugar can make a huge difference.

**model: Copilot**

*answer:*

Diabetic retinopathy is damage to the retina's blood vessels caused by long-term high blood sugar, and it is one of the leading causes of vision loss in people with diabetes. It often develops silently, without early symptoms, and progresses through stages that can eventually lead to blindness if untreated.

What diabetic retinopathy is

Diabetic retinopathy happens when high blood sugar weakens and damages the tiny blood vessels in the retina, the light-sensitive tissue at the back of the eye. These vessels may

Leak fluid or blood

Swell or close off

Trigger the growth of abnormal new vessels that bleed easily

Over time, these changes interfere with vision and can cause permanent damage.

The two major stages

Non-proliferative diabetic retinopathy (NPDR) - Early stage

Blood vessels weaken and leak. The retina may swell (macular edema), causing blurry vision.

Proliferative diabetic retinopathy (PDR) - Advanced stage

New, fragile blood vessels grow and bleed into the eye. Scar tissue can form, leading to retinal detachment or glaucoma.

Symptoms (often appear late)

Many people notice nothing early on. As it progresses, symptoms may include:

Floaters

Blurry or fluctuating vision

Dark or empty spots

Poor night vision

Sudden vision loss

How it's diagnosed

A dilated eye exam is the primary method. Additional tests include:

Optical coherence tomography (OCT) - scans retinal thickness

Fluorescein angiography - highlights leaking or blocked vessel

Treatment options

Treatment depends on severity:

Anti-VEGF injections (e.g., Eylea, Lucentis) to reduce swelling and slow progression

Laser therapy to seal leaking vessels or shrink abnormal ones

Vitrectomy surgery for severe bleeding or retinal detachment

Strict blood sugar, blood pressure, and cholesterol control

Prevention

Good diabetes management is the strongest protection:

Maintain healthy blood sugar (A1C goals set with your doctor)

Control blood pressure and cholesterol

Avoid smoking

Get a yearly dilated eye exam (more often if retinopathy is present)

**model: DeepSeek**

*answer:*

This is an excellent question, as diabetic retinopathy is a leading cause of blindness worldwide, but it is largely preventable with proper care.

The Simple Definition

Diabetic retinopathy is a diabetes complication that affects the eyes. It's caused by damage to the blood vessels of the light-sensitive tissue at the back of the eye (the retina).

Think of the retina like the film in a camera. If the film is damaged, the picture will be blurry or have blank spots. Likewise, if a person's retina is damaged by diabetic retinopathy, their vision becomes impaired.

The Underlying Problem: How High Blood Sugar Causes Damage

The root cause is chronically high blood sugar (glucose) levels, a hallmark of both Type 1 and Type 2 diabetes. Over time, excess sugar in the bloodstream can weaken and damage the tiny blood vessels (capillaries) throughout the body, including those in the retina.

The damage happens in two main stages:

Stage 1: Non-Proliferative Diabetic Retinopathy (NPDR) - The Early Stage

This is the most common form. The blood vessels in the retina weaken and develop tiny bulges called microaneurysms. These weak spots can leak fluid and small amounts of blood into the retina.

Symptoms: Often none in the early stage. This is why regular eye exams are critical.

Consequences: The leaking fluid can cause the retina to swell. When the central part of the retina (the macula, responsible for sharp, central vision) swells, it's called macular edema. This is the most common reason for vision loss in people with diabetic retinopathy.

Stage 2: Proliferative Diabetic Retinopathy (PDR) - The Advanced Stage

As the disease progresses, the lack of blood flow (ischemia) caused by the damaged vessels triggers the retina to send signals for the growth of new, abnormal blood vessels. This process is called proliferation (hence the name).

The Problem: These new vessels are fragile and grow in the wrong places (like the surface of the retina and into the vitreous gel that fills the eye).

The Dangers:

Bleeding: The new vessels are very prone to leaking and breaking, causing significant bleeding (vitreous hemorrhage) that blocks vision.

Scarring: The bleeding and abnormal growth can lead to scar tissue, which can contract and pull the retina out of its normal position, causing a retinal detachment (a medical emergency).

Glaucoma: The new vessels can grow on the eye's drainage system, blocking it and leading to a painful increase in eye pressure (neovascular glaucoma).

Bleeding: The new vessels are very prone to leaking and breaking, causing significant bleeding (vitreous hemorrhage) that blocks vision.

Scarring: The bleeding and abnormal growth can lead to scar tissue, which can contract and pull the retina out of its normal position, causing a retinal detachment (a medical emergency).

Glaucoma: The new vessels can grow on the eye's drainage system, blocking it and leading to a painful increase in eye pressure (neovascular glaucoma).

Key Risk Factors

While anyone with diabetes is at risk, certain factors increase the chances of developing retinopathy:

Duration of Diabetes: The single biggest risk factor. The longer you've had diabetes (especially 10+ years), the higher your risk.

Poor Blood Sugar Control: Consistently high blood glucose levels accelerate damage.

High Blood Pressure (Hypertension): This puts extra stress on the already weakened retinal vessels.

High Cholesterol: Can lead to fatty deposits in the vessels.

Pregnancy: Women with diabetes are at higher risk during pregnancy.

Anemia or Kidney Disease: These are also linked to worsening retinopathy.

Duration of Diabetes: The single biggest risk factor. The longer you've had diabetes (especially 10+ years), the higher your risk.

Poor Blood Sugar Control: Consistently high blood glucose levels accelerate damage.

High Blood Pressure (Hypertension): This puts extra stress on the already weakened retinal vessels.

High Cholesterol: Can lead to fatty deposits in the vessels.

Pregnancy: Women with diabetes are at higher risk during pregnancy.

Anemia or Kidney Disease: These are also linked to worsening retinopathy.

Symptoms to Watch For

In the early stages, there are often no warning signs. As the disease progresses, symptoms may include:

Gradually blurry or "wavy" vision.

Spots or dark strings floating in your vision (floaters).

Fluctuating vision (clear one day, blurry the next).

Dark or empty areas in your vision.

Difficulty seeing at night.

Difficulty with color perception.

Sudden, complete loss of vision (in advanced cases).

Gradually blurry or "wavy" vision.

Spots or dark strings floating in your vision (floaters).

Fluctuating vision (clear one day, blurry the next).

Dark or empty areas in your vision.

Difficulty seeing at night.

Difficulty with color perception.

Sudden, complete loss of vision (in advanced cases).

Diagnosis and Treatment

Diagnosis: Through a comprehensive dilated eye exam. The eye doctor puts drops in your eyes to widen the pupils, allowing them to clearly see the retina and blood vessels. A special test called OCT (Optical Coherence Tomography) creates a 3D image of the retina to look for swelling.

Treatment: Treatments are very effective, especially when caught early.

Best Treatment: Prevention. The most important step is strict control of blood sugar, blood pressure, and cholesterol.

Injections (Anti-VEGF drugs): Medications like Eylea, Lucentis, or Avastin are injected into the eye to stop the growth of abnormal vessels and reduce macular edema.

Laser Surgery (Photocoagulation): A laser is used to seal leaking blood vessels or, in advanced cases, to shrink abnormal ones.

Vitrectomy: A surgical procedure that removes blood and scar tissue from the center of the eye (the vitreous) in advanced PDR cases.

Diagnosis: Through a comprehensive dilated eye exam. The eye doctor puts drops in your eyes to widen the pupils, allowing them to clearly see the retina and blood vessels. A special test called OCT (Optical Coherence Tomography) creates a 3D image of the retina to look for swelling.

Treatment: Treatments are very effective, especially when caught early.

Best Treatment: Prevention. The most important step is strict control of blood sugar, blood pressure, and cholesterol.

Injections (Anti-VEGF drugs): Medications like Eylea, Lucentis, or Avastin are injected into the eye to stop the growth of abnormal vessels and reduce macular edema.

Laser Surgery (Photocoagulation): A laser is used to seal leaking blood vessels or, in advanced cases, to shrink abnormal ones.

Vitrectomy: A surgical procedure that removes blood and scar tissue from the center of the eye (the vitreous) in advanced PDR cases.

The Most Important Takeaway

Diabetic retinopathy is a "silent" disease in its early, most treatable stages. You can have significant retinal damage and not notice any change in your vision until it's advanced and harder to treat.

This is why the American Diabetes Association recommends that everyone with diabetes have a comprehensive dilated eye exam at least once a year, or more frequently as advised by their eye doctor.

**model: Doubao**

*answer:*

Diabetic retinopathy is a microvascular complication of diabetes mellitus that damages the retina (the light-sensitive nerve tissue at the back of the eye).

Core Cause

Prolonged high blood sugar damages the tiny blood vessels in the retina over time. These vessels may:

Leak fluid, blood, or lipids into retinal tissue

Become blocked, cutting off oxygen and nutrients to retinal cells

Grow abnormal, fragile new blood vessels (neovascularization)

Key Classification

Non-proliferative Diabetic Retinopathy (NPDR)

Early stage: Microaneurysms, retinal hemorrhages, hard exudates, macular edema. Often no obvious symptoms at first.

Proliferative Diabetic Retinopathy (PDR)

Advanced stage: Abnormal new blood vessels grow on the retina and vitreous. Can cause vitreous hemorrhage, retinal detachment, and permanent vision loss or blindness.

High-Risk & Impact

Long-standing type 1 / type 2 diabetes, poor blood sugar/blood pressure/lipid control increase risk.

It is one of the leading causes of preventable blindness in working-age adults worldwide.

Main Symptoms (Late Stage)

Blurred vision

Floaters

Dark spots

Distorted vision

Sudden vision loss

Screening & Management

Regular annual eye fundus screening for all diabetic patients.

Control blood glucose, BP, lipids; treat macular edema with intravitreal injections, laser photocoagulation, or vitreoretinal surgery in severe cases.

**model: Gemini**

*answer:*

Diabetic retinopathy is a medical condition caused by high blood sugar levels damaging the back of the eye (the retina). It is the leading cause of vision loss among working-age adults.

How It Develops

The retina requires a constant supply of blood through a network of tiny blood vessels. Over time, persistent hyperglycemia (high blood sugar) damages these vessels in two primary ways:

1. Non-proliferative Diabetic Retinopathy (NPDR): This is the early stage. The tiny blood vessels leak fluid or blood into the retina, causing swelling. Small bulges called microaneurysms may also form.

2. Proliferative Diabetic Retinopathy (PDR): This is the advanced stage. Because the original vessels are damaged, the eye attempts to grow new ones. However, these new vessels are fragile and prone to leaking into the vitreous (the clear jelly in the center of the eye), which can lead to scar tissue or retinal detachment.

Common Symptoms

In the early stages, there are often no symptoms. As it progresses, a person might experience:

Floating spots or dark strings (floaters).

Blurred or fluctuating vision.

Dark or empty areas in the vision.

Difficulty seeing colors.

Key Management Strategies

Managing the condition focuses on preventing further damage and preserving existing vision:

Systemic Control: Keeping blood glucose, blood pressure, and cholesterol levels within target ranges is the most effective way to slow progression.

Regular Screenings: Annual dilated eye exams are critical because the condition can often be treated before vision loss occurs.

Medical Interventions: Anti-VEGF Injections: Medications that reduce swelling and stop the growth of new blood vessels.

Laser Treatment (Photocoagulation): Used to seal leaking vessels or shrink abnormal ones.

Vitrectomy: Surgery to remove blood or scar tissue from the center of the eye.

**model: Perplexity**

*answer:*

Diabetic retinopathy is an eye disease caused by diabetes that damages the blood vessels in the retina, the light-sensitive tissue at the back of the eye. It can lead to blurred vision and, if untreated, vision loss or blindness.

How it develops

High blood sugar over time weakens the tiny retinal blood vessels. Those vessels can leak, swell, or close off, and in later stages the eye may grow abnormal new vessels that are fragile and bleed easily.

Symptoms

Early on, it often causes no symptoms. Later signs can include blurry vision, floaters, trouble seeing at night, and vision changes in one or both eyes.

Why it matters

It is one of the main causes of preventable vision loss in people with diabetes, but early detection and treatment can reduce the risk

**Q2. Can diabetic retinopathy make me blind?**

**model: ChatGPT**

*answer:*

Yes, diabetic retinopathy (DR) can lead to blindness if it progresses without treatment. DR is a complication of diabetes that damages the blood vessels in the retina, the light-sensitive tissue at the back of the eye. There are two main stages:

1. Non-proliferative diabetic retinopathy (NPDR) - Early stage; blood vessels may leak fluid or bleed, but vision loss is often mild or absent.

2. Proliferative diabetic retinopathy (PDR) - Advanced stage; abnormal new blood vessels grow on the retina, which can cause severe bleeding, retinal detachment, and significant vision loss or blindness.

Other risks include diabetic macular edema (DME), where fluid accumulates in the central retina, affecting sharp vision.

Good blood sugar control, blood pressure management, regular eye exams, and timely treatments (like laser therapy, anti-VEGF injections, or surgery) greatly reduce the risk of blindness. Early detection is key because DR often has no symptoms in its early stages.

**model: Copilot**

*answer:*

Yes - diabetic retinopathy can cause blindness, but it is usually preventable with early detection and proper diabetes and eye care.

The core fact

Diabetic retinopathy is an eye disease caused by damage to the retina's blood vessels from high blood sugar. If it progresses untreated, it can lead to severe vision loss or complete blindness. This is confirmed by major medical sources, including the Mayo Clinic, which notes that diabetic retinopathy "can lead to blindness, especially if diabetes or other health problems are poorly managed."

How diabetic retinopathy leads to blindness

Retinal bleeding - Fragile new blood vessels can leak into the eye, blocking vision.

Macular edema - Swelling in the macula blurs central vision.

Retinal detachment - Scar tissue can pull the retina away, causing severe vision loss.

Neovascular glaucoma - New vessels block fluid drainage, raising eye pressure and damaging the optic nerve.

All of these complications are documented by the National Eye Institute as pathways to blindness.

The hopeful part: blindness is largely preventable

Most people do not go blind if they:

Keep blood sugar, blood pressure, and cholesterol under control

Get yearly dilated eye exams (or more often if advised)

Treat retinopathy early with injections, laser therapy, or surgery when needed

Early treatment can stop or slow progression, and sometimes even improve vision.

When to seek care immediately

If you notice:

Sudden blurry vision

New floaters

Dark spots or "curtains" in your vision

Rapid vision changes

These can be signs of bleeding or retinal detachment and need urgent evaluation.

**model: DeepSeek**

*answer:*

Yes, diabetic retinopathy can lead to blindness if it is not treated. However, it is important to know that blindness is not inevitable.

Here's the breakdown:

How it causes vision loss: High blood sugar damages the tiny blood vessels in your retina (the light-sensitive tissue at the back of your eye). These vessels can leak fluid or blood, or abnormal new vessels can grow. This leads to:

Macular edema: Swelling of the macula (the part of the retina responsible for sharp, central vision). This is the most common cause of vision loss from diabetic retinopathy.

Proliferative diabetic retinopathy (PDR): The advanced stage where abnormal new blood vessels grow on the retina. These vessels are fragile and bleed easily, leading to vitreous hemorrhage (blood in the gel of the eye), retinal detachment, or glaucoma.

The good news: With proper and timely treatment, severe vision loss can be prevented in the vast majority of cases. Treatments are very effective, especially when the disease is caught early.

Key factors that determine your risk:

Blood sugar control: The single most important factor. Tight control significantly slows or stops progression.

Blood pressure control: High blood pressure makes retinopathy worse.

Regular eye exams: You can have retinopathy for a long time without symptoms. A dilated eye exam by an ophthalmologist can detect it early, when treatment is most effective.

Summary: Diabetic retinopathy is a leading cause of blindness in working-age adults, but only if it is neglected. Regular eye exams, good blood sugar management, and timely treatment (laser, injections, or surgery) can reduce the risk of severe vision loss by over 90%.

**model: Doubao**

*answer:*

Yes, diabetic retinopathy absolutely can lead to permanent blindness if left undiagnosed or untreated.

Key points

1.High blood sugar damages tiny blood vessels in the retina, causing leakage, swelling, and abnormal new blood vessel growth.

2.Advanced stages (proliferative diabetic retinopathy, diabetic macular edema) can lead to retinal detachment, vitreous hemorrhage, and irreversible vision loss.

3.It's one of the top leading causes of preventable blindness in adults worldwide.

Good news

With regular eye screening, good blood sugar/blood pressure/lipid control, and timely treatment (laser, intravitreal injections, surgery), severe vision loss or blindness can almost always be prevented.

**model: Gemini**

*answer:*

It is understandable to be concerned about your vision when managing a condition like diabetes. To answer your question directly: yes, diabetic retinopathy can lead to blindness if it is left untreated.

However, it is important to know that total vision loss is often preventable with early detection and modern medical interventions.

How Vision Loss Occurs

Diabetic retinopathy damages the tiny blood vessels in the retina (the light-sensitive tissue at the back of the eye). Vision loss typically happens through a few different stages:

Macular Edema: This occurs when damaged blood vessels leak fluid into the macula, the part of the eye responsible for sharp, central vision. This can cause significant blurring.

Proliferative Retinopathy: In advanced stages, the retina begins growing new, fragile blood vessels. these can leak blood into the center of the eye (Vitreous Hemorrhage), blocking vision.

Retinal Detachment: The growth of new blood vessels can create scar tissue that eventually pulls the retina away from the back of the eye.

Glaucoma: New blood vessels can also grow in the front of the eye, interfering with fluid drainage and increasing eye pressure, which damages the optic nerve.

Prevention and Management

The risk of severe vision loss is significantly reduced through proactive care. Most experts recommend the following strategies:

1. Annual Dilated Eye Exams: Because early stages of retinopathy often have no symptoms, regular exams are the only way to catch damage before it affects your sight.

2. Glycemic Control: Keeping blood sugar levels within your target range is the most effective way to slow the progression of retinal damage.

3. Blood Pressure and Cholesterol: Managing these "co-factors" is crucial, as high blood pressure can further strain the delicate vessels in your eyes.

4. Modern Treatments: If the condition progresses, treatments such as anti-VEGF injections, laser therapy (photocoagulation), or vitrectomy surgery are highly effective at stabilizing vision.

5. If you notice any sudden changes-such as "floaters" that look like cobwebs, flashes of light, or dark spots-you should contact an ophthalmologist immediately.

**model: Perplexity**

*answer:*

Yes. Diabetic retinopathy can cause permanent vision loss and blindness, especially if it is not treated or if diabetes is poorly controlled.

What happens

High blood sugar damages the small blood vessels in the retina, which can lead to leaking, swelling, bleeding, retinal detachment, and eventually severe vision loss. It often starts with no symptoms, so people may not notice it until the damage is advanced.

Why treatment matters

The good news is that early detection and treatment can greatly reduce the risk of blindness. Regular eye exams, blood sugar control, blood pressure control, and treatment such as injections, laser therapy, or surgery when needed can help protect vision.

Get urgent care sooner

Seek prompt eye care if you notice blurred vision, floaters, dark spots, trouble seeing colors, or any sudden vision change. Sudden vision loss is an emergency

**Q3. What is diabetic macular edema, and how is it related to diabetic retinopathy?**

**model: ChatGPT**

*answer:*

Macular edema is a condition where fluid accumulates in the macula, the central part of the retina responsible for sharp, detailed vision. This swelling occurs when blood vessels in the retina leak fluid, which can distort vision and, if severe or untreated, lead to vision loss.

Its connection to diabetic retinopathy lies in the effect of high blood sugar on retinal blood vessels. In diabetic retinopathy, chronic hyperglycemia damages the small blood vessels in the retina, making them more permeable or prone to leakage. When this leakage happens specifically in the macula, it is called diabetic macular edema (DME). DME is actually one of the leading causes of vision loss in people with diabetes, even if the overall retinopathy is in early stages.

Key points:

Macular edema: Swelling in the macula due to fluid accumulation.

Diabetic retinopathy: Damage to retinal blood vessels caused by diabetes.

Connection: Damaged vessels leak fluid into the macula to diabetic macular edema to vision impairment.

Treatment for DME often includes anti-VEGF injections, corticosteroids, or laser therapy to reduce swelling and prevent further vision loss.

**model: Copilot**

*answer:*

Macular edema is the buildup of fluid in the macula - the part of the retina responsible for sharp, central vision - and it becomes blurry or distorted when this tissue swells. Its most common cause is diabetic retinopathy, where chronically high blood sugar damages retinal blood vessels, causing them to leak fluid into the macula.

What macular edema is

Macular edema occurs when weakened or damaged retinal blood vessels leak fluid into the macula, causing it to thicken and swell. This leads to symptoms such as:

Blurry or wavy central vision

Dull or faded colors

Objects appearing distorted or different sizes

How it relates to diabetic retinopathy

Diabetic retinopathy (DR) is a complication of diabetes that progressively damages the retina. High blood sugar weakens retinal capillaries, causing:

Microaneurysms (tiny bulges in blood vessels)

Leakage of fluid, proteins, and lipids

Breakdown of the blood-retina barrier

When this leakage occurs specifically in the macula, it leads to diabetic macular edema (DME) - the most common cause of vision loss in people with diabetes.

Key points about the relationship:

You can have diabetic retinopathy without macular edema, but not macular edema without diabetic retinopathy.

DME results from fluid accumulation due to damaged or abnormal retinal vessels caused by diabetes.

Poor blood sugar control, high blood pressure, and long-standing diabetes increase the risk.

Why this matters

Macular edema is the leading cause of vision loss in diabetic retinopathy. Early detection and management of diabetes - along with regular dilated eye exams - can prevent or slow progression.

**model: DeepSeek**

*answer:*

This is an excellent question, as it gets to the heart of why diabetes is a leading cause of vision loss.

Here's a breakdown of what macular edema is and its direct relationship to diabetic retinopathy.

What is Macular Edema?

In simplest terms, macular edema is a swelling (edema) of the macula.

The Macula: The macula is the small, central area of the retina (the light-sensitive tissue at the back of the eye). It's responsible for your sharp, central vision needed for activities like reading, driving, recognizing faces, and seeing fine detail.

Edema (Swelling): This swelling is caused by fluid leaking from damaged blood vessels into the macular tissue.

Think of the retina like a carpet. The macula is the finely woven, special central patch of that carpet. Macular edema is like spilling water on that central patch, causing it to become soggy, swollen, and distorted.

Symptoms of Macular Edema:

Blurred or wavy central vision (straight lines, like a doorframe or a line of text, may look bent or wavy).

Colors appear washed out or faded.

Difficulty reading (words may seem to jump around or disappear).

Increased sensitivity to glare.

Crucially, peripheral (side) vision is usually not affected. A person with macular edema might be able to walk around fine but cannot read a sentence or recognize a friend's face across a room.

How is it Related to Diabetic Retinopathy?

This is the most common and important cause of macular edema. The relationship is one of cause and effect, where diabetic retinopathy is the underlying disease that leads to the complication of macular edema.

Here is the step-by-step connection:

The Root Problem: High Blood Sugar. Chronically high blood sugar (glucose) damages the tiny blood vessels throughout the body, including the delicate capillaries in the retina.

Diabetic Retinopathy (DR) Develops. This damage to the retinal blood vessels is the disease called diabetic retinopathy. It initially has two main forms:

Non-Proliferative DR (NPDR): The early stage. Vessel walls weaken and develop tiny bulges called microaneurysms. These microaneurysms are fragile and leaky.

Leakage = Macular Edema. As diabetic retinopathy progresses (especially in NPDR), the weakened, leaky microaneurysms and damaged vessel walls begin to leak fluid, proteins, and fats (lipids). When this leakage happens directly in the macula, the macula swells. That swelling is diabetic macular edema (DME).

A Vicious Cycle. The edema fluid disrupts the careful organization of light-sensing cells in the macula, preventing them from sending clear signals to the brain. The longer the fluid remains, or the more it recurs, the higher the risk of permanent damage and vision loss.

A crucial distinction: You can have diabetic retinopathy without macular edema. Many people have background retinopathy (leaky vessels elsewhere in the retina) that doesn't affect the macula, and they may have no vision problems. Macular edema is the specific complication of DR that directly threatens central vision.

Summary Table: DR vs. DME

Feature; Diabetic Retinopathy (DR); Diabetic Macular Edema (DME)

What it is; The overall disease of damaged retinal blood vessels.; A specific complication of DR (fluid swelling in the macula).

Location; Throughout the entire retina.; Only in the central macula.

Primary Threat; Can lead to severe vision loss from bleeding or retinal detachment (in advanced stages).; Directly threatens sharp, central vision needed for reading, driving, etc.

Symptoms; Often none in early stages. May include floaters, dark spots, or vision fluctuations.; Blurred/wavy central vision, faded colors, reading difficulty.

Relation; The underlying cause.; The sight-threatening effect.

Treatment and Management

The goal is to stop the leakage and reduce the swelling.

Diabetic Macular Edema: The current gold-standard treatment is anti-VEGF injections (e.g., Lucentis, Eylea, Avastin). These drugs are injected directly into the vitreous gel of the eye to block the chemical (VEGF) that makes vessels leak. Laser (focal/grid laser) and steroid injections are other options.

Underlying Diabetic Retinopathy: Strict systemic control is essential. This means managing blood sugar, blood pressure, and cholesterol. Good control can slow the progression of DR and reduce the risk of developing DME in the first place.

The Most Important Takeaway

Diabetic macular edema is the primary reason why people with diabetes lose central vision. It is a direct consequence of the vessel damage caused by diabetic retinopathy. Because early diabetic retinopathy and even early macular edema often have no symptoms, regular, comprehensive dilated eye exams are absolutely critical for anyone with diabetes. Early detection and treatment (like anti-VEGF injections) can be highly effective at preserving vision.

**model: Doubao**

*answer:*

1. Definition of Macular Edema (ME)

Macular edema is the swelling and fluid accumulation in the macula-the central part of the retina responsible for central sharp vision, reading, facial recognition, and detailed eyesight.

It occurs when damaged retinal blood vessels leak fluid, lipids, and protein into the macular tissue, causing the macula to thicken and distort its normal structure.

2. What is Diabetic Retinopathy (DR)?

Diabetic retinopathy is a chronic microvascular complication of diabetes. Persistent high blood sugar damages the tiny blood vessels in the retina over time, leading to:

Vessel weakening, leakage, and blockage

Formation of microaneurysms

Retinal ischemia and abnormal new blood vessel growth (proliferative DR)

3. Relationship Between Macular Edema and Diabetic Retinopathy

Diabetic Macular Edema (DME) is the most common and visually disabling complication of diabetic retinopathy, and can occur at any stage of DR:

Pathological linkChronic hyperglycemia damages retinal capillary walls to increased vascular permeability to fluid/exudates leak into the macular layers to macular thickening and edema.

Clinical classification

DME can develop in non-proliferative diabetic retinopathy (NPDR)-the early stage-even without severe retinal vessel proliferation.

It also frequently accompanies proliferative diabetic retinopathy (PDR).

Vision impactWhile peripheral retina damage causes side vision loss, DME directly impairs central vision. It is the leading cause of irreversible central vision loss in working-age adults with diabetes worldwide.

Key connectionMacular edema is not a separate disease; DME is a direct secondary complication driven by the microvascular damage of diabetic retinopathy. Poor long-term blood glucose, blood pressure, and lipid control are the main risk factors for both progressive DR and onset/worsening of DME.

4. Brief Management Overview

Common interventions for DME:

Intravitreal anti-VEGF injections (first-line)

Intravitreal steroids

Focal/grid laser photocoagulation

Strict systemic control of blood sugar, BP, and lipids

**model: Gemini**

*answer:*

Macular edema is a condition where fluid builds up in the macula, the central part of the retina responsible for sharp, detailed vision. This fluid causes the macula to swell and thicken, distorting vision and making it difficult to perform tasks like reading, driving, or recognizing faces.

The relationship between macular edema and diabetic retinopathy is direct and physiological, often representing a significant progression of the disease.

1. The Pathological Connection

Diabetic retinopathy occurs when chronically high blood sugar levels damage the tiny blood vessels (capillaries) in the retina. This damage leads to two primary issues that cause macular edema:

Vascular Leakage: The damaged capillary walls become weak and "leaky." This allows fluid, proteins, and fats from the blood to seep into the retinal tissue.

Microaneurysms: Small, balloon-like swellings can form in the blood vessel walls. These microaneurysms often rupture or leak fluid directly into the macula.

When this occurs specifically due to diabetes, it is referred to as Diabetic Macular Edema (DME).

2. Stages of Development

Macular edema can occur at any stage of diabetic retinopathy, though it is more common as the disease progresses:

Non-proliferative Diabetic Retinopathy (NPDR): In the early stages, blood vessels leak, and the resulting swelling can cause vision to blur. This is the most common cause of vision loss in people with NPDR.

Proliferative Diabetic Retinopathy (PDR): In advanced stages, the retina may begin growing new, fragile blood vessels (neovascularization). These vessels are highly prone to leaking, significantly increasing the risk of severe DME.

3. Symptoms and Impact

While early diabetic retinopathy may have no symptoms, the onset of macular edema usually triggers noticeable changes:

Blurred or "wavy" central vision.

Colors appearing washed out or faded.

Dark spots or "holes" in the center of the visual field.

4. Comparison of the Two Conditions

Feature; Diabetic Retinopathy; Macular Edema (DME)

Definition; General damage to retinal blood vessels due to diabetes.; Swelling of the central retina due to fluid accumulation.

Location; Can affect the entire retina (peripheral and central).; Specifically affects the macula (central vision).

Vision Loss; Can be gradual or sudden (e.g., from vitreous hemorrhage).; Primarily affects central detail and clarity.

Relationship; The underlying disease process.; A specific complication caused by the disease.

Management typically involves controlling blood sugar, blood pressure, and cholesterol, alongside ophthalmic treatments such as anti-VEGF injections, laser therapy, or corticosteroids to reduce swelling and prevent further vision loss.

**model: Perplexity**

*answer:*

Diabetic macular edema (DME) is swelling in the macula, the part of the retina responsible for sharp central vision, caused by fluid leaking from damaged blood vessels in people with diabetes. It is closely related to diabetic retinopathy because it usually develops as a complication of that eye disease, which damages the retina's blood vessels.

How they're connected

Diabetic retinopathy is the broader disease: diabetes damages the blood vessels in the retina.

Diabetic macular edema is a specific outcome of that damage: the leaky vessels allow fluid to collect in the macula, making it swell.

Not everyone with diabetic retinopathy develops DME, but DME is one of the main causes of vision loss in people with diabetic retinopathy.

What it can cause

DME can lead to blurry or distorted vision, trouble reading, and difficulty seeing fine detail because the macula handles central vision. Since the macula is central to detailed sight, swelling there can affect daily tasks more than retinopathy in other parts of the retina.

In one sentence

Diabetic retinopathy is the underlying retinal blood-vessel disease, and diabetic macular edema is when that disease causes fluid buildup and swelling in the macula

**Q4. Can diabetic retinopathy be reversed or cured?**

**model: ChatGPT**

*answer:*

Diabetic retinopathy (DR) is generally not considered fully reversible or curable, but its progression can be slowed, managed, and in some cases partially improved with proper treatment and blood sugar control. Here's a breakdown:

1. Early Stage (Non-Proliferative DR)

1. At this stage, the damage to the retinal blood vessels is often mild.

2. Tight blood sugar, blood pressure, and cholesterol control can prevent progression and sometimes improve mild retinal changes.

3. Vision loss is often preventable if detected early.

2. Advanced Stage (Proliferative DR or Diabetic Macular Edema)

1. Treatments such as laser photocoagulation, anti-VEGF injections, and vitrectomy surgery can reduce further vision loss and, in some cases, improve vision.

2. However, these treatments do not completely reverse existing retinal damage. The goal is mainly to preserve remaining vision.

3. Long-Term Management

1. Lifelong monitoring is essential because DR can recur or worsen even after treatment.

2. Good systemic management of diabetes remains the cornerstone for prevention and slowing progression.

Summary: While DR cannot be "cured" in the conventional sense, early detection, proper diabetic control, and timely ophthalmic treatments can halt progression, prevent blindness, and sometimes partially restore vision.

**model: Copilot**

*answer:*

Short answer: Diabetic retinopathy cannot be fully "cured", but its progression can be stopped, and some vision can improve-especially when treated early and when blood sugar, blood pressure, and cholesterol are well controlled. Treatments like anti-VEGF injections, laser therapy, and vitrectomy can reverse certain changes or prevent further damage, but permanent retinal damage cannot be undone.

What "reversal" really means

Reversal in diabetic retinopathy usually refers to:

Reduced swelling of the macula after treatment

Regression of abnormal blood vessels with anti-VEGF injections

Improved vision when swelling decreases

But:

Dead retinal cells cannot regenerate, so any vision lost from that damage is typically permanent.

Advanced stages (PDR) often leave scarring that cannot be reversed.

What treatments can improve or stabilize vision?

1. Anti-VEGF injections

These medications (Eylea, Lucentis, Avastin) can:

Reduce macular swelling

Improve vision

Cause abnormal blood vessels to shrink

2. Laser therapy

Used to:

Seal leaking vessels

Prevent growth of new abnormal vessels

Reduce risk of severe vision loss

3. Vitrectomy surgery

Recommended for:

Severe bleeding into the vitreous

Scar tissue pulling on the retina

Retinal detachment

4. Medical control

Good control of:

Blood sugar (A1C)

Blood pressure

Cholesterol

...can stop progression and sometimes restore some vision.

What can and cannot be reversed?

Stage; What can improve?; What cannot be reversed?

Mild NPDR; Swelling, leakage, early vessel changes; None-if treated early

Moderate NPDR; Macular edema, some vessel leakage; Cell loss from ischemia

Severe NPDR; Swelling, some leakage; Widespread vessel closure

PDR (advanced); Regression of new vessels, clearing of blood after vitrectomy; Scar tissue, retinal detachment damage

What matters most for long-term outcomes

Early detection - yearly dilated eye exams

Tight glucose control (A1C goal often <7%, individualized)

Blood pressure control

Cholesterol management

No smoking

Bottom line

Diabetic retinopathy is not curable, but it is highly treatable. Many people maintain good vision for life with early detection and proper management.

**model: DeepSeek**

*answer:*

The short answer is: In its advanced stages, diabetic retinopathy cannot be fully "cured" or reversed to a completely normal, pre-diabetic state. However, early-stage diabetic retinopathy can show significant improvement or even regression, and vision loss can often be prevented with timely treatment.

Let's break this down by the stages of the disease.

1. Early, Non-Proliferative Diabetic Retinopathy (NPDR)

In this earliest stage, high blood sugar weakens the tiny blood vessels in the retina, causing them to develop tiny bulges (microaneurysms) that can leak fluid or small amounts of blood.

Can it be reversed? Partially, yes. Research shows that if you dramatically improve your blood sugar control (and blood pressure/cholesterol) at this stage, the retinal changes can regress. The microaneurysms may become less active, and leakage can decrease.

How to achieve this: Intensive glycemic control (lowering your HbA1c) is the key. Studies like the DCCT (Diabetes Control and Complications Trial) proved that strict blood sugar management reduces the risk of developing retinopathy and can slow the progression of early-stage disease. In some cases, the retina can return to nearly normal.

2. Advanced, Proliferative Diabetic Retinopathy (PDR)

This is a more severe stage. The damaged, oxygen-starved retina responds by growing abnormal, fragile new blood vessels (a process called neovascularization). These vessels are worthless-they don't provide proper oxygen, and they are very prone to bleeding (vitreous hemorrhage), which causes sudden vision loss. Scar tissue can also form, leading to retinal detachment.

Can it be reversed? No, the structural damage cannot be reversed. Once these abnormal, fragile blood vessels have grown, they will not turn back into healthy vessels. The scar tissue will not disappear on its own.

But can it be treated and vision saved? Absolutely, yes. Treatment aims to stop the disease process and prevent further vision loss, not to "cure" the existing damage. The primary goals are to:

Make the abnormal vessels shrink or regress.

Prevent future bleeding and retinal detachment.

Effective treatments for PDR include:

Anti-VEGF Injections: Drugs injected into the eye (e.g., Eylea, Lucentis, Avastin) block the chemical signal (VEGF) that causes abnormal vessels to grow. These injections can cause the existing abnormal vessels to shrink significantly and stop the growth of new ones.

Panretinal Photocoagulation (PRP) Laser: This laser treatment creates tiny burns in the peripheral retina. This reduces the retina's demand for oxygen, which lowers the production of VEGF. It is very effective at making abnormal vessels regress and preventing future severe vision loss.

3. Diabetic Macular Edema (DME)

This is swelling in the macula (the central part of the retina responsible for sharp, detailed vision). DME can occur at any stage of retinopathy and is the most common cause of vision loss in people with diabetes.

Can it be reversed? Usually, yes. The swelling (edema) can be effectively treated and reduced.

Treatment: The gold standard is Anti-VEGF injections. Multiple studies show these injections can dry up the macular swelling, leading to significant improvement in vision for many patients. Sometimes, laser (focal/grid laser) or steroid injections are also used.

Summary Table: Reversal vs. Treatment

Condition; Can it be fully reversed/cured?; Can it be improved or controlled with treatment?; Primary Goal of Treatment

Early NPDR (mild); Potentially yes, with strict blood sugar control.; Yes.; Prevent progression to advanced disease.

PDR (abnormal vessels); No, the fragile, abnormal vessels represent permanent change.; Yes. Anti-VEGF or laser can make abnormal vessels regress.; Prevent severe vision loss from bleeding/detachment.

DME (macular swelling); The swelling can be reversed, restoring vision.; Yes. Anti-VEGF injections are highly effective at drying the macula.; Improve or stabilize central vision.

The Most Important Takeaway: Prevention is Key

While a "cure" for advanced damage isn't possible, you have immense power to prevent ever reaching that stage.

The single most effective thing you can do is manage your systemic health:

Control your blood sugar (HbA1c). This is the #1 factor.

Control your blood pressure. Hypertension dramatically accelerates retinopathy.

Control your cholesterol.

Get a comprehensive, dilated eye exam every year (or more often if recommended by your ophthalmologist). Early detection allows treatment before permanent vision loss occurs.

In conclusion: Don't think of it as "all or nothing." While advanced diabetic retinopathy is not curable, vision loss from the most common blinding complications (PDR and DME) is often preventable and treatable. For early disease, dramatic improvement is even possible. The key is early detection through regular eye exams and aggressive systemic control. If you have diabetes, see an ophthalmologist regularly-it truly saves sight.

**model: Doubao**

*answer:*

Diabetic retinopathy (DR) cannot be fully cured permanently because the underlying cause-chronic diabetes and long-term blood glucose/vascular damage-is a lifelong condition. However:

Early-stage mild DR (non-proliferative) can be partially reversed and stabilized with strict metabolic control.

Advanced DR (proliferative DR, macular edema) cannot be reversed completely, but progression can be halted, vision loss can be prevented or reduced with timely treatment.

1. Early Non-Proliferative Diabetic Retinopathy

Mild microaneurysms, minor retinal hemorrhages:Tight control of blood sugar, blood pressure, blood lipids can make mild retinal lesions regress partially, reduce exudates and bleeding, and keep vision stable long-term.

No irreversible retinal nerve damage at this stage, so functional recovery is possible.

2. Diabetic Macular Edema (DME)

Cannot be fully cured, but edema can be resolved significantly:Anti-VEGF intravitreal injections, steroid implants, laser therapy can reduce macular swelling, recover partial lost vision and prevent further visual decline.

Recurrence is common, requiring long-term follow-up and repeated treatment.

3. Advanced Proliferative Diabetic Retinopathy (PDR)

Neovascularization, vitreous hemorrhage, retinal detachment:These structural damages are irreversible.

Treatments (laser photocoagulation, vitrectomy) only control complications, save remaining vision, prevent blindness-they cannot restore already lost retinal nerve cells or cure the disease fundamentally.

4. Core Fundamental Point

DR is a chronic progressive vascular complication of diabetes. Even if lesions improve or stabilize, the diabetic microvascular vulnerability remains. Lifelong metabolic management and regular retinal screening are mandatory to avoid recurrence or progression.

**model: Gemini**

*answer:*

Whether diabetic retinopathy (DR) can be "reversed" or "cured" depends largely on the stage of the disease and how strictly the underlying cause-diabetes-is managed.

In short: While it is generally not considered "curable" in the sense that the damage can be permanently erased, it is highly manageable, and early-stage changes can sometimes be reversed.

1. Early Stages (Non-Proliferative)

In the early stages, known as Non-Proliferative Diabetic Retinopathy (NPDR), the blood vessels in the retina begin to swell or leak.

Reversibility: If blood sugar, blood pressure, and cholesterol levels are brought into a healthy range, some of the early clinical signs (like microaneurysms or minor hemorrhages) can actually regress or disappear.

The Goal: At this stage, the focus is on metabolic control to prevent the disease from progressing to more severe forms.

2. Advanced Stages (Proliferative)

Proliferative Diabetic Retinopathy (PDR) occurs when the retina begins growing new, fragile blood vessels that bleed easily and can cause scarring or retinal detachment.

Reversibility: Damage at this stage-such as significant scarring or vision loss from a detached retina-is generally permanent.

Management: Modern treatments are very effective at stopping further progression, but they typically don't restore the eye to its "pre-disease" state.

Common Clinical Interventions

While these treatments don't "cure" the diabetes causing the issue, they are used to stabilize the retina and protect remaining vision:

Anti-VEGF Injections: Medications injected into the eye to reduce swelling and stop the growth of abnormal blood vessels. This can sometimes lead to significant improvement in visual acuity.

Laser Treatment (Photocoagulation): Used to shrink abnormal blood vessels or seal leaking ones. While it saves central vision, it can sometimes reduce peripheral or night vision.

Vitrectomy: A surgery to remove blood or scar tissue from the center of the eye in very advanced cases.

Summary Table

Feature; Early Stage (NPDR); Advanced Stage (PDR)

Primary Issue; Leaky/swollen vessels; New, fragile vessel growth

Can it be reversed?; Yes, often through strict glucose control.; No, damage is usually permanent.

Main Goal; Prevention and stabilization.; Vision preservation and surgery.

Visual Recovery; High potential for stable vision.; Guarded; depends on the extent of damage.

The "Bottom Line"

The most important factor is that diabetic retinopathy is often asymptomatic until it reaches a late stage. Because the "cure" is essentially lifelong management of blood glucose, regular dilated eye exams are the only way to catch the disease in the phase where it is still reversible.

**model: Perplexity**

*answer:*

Diabetic retinopathy usually cannot be fully cured, but early stages can sometimes be stabilized, and some vision loss from swelling or bleeding can improve with treatment.

What can improve

Better blood sugar, blood pressure, and cholesterol control can slow or stop progression, especially when caught early.

Treatments such as laser therapy, anti-VEGF injections, corticosteroid injections, and vitrectomy can reduce damage and sometimes improve vision, depending on the stage.

What usually cannot be reversed

Once retinal blood vessels are significantly damaged or scar tissue has formed, that damage is generally not fully reversible.

Advanced proliferative disease is especially difficult to reverse, so preventing progression is the main goal.

Practical takeaway

The best outcome is usually early detection and treatment, because that offers the greatest chance to preserve vision and possibly regain some clarity if swelling is driving the symptoms.

**Q5. Can children or teenagers with diabetes develop diabetic retinopathy?**

**model: ChatGPT**

*answer:*

Yes, children and teenagers with diabetes can develop diabetic retinopathy (DR), although it is less common than in adults. The risk largely depends on:

1. Duration of diabetes: DR is more likely to appear after having diabetes for several years. In children and adolescents, it usually develops after 5-10 years of disease onset.

2. Type of diabetes: Both type 1 and type 2 diabetes can cause DR. In type 1 diabetes, which often begins in childhood, screening is recommended starting about 3-5 years after diagnosis.

3. Glycemic control: Poor blood sugar control accelerates the risk of developing DR, even in younger patients.

4. Other factors: High blood pressure, high cholesterol, and puberty can increase the risk in teenagers.

Screening recommendations typically suggest:

For type 1 diabetes: Start retinal screening 3-5 years after diagnosis and once the child is around 9-10 years old.

For type 2 diabetes: Screening should begin at diagnosis, as type 2 diabetes can often go undetected for years, and retinopathy may already be present.

Early detection is crucial because diabetic retinopathy in young people is often asymptomatic initially, but timely management can prevent vision loss.

**model: Copilot**

*answer:*

Yes - children and teenagers with diabetes can develop diabetic retinopathy, although the risk depends on diabetes type, duration, and blood-sugar control. The condition is less common in early childhood but becomes increasingly likely during puberty and adolescence.

Key takeaway

Any young person with diabetes is at risk for diabetic retinopathy, and the risk rises with longer disease duration, higher HbA1c, puberty, and type 2 diabetes.

What the research shows

1. Children and teens do develop diabetic retinopathy

A large U.S. study of 343 patients under age 21 found 3.8% had diabetic retinopathy.

3.4% in type 1 diabetes

6% in type 2 diabetes

Another 50-year population study showed that youth with type 2 diabetes develop retinopathy earlier and at higher rates than those with type 1.

2. Puberty increases risk

Hormonal changes and typically worse glycemic control during puberty make retinopathy more likely to appear in the teen years.

3. Poor blood-sugar control is the strongest predictor

In the Johns Hopkins study:

No child with HbA1c < 8% developed retinopathy

Higher HbA1c and higher systolic blood pressure increased risk

4. Type 2 diabetes in youth is especially concerning

Young people with type 2 diabetes:

Developed retinopathy after a shorter duration of disease

Had higher prevalence (up to 52.7% in long-term follow-up)

Screening recommendations for young people

According to the American Diabetes Association (summarized in Stanford Children's Health):

Type 1 diabetes: first dilated eye exam 3-5 years after diagnosis, then every 1-2 years

Type 2 diabetes: first exam at diagnosis, then every 1-2 years

More frequent exams if retinopathy is already present or if blood-sugar control is poor

What this means for families

If you're caring for a child or teen with diabetes, the most important steps to reduce risk are:

Maintain good blood-sugar control (HbA1c < 7-8%)

Annual dilated eye exams

Manage blood pressure

Avoid smoking exposure

**model: DeepSeek**

*answer:*

Yes, children and teenagers with diabetes can develop diabetic retinopathy, although it is less common than in adults. The risk increases with longer duration of diabetes, poor blood sugar control, and the presence of other factors like high blood pressure. Both type 1 and type 2 diabetes in young people can lead to retinal damage over time. For this reason, major medical guidelines recommend regular eye exams starting approximately 5 years after diagnosis for those with type 1 diabetes, and at the time of diagnosis for those with type 2 diabetes, with follow-ups annually or as advised by an eye care professional. Early detection and good diabetes management are key to preventing vision loss.

**model: Doubao**

*answer:*

Main high-risk groupType 1 diabetes (the most common form in children/teens) is the primary cause. DR rarely develops within the first 5 years of Type 1 diabetes diagnosis or before puberty. The risk rises sharply after 5-10 years of diabetes duration, especially after adolescence.

Type 2 diabetes in youthWith rising childhood obesity, Type 2 diabetes is increasingly seen in teens. Young people with Type 2 diabetes can develop retinopathy much earlier-sometimes even at the time of diagnosis-due to longer undiagnosed high blood sugar.

Risk factors that speed up onset

Poor long-term blood glucose control

High blood pressure

High cholesterol

Early puberty / longer diabetes duration

Obesity and metabolic syndrome

Screening guidelinesFor children/teens with Type 1 diabetes:First eye screening within 3-5 years of diagnosis, or by age 10-11 (whichever comes first), then annual follow-ups.For Type 2 diabetes in youth:Eye screening at the time of diagnosis, then yearly exams.

Clinical notePediatric diabetic retinopathy may be mild at first (non-proliferative) but can progress to severe proliferative retinopathy and permanent vision loss if unmonitored and poorly managed. Tight blood sugar, blood pressure control and regular eye screening can greatly reduce risk.

**model: Gemini**

*answer:*

Yes, children and teenagers with diabetes can develop diabetic retinopathy (DR), although the risk and prevalence vary significantly based on the type of diabetes, age, and duration of the condition.

Prevalence and Risk Factors

While DR is more common in adults, studies show it is increasingly present in younger populations:

Type 1 Diabetes: Prevalence is generally low in children under 10. However, it rises significantly after the onset of puberty, which is considered a major risk factor due to hormonal changes. Recent data suggests about 12% to 17% of youth with Type 1 may show signs of DR, often after living with the condition for at least 5 years.

Type 2 Diabetes: Children with Type 2 diabetes often develop retinopathy sooner and more aggressively than those with Type 1. Studies have found prevalence rates as high as 9% to 42% in this group, with signs sometimes appearing within 2.5 to 5 years of diagnosis.

Key Drivers: The primary risk factors include poor glycemic control (high HbA1c), high blood pressure (hypertension), and the total duration of the disease.

Screening Guidelines

Because DR is often asymptomatic in its early stages, regular screening is vital for prevention:

Timing for Type 1: Screening typically begins 5 years after diagnosis or at age 11-12 (whichever comes first).

Timing for Type 2: Because the exact onset of Type 2 is often unknown, screening is recommended at the time of diagnosis and annually thereafter.

Method: A comprehensive dilated eye exam performed by an ophthalmologist or optometrist is necessary, as standard vision tests (like those at school) cannot detect retinal damage.

Prevention and Management

The progression of retinopathy can be significantly slowed or even prevented through:

Intensive Glycemic Control: Maintaining HbA1c levels within target ranges (often $\leq 7.0\%$).

Blood Pressure Management: Keeping blood pressure stable to reduce stress on retinal capillaries.

Early Intervention: If caught early, treatments like laser therapy or specific medications can prevent severe vision loss.

Since the retina is often one of the first targets of diabetic complications in youth, consistent monitoring and early management of blood sugar levels remain the most effective tools for protecting long-term vision.

**model: Perplexity**

*answer:*

Yes. Children and teenagers with diabetes can develop diabetic retinopathy, although it is less common early on and the risk rises with longer diabetes duration, poorer blood sugar control, and puberty. Youth with type 2 diabetes may develop it sooner and at higher rates than youth with type 1 diabetes.

What that means

Diabetic retinopathy is damage to the retina's blood vessels caused by diabetes, and it can occur in anyone with type 1 or type 2 diabetes. In children, it may appear after several years of diabetes, so eye screening is still important even when they feel fine.

Screening timing

For children with type 1 diabetes, screening is commonly recommended after about 5 years of diabetes, with earlier attention in puberty or when type 2 diabetes is present. Pediatric studies also show that some adolescents already have retinopathy by age 12 or 13, especially with longer diabetes duration.

Why early care matters

Early retinopathy often has no symptoms, so regular dilated eye exams are the best way to catch it before vision is affected. Good glucose control helps lower risk

**Q6. Can I have diabetic retinopathy even if my vision seems normal?**

**model: ChatGPT**

*answer:*

Yes, you can have diabetic retinopathy even if your vision seems normal. In the early stages of diabetic retinopathy, damage to the blood vessels in the retina can occur without noticeable changes in vision. This is why diabetic retinopathy often goes undetected until it becomes more advanced.

Routine eye exams, especially dilated retinal exams, are crucial for people with diabetes because they allow early detection and management before vision loss occurs. Early treatment can prevent or slow progression, even if you don't notice vision problems yet.

If you want, I can explain the different stages of diabetic retinopathy and how vision can remain normal while the disease progresses.

**model: Copilot**

*answer:*

Yes - you absolutely can have diabetic retinopathy even if your vision seems completely normal. In fact, the early stages almost always develop silently, with no pain and no noticeable vision changes. This is one of the reasons eye doctors emphasize routine dilated eye exams for anyone with diabetes.

Why vision can seem normal even when retinopathy is present

Early diabetic retinopathy has no symptoms. Multiple medical sources confirm that the earliest changes in the retina often cause no vision problems at all.

Damage begins with tiny blood vessel changes in the retina - swelling, leakage, or closure - but these don't immediately affect the parts of the retina responsible for sharp vision.

Vision changes typically appear only when the disease progresses, such as when:

bleeding occurs into the vitreous,

swelling affects the macula (macular edema),

or abnormal new blood vessels begin to grow.

What this means for you

Even if your sight feels perfect, diabetic retinopathy can still be developing quietly. That's why medical guidelines recommend:

A yearly dilated eye exam for anyone with diabetes - even with normal vision.

More frequent exams if your doctor has already seen early changes.

A dilated exam or imaging test (like OCT or fluorescein angiography) can detect early vessel damage long before symptoms appear.

When to seek care sooner

If you ever notice:

new floaters

blurry or fluctuating vision

dark or empty spots

faded colors

sudden vision loss

you should contact an eye doctor promptly, as these can be signs of more advanced disease.

**model: DeepSeek**

*answer:*

Yes, it is absolutely possible to have diabetic retinopathy even if your vision seems completely normal. In fact, early and even moderate stages of diabetic retinopathy often cause no noticeable vision changes. This is because the disease initially affects the small blood vessels in the retina before the macula (the part responsible for sharp, central vision) is involved. You may have bleeding, swelling, or new abnormal vessel growth without a drop in visual acuity. That's why regular dilated eye exams are essential for anyone with diabetes-detecting retinopathy early allows for treatment that can prevent vision loss later. Waiting until you notice symptoms often means the disease is advanced and more difficult to treat.

**model: Doubao**

*answer:*

Absolutely yes - you can have diabetic retinopathy (DR) with completely normal vision, especially in the early stages.

Key reasons

Early DR is silentMild non-proliferative diabetic retinopathy only causes tiny blood vessel leaks, microaneurysms, or small hemorrhages in the retina. These changes happen in peripheral or non-central retina and do not affect sharp central vision at all.

Macula involvement comes laterVision loss only occurs when the macula (central vision area) develops edema, ischemia, or when advanced proliferative DR causes vitreous hemorrhage or retinal detachment. Early stages spare the macula, so your eyesight feels perfect.

No obvious symptoms at firstEarly DR usually has no warning signs: no blurriness, no floaters, no pain. By the time you notice vision changes, the damage is often already moderate or severe.

Clinical guideline takeaway

All people with diabetes need regular retinal screening (dilated eye exam) even with normal vision:

Type 1: screen within 5 years of diagnosis

Type 2: screen immediately at diagnosis

Annual follow-up routinely

**model: Gemini**

*answer:*

Yes, you can absolutely have diabetic retinopathy even if your vision feels perfectly normal. In fact, this is one of the most common ways the condition behaves in its early stages.

Because the retina is a large area of tissue, damage to the small blood vessels can occur on the periphery without affecting your central "20/20" vision. Here is a breakdown of why it often goes unnoticed:

The "Silent" Stages

Stage 1 & 2 (Non-proliferative): At these early stages, tiny bulges (microaneurysms) may form in the blood vessels. They can leak small amounts of fluid or blood, but if this doesn't happen in the macula (the part of the eye responsible for sharp, central vision), you won't notice any blurriness or change in how you see.

The Brain's Compensation: Your brain is remarkably good at "filling in the gaps." If you have small spots or damaged areas in one part of your retina, your other eye or the healthy parts of that same eye often compensate, making it impossible for you to detect the problem yourself.

Why It's Treacherous

By the time you actually notice symptoms-like floaters, blurred vision, or dark spots-the disease has often progressed to a more advanced stage.

Vitreous Hemorrhage: Sometimes the first symptom a person notices is a sudden "cloud" of floaters, which happens when a weak blood vessel finally leaks into the center of the eye.

Macular Edema: If fluid leaks into the center of the retina, it causes swelling that leads to blurriness. This can happen quite suddenly or very gradually.

The Importance of Screening

Because you cannot "feel" or "see" the early damage, the only way to catch it is through a dilated eye exam or retinal imaging. An eye specialist can see the physical changes to your blood vessels years before they actually impact your sight.

Standard guidelines generally recommend:

Annual Exams: Even if you have "perfect" vision, a comprehensive eye exam once a year is the gold standard for anyone with diabetes.

Early Detection: If caught early, lifestyle changes and treatments (like laser therapy or injections) can prevent the condition from ever reaching the stage where it threatens your vision.

**model: Perplexity**

*answer:*

Yes - diabetic retinopathy can be present even when your vision seems normal. Early stages often cause no symptoms, and some people can even have more advanced retinopathy or macular edema while still seeing fine, which is why regular dilated eye exams matter.

Why symptoms may be absent

Diabetic retinopathy starts with damage to tiny blood vessels in the retina, and that damage can progress before it affects everyday vision. Vision changes often show up later, such as blur, floaters, dark spots, or trouble seeing at night.

What to do

If you have diabetes, keep up with routine eye screening even if your sight feels normal. Good blood sugar, blood pressure, and cholesterol control also lowers risk and can slow progression.

When to act sooner

Seek prompt eye care if you notice new floaters, flashes, blurry vision, dark areas, or sudden vision loss, because those can signal more advanced disease

**Q7. What increases my risk of diabetic retinopathy?**

**model: ChatGPT**

*answer:*

Several factors can increase your risk of developing diabetic retinopathy. Based on the latest guidance and public consultation question sets on this topic, key risk factors include:

1. Poor blood sugar control - High or fluctuating blood glucose levels are the primary driver of diabetic retinopathy. Maintaining stable, well-controlled blood sugar significantly reduces risk.

2. High blood pressure (hypertension) - Elevated blood pressure can worsen damage to the retinal blood vessels and accelerate disease progression.

3. High cholesterol - Elevated lipid levels contribute to vascular damage in the retina and can exacerbate retinopathy.

4. Smoking - Smoking is an independent lifestyle-related risk factor that increases the likelihood of eye complications in diabetes.

5. Duration of diabetes - The longer you have diabetes, the greater your risk, though this is often included as part of the broader risk profile rather than as an isolated factor.

6. Rapid changes in blood sugar - Very fast improvement in blood sugar can sometimes temporarily worsen retinopathy, particularly in those with pre-existing advanced disease.

Additional factors can include kidney disease, pregnancy, or other systemic health conditions, but the above are the most actionable for prevention and monitoring.

Maintaining good glycemic control, managing blood pressure and cholesterol, avoiding smoking, and attending regular eye exams are the most effective ways to reduce your risk of diabetic retinopathy

**model: Copilot**

*answer:*

The biggest drivers of diabetic retinopathy risk are long-term high blood sugar, high blood pressure, and high cholesterol. Research shows that the longer you've had diabetes and the less controlled these factors are, the more likely retinopathy becomes.

Below is a clear, structured breakdown of the major risk factors, why they matter, and what they mean for you.

Major Risk Factors for Diabetic Retinopathy

Long duration of diabetes - Risk rises sharply after 10-20 years. Nearly all people with type 1 diabetes and over half with type 2 show some retinopathy after 20 years.

Poor blood sugar control - Chronic hyperglycemia damages retinal blood vessels. High HbA1c strongly correlates with retinopathy progression.

High blood pressure - Hypertension accelerates retinal vessel damage and increases severity.

High cholesterol - Elevated lipids contribute to retinal exudates and macular edema.

Pregnancy - Retinopathy can worsen more quickly during pregnancy.

Smoking - Tobacco use increases vascular damage and raises risk.

Ethnicity - Higher risk is seen in Black, Latino, Native American, and Alaska Native populations.

Why These Factors Matter

High glucose injures retinal capillaries to leakage, swelling, ischemia.

High blood pressure increases mechanical stress on fragile vessels.

High cholesterol contributes to fatty deposits (hard exudates) in the retina.

Smoking worsens inflammation and reduces oxygen delivery.

Pregnancy temporarily increases metabolic demands and vascular changes.

These mechanisms combine to increase the likelihood of both non-proliferative and proliferative diabetic retinopathy.

Quick Reference Table

Risk Factor; How It Increases Risk; Most Important Action

High blood sugar; Damages retinal vessels; Keep glucose in target range (with your clinician's guidance)

High blood pressure; Speeds vessel damage; Manage BP consistently

High cholesterol; Causes retinal deposits; Improve lipid levels

Smoking; Reduces oxygen, increases inflammation; Quit smoking

Pregnancy; Speeds progression; More frequent eye exams

Long diabetes duration; Cumulative damage; Annual dilated eye exams

What You Can Do to Lower Risk

Even if you have several risk factors, good management dramatically reduces the chance of vision loss. Studies show that lowering HbA1c by just 1% reduces microvascular complications by 37%.

Key steps include:

Annual dilated eye exams (or more often if recommended)

Consistent glucose, BP, and cholesterol management

Stopping smoking

Staying physically active

If you notice sudden blurriness, floaters, flashes, or dark spots, contact an eye doctor promptly.

**model: DeepSeek**

*answer:*

Several key factors increase the risk of developing diabetic retinopathy, a diabetes complication that damages blood vessels in the retina. The most significant risks include:

Poor blood sugar (glucose) control - Chronically high blood sugar levels are the primary driver.

Long duration of diabetes - The longer you have diabetes (especially 10+ years), the higher the risk.

High blood pressure (hypertension) - Uncontrolled hypertension worsens retinal blood vessel damage.

High cholesterol or blood lipids - Elevated LDL or triglycerides can contribute to retinal vessel damage.

Pregnancy (in women with diabetes) - Pregnancy can accelerate retinopathy, particularly if glucose control is suboptimal.

Kidney disease (diabetic nephropathy) - Often coexists with and predicts retinopathy.

Anemia - Reduces oxygen delivery to the retina, increasing vulnerability.

Genetics/family history - Some individuals may have genetic susceptibility.

Certain ethnicities - Higher risk has been noted in some populations (e.g., Hispanic, African American, Native American, South Asian).

Important note: Early retinopathy often has no symptoms. Regular comprehensive eye exams (at least annually, or more often if advised) are essential for early detection and treatment, which can help prevent vision loss.

If you have diabetes, controlling your blood sugar, blood pressure, and cholesterol through medication, diet, and exercise is the most effective way to lower your risk.

**model: Doubao**

*answer:*

1. Duration of Diabetes

The longer you have type 1 or type 2 diabetes, the higher your risk. Retinopathy is very common after 10+ years of diabetes.

2. Poor Long-Term Blood Glucose Control

Elevated HbA1c and persistent high blood sugar damage tiny retinal blood vessels over time-the strongest modifiable risk factor.

3. Uncontrolled High Blood Pressure

Hypertension worsens retinal microvascular damage and accelerates retinopathy progression, increases risk of macular edema.

4. Dyslipidemia (High Cholesterol / Triglycerides)

Abnormal blood lipids raise the risk of macular edema and severe proliferative diabetic retinopathy.

5. Uncontrolled Kidney Disease

Diabetic nephropathy and retinopathy share microvascular damage mechanisms; kidney disease strongly raises retinopathy risk.

6. Pregnancy

Pregnancy can rapidly worsen existing diabetic retinopathy, especially with poor pre-pregnancy glucose control.

7. Lifestyle & Demographic Factors

Smoking: significantly accelerates retinal vascular damage

Obesity / metabolic syndrome

Lack of regular eye screening

Older age at diabetes diagnosis

8. Other Conditions

Anemia

Sleep apnea

History of cardiovascular disease

**model: Gemini**

*answer:*

The risk of developing or worsening diabetic retinopathy (DR) is influenced by several factors, which are generally categorized into non-modifiable traits and modifiable lifestyle or medical markers.

Primary Risk Factors

Duration of Diabetes: This is often cited as the strongest predictor. The longer you have lived with diabetes, the higher the cumulative risk of retinal damage.

Glycemic Control ($HbA1c$): Consistently high blood sugar levels damage the small blood vessels in the retina. Maintaining a stable hemoglobin A1C level is the primary way to reduce this risk.

Hypertension (High Blood Pressure): Excessive pressure can further strain and damage the delicate retinal capillaries, leading to leaks or swelling (edema).

Hyperlipidemia (High Cholesterol): Elevated levels of fats in the blood, particularly triglycerides, are associated with an increased risk of retinal exudates (fatty deposits in the retina).

Other Contributing Factors

Factor; Description

Pregnancy; For those with pre-existing diabetes, pregnancy can sometimes cause rapid progression of retinopathy due to hormonal and vascular changes.

Kidney Disease; Diabetic nephropathy (kidney damage) is often closely linked with retinopathy, as both involve damage to small blood vessels (microvascular complications).

Ethnicity; Studies indicate higher prevalence rates among Black, Latino, and Native American populations.

Smoking; Tobacco use contributes to systemic vascular damage, which can exacerbate eye complications.

Management and Prevention

While some factors like the duration of your condition cannot be changed, several strategies can significantly slow or prevent progression:

Regular Screenings: Annual dilated eye exams can detect "silent" early stages before vision is affected.

Blood Pressure Management: Aiming for stable readings (typically below 140/80 mmHg, though targets vary by individual) helps protect retinal integrity.

Lifestyle Adjustments: Integrating 150 minutes of moderate aerobic activity per week and a diet rich in fiber and healthy fats (like Omega-3s) supports better metabolic health.

**model: Perplexity**

*answer:*

Several things can increase your risk of diabetic retinopathy: having diabetes for a long time, poorly controlled blood sugar, high blood pressure, high cholesterol, pregnancy, and tobacco use. Risk is also higher in Black, Latino, and Native American people with diabetes.

What matters most

The biggest drivers are usually how long you've had diabetes and how well your blood sugar and blood pressure are controlled. High cholesterol can also contribute, and risk goes up if diabetes is already causing complications in other organs.

Practical takeaway

If you have diabetes, the best ways to lower risk are to keep your glucose, blood pressure, and cholesterol in target range, avoid tobacco, and get regular dilated eye exams

**Q8. Does high blood pressure make diabetic retinopathy worse?**

**model: ChatGPT**

*answer:*

Yes, high blood pressure can make diabetic retinopathy worse. Here's why:

1. Blood Vessel Damage: Both diabetes and high blood pressure can damage small blood vessels in the retina. High blood pressure increases the pressure on already weakened retinal vessels, accelerating damage.

2. Progression of Retinopathy: Studies show that patients with diabetes who also have hypertension are more likely to develop severe forms of diabetic retinopathy, including proliferative retinopathy and macular edema.

3. Vision Risk: Poorly controlled blood pressure increases the risk of vision loss in diabetic patients because it promotes leakage, hemorrhages, and abnormal blood vessel growth in the retina.

4. Management Implication: Controlling blood pressure is a key part of preventing the progression of diabetic retinopathy. Guidelines recommend maintaining blood pressure within target ranges to reduce retinal complications.

In short, high blood pressure acts as a compounding factor in diabetic retinopathy, worsening vascular stress and retinal damage.

**model: Copilot**

*answer:*

Yes - high blood pressure does make diabetic retinopathy worse. Elevated blood pressure increases the stress on retinal blood vessels already weakened by diabetes, accelerating damage and raising the risk of complications such as macular edema, proliferative diabetic retinopathy, and vision loss.

Why high blood pressure worsens diabetic retinopathy

Retinal vessel damage increases with hypertension. High blood pressure narrows, thickens, and stresses retinal vessels, making them more likely to leak or bleed - the same mechanisms that drive diabetic retinopathy progression.

Risk rises even at moderately elevated BP. A large population study of 152,844 diabetic patients found that blood pressure above 120/80 mmHg increased the prevalence of diabetic retinopathy by 10-20%, regardless of whether the patient had diagnosed hypertension.

Hypertension and diabetes amplify each other's damage. Both conditions independently injure retinal microvasculature; together, they accelerate leakage, ischemia, and abnormal vessel growth.

What this means for someone with diabetes

Faster progression: High blood pressure increases the likelihood of moving from mild NPDR to severe NPDR or PDR.

Higher risk of macular edema: Hypertension increases fluid leakage into the macula, worsening central vision.

Greater chance of vision-threatening complications: Including vitreous hemorrhage, retinal detachment, and neovascular glaucoma.

What helps slow or prevent worsening

Tight blood pressure control (often targeting <130/80 mmHg unless your clinician advises otherwise) is strongly associated with reduced retinopathy progression.

Good blood sugar control remains the most important factor, but BP control is a close second.

Regular dilated eye exams allow early detection and treatment before vision loss occurs.

**model: DeepSeek**

*answer:*

Yes, high blood pressure (hypertension) can make diabetic retinopathy worse.

Hypertension damages the small blood vessels in the retina by increasing pressure and mechanical stress, leading to leakage, thickening of vessel walls, and reduced blood flow. In someone with diabetes, who already has compromised retinal vessels due to high blood sugar, this additional damage accelerates the progression of retinopathy. Specifically, hypertension increases the risk of:

Hard exudates and macular edema (swelling that blurs central vision)

Vitreous hemorrhage (bleeding from fragile new vessels)

Ischemia (poor blood flow causing vision loss)

Studies show that strict blood pressure control (typically aiming for <130/80 mmHg or as recommended by a physician) significantly slows the progression of diabetic retinopathy and reduces vision loss. Managing both conditions together-through glucose control, blood pressure medications (e.g., ACE inhibitors or ARBs), and regular eye exams-is essential.

**model: Doubao**

*answer:*

Yes, high blood pressure definitely worsens diabetic retinopathy and accelerates its progression.

Key reasons

Damages tiny retinal blood vesselsHigh BP increases pressure on the fragile capillaries in the retina, causing them to leak fluid, bleed, or become blocked-core changes in diabetic retinopathy.

Speeds up all stagesIt makes mild non-proliferative retinopathy advance faster to macular edema and proliferative diabetic retinopathy, raising risks of vision loss and blindness.

Worsens macular edemaHypertension increases retinal fluid accumulation, directly blurring central vision.

Higher risk of complicationsPoorly controlled BP raises the chance of retinal hemorrhage, retinal detachment, and failed response to laser or anti-VEGF treatment.

Clinical guideline takeaway

Strict blood pressure control (typically target <130/80 mmHg for diabetic patients) is a critical part of preventing and slowing diabetic retinopathy, alongside blood sugar and lipid management.

**model: Gemini**

*answer:*

Yes, high blood pressure (hypertension) is a significant risk factor that can both accelerate the development and worsen the progression of diabetic retinopathy.

The relationship between the two is often described as a "dual hit" to the retinal blood vessels. While diabetes damages the vessels through high blood sugar, hypertension adds mechanical stress, leading to a faster breakdown of the blood-retinal barrier.

Why Hypertension Worsens Retinopathy

Mechanical Stress: High pressure forces blood through already weakened capillaries, causing them to leak fluid or blood into the retina more easily.

Endothelial Damage: Chronic high pressure damages the lining of the blood vessels (endothelium), which can trigger the formation of microaneurysms and "cotton wool spots" (areas of retinal ischemia).

Macular Edema: Hypertension is strongly associated with an increased risk of diabetic macular edema (swelling in the center of the retina), which is a leading cause of vision loss in diabetic patients.

Vascular Endothelial Growth Factor (VEGF): High blood pressure can increase the expression of VEGF, a protein that promotes the growth of abnormal, leaky new blood vessels, potentially leading to the more advanced proliferative stage of the disease.

Clinical Impact of Control

Large-scale clinical trials (such as the UKPDS) have demonstrated that tight blood pressure control is just as critical as blood sugar management for eye health:

Risk Reduction: Research suggests that for every 10 mmHg reduction in systolic blood pressure, the risk of diabetic retinopathy complications can decrease by roughly 10%.

Targets: Most clinical guidelines (including those from the American Diabetes Association) generally recommend a blood pressure target of less than 130/80 mmHg for patients with diabetes and retinopathy.

Comparison Table: Overlapping Effects

Feature; Diabetic Retinopathy Effect; Hypertensive Contribution

Vessel Integrity; Weakened by high glucose.; Ruptured or leaked by high pressure.

Oxygen Supply; Reduced due to vessel blockage.; Further reduced by vessel narrowing (vasospasm).

Swelling; Fluid leakage into the macula.; Increased hydrostatic pressure forces more fluid out.

New Vessel Growth; Triggered by retinal "starvation."; Accelerated by increased VEGF expression.

Management Strategy

Managing this "silent" partnership typically involves:

ACE Inhibitors or ARBs: These specific classes of blood pressure medications are often preferred because they may provide protective effects for the retinal and renal (kidney) microvasculature.

Regular Monitoring: Because both conditions can progress without early symptoms, frequent dilated eye exams are essential to catch changes before vision is permanently affected.

**model: Perplexity**

*answer:*

Yes. High blood pressure can worsen diabetic retinopathy and is linked to a higher risk of developing it, along with more severe forms like proliferative diabetic retinopathy and diabetic macular edema.

Why it matters

Blood vessels in the retina are already vulnerable in diabetes, and high blood pressure adds extra stress that can damage them further. Studies also show that better blood pressure control is associated with less retinopathy progression and less need for retinal treatment.

Practical takeaway

If you have diabetes, keeping blood pressure under good control is one of the ways to help protect your vision, alongside blood sugar control and regular eye exams. Many sources point to a target around or below 120/80 mmHg for reducing retinopathy risk, though the ideal target can vary by person and overall cardiovascular risk.

When to act

If you already have diabetic retinopathy, blood pressure control becomes even more important because hypertension can make eye disease progress faster and raise the chance of vision-threatening complications.

**Q9. Does high cholesterol affect diabetic retinopathy?**

**model: ChatGPT**

*answer:*

Yes - high cholesterol (especially certain lipid abnormalities) can be related to diabetic retinopathy (DR), although the relationship is complex and isn't as clear-cut as with blood sugar or blood pressure. Here's what current evidence suggests:

How cholesterol links with diabetic retinopathy

1. Chronic high cholesterol and hard exudates

Elevated total cholesterol and LDL ("bad") cholesterol have been associated with more retinal hard exudates (fatty deposits in the retina), which are a hallmark of diabetic retinal damage and diabetic macular edema (a common cause of vision loss).

2. Some studies show higher LDL with more severe DR

Older and some recent research found that higher LDL cholesterol levels correlate with more severe diabetic retinopathy, particularly in proliferative stages.

3. HDL ("good") cholesterol findings are inconsistent

Some studies have surprisingly found that higher HDL sometimes correlates with DR, and other research shows complex nonlinear relationships.

A large U.S. cohort study suggested higher HDL had an inverse or neutral association with new DR diagnosis but associations varied depending on circumstances.

4. Cholesterol may influence specific outcomes

Elevated LDL is more consistently linked with diabetic macular edema and progression of retinopathy after it's already present.

5. Biological mechanisms

Diabetes and dyslipidemia can cause lipid deposition and inflammation in retinal vessels, contributing to vessel leakage and retinal damage. Some research even finds cholesterol can crystalize within the retina, potentially contributing to DR pathology.

What this means clinically

High cholesterol itself is not the primary cause of diabetic retinopathy - that role is dominated by poorly controlled blood sugar, duration of diabetes, and hypertension.

However, dyslipidemia can worsen certain aspects of DR (especially hard exudates and macular edema) and may be considered a modifiable risk factor in people with diabetes.

Managing risk

For most people with diabetes, managing cholesterol - particularly LDL cholesterol - is part of comprehensive care. This includes lifestyle changes (diet, exercise) and, when needed, medications such as statins. Good lipid control may help reduce vascular inflammation and indirectly benefit the eyes, though the direct impact on DR progression varies depending on specific lipid profiles and individual risk factors.

**model: Copilot**

*answer:*

High cholesterol does affect diabetic retinopathy - it increases both the risk of developing it and the severity of existing disease. The strongest evidence shows that elevated lipid levels contribute to retinal blood-vessel damage and worsen leakage, swelling, and progression toward vision-threatening stages.

How high cholesterol influences diabetic retinopathy

High cholesterol - High lipid levels promote fatty deposits in retinal vessels, reducing blood flow and increasing vascular damage. This accelerates the development of diabetic retinopathy.

Retinal vessel blockage - Cholesterol-related deposits can obstruct small retinal vessels, worsening ischemia and triggering abnormal new vessel growth.

Macular edema risk - High cholesterol increases the likelihood of diabetic macular edema, a major cause of vision loss in diabetes.

Faster progression - Clinical guidelines emphasize that controlling lipids slows retinopathy progression, alongside glucose and blood pressure control.

Why this matters if you have diabetes

High cholesterol adds to the damage already caused by high blood sugar.

It increases the chance of leaky vessels, exudates, and macular swelling - all of which impair vision.

Managing cholesterol is as important as managing glucose and blood pressure for protecting your eyes.

What helps reduce the risk

Lipid-lowering treatment - Medications like statins or fenofibrate are being studied for protective effects on diabetic retinopathy.

Regular eye exams - Early detection prevents vision loss.

Tight glucose control - Still the strongest protective factor.

Blood pressure management

**model: DeepSeek**

*answer:*

This is an excellent question, as it gets at a crucial intersection of two common metabolic conditions.

The short answer is yes, high cholesterol (specifically high LDL and triglycerides) significantly increases the risk and progression of diabetic retinopathy.

While high blood sugar (hyperglycemia) is the primary driver of diabetic retinopathy, high cholesterol acts as a powerful secondary accelerator. It doesn't usually start the disease, but it makes it much worse, much faster.

Here's a detailed breakdown of how they interact.

The Connection: How Cholesterol Makes Retinopathy Worse

Diabetic retinopathy damages the tiny blood vessels in the retina (the light-sensitive tissue at the back of your eye). High cholesterol contributes to this damage in several key ways:

Hard Exudates: This is the most visible link. When blood vessels are leaky from high blood sugar, cholesterol and other fats can seep out and deposit into the retinal tissue. These deposits look like small, waxy, yellow patches called hard exudates.

Clinical Significance: When hard exudates form in the macula (the central part of the retina responsible for sharp, detailed vision), they cause Diabetic Macular Edema (DME). DME is a leading cause of vision loss in people with diabetes.

Increasing Ischemia (Lack of Blood Flow): High cholesterol, particularly LDL ("bad") cholesterol, can stick to the walls of retinal blood vessels, causing them to narrow and harden (atherosclerosis). This reduces blood flow, starving the retina of oxygen. This state, called retinal ischemia, signals the eye to grow abnormal, fragile new blood vessels-a condition known as Proliferative Diabetic Retinopathy (PDR), the most advanced stage of the disease.

Fueling Inflammation: Both high blood sugar and high cholesterol create a state of chronic, low-grade inflammation throughout the body, including the eye. This inflammation further damages the blood-retinal barrier, making vessels leakier and accelerating the entire disease process.

What the Research Shows

The link is well-established by major clinical trials:

The DCCT (Diabetes Control and Complications Trial) and EDIC (Epidemiology of Diabetes Interventions and Complications) study found that higher LDL and triglycerides were independent risk factors for the development of hard exudates and vision-threatening retinopathy.

Lowering cholesterol has been shown in some studies to slow the progression of retinopathy and reduce the risk of vision loss, although the effect is not as strong as strict blood sugar control.

Important Distinctions: Which Lipids Matter Most?

High LDL ("Bad") Cholesterol: The primary culprit. Directly linked to hard exudates, atherosclerosis, and inflammation.

High Triglycerides: Also a significant, often overlooked, risk factor. High triglycerides are strongly associated with the development of hard exudates and DME, especially in people with type 2 diabetes.

Low HDL ("Good") Cholesterol: This is a problem because HDL helps remove excess cholesterol. Low levels remove this protective effect, indirectly increasing risk.

The Clinical Takeaway: A Triple-Pronged Strategy

For anyone with diabetes, managing eye health requires managing three things together. You cannot focus on blood sugar alone.

Risk Factor; Goal; Why it matters for the eye

Blood Glucose; HbA1c < 7.0% (or individualized goal); Primary driver. Reduces the initial leakiness and damage to vessels.

LDL Cholesterol; < 70 mg/dL (often < 55 mg/dL for high-risk); Reduces hard exudates, atherosclerosis, and inflammation in retinal vessels.

Blood Pressure; < 130/80 mmHg; Prevents mechanical stress that makes leaky vessels even worse.

What you should do:

Get a Dilated Eye Exam at least once a year (more often if any retinopathy is present). An ophthalmologist can see hard exudates long before you notice vision changes.

Talk to your doctor about your lipid panel (LDL, HDL, triglycerides). If you have any stage of retinopathy, your LDL goal should likely be aggressively low (e.g., < 55-70 mg/dL).

Medications: Statins are the mainstay of cholesterol treatment. While their primary benefit for the eye is lowering lipids, some studies suggest they may also have independent anti-inflammatory effects that could be beneficial.

Lifestyle: A heart-healthy, Mediterranean-style diet (low in saturated fats and refined carbs) and regular exercise help manage all three factors (cholesterol, blood sugar, and blood pressure) simultaneously.

A Nuanced Note: What High Cholesterol Does NOT Do

It's important to be precise: High cholesterol is not the initial trigger for diabetic retinopathy. You can have perfect cholesterol and still develop retinopathy if your blood sugar is poorly controlled. Conversely, lowering cholesterol will not reverse existing, severe retinopathy, but it is a critical step to prevent it from worsening and to save central vision by limiting macular edema.

In summary: Yes, high cholesterol directly worsens diabetic retinopathy, primarily by creating vision-damaging hard exudates and starving the retina of oxygen. Aggressive management of LDL and triglycerides is just as important as controlling blood sugar for preserving your sight.

**model: Doubao**

*answer:*

Yes, high cholesterol significantly affects the onset, progression, and severity of diabetic retinopathy (DR).

Key Mechanisms

Vascular endothelial damageElevated LDL (bad cholesterol) causes lipid deposition and oxidative stress in retinal microvessels, damaging capillary walls, increasing permeability, and triggering microaneurysms-core early lesions of DR.

Exacerbates macular edemaHigh cholesterol promotes inflammatory responses and vascular leakage in the diabetic macular edema (DME) stage, which is the leading cause of vision loss in DR patients.

Accelerates atherosclerosis & retinal ischemiaDyslipidemia worsens systemic and retinal arteriosclerosis, reducing retinal blood perfusion, leading to retinal ischemia, and driving the progression to proliferative diabetic retinopathy (PDR) with neovascularization.

Synergistic harm with hyperglycemiaChronic high blood sugar + high cholesterol creates a synergistic effect on retinal microvascular injury, making DR develop faster and more severe than with poor glycemic control alone.

Clinical Evidence & Guidelines

Clinical studies confirm elevated total cholesterol, LDL, and low HDL are independent risk factors for DR incidence and progression.

Diabetic retinopathy management guidelines strongly recommend routine lipid monitoring and aggressive cholesterol control alongside blood glucose and blood pressure management.

Clinical Implication

Strict lipid control (targeted LDL reduction) can slow DR progression, reduce macular edema risk, and lower the need for laser/anti-VEGF injection treatment.

**model: Gemini**

*answer:*

The short answer is yes. High cholesterol (and dyslipidemia in general) is a significant modifiable risk factor that can accelerate the progression of diabetic retinopathy (DR) and contribute to vision-threatening complications.

While high blood sugar remains the primary driver of DR, researchers have identified several specific ways that elevated lipids impact the eye.

1. Hard Exudates and Macular Edema

The most direct link between high cholesterol and diabetic eye disease is the formation of hard exudates. These are yellowish deposits of lipids and proteins that leak from damaged retinal blood vessels.

Mechanism: High serum lipids increase the "leakiness" of the blood-retinal barrier. When blood vessels become permeable due to diabetes, the excess fats in the bloodstream seep into the retinal tissue.

Outcome: If these deposits accumulate in the macula (the center of the retina responsible for sharp vision), they cause Diabetic Macular Edema (DME), which is a leading cause of moderate vision loss.

2. Microvascular Damage

High cholesterol levels, particularly LDL ("bad" cholesterol) and triglycerides, contribute to endothelial dysfunction.

Vascular Strain: Just as cholesterol clogs arteries in the heart, it can cause structural changes in the tiny capillaries of the eye.

Inflammation: Research suggests that oxidized byproducts of cholesterol (like 7-ketocholesterol) trigger inflammatory pathways in retinal cells, leading to cell death and further damage to the blood vessels.

3. Synergistic Effects

High cholesterol rarely acts alone. When combined with other factors common in diabetic patients, the risk increases exponentially:

Hypertension + Dyslipidemia: Clinical studies from 2024 and 2025 indicate that patients with both high blood pressure and high cholesterol have a threefold higher risk of progressing to severe or proliferative diabetic retinopathy compared to those with well-managed levels.

Comparison of Lipid Types and Their Impact

Lipid Type; Primary Impact on the Eye

LDL Cholesterol; Strongly associated with the presence of hard exudates and Clinically Significant Macular Edema (CSME).

Triglycerides; Often linked to the overall severity of retinopathy and the progression from non-proliferative (NPDR) to proliferative (PDR) stages.

HDL (Good); Higher levels are generally thought to be protective, though the relationship is less statistically consistent than the negative impact of LDL.

Why This Matters for Management

Managing cholesterol isn't just about heart health; it is a key "pillar" of eye preservation.

Systemic Control: Keeping total cholesterol below $5.2 \text{ mmol/L}$ (approx. $200 \text{ mg/dL}$) is a common clinical target to reduce microvascular risk.

Medication: Certain lipid-lowering drugs, such as fenofibrate, have been shown in large clinical trials (like the ACCORD Eye Study) to slow the progression of retinopathy, sometimes even independent of their effect on blood lipid levels.

**model: Perplexity**

*answer:*

Yes - high cholesterol can affect diabetic retinopathy, but the relationship is a bit nuanced. Evidence suggests dyslipidemia is linked to diabetic retinal changes, especially hard exudates and diabetic macular edema, and higher LDL or total cholesterol has been associated with worse retinal findings in several studies.

How it matters

High cholesterol does not appear to be the only driver of diabetic retinopathy; blood sugar control is still the main factor. But lipid abnormalities can add to retinal vessel damage and may increase the chance of lipid leakage in the retina, which contributes to exudates and swelling. Some studies also found that people with preexisting diabetic retinopathy and high LDL had a higher risk of diabetic macular edema.

What the evidence suggests

Research is not perfectly consistent, and different studies have found different patterns for HDL, LDL, and triglycerides. Still, the overall picture is that abnormal lipids are relevant to both the development and progression of diabetic eye disease, and cholesterol-lowering treatment has been studied as a way to slow vision loss in diabetes.

Practical takeaway

If you have diabetes, it is worth treating cholesterol as part of eye protection, alongside glucose and blood pressure control. Good lipid control may not prevent retinopathy on its own, but it can reduce one more source of retinal injury

**Q10. Does smoking increase the risk of diabetic retinopathy?**

**model: ChatGPT**

*answer:*

Yes - smoking is generally considered to increase the risk of diabetic retinopathy and other diabetes-related eye complications, although research findings vary somewhat depending on study design and diabetes type. Here's a clear breakdown of what the evidence shows:

What Major Health Sources Say

Tobacco use is listed as a risk factor for diabetic retinopathy.

Trusted medical authorities like the Mayo Clinic include smoking (tobacco use) among the risk factors that can raise the chance of developing diabetic retinopathy in people with diabetes.

The CDC similarly lists smoking as a risk factor for vision loss from diabetic retinopathy.

Many clinical eye-health resources also emphasize that smoking worsens blood vessel health and oxidative stress - key processes involved in diabetes complications.

What Research Studies Show

Some large database analyses have found that smokers with diabetes are more likely to develop diabetic retinopathy over time compared with nonsmokers. For example, in one cohort study analyzing millions of health records, smoking was associated with a ≈21% higher risk of diabetic retinopathy over 10 years.

However, other studies have shown mixed or even null associations. A long-term cohort from the 1990s found that smoking status was not significantly linked to long-term progression of diabetic retinopathy.

A more recent genetic and population analysis reported surprisingly lower odds of diabetic retinopathy among smokers in some statistical models - though the authors acknowledged the findings were controversial and not a basis to recommend smoking, and that overall evidence has been inconsistent.

A meta-analysis from 2018 found that smoking was associated with higher risk in people with type 1 diabetes but lower risk in many with type 2 diabetes, though public-health experts caution that this does not negate other harms of smoking.

Why the Evidence Isn't Totally Consistent

Different study designs (cross-sectional vs. longitudinal), types of diabetes, and methods of controlling for other risk factors (like blood sugar control, blood pressure, and lipid levels) can influence results. Smoking also affects many health pathways - vascular inflammation, oxidative stress, and blood flow - that are relevant to both diabetes and eye disease, making it tricky to isolate effects. However, the weight of clinical guidance is cautious: smoking is harmful overall and is generally considered to increase risk for diabetic complications, including retinopathy.

Bottom Line

Smoking is widely recognized by health organizations as a risk factor for diabetic retinopathy and other eye diseases linked to diabetes.

Scientific studies show mixed findings - with some suggesting higher risk, others no clear link, and a few finding complex patterns depending on diabetes type - but these do not override the broader harmful effects of smoking on eye and vascular health.

Quitting smoking is beneficial: It reduces overall vascular damage, supports better diabetes control, and lowers the risk of many complications, including those affecting vision.

**model: Copilot**

*answer:*

Short answer: Smoking does not clearly increase the risk of diabetic retinopathy (DR), and recent large studies even suggest smokers may have a slightly lower risk of diabetic macular edema (a vision-threatening form of DR). But this does not mean smoking is protective - smoking worsens diabetes overall and increases many other serious complications.

What the evidence shows

Most earlier research found no consistent association between smoking and diabetic retinopathy. Recent large-scale data adds nuance:

A 2024 matched-cohort study of 138,894 patients with diabetes found that current and former smokers had a lower 6-year risk of diabetic macular edema compared with never-smokers (0.88-0.90% vs. 1.18%).

The same study's hazard ratios showed current smokers had a 26% lower risk of developing macular edema (HR 0.74) and former smokers had a 24% lower risk (HR 0.76).

Earlier studies have been inconsistent, with many showing no association between smoking and DR progression.

Important: Researchers emphasize that this apparent lower risk is likely due to confounding factors (e.g., survival bias, differences in health-care utilization) - not a biological benefit of smoking.

Why smoking is still harmful for people with diabetes

Even if smoking doesn't clearly increase DR risk, it definitely increases:

Risk of developing type 2 diabetes (30-40% higher)

Cardiovascular disease

Stroke

Kidney disease

Peripheral vascular disease

Overall mortality

These conditions indirectly worsen eye health and diabetes control.

Bottom line

Smoking does not appear to increase the risk of diabetic retinopathy in a consistent or clinically meaningful way. But smoking is still one of the most harmful things a person with diabetes can do, and quitting remains one of the strongest ways to protect long-term health.

**model: DeepSeek**

*answer:*

Yes, smoking is associated with an increased risk of developing or worsening diabetic retinopathy (DR). Multiple studies and meta-analyses have found that smoking contributes to oxidative stress, endothelial dysfunction, and chronic inflammation-all of which can damage retinal microvasculature in people with diabetes. However, the relationship is complex: while some large cohort studies show a modest but significant risk increase, others suggest heavy smoking is more clearly linked to progression of DR (e.g., to proliferative diabetic retinopathy or diabetic macular edema). Quitting smoking is generally recommended for overall vascular health, though tight glycemic and blood pressure control remain the primary interventions for preventing DR.

**model: Doubao**

*answer:*

Yes, smoking significantly increases the risk, severity, and progression of diabetic retinopathy (DR).

Key Evidence & Mechanisms

Elevated incidence & progressionSmokers with diabetes have a 2-3 times higher risk of developing DR, and faster progression from mild retinopathy to proliferative diabetic retinopathy (PDR) and macular edema.

Pathological mechanisms

Damages retinal microvasculature, aggravates capillary occlusion and ischemia.

Increases oxidative stress, inflammation, and endothelial dysfunction.

Reduces retinal blood flow and oxygen supply.

Worsens dyslipidemia and hypertension, major comorbidities accelerating DR.

Higher complication riskSmoking raises the risk of vision loss, vitreous hemorrhage, retinal detachment, and poor response to laser/anti-VEGF treatment for DR.

Quitting benefitsSmoking cessation reduces DR progression risk over time and improves long-term visual prognosis in diabetic patients.

**model: Gemini**

*answer:*

Recent clinical research and large-scale studies in 2026 highlight a significant, though complex, relationship between smoking and diabetic retinopathy (DR).

While the data has historically been debated, the latest evidence strongly suggests that smoking increases the risk of developing more severe forms of the disease.

1. Increased Risk and Disease Severity

A major global study involving over 12 million patients published in 2026 found that smokers have a statistically significant increase in the 10-year incidence of diabetic retinopathy compared to non-smokers. Specifically:

Overall Risk: Smokers are approximately 21% more likely to develop DR ($RR = 1.21$).

Disease Progression: The risk is higher as the disease progresses. Smokers show a significantly higher likelihood of moving from mild non-proliferative stages to Proliferative Diabetic Retinopathy (PDR), which is the most vision-threatening stage.

Complications: Cigarette dependence is linked to higher rates of secondary complications, including:

Vitreous hemorrhage

Tractional retinal detachment

Neovascular glaucoma

2. Biological Mechanisms

Smoking exacerbates the primary drivers of retinal damage in several ways:

Oxidative Stress & Inflammation: Tobacco smoke contains over 4,000 toxic compounds that trigger chronic inflammation and oxidative stress, damaging the delicate blood vessels in the retina.

Vascular Dysfunction: Nicotine and carbon monoxide reduce oxygen delivery to retinal tissues (ischemia) and impair the function of the vascular endothelium.

Blood Sugar Control: Smoking impairs insulin activity, making it more difficult for individuals to maintain stable blood glucose levels, which is the cornerstone of managing DR.

3. Variations by Diabetes Type

Interestingly, research indicates that the impact of smoking can vary based on the type of diabetes:

Type 1 Diabetes: There is a consistent and strong positive correlation between smoking and both the onset and progression of DR and PDR.

Type 2 Diabetes: Some studies show more heterogeneous results. While many recent large-scale database analyses confirm an increased risk, some older or specific cross-sectional studies have suggested a "protective" or neutral effect. However, medical experts emphasize that this should not detract from the clear cardiovascular and overall ocular risks smoking poses.

Key Summary Table

Condition; Risk for Smokers (vs. Non-Smokers)

Mild NPDR; 1.37x higher risk

Severe NPDR; 1.98x higher risk

Proliferative DR (PDR); ~1.20x to 1.48x higher risk

Retinal Vascular Occlusion; 1.64x to 2.45x higher risk

[!IMPORTANT]

Smoking cessation remains one of the most effective modifiable factors for protecting eye health. Recent data shows that quitting smoking significantly reduces the hazard ratio for developing first-instance PDR and its related complications.

**model: Perplexity**

*answer:*

Yes-smoking can increase the risk of diabetic retinopathy, especially in type 1 diabetes, and it may also worsen progression of eye disease in people with diabetes. Evidence is mixed across studies, with some older studies finding no clear association, but the overall medical advice still strongly favors smoking cessation because smoking harms blood vessels and worsens diabetes-related complications.

What the evidence shows

A 2018 meta-analysis found smokers with type 1 diabetes had a higher risk of diabetic retinopathy and proliferative diabetic retinopathy, while the association in type 2 diabetes was less consistent across studies. Older studies reported no excess retinopathy risk in smokers, which is part of why the literature looks mixed. Despite that inconsistency, smoking is still considered harmful for diabetic eye health and overall diabetes outcomes.

Why it matters

Smoking can impair blood flow, increase inflammation, and make blood sugar control harder, all of which can contribute to damage in the retina. For someone with diabetes, quitting smoking is one of the best steps to reduce the chance of eye and cardiovascular complications.

Practical takeaway

If you have diabetes, the safest assumption is that smoking raises your risk of eye damage and may speed progression if retinopathy is already present. Regular dilated eye exams and smoking cessation are both important parts of protecting vision

**Q11. Can good blood sugar control prevent diabetic retinopathy?**

**model: ChatGPT**

*answer:*

Yes, good blood sugar control can significantly help prevent or slow the progression of diabetic retinopathy (DR). Research consistently shows that maintaining blood glucose levels close to normal reduces the risk of developing DR and also slows its advancement in people who already have the condition.

Key points:

1. Tight glycemic control: Keeping HbA1c levels within recommended targets (usually <7% for many adults with diabetes) lowers the likelihood of both the onset and progression of DR.

2. Early intervention: The benefits are most pronounced when good blood sugar control is maintained from the early stages of diabetes, even before DR develops.

3. Supporting factors: Blood pressure and lipid management, along with regular eye exams, complement glucose control to further reduce DR risk.

Major landmark studies, like the Diabetes Control and Complications Trial (DCCT) for type 1 diabetes and the UK Prospective Diabetes Study (UKPDS) for type 2 diabetes, demonstrated that intensive glycemic control reduced the risk of developing DR by approximately 25-76%, depending on the study and population.

In short, while good blood sugar control doesn't guarantee complete prevention, it is the single most effective measure to reduce the risk and severity of diabetic retinopathy.

**model: Copilot**

*answer:*

Good blood sugar control significantly lowers the risk of developing diabetic retinopathy and slows its progression, but it does not guarantee complete prevention. Consistently high blood sugar damages the tiny blood vessels in the retina, and keeping glucose levels in a healthy range reduces this damage.

How Blood Sugar Control Helps

Stable glucose levels reduce stress on retinal blood vessels, lowering the chance of leaks, swelling, and abnormal vessel growth - the hallmarks of diabetic retinopathy.

People who maintain good glycemic control show significantly reduced risk of vision problems compared with those with poorly controlled diabetes.

Good control can even improve vision in some cases by reducing retinal swelling.

Why It Doesn't Guarantee Full Prevention

Even with excellent blood sugar control, factors like duration of diabetes, blood pressure, cholesterol, and genetics also influence risk. Over time, more than half of people with diabetes develop some degree of retinopathy.

What Else Protects Your Eyes

Regular dilated eye exams - at least once a year; early detection is key.

Healthy blood pressure and cholesterol control - both reduce retinal vessel damage.

Exercise - at least 150 minutes/week helps stabilize glucose and protect retinal health.

Smoking cessation - smoking increases the risk of diabetic retinopathy.

Bottom Line

Yes - good blood sugar control is one of the strongest tools to prevent or delay diabetic retinopathy, but it works best when combined with regular eye exams, healthy blood pressure, cholesterol management, and lifestyle habits.

**model: DeepSeek**

*answer:*

This is an excellent and important question.

The short answer is yes, absolutely. Good blood sugar control is the single most important modifiable factor in preventing the onset and slowing the progression of diabetic retinopathy.

This conclusion is backed by decades of large-scale clinical trials, most notably the Diabetes Control and Complications Trial (DCCT) for type 1 diabetes and the United Kingdom Prospective Diabetes Study (UKPDS) for type 2 diabetes.

Here's a more detailed breakdown of how it works, what "good control" means, and crucial caveats.

How High Blood Sugar Damages the Retina

Chronically high blood sugar (hyperglycemia) damages the tiny blood vessels in the retina, the light-sensitive tissue at the back of your eye. The damage happens in several ways:

Biochemical Damage: High glucose levels disrupt normal cellular metabolism, creating harmful byproducts (like advanced glycation end-products or AGEs) and causing oxidative stress.

Blood Vessel Weakness: The walls of the retinal capillaries become weak and leaky, leading to macular edema (swelling in the central retina, the macula) and the formation of microaneurysms (tiny bulges in vessel walls).

Clogged Vessels: Damaged cells cause capillaries to close off, depriving the retina of oxygen (hypoxia).

Abnormal New Vessel Growth: In response to widespread hypoxia, the retina releases growth factors (like VEGF) to try and create new blood vessels. However, these proliferative diabetic retinopathy (PDR) vessels are fragile, grow in the wrong places (along the retina and into the vitreous gel), and can easily bleed, leading to severe vision loss and retinal detachment.

The Evidence: What the Studies Show

For Type 1 Diabetes (DCCT): Intensive blood sugar control reduced the risk of developing any diabetic retinopathy by 76% and slowed the progression of existing retinopathy by 54% compared to standard control.

For Type 2 Diabetes (UKPDS): Intensive control reduced the risk of developing retinopathy by 25-35% and reduced the need for laser treatment by 29%.

The benefits are also long-lasting. Follow-up studies (EDIC for type 1, and long-term UKPDS follow-up for type 2) show a "legacy effect" - early years of good control provide protective benefits against retinopathy and other complications for a decade or more, even if blood sugar becomes harder to manage later.

What Does "Good Blood Sugar Control" Actually Mean?

It's not about a single perfect reading, but about maintaining levels within a target range consistently over time. The key metrics are:

HbA1c (Hemoglobin A1c): This is your 2-3 month average blood sugar. The primary goal for most non-pregnant adults is an HbA1c below 7.0% (53 mmol/mol). Someone without diabetes has an A1c around 5%. Studies show that for every 1-point reduction in A1c (e.g., from 8% to 7%), the risk of retinopathy drops by about 35-40%.

Time in Range (TIR): A newer, more dynamic metric. This is the percentage of time your glucose levels stay between 70 and 180 mg/dL (3.9-10.0 mmol/L). A good target is a TIR of >70% (about 17 hours per day) with very little time above 180 mg/dL.

Crucial Caveats and "The Paradox of Rapid Improvement"

While good control is crucial, there are two very important warnings:

Hypoglycemia Risk: The biggest barrier to achieving very low A1c (e.g., <6.0%) is the risk of dangerously low blood sugar (severe hypoglycemia), which can cause seizures, unconsciousness, or death. This is why an A1c of 7.0% is generally the sweet spot - balancing eye protection with safety.

The "Early Worsening" Phenomenon (VERY IMPORTANT): If your blood sugar has been very poor for a long time (e.g., A1c consistently >10%) and you suddenly achieve near-normal control (e.g., A1c drops to 6.5% in a few weeks), diabetic retinopathy can temporarily and paradoxically worsen in the first few months. This is not a reason to avoid good control, but it requires a strategic approach: gradual improvement rather than an abrupt, drastic drop, and very close monitoring by an ophthalmologist. Patients with pre-existing severe retinopathy are most at risk.

It's Not Just About Blood Sugar

To maximize prevention, you must manage other risk factors simultaneously. Think of high blood sugar as the "fuel on the fire," but these other factors are the "wind":

High Blood Pressure (Hypertension): The single most important risk factor for macular edema and hemorrhages. The target is usually <130/80 mmHg.

High Cholesterol (Dyslipidemia): Contributes to blood vessel damage and the formation of "hard exudates" (fatty deposits) in the retina.

Anemia, Sleep Apnea, Kidney Disease: All can worsen retinopathy independent of A1c.

Smoking: Drastically increases oxidative stress and vascular damage.

Pregnancy: Can accelerate retinopathy, requiring very intensive monitoring.

The Essential Action Plan

Work with your endocrinologist/primary care doctor: Set a personalized HbA1c and TIR goal. Aim for gradual, sustained improvement.

Get a Comprehensive Dilated Eye Exam:

At diagnosis for type 2 diabetes (retinopathy can be present at diagnosis).

Within 5 years of diagnosis for type 1 diabetes (after puberty/age 11).

Then at least once a year (or more often if retinopathy is found).

Control your blood pressure and cholesterol with diet, exercise, and medications (ACE inhibitors, ARBs, statins) as prescribed.

Don't let perfect be the enemy of good. Sustained, moderately good control (A1c ~7.5-8.0%) is vastly superior to a "rollercoaster" of very high and very low levels.

The Bottom Line

Yes, good blood sugar control is the most powerful preventive weapon against diabetic retinopathy. It can cut your risk by over 75%. However, it is not a 100% guarantee. Genetics, diabetes duration, and other risk factors play a role. The winning strategy is a holistic one: consistently good (but safely achieved) blood sugar control + optimal blood pressure and cholesterol management + lifelong, annual dilated eye exams.

If you have diabetes, you have tremendous power to protect your vision, starting with your next meal and your next glucose check.

**model: Doubao**

*answer:*

Yes, strict and sustained blood sugar control is the most critical proven measure to prevent and slow diabetic retinopathy (DR).

Key evidence & conclusions

DCCT / UKPDS landmark trialsTight glycemic control significantly reduces the risk of developing diabetic retinopathy in type 1 and type 2 diabetes, and slows progression of existing retinopathy.

MechanismChronic hyperglycemia damages retinal microvasculature via oxidative stress, inflammation, and accumulation of advanced glycation end products (AGEs). Good glycemic control reduces this microvascular injury.

Real limitationEven with optimal blood sugar, long-duration diabetes still carries residual DR risk, so regular retinal screening is still mandatory.

Additional key controls that work together

Stable blood pressure control

Lipid management

Avoid smoking

Regular annual fundus screening

**model: Gemini**

*answer:*

The short answer is yes, but with some important nuances. Consistent blood sugar control is the single most effective way to lower the risk of developing diabetic retinopathy and to slow its progression if it has already started.

How Blood Sugar Impacts Your Eyes

High blood glucose levels over time damage the tiny blood vessels (capillaries) that nourish the retina. When these vessels are damaged, they may leak fluid or bleed, leading to vision loss. By keeping blood sugar within a target range, you reduce the "metabolic stress" on these delicate vessels.

The Evidence for Prevention

Major clinical trials, such as the DCCT (Diabetes Control and Complications Trial) and UKPDS (United Kingdom Prospective Diabetes Study), have shown that intensive blood sugar management significantly reduces the risk of eye complications:

Primary Prevention: In people without retinopathy, tight control can reduce the risk of it developing by roughly 70%.

Secondary Prevention: For those who already have early-stage retinopathy, good control can slow the progression to more severe stages by about 50%.

Important Considerations

While control is vital, it isn't the only factor:

1. Metabolic Memory: Long periods of high blood sugar in the past can sometimes "program" the body for complications later, even if current control is excellent. This is why early intervention is so critical.

2. The "Tightly Controlled" Paradox: In rare cases, if blood sugar is brought down extremely rapidly after years of very high levels, retinopathy can temporarily worsen before it gets better. This should always be managed under a doctor's supervision.

3. Other Factors: Blood sugar doesn't act alone. Blood pressure and cholesterol levels are equally important "pillars" of eye health. High blood pressure, in particular, puts extra physical strain on those same fragile retinal vessels.

Proactive Steps

The A1C Target: For most adults, an A1C goal of 7% or lower is generally recommended to prevent complications, though your specific target should be personalized by your physician.

Annual Exams: Even with perfect blood sugar, diabetic retinopathy can be "silent" in its early stages. A dilated eye exam once a year is the only way to catch it before vision is affected.

**model: Perplexity**

*answer:*

Yes-good blood sugar control can reduce the risk of diabetic retinopathy and help slow its progression, but it does not guarantee prevention.

What the evidence says

Blood glucose control is one of the most important factors for preventing diabetic retinopathy, and the better HbA1c is reduced, the greater the protective effect tends to be. Even so, diabetic retinopathy can still develop in some people despite good control because other factors also matter, including how long someone has had diabetes, blood pressure, cholesterol, smoking, and pregnancy.

Practical takeaway

Keeping blood sugar in target range is a major step for eye protection, but it works best alongside regular dilated eye exams and control of blood pressure and cholesterol. In other words, good glucose control lowers risk, but it is not a complete shield

**Q12. Are there vitamins, supplements, or eye drops that prevent diabetic retinopathy?**

**model: ChatGPT**

*answer:*

Short answer: No vitamin, supplement, or eye drop has been proven to reliably prevent diabetic retinopathy in humans. There's research interest in nutrients and experimental eye drugs, but solid clinical evidence for prevention is lacking, and standard medical care does not include supplements or eye drops specifically to stop diabetic retinopathy from developing.

Here's what the science and clinical guidance currently say:

1. What does cause diabetic retinopathy?

Diabetic retinopathy is a complication of diabetes caused by chronic high blood sugar damaging the tiny blood vessels in the retina. It's the leading cause of vision loss in adults with diabetes.

Because the cause is metabolic (high blood sugar, blood pressure, cholesterol), the most effective prevention strategies are systemic:

Tight glucose (A1C) control

Blood pressure and cholesterol control

Regular dilated eye exams

Early treatment if retinopathy starts

These steps reduce risk far more than any supplement evidence so far.

2. Vitamins & Supplements - What the Research Shows

There are studies looking at nutrients and diabetic retinopathy, but the evidence is weak and mixed:

Vitamin levels and risk:

Some observational studies have found associations between lower levels of certain vitamins (such as vitamin D or B vitamins) and higher rates or severity of retinopathy - but association isn't proof of prevention, and results are inconsistent.

Small small trials:

A few small pilot studies have looked at combinations of B vitamins (like B6, B9/folate, B12) or antioxidant frameworks and seen minor changes in retinal sensitivity or thickness, but these trials are not large enough or long enough to prove that supplements actually prevent disease progression.

Systematic reviews:

Larger reviews show some nutrients might have beneficial biological effects in theory or in lab/animal models, but convincing clinical evidence that supplements prevent diabetic retinopathy in people does not exist yet.

Official guidance:

Government and eye-health organizations (like the U.S. National Center for Complementary and Integrative Health) note that, for diabetic retinopathy specifically, no supplements have conclusive helpful evidence, and people should not delay proven medical care to try unproven supplements.

3. What about Eye Drops?

There are experimental eye drug therapies in clinical research - some target inflammatory and vascular pathways involved in retinopathy - but these are not available as preventive drops for everyday use. Most approved eye medications (like anti-VEGF injections) are used to treat advanced diabetic eye disease, not to prevent it.

A class of eye drops (ripasudil, a Rho kinase inhibitor) has been studied for diabetic retinal conditions but has not become a recommended preventive eye drop.

So at present, there's no clinically proven eye drop that prevents diabetic retinopathy.

4. What can help protect your eyes? (Evidence-based)

While vitamins and supplements aren't proven to prevent retinopathy on their own, a healthy lifestyle does help support your overall eye health:

Keep blood sugar (A1C) in target range

Control blood pressure and cholesterol

Maintain a balanced diet rich in leafy greens, colorful vegetables, and omega-3 fats

Get annual dilated eye exams

Avoid smoking

These actions are linked with lower risk of eye complications from diabetes.

Bottom Line

No supplements, vitamins, or eye drops are proven to prevent diabetic retinopathy in people.

Some nutrients are being studied, but evidence isn't strong enough to recommend them for prevention.

The most reliable way to prevent or delay diabetic eye disease is excellent diabetes control and regular medical eye screening

**model: Copilot**

*answer:*

Short answer: No vitamin, supplement, or eye drop has been proven to prevent diabetic retinopathy. Some nutrients show early promise as supportive (not primary) therapy, but the strongest protection still comes from tight blood sugar control, blood pressure control, cholesterol management, and regular dilated eye exams.

Below is a structured, evidence-based breakdown of what may help, what is unproven, and what to avoid assuming.

What actually prevents diabetic retinopathy

Good diabetes control - Maintaining healthy blood sugar levels is the most effective way to prevent or delay diabetic retinopathy.

Blood pressure & cholesterol control - High BP and cholesterol increase risk. Managing them lowers progression risk.

Annual dilated eye exams - Early detection prevents vision loss.

These remain the foundation. Supplements can only be adjuncts.

Vitamins & supplements with some evidence (supportive, not preventive)

1. Vitamin D

A randomized clinical trial in diabetic macular edema found that correcting vitamin D deficiency may improve response to anti-VEGF injections, but this does not prove prevention of retinopathy.

Best viewed as supportive if you are deficient.

2. Carotenoids (Lutein & Zeaxanthin)

Present in leafy greens; accumulate in the retina.

Lab, animal, and small human studies show antioxidant and anti-inflammatory effects that may reduce oxidative stress and support retinal health.

A small retrospective study showed improved retinal function with supplementation.

Evidence is promising but not definitive.

3. B-vitamins & methylated folate

Some patients with diabetic retinopathy have deficiencies in B-vitamins and elevated homocysteine.

Addressing deficiencies (B1, B2, B6, methylfolate, B12) may reduce oxidative stress and support retinal health.

This is supportive, not a proven preventive therapy.

4. Alpha-lipoic acid and N-acetylcysteine

Antioxidants that may reduce oxidative stress in the retina.

Evidence is mostly mechanistic or early-stage.

5. Curcumin

Anti-inflammatory and antioxidant effects shown in lab and animal studies.

Human evidence is limited.

Supplements with weak or insufficient evidence

Many "diabetic eye health" supplements are based on formulas for age-related macular degeneration, not diabetic retinopathy.

These include vitamins C, E, zinc, copper, lutein, and zeaxanthin - helpful for general retinal health but not proven to prevent diabetic retinopathy.

Eye drops marketed for diabetic retinopathy

No over-the-counter eye drop has been shown to prevent diabetic retinopathy.

Prescription drops (e.g., anti-VEGF) are injections, not drops, and are used to treat existing disease, not prevent it.

What supplements cannot do

Replace anti-VEGF injections, laser therapy, or medical management.

Prevent diabetic retinopathy on their own.

Reverse existing retinal damage.

Practical guidance

If you want to use supplements safely:

Discuss them with your ophthalmologist or endocrinologist.

Check for interactions with diabetes medications.

Focus on correcting documented deficiencies (vitamin D, B-vitamins) rather than taking broad "eye formulas."

**model: DeepSeek**

*answer:*

Based on current medical research, there is no definitive proof that any vitamin, supplement, or eye drop can prevent diabetic retinopathy. However, several promising candidates have shown potential benefits in early studies and are being actively investigated.

The single most effective way to prevent diabetic retinopathy remains strict management of blood sugar, blood pressure, and cholesterol.

Here is a summary of key substances currently under investigation, categorized by their route of administration.

At a Glance: Potential Protective Agents

Category; Substance(s); Key Evidence & Potential Effects; Current Verdict

Oral Supplements; B Vitamins (B1, B6, B9, B12); Associated with improved retinal function and slower disease progression.; Promising, but requires larger trials.

Vitamin D; Low blood levels are strongly linked to higher risk of developing retinopathy.; Associational, need interventional studies.

Vitamin E & Omega-3s; May protect retinal blood vessels and nerve fibers.; Mixed results; ongoing research.

AREDS Formula; Prevented retinopathy in diabetic rats. Contains vitamins C, E, beta-carotene, zinc, copper.; Promising in animals, not yet proven in humans.

Eye Drops; Vitamin B12 & Citicoline; Shown to stabilize retinal structure and function, and reduce fatty deposits.; Early but positive clinical results.

Brimonidine & Somatostatin; Aim to protect retinal nerves. Safety confirmed, but final efficacy results are pending.; Research ongoing; no final data yet.

Oral Supplements: The Most Researched Candidates

Several oral supplements are being studied for their potential role in slowing the progression of diabetic retinopathy, primarily by reducing oxidative stress and inflammation.

B Vitamins (B1, B6, B9, B12): This group has the most consistent positive data. A systematic review suggested they have a "promising preventive role". Vitamin B9 (folate) and B12 help lower homocysteine, an amino acid linked to higher vascular damage risk in diabetes.

One major study found that a high dietary intake of vitamin B6, as well as fruits rich in vitamins C and E, could lower the risk of diabetic retinopathy by up to 50% after eight years.

Vitamin D: Numerous studies have found a significant association between low vitamin D levels in the blood and the development or severity of diabetic retinopathy. While this suggests a protective role, it does not prove that taking supplements will prevent the condition.

Vitamin E and Omega-3 Fatty Acids: As antioxidants, these have shown mixed results. Some studies found vitamin E could normalize retinal blood flow, while others saw no benefit in reducing hemorrhage or edema. An ongoing clinical trial is currently testing whether 1000 mg of omega-3 supplements daily can protect retinal vessels in people with type 1 diabetes.

The AREDS Formula: This combination-high doses of vitamins C and E, beta-carotene, zinc, and copper-is a proven treatment for age-related macular degeneration. In a 12-month study on diabetic rats, this formula significantly prevented the formation of degenerative retinal capillaries. However, this success has not yet been replicated in human trials for diabetic retinopathy.

Eye Drops: A Novel Approach

Eye drops offer a non-invasive way to deliver drugs directly to the retina. Current research focuses on neuroprotection-preserving the retinal nerves that are damaged early in the disease.

Vitamin B12 and Citicoline: This combination is the most promising candidate in this category. A small, three-year trial on patients with early diabetic retinopathy found that those using these eye drops had stabilized retinal structure and function, while the placebo group worsened. A more recent 2025 study reported that citicoline eye drops led to the reduction or complete disappearance of hard exudates (fatty deposits in the retina) in several patients. While promising, this was a small, observational study, and the authors call for confirmation with larger trials.

Brimonidine and Somatostatin: These drugs, which are not vitamins but medications, were tested in the large-scale EUROCONDOR clinical trial to see if they could prevent early diabetic retinopathy. While the safety of the drops was confirmed, the final results on their effectiveness for prevention are still being fully evaluated.

What is Proven to Work: The Foundation of Prevention

While the hunt for an effective supplement continues, the only proven strategies to prevent diabetic retinopathy are well-established and backed by decades of research.

Rigorous Blood Sugar Control: This is the single most important factor. Consistently maintaining blood sugar levels within your target range significantly reduces the risk of developing retinopathy and slows its progression.

Managing Blood Pressure and Cholesterol: Controlling hypertension and dyslipidemia is crucial, as high blood pressure and cholesterol levels directly damage the delicate blood vessels in the retina.

Regular, Comprehensive Eye Exams: An annual dilated eye exam is critical. Diabetic retinopathy often has no early symptoms, but an ophthalmologist can detect the earliest signs years before you notice any vision changes, allowing for timely treatment.

If you are considering any supplement to support your eye health, it is essential to have an open conversation with your ophthalmologist or endocrinologist. They can help you weigh the potential benefits against any risks, such as interactions with your current medications, and ensure these supplements do not distract from the most important goal: maintaining optimal blood sugar control. The evidence is not yet strong enough to recommend any specific supplement as a proven preventive therapy

**model: Doubao**

*answer:*

1. Key Evidence-Based Vitamins & Nutrients

Antioxidant vitamins with protective effects

Vitamin C, Vitamin E: Reduce oxidative stress in retinal blood vessels; slow microvascular damage from chronic hyperglycemia.

B Vitamins (B1, B6, B12, Folate): Lower homocysteine levels (high homocysteine worsens retinal microangiopathy); protect retinal nerve fibers and capillaries.

Specific evidence-backed supplements

Omega-3 fatty acids (DHA/EPA)Strong clinical evidence: Reduce retinal inflammation, improve retinal vascular integrity, lower risk of proliferative DR and macular edema in diabetics.

Lutein & ZeaxanthinBuild retinal macular pigment; reduce oxidative light damage, support retinal cell health (adjunctive prevention for early DR).

ZincEssential for retinal enzyme function and antioxidant defense; helps slow retinal degenerative changes in diabetes.

Alpha-lipoic acid (ALA)Powerful antioxidant; improves insulin sensitivity, reduces oxidative stress in retinal microvessels, aids diabetic neuroprotection.

2. Eye Drops for DR Prevention/Adjunct Use

No over-the-counter eye drops can directly prevent diabetic retinopathy.

Prescription topical drops only serve adjunctive roles:

Anti-VEGF eye drops: Limited role; main anti-VEGF treatment is intravitreal injections (not eye drops).

Artificial tears/anti-inflammatory eye drops: Relieve dry eye common in diabetes; do not reverse or prevent DR microvascular damage.

No nutrient-based eye drops are proven to stop DR progression in large randomized trials.

3. Critical Clarification

Supplements are adjunctive, NOT a replacementNo vitamin/supplement can replace tight blood sugar, blood pressure, lipid control-these are the primary proven prevention for DR.

No supplement cures or reverses established DRThey only help lower risk in pre-diabetes or early diabetes without retinopathy.

Medical guideline takeawayAmerican Academy of Ophthalmology & ADA:Routine supplementation of lutein/zeaxanthin, omega-3, B vitamins is reasonable for diabetic patients to reduce DR risk; avoid high-dose unregulated supplements without physician approval.

4. Best Prevention Strategy Summary

Strict HbA1c, BP, lipid management (first-line)

Annual retinal screening

Adjunct: Omega-3, lutein/zeaxanthin, B-complex, ALA (standard doses)

Eye drops have no preventive effect on DR itself-only symptom relief.

**model: Gemini**

*answer:*

While there are no officially approved "preventative" eye drops or supplements for diabetic retinopathy (DR), several nutritional and pharmaceutical interventions show promise in slowing its progression. Current medical consensus emphasizes that the most effective prevention remains optimal management of blood glucose, blood pressure, and lipids (Sun et al., 2023).

Vitamins and Supplements

Research suggests that oxidative stress and inflammation play key roles in retinal damage. Several supplements have been studied for their potential neuroprotective benefits:

B Vitamins: High doses of Vitamin B1 (Thiamine) may protect vascular endothelium and prevent the activation of pathways that cause retinal damage (Shi et al., 2020). Vitamin B12 and Folate are also noted for their role in lowering homocysteine levels, which is associated with reduced risk of retinal thinning (Milluzzo et al., 2022).

Carotenoids (Lutein and Zeaxanthin): These antioxidants accumulate in the retina and may improve visual performance and protect against capillary degeneration in diabetic patients (Lem et al., 2021).

Antioxidant Cocktails: Studies on combinations of Vitamins C, E, and D, along with alpha-lipoic acid, have shown some ability to improve endothelial function, though large-scale clinical evidence remains less conclusive than for other eye conditions like macular degeneration (Kowluru & Zhong, 2011; Tabatabaei-Malazy et al., 2019).

Fenofibrate: While technically a systemic medication for cholesterol, large-scale trials and expert consensus support the early use of oral fenofibrate to slow the progression of mild to moderate non-proliferative DR (Delphi Consensus, 2026).

Eye Drops and Pharmacological Treatments

Currently, there are no approved eye drops that can prevent the onset of diabetic retinopathy. Most pharmacological treatments are invasive and reserved for advanced stages:

Intraocular Injections: Anti-VEGF agents (like ranibizumab or aflibercept) and steroids are the standard of care for treating diabetic macular edema and proliferative DR, rather than preventing the disease in healthy eyes (Sun et al., 2023).

Emerging Research: Scientific interest is growing in the development of neuroprotective and anti-inflammatory eye drops to target the disease in its earliest stages, but these are largely still in the clinical trial phase (Sun et al., 2023).

**model: Perplexity**

*answer:*

Not really-there is no vitamin, supplement, or eye drop proven to prevent diabetic retinopathy on its own. Some nutrients may be supportive, but the strongest evidence still favors tight blood sugar, blood pressure, and lipid control plus regular eye screening.

What the evidence suggests

A few supplements have shown promising but limited results in small studies, including B vitamins (especially B1, B6, B9, B12), vitamin D, vitamin E, lutein/zeaxanthin, benfotiamine, and alpha-lipoic acid. However, reviews also note that properly designed randomized trials are still needed before any of these can be considered proven prevention.

Eye drops

Eye drops may help with dry eye, irritation, or other symptoms, but they do not fix the retinal blood-vessel damage that causes diabetic retinopathy. A systematic review found some topical treatments may help in selected cases, but the evidence is heterogeneous and not enough to make eye drops a standard preventive treatment for diabetic retinopathy.

Practical approach

The best proven prevention is:

Keep glucose, blood pressure, and cholesterol well controlled.

Get regular dilated eye exams.

Treat eye disease early if it appears.

Supplements can be discussed with your clinician, especially if you have deficiencies, but they should not replace standard diabetes care or eye treatment.

One caution

Some "eye vitamins" are marketed aggressively, but the evidence for routine use to prevent diabetic retinopathy remains weak or incomplete. If you tell me your diabetes type and whether you already have retinopathy, I can narrow this down to the most relevant options.

**Q13. Can traditional or herbal medicines cure diabetic retinopathy?**

**model: ChatGPT**

*answer:*

Currently, there is no strong scientific evidence that traditional or herbal medicines can cure diabetic retinopathy (DR). Diabetic retinopathy is a microvascular complication of diabetes, and its progression is primarily driven by chronic high blood sugar, hypertension, and other metabolic factors.

Here's what is known:

1. Standard treatments: The only proven ways to slow or prevent progression of DR are:

1. Tight blood sugar control (glycemic control)

2. Blood pressure and lipid management

3. Laser therapy, intravitreal anti-VEGF injections, or vitrectomy for advanced disease

2. Traditional/herbal medicines: Some herbal supplements and traditional remedies (like ginkgo biloba, bilberry, or certain Chinese herbal formulas) have been investigated for potential antioxidant or anti-inflammatory effects. However:

1. Most studies are small, poorly controlled, or preclinical (animal/cell studies)

2. There is no evidence that they reverse retinal damage or restore vision

3. They may provide supportive benefits, but they cannot replace standard medical therapy

3. Potential risks: Herbal remedies can sometimes interact with diabetes medications, affect blood pressure, or cause liver/kidney issues. Therefore, patients must discuss any supplements with their healthcare provider.

Bottom line: Traditional or herbal medicines may have a supportive role in eye health or diabetes management, but they cannot cure diabetic retinopathy. Early detection, medical management of diabetes, and ophthalmologic interventions remain essential.

**model: Copilot**

*answer:*

Short answer: No - traditional or herbal medicines cannot cure diabetic retinopathy. They may offer supportive or adjunctive benefits, but they cannot replace proven medical treatments such as anti-VEGF injections, laser therapy, or surgery.

What the evidence shows

1. No herbal or traditional remedy has been proven to cure diabetic retinopathy

Major medical authorities (Mayo Clinic, AAO, Cleveland Clinic) state clearly that diabetic retinopathy is treatable but not curable, and delaying standard treatment risks permanent vision loss.

2. Herbal and natural products may help as complementary therapy

Systematic reviews show that some natural compounds (flavonoids, phenols, terpenoids, vitamins, etc.) have anti-inflammatory, antioxidant, and anti-neovascular effects, which may support retinal health. However, these studies emphasize that:

Evidence is limited,

Benefits are adjunctive, not curative,

More clinical trials are needed.

3. Ayurvedic or holistic approaches may help overall diabetes control

Ayurveda and holistic systems propose lifestyle changes, stress reduction, and herbal remedies to support blood sugar control and inflammation. These may help slow progression only when combined with proper medical care, not as a standalone cure.

Why herbal medicine alone cannot cure diabetic retinopathy

Diabetic retinopathy involves:

Retinal blood vessel leakage

Ischemia (loss of blood flow)

Abnormal new vessel growth

Potential retinal detachment

Once structural damage occurs, only medical treatments (anti-VEGF injections, laser photocoagulation, vitrectomy) can stop or reverse the process. Herbal remedies cannot repair damaged retinal vessels or stop neovascularization.

What herbal or natural approaches can do

These may offer supportive benefits when used alongside medical treatment:

Improve glycemic control

Reduce oxidative stress

Reduce inflammation

Support vascular health

Examples studied include flavonoids, saponins, and other plant-derived compounds. But again, these are adjuncts, not cures.

Important safety notes

Herbal supplements can interact with medications or increase bleeding risk during eye procedures.

Delaying medical treatment to try natural remedies can lead to irreversible blindness.

Always discuss supplements with your ophthalmologist.

Bottom line

Traditional or herbal medicines cannot cure diabetic retinopathy. They may provide supportive benefits for blood sugar control and inflammation, but only medical treatments can prevent vision loss.

**model: DeepSeek**

*answer:*

This is an important question. To be direct: No, traditional or herbal medicines cannot cure diabetic retinopathy (DR). There is no scientific evidence from rigorous clinical trials that any herbal or traditional remedy can reverse the advanced stages of this condition or replace proven medical treatments.

However, let's break down what these medicines can and cannot do, so you have a clear and safe understanding.

The Bottom Line Up Front

Cure? No. Once the retina is damaged by new, leaking blood vessels (proliferative DR) or macular edema, it cannot be "cured" by herbs.

Prevention & Adjunct? Possibly. Some herbs may have a supportive role in managing blood sugar, inflammation, and oxidative stress-key drivers of DR. But this is only ever alongside standard medical care, not instead of it.

Risk of Harm? Significant. Some herbs can interact dangerously with diabetes medications or worsen eye health.

Why Herbal Medicine Falls Short of a "Cure"

Diabetic retinopathy is caused by years of high blood sugar damaging the tiny blood vessels in the retina. This leads to:

Leaky vessels (macular edema - swelling that blurs vision).

Clogged vessels (leading to retinal ischemia - lack of oxygen).

Abnormal new vessel growth (proliferative DR - fragile vessels that bleed easily, causing floaters, blindness, or retinal detachment).

Standard medicine cures the effects of DR using:

Laser (to seal leaking vessels or burn oxygen-starved areas).

Anti-VEGF injections (to stop abnormal vessel growth and reduce swelling).

Steroid implants (for persistent swelling).

Vitrectomy surgery (to remove blood or scar tissue).

No herb can laser a vessel or block VEGF (vascular endothelial growth factor) as powerfully and specifically as an injectable drug.

What the Research Actually Says (Honest Summary)

Some individual herbs or formulas have been studied for DR, mostly in animals or small human pilot studies. The quality of evidence is low to very low. Here are a few commonly mentioned ones:

Herb; Proposed Mechanism; Evidence Level; Key Limitation

Astragalus (Astragalus membranaceus); Reduces oxidative stress, lowers blood sugar; Very low (mostly lab/animal); No high-quality human trial showing vision improvement.

Berberine (from Coptis, Goldenseal); Activates AMPK pathway (improves metabolism), anti-inflammatory; Low (small human studies for metabolic effects, not directly for DR); Poor absorption, potential drug interactions (CYP450, digoxin, cyclosporine).

Bilberry (Vaccinium myrtillus); Contains anthocyanins (antioxidants), historically used for night vision; Very low; controlled trials show no benefit for diabetic retinopathy; Popular myth from WWII RAF pilots; modern trials negative.

Ginkgo (Ginkgo biloba); Improves blood flow, anticoagulant effect; Very low; concerns about bleeding risk; Potentially harmful - can increase risk of retinal or vitreous hemorrhage in proliferative DR.

Goji berry (Lycium barbarum); Carotenoids (zeaxanthin), antioxidant; Very low (small short-term trials on retinal thickness); May interact with warfarin (blood thinner) and diabetes drugs.

TCM formulas (e.g., Qiming, Shuangdan, Baoyuan); Multiple targets - inflammation, vascular permeability; Low; one small RCT showed reduced macular thickness vs. placebo; Needs larger trials; not a cure, only a possible mild adjunct.

Note on Traditional Chinese Medicine (TCM): TCM approaches DR as "retinal blood stasis" and "yin deficiency with fire". Prescribed formulas (like Qi Ju Di Huang Wan modified) aim to improve circulation and clear heat. Some very small RCTs (randomized controlled trials) from China suggest a possible mild benefit in slowing non-proliferative DR progression when added to tight glycemic control. However, these studies have high risk of bias, and the effect is modest at best-nowhere near a cure.

The Real Dangers of Relying on Herbal Medicine for DR

Delaying sight-saving treatment. DR can progress silently. If you choose herbs instead of laser or injections, you can go from seeing 20/20 to legal blindness in a few months. Once vision is lost from macular scarring or retinal detachment, it is permanent.

Herb-drug interactions.

Bleeding risk: Ginkgo, danshen (Salvia), ginger, ginseng, garlic (in high doses), and bilberry (mildly) can increase bleeding risk if taken with antiplatelets (aspirin, clopidogrel) or anticoagulants (warfarin, apixaban). This is especially dangerous if you later need eye injections or surgery.

Liver/kidney toxicity: Unregulated or misidentified herbs (e.g., aristolochic acid in some TCM preparations, or high doses of certain Ayurvedic minerals) can cause liver failure or kidney injury-worsening diabetic complications.

Hypoglycemia: Some herbs (bitter melon, fenugreek, gymnema) can lower blood glucose too much when taken with insulin or sulfonylureas, leading to dangerous lows.

No standardization. Herbal products vary wildly. Some contain hidden pharmaceuticals (steroids, NSAIDs, even oral diabetes drugs), heavy metals, or pesticides.

What Actually Works to Prevent and Treat Diabetic Retinopathy (Evidence-Based)

Stage; What Works; Herbal Role?

No DR - Prevention; Strict glycemic control (HbA1c <7% ideally - individualized), blood pressure control (<130/80), LDL cholesterol management, smoking cessation, annual dilated eye exams.; No role, except possibly as part of a hypoglycemic lifestyle (e.g., using spices instead of salt/sugar). Do not replace metformin or insulin.

Mild to Moderate Non-Proliferative DR; Same as above. No eye treatment needed unless macular edema develops.; Unproven. Some small studies suggest certain antioxidants (vitamins C, E, zinc - in very high doses? No proven benefit in large trials like ETDRS or AREDS2). Do not use.

Diabetic Macular Edema; Anti-VEGF injections (ranibizumab, aflibercept, bevacizumab) - first line. Focal laser is second line. Steroid implants for certain cases.; No. Injections reduce swelling by 50-60% and improve vision. Herbs cannot match this.

Proliferative DR; Panretinal laser (to shrink abnormal vessels) + anti-VEGF injections. Vitrectomy for non-clearing hemorrhage or traction detachment.; No and dangerous - any herb with anticoagulant effect (ginkgo, danshen, garlic, ginseng) raises hemorrhage risk in eyes with fragile new vessels.

Traction Retinal Detachment / Neovascular Glaucoma; Surgery and/or glaucoma procedures.; Absolutely none. Permanent blindness if not treated urgently.

A Reasonable Approach: Integration vs. Rejection

If you still wish to explore herbal medicine as a supportive measure for general diabetic health (not a treatment for DR), please follow these safety rules:

Get a confirmed DR severity grading from a retina specialist. If you have proliferative DR or macular edema, do not take any herb without explicit permission from your ophthalmologist.

Only under the guidance of a qualified practitioner (e.g., a licensed, evidence-informed TCM doctor or clinical herbalist trained in drug-herb interactions), and be transparent with your endocrinologist and ophthalmologist.

Avoid herbs with known bleeding risk if you have moderate/severe non-proliferative or proliferative DR, or if you take aspirin/clopidogrel/anticoagulants.

Do not replace standard treatments. Use herbs after you have optimized blood sugar, blood pressure, and lipids, and while receiving indicated eye treatments (laser, injections, surgery).

Monitor your blood glucose closely if the herb is known to lower sugar.

Final Verdict

Traditional or herbal medicines do not cure diabetic retinopathy. Relying on them to do so risks irreversible blindness.

Their only plausible role is as a possible, unproven, low-grade adjunct to standard medical care-mostly for general metabolic health-in patients with early, non-proliferative DR and under close medical supervision. For anyone with vision-threatening DR (macular edema or proliferative disease), herbs are not only ineffective but potentially harmful.

The only proven "cure" for diabetic retinopathy is preventing it through tight diabetes control. Once it becomes advanced, modern procedures (laser, injections, surgery) are the only things that save sight.

**model: Doubao**

*answer:*

Short answer: No traditional or herbal medicine can cure diabetic retinopathy (DR). There is no proven herbal/traditional therapy that can reverse advanced DR or permanently eliminate the disease.

Key evidence & clarification

No curative effectDR is a chronic, progressive microvascular complication of long-term hyperglycemia. Damage to retinal capillaries, ischemia, edema, and neovascularization are structural organic changes. No herbal medicine, traditional Chinese medicine formula, or folk herbal remedy can repair damaged retinal blood vessels fundamentally or cure the condition.

Possible adjunctive role (not a cure)Some standardized herbal/traditional formulations may serve as adjuvant therapy under formal ophthalmology supervision:

Mild non-proliferative DR: May help improve retinal microcirculation, reduce macular edema progression, and relieve blood stasis symptoms.

Cannot replace core standard treatments: blood sugar/blood pressure/lipid control, retinal laser photocoagulation, intravitreal anti-VEGF injections, vitreoretinal surgery.

Risks of relying solely on herbal medicine

Delays evidence-based standard treatment, leading to irreversible vision loss, macular degeneration, retinal detachment, or blindness.

Many herbal products have unknown interactions with diabetes medications, may affect liver/kidney function, or cause blood sugar fluctuations.

Clinical guideline consensusGlobal ophthalmology and diabetes guidelines (ADA, EASD, AAO) do not recognize any traditional/herbal medicine as a curative treatment for DR. They only allow controlled adjuvant use alongside conventional medical and ophthalmological intervention.

Conclusion

Cure: Impossible with traditional/herbal medicine.

Safe use: Can only be used as adjuvant auxiliary care with prescription from a professional ophthalmologist and TCM physician, while strictly adhering to blood sugar management and regular retinal screening + standard Western ophthalmic treatment.

**model: Gemini**

*answer:*

The short answer is no, traditional or herbal medicines cannot "cure" diabetic retinopathy in the sense of permanently reversing the disease or removing the need for medical management.

However, clinical research-much of it centered on Traditional Chinese Medicine (TCM)-suggests that certain herbal formulations can be effective supportive therapies to help slow progression, manage symptoms like blurred vision, and improve retinal blood flow.

Current Scientific Standing

Supportive, Not Curative: Herbs are generally used to manage early-stage (non-proliferative) retinopathy. Once the disease reaches advanced stages (proliferative retinopathy or macular edema), herbal treatments cannot replace standard medical interventions like laser therapy, anti-VEGF injections, or surgery.

Combination Therapy: Most successful clinical trials involve using herbal medicine alongside "Western" treatments (conventional blood sugar and blood pressure control). Meta-analyses suggest that this combined approach often yields better results for visual acuity than conventional treatment alone.

Quality of Evidence: While many small-scale studies show promise, large-scale, high-certainty clinical evidence is still limited. Many international health organizations view herbal treatments as "experimental" or "complementary" rather than primary treatments.

Commonly Studied Herbal Treatments

Research has identified several specific compounds and formulas that may help protect the retina:

Herb/Formula; Potential Benefit; Key Mechanism

Compound Danshen (Salvia); Improves retinal microcirculation.; Activates blood circulation and reduces "blood stasis."

Bilberry Extract; Strengthens blood vessels and improves night vision.; High in anthocyanosides (antioxidants).

Qiming Granules; Alleviates retinal hypoxia and ischemia.; Often used in China for early-stage NPDR.

Astragalus (Huangqi); Reduces oxidative stress and inflammation.; Neuroprotective effects on retinal cells.

Ginkgo Biloba; May improve visual function in mild cases.; Enhances blood flow to the small vessels of the eye.

Risks and Considerations

1. Delayed Treatment: The biggest risk of relying solely on herbal medicine is the potential delay of proven medical treatments. Diabetic retinopathy can progress silently; waiting until vision is lost to seek conventional care can lead to permanent blindness.

2. Blood Sugar Interference: Some herbs can interact with insulin or oral diabetes medications, potentially causing dangerous drops in blood sugar (hypoglycemia).

3. Liver and Kidney Strain: Because diabetes already stresses the kidneys, it is vital to ensure any herbal supplements are high-quality and free of heavy metals or contaminants.

Recommendation

If you are considering herbal options, they should be viewed as a complement to-not a replacement for-regular eye exams and blood sugar management. Always consult with both an ophthalmologist and your primary care physician before adding supplements to your regimen to ensure they won't interfere with your current medications.

**model: Perplexity**

*answer:*

No-traditional or herbal medicines have not been proven to cure diabetic retinopathy. The best available reviews suggest some herbal treatments may improve measures like visual acuity in certain studies, but the evidence is low certainty, biased, and does not show that they stop or reverse the disease.

What the evidence says

A Cochrane review found that none of the included trials reported the key outcome of retinopathy progression, so there is no solid proof that herbal medicine can prevent worsening of diabetic retinopathy. Another review of traditional Chinese medicine reported possible improvements in visual acuity, microaneurysms, and HbA1c, but it also concluded that further trials are needed for reliable evidence.

Practical meaning

Herbal or traditional remedies may be used as adjuncts in some settings, but they should not replace proven care such as blood sugar control, blood pressure control, eye examinations, laser treatment when indicated, anti-VEGF injections, or surgery for advanced disease. Some reviews describe plausible anti-inflammatory or microcirculation effects, but that is not the same as a cure.

Safety note

"Natural" does not automatically mean safe, and some herbal products can interact with diabetes medicines or affect bleeding risk. Because diabetic retinopathy can progress silently, the safest approach is to treat any herbal use as supplemental and discuss it with an eye doctor or diabetes clinician.

**Q14. If I have type 1 diabetes, when should I have my first dilated eye exam?**

**model: ChatGPT**

*answer:*

For a person with type 1 diabetes, the recommendation for the first dilated eye exam is generally:

Within 5 years of diabetes onset for children and adolescents.

For adults diagnosed with type 1 diabetes, the first comprehensive dilated and retinal examination should also occur within 5 years after diagnosis.

After the initial exam, the frequency of follow-up depends on the findings:

No diabetic retinopathy: every 1-2 years.

Mild to moderate non-proliferative diabetic retinopathy: annually or more frequently.

Severe non-proliferative or proliferative diabetic retinopathy: more frequent, as recommended by an ophthalmologist.

These guidelines are consistent with the American Diabetes Association (ADA) Standards of Care in Diabetes-2025 and the American Academy of Ophthalmology (AAO) Preferred Practice Patterns for Diabetic Retinopathy.

**model: Copilot**

*answer:*

A person with type 1 diabetes should have their first dilated, comprehensive eye exam within 5 years of diabetes onset. This timing is based on strong evidence that diabetic retinopathy rarely develops earlier than 5 years after hyperglycemia begins. This recommendation is confirmed by both the American Diabetes Association (ADA) and NCQA.

Why the first exam is at 5 years

Retinopathy takes time to develop - typically at least 5 years after the onset of type 1 diabetes.

After that first exam, annual exams are recommended, with the option to extend to every 1-2 years if exams remain normal and blood sugar is well controlled.

What counts as the correct exam?

A dilated and comprehensive eye exam performed by an ophthalmologist or optometrist. This exam looks for:

Diabetic retinopathy

Macular edema

Glaucoma

Cataracts

Summary (the key point)

Initial exam: Within 5 years

**model: DeepSeek**

*answer:*

According to the American Diabetes Association (ADA) and other major endocrinology guidelines, a person with type 1 diabetes should have their first dilated eye exam within 5 years of their diagnosis, provided they are age 10 or older.

Here's the breakdown:

If diagnosed before age 10: The 5-year clock starts at age 10 (i.e., first exam at age 10-11, regardless of diagnosis year).

If diagnosed at age 10 or older: First exam within 5 years of diagnosis.

After the first exam, most guidelines recommend a dilated eye exam annually, though your eye care provider may extend the interval to every 1-2 years if no retinopathy is present and blood sugar control is stable.

Important exception: If you have any signs of retinopathy or poor glycemic control from the start, the first exam may be recommended earlier than 5 years.

Note: This differs from type 2 diabetes, where the first dilated eye exam is recommended at the time of diagnosis because type 2 often goes undiagnosed for many years.

**model: Doubao**

*answer:*

For Type 1 Diabetes:The first dilated comprehensive eye exam should be done within 5 years after diabetes diagnosis, and no later than age 10.

After the first exam:

Annual dilated retinal screening is recommended for life.

If retinopathy is found, follow-up exams are scheduled more frequently (every 6 months or as advised by an ophthalmologist).

**model: Gemini**

*answer:*

According to guidelines from the American Diabetes Association (ADA) and the American Academy of Ophthalmology (AAO), the timing for the first dilated eye exam for a person with type 1 diabetes depends on their age at diagnosis.

Standard Timing Guidelines

Adults (Diagnosed as adults): The first dilated, comprehensive eye exam should occur within 5 years of the initial diagnosis.

Children and Adolescents: The first exam is typically recommended 3 to 5 years after diagnosis, provided the child is at least age 10 or 11, or has reached puberty (whichever comes first).

Retinopathy is extremely rare before puberty or within the first few years of type 1 diabetes, which is why the 5-year grace period is generally safe.

Special Circumstances

While the "5-year rule" is standard for type 1, there are exceptions where an exam should happen sooner:

Pregnancy: If a person with type 1 diabetes is planning to become pregnant or has already become pregnant, an eye exam should be scheduled before conception or in the first trimester. Pregnancy can significantly accelerate the progression of diabetic retinopathy.

Symptoms: If a person experiences any sudden vision changes (blurriness, floaters, or "flashing lights"), an exam should be scheduled immediately, regardless of how long they have had diabetes.

Why is it different from Type 2?

Unlike type 2 diabetes-where an eye exam is required immediately at diagnosis because the condition may have been present undiagnosed for years-type 1 usually has a clear onset. It takes time for high blood sugar to cause detectable damage to the retinal blood vessels, hence the 5-year window.

Follow-up Schedule

After the initial exam, patients with type 1 diabetes should generally have a dilated eye exam every year. If there is no evidence of retinopathy after one or more annual exams and blood sugar is well-controlled, some eye care professionals may suggest screening every 1-2 years.

**model: Perplexity**

*answer:*

If you have type 1 diabetes, your first dilated eye exam should be within 5 years of diagnosis, and then you should have it every year after that.

If you've already had vision changes, eye pain, or been told you have diabetic eye disease, your eye doctor may want to see you sooner and more often

**Q15. If I have type 2 diabetes, when should I have my first dilated eye exam?**

**model: ChatGPT**

*answer:*

For people with type 2 diabetes, current guidelines recommend that the first dilated eye exam should be conducted at the time of diagnosis. This is because type 2 diabetes can often be present for years before being detected, and some patients may already have signs of diabetic retinopathy at diagnosis.

After the initial exam:

If no diabetic retinopathy is found, exams are usually recommended every 1-2 years.

If any level of diabetic retinopathy is present, follow-up frequency is determined by severity, ranging from every 3-12 months.

This approach aligns with recommendations from the American Diabetes Association (ADA) and the American Academy of Ophthalmology (AAO).

**model: Copilot**

*answer:*

A person with type 2 diabetes should have their first dilated, comprehensive eye exam at the time of diagnosis. This is because type 2 diabetes often develops slowly, and many people have had elevated blood sugar for years before being diagnosed - long enough for eye damage to begin.

Evidence from authoritative sources supports this:

The American Diabetes Association states that people with type 2 diabetes need an eye exam soon after diagnosis because the disease may have been present for some time before detection.

The NCQA (which summarizes ADA Standards of Care) also states that adults with type 2 diabetes should have an initial dilated eye exam at the time of diagnosis.

The CDC likewise recommends an eye exam immediately after a type 2 diabetes diagnosis.

What happens after the first exam?

If no diabetic retinopathy is found and blood sugar is well controlled, follow-up exams may be needed every 1-2 years.

If any retinopathy is present, exams should occur at least annually, and more often if disease is progressing.

Why this matters

Diabetic retinopathy is the leading cause of blindness in working-age adults, and early stages often have no symptoms. Early detection through a dilated exam is the best way to prevent vision loss.

**model: DeepSeek**

*answer:*

According to the American Diabetes Association (ADA) standards of care, a person with type 2 diabetes should have their first dilated eye exam at the time of diagnosis.

Unlike type 1 diabetes (where the exam typically starts 5 years after diagnosis), type 2 diabetes often remains undiagnosed for several years, meaning significant hyperglycemia and potential diabetic retinopathy may already be present at diagnosis. Therefore, the initial comprehensive dilated eye exam by an ophthalmologist or optometrist should occur immediately upon diagnosis of type 2 diabetes.

**model: Doubao**

*answer:*

For Type 2 Diabetes:A person with type 2 diabetes should have their first dilated comprehensive eye exam immediately at the time of diagnosis - no waiting period.

Key guideline rationale

Unlike type 1 diabetes (screening starts 5 years after diagnosis), type 2 diabetes often has a prolonged asymptomatic pre-diabetic phase. Retinal damage can already be present by the time type 2 diabetes is formally diagnosed.

Follow-up

If the exam is normal: repeat annually.

If mild diabetic retinopathy is found: more frequent monitoring (every 6 months or as ophthalmologist recommends).

**model: Gemini**

*answer:*

For a person with type 2 diabetes, the first dilated eye exam should occur at the time of diagnosis.

Unlike type 1 diabetes, where the onset is usually sudden and clear, type 2 diabetes can go undiagnosed for years. Consequently, about 1 in 5 people already have some degree of retinopathy (damage to the retina) by the time they are officially diagnosed.

Screening Guidelines

According to the American Diabetes Association (ADA) and the American Academy of Ophthalmology (AAO), the schedule is as follows:

Initial Exam: Immediately upon diagnosis of type 2 diabetes.

Follow-up Exams: Annually: If any signs of retinopathy are found, or if blood sugar is not well-controlled.

Every 1-2 Years: If one or more annual exams are completely normal and blood sugar is well-managed, your eye doctor might suggest a longer interval.

Pregnancy: If someone with type 2 diabetes is planning pregnancy or becomes pregnant, an exam should be scheduled before conception or in the first trimester, as pregnancy can accelerate the progression of eye issues.

Why Dilation is Necessary

A standard vision test or a quick look with a light isn't enough to catch early diabetic eye disease. The doctor uses drops to widen the pupil so they can see the entire retina, optic nerve, and blood vessels at the back of the eye. Early stages of diabetic retinopathy often have no symptoms, so this exam is the only way to catch it before it affects your sight.

Feature; Type 1 Diabetes; Type 2 Diabetes

First Exam; Within 5 years of diagnosis; At the time of diagnosis

Why?; Retinopathy takes time to develop after onset.; The disease may have been present for years before diagnosis.

**model: Perplexity**

*answer:*

If you have type 2 diabetes, your first dilated eye exam should be scheduled soon after diagnosis-ideally right away or within the first few months. The reason is that type 2 diabetes can affect the eyes before you notice any symptoms.

After that first exam, the usual follow-up is every year, though your eye doctor may adjust the interval based on your results

**Q16. How often should people with diabetes have a dilated eye exam?**

**model: ChatGPT**

*answer:*

People with diabetes should have a comprehensive dilated eye exam to check for diabetic retinopathy according to the type of diabetes and the condition of their eyes:

Type 1 Diabetes: The first dilated eye exam is recommended within 3-5 years after diagnosis.

Type 2 Diabetes: Because the disease may have been present before diagnosis, a dilated eye exam should be done at the time of diagnosis.

During Pregnancy: Women with pre-existing diabetes should have an eye exam in the first trimester and be closely monitored throughout pregnancy.

Follow-up frequency depends on the findings:

No or minimal retinopathy: Exams are typically recommended every 1-2 years.

Mild to moderate non-proliferative diabetic retinopathy: Exams every 6-12 months.

Severe non-proliferative or proliferative diabetic retinopathy: Exams every 3-6 months, often with treatment such as laser or injections.

Even if you have no vision symptoms, regular dilated exams are essential, because early diabetic retinopathy often has no symptoms but can still progress to vision loss if not detected early.

**model: Copilot**

*answer:*

People with diabetes should have a dilated eye exam every year, with some exceptions based on diabetes type and existing eye disease. This schedule is grounded in current guidance from the American Diabetes Association and related clinical standards.

Core recommendation

A yearly dilated and comprehensive eye exam is the standard for most people with diabetes. This helps detect diabetic retinopathy early-often before symptoms appear.

How often by diabetes type

Type 1 diabetes - First exam within 5 years of diagnosis, then every year afterward.

Type 2 diabetes - First exam at the time of diagnosis, then every year. (Because type 2 can be present for years before detection.)

Pregnancy with diabetes - Exam before pregnancy or in the first trimester, then each trimester if retinopathy is present, and sometimes up to 1 year postpartum.

When exams may be more or less frequent

If no retinopathy is found and blood sugar is well controlled: Screening every 1-2 years may be considered.

If any retinopathy is present: Exams should occur at least annually, and every 3-6 months if disease is progressing.

Why this matters

Diabetic retinopathy is the leading cause of blindness in adults 20-74. Regular dilated exams catch changes early, when treatment is most effective.

What a dilated exam looks like

**model: DeepSeek**

*answer:*

The general recommendation for people with diabetes is to have a dilated eye exam at least once a year. However, depending on the type and duration of diabetes, blood sugar control, and whether any existing eye damage (diabetic retinopathy) is present, your eye doctor may recommend more frequent exams-for example, every 6 to 9 months. If you have no signs of retinopathy and good glucose control, some guidelines suggest an exam every 1 to 2 years, but yearly is the standard for safety. Always follow your individual ophthalmologist's advice.

**model: Doubao**

*answer:*

For people with diabetes, the recommended frequency of dilated eye exams follows standard clinical guidelines:

Type 1 DiabetesStart a dilated eye exam within 5 years after diabetes diagnosis, then once every year thereafter.

Type 2 DiabetesHave a dilated eye exam right at the time of diagnosis, then annually.

During pregnancy with diabetesNeed an eye exam before pregnancy or early in the first trimester, with follow-up exams as advised by an ophthalmologist.

If diabetic retinopathy is already presentMore frequent monitoring (every 6 months or shorter intervals) is required, based on the severity of retinal changes assessed by an eye doctor.

**model: Gemini**

*answer:*

The timing for a dilated eye exam depends largely on the type of diabetes you have and how well it's being managed. Because many eye complications don't show symptoms until they are advanced, regular screening is a "must-do" for long-term vision health.

According to the 2026 American Diabetes Association (ADA) and American Academy of Ophthalmology (AAO) standards, here are the general guidelines:

Recommended Screening Intervals

Group; Initial Exam Timing; Follow-Up Frequency

Type 1 Diabetes; Within 5 years of diagnosis.; At least annually.

Type 2 Diabetes; At the time of diagnosis.; At least annually.

Pregnancy; Before conception or in the 1st trimester.; Every trimester and 1 year postpartum (as indicated).

Important Nuances

Low-Risk "Extended" Intervals: If your eye exams have been normal for one or more years and your blood glucose is well-controlled, your doctor may consider moving to an exam every 1-2 years.

High-Risk Frequent Exams: If you already show signs of diabetic retinopathy or other issues, you will likely need exams more frequently than once a year to monitor for progression.

The Role of AI Screening: You might encounter AI-based retinal imaging in primary care clinics now. While these are excellent for quick screenings, they don't always replace the need for a comprehensive dilated exam, especially if an abnormality is detected.

Children and Adolescents: For those with Type 1, screening usually begins at age 11 or at the start of puberty (whichever is earlier), once they have had diabetes for 3-5 years.

Why the difference between Type 1 and Type 2?

People with Type 2 diabetes often have high blood sugar for years before they are officially diagnosed, meaning eye damage could already be present. In Type 1, the onset is usually sudden, and it typically takes several years of hyperglycemia for detectable retinopathy to develop.

If you notice sudden changes-like dark spots (floaters), flashes of light, or blurry vision-don't wait for your annual appointment; schedule an exam immediately.

**model: Perplexity**

*answer:*

People with diabetes should generally have a dilated eye exam once a year. For type 1 diabetes, the first exam is usually within 5 years of diagnosis; for type 2 diabetes, it should be soon after diagnosis, then typically yearly after that.

If an eye doctor finds diabetic eye disease or other concerning changes, exams may need to happen more often than once a year

**Q17. If my last eye exam was normal, can I wait longer than one year?**

**model: ChatGPT**

*answer:*

For people with diabetes who have had a normal eye exam, the typical recommendation is to continue annual screenings. Even if the last exam was normal, diabetic eye disease can develop without noticeable symptoms, and annual monitoring helps detect it early.

Some nuances:

If you have type 1 diabetes, the first eye exam is usually 5 years after diagnosis, then annually.

For type 2 diabetes, the first exam is at diagnosis, then usually every year.

Some clinicians may consider extending the interval to 18-24 months in adults with type 2 diabetes who have had multiple consecutive normal exams, good blood sugar control, and no other risk factors. However, this should only be done under guidance from your eye doctor.

So, you generally shouldn't delay more than a year without discussing it with your ophthalmologist, even if your last exam was normal. Annual checks remain the safest approach.

**model: Copilot**

*answer:*

Yes - if your last eye exam was normal and you have no risk factors, you can usually wait longer than one year. Most major guidelines support extending the interval to 2-4 years for healthy adults under 55 with no symptoms or risk factors.

What the guidelines say (based on your situation)

The American Academy of Ophthalmology recommends that adults 40-54 years old with no symptoms and no risk factors get an eye exam every 2-4 years rather than annually.

The Cleveland Clinic echoes this: if you're under 40, have 20/20 vision, and no risk factors, your doctor may advise less frequent than yearly exams. For ages 40-54, they recommend every 2-4 years if healthy and asymptomatic.

So yes - a normal exam buys you more time, as long as nothing else puts you at higher risk.

When you should not wait longer

You should stick to annual exams if you have any of these risk factors:

Diabetes - requires yearly dilated exams

High blood pressure - can cause retinal changes

Family history of glaucoma or macular degeneration

Wearing glasses or contacts

Past eye surgery or injury

Medications with eye side effects (e.g., steroids)

If any of these apply, annual exams are safer.

A simple rule of thumb

Healthy, no symptoms, no risk factors: every 2-4 years

Age 55-64: every 1-3 years

Age 65+: every 1-2 years

Any risk factor: every year

When to go sooner even if you're "not due"

Schedule an exam right away if you notice:

New floaters or flashes

Blurry or double vision

Trouble seeing at night

Eye pain or pressure

A sudden change in vision

These can signal urgent issues.

**model: DeepSeek**

*answer:*

This is a great question, and the answer isn't a simple "yes" or "no" for everyone. While a normal exam is reassuring, the one-year recommendation depends heavily on your age, health, and risk factors.

Here's a breakdown to help you decide.

The Short Answer

For a healthy adult (20-39) with no risk factors and a truly normal exam, you can often safely wait 2 to 3 years.

However, for people over 40, or those with any risk factors, waiting longer than a year is not recommended.

The Detailed Breakdown by Age and Risk

The American Academy of Ophthalmology provides these general guidelines:

1. Ages 20-39: Low Risk

Recommendation: Every 2-3 years.

Reasoning: The risk of serious eye disease is very low. The main goal is to update your glasses/contact prescription and screen for rare, early-onset issues.

Can you wait longer than 1 year? Yes, probably. Waiting 2 years is very safe.

2. Ages 40-64: The Critical Decade

Recommendation: Every 2-4 years, but many experts suggest every 1-2 years starting at 40.

Reasoning: This is when age-related conditions begin, especially presbyopia (need for reading glasses), and early signs of cataracts, glaucoma, or macular degeneration can appear.

Can you wait longer than 1 year? Be cautious. A "normal" exam at 40 can change significantly by age 42. Waiting 2 years is often the maximum recommended.

3. Ages 65+

Recommendation: Every 1-2 years.

Reasoning: Risk for all major eye diseases (glaucoma, cataracts, macular degeneration, diabetic retinopathy) increases sharply. These diseases start without symptoms.

Can you wait longer than 1 year? Not recommended. A yearly check is the safest approach.

Do You Have Any of These "Risk Factors"?

This is the most important part. If you answer yes to any of the following, you should not wait longer than one year, even if your last exam was perfect.

Diabetes (Type 1 or 2): High risk for diabetic retinopathy, which can cause sudden, irreversible blindness. Annual exams are mandatory.

High Blood Pressure or Heart Disease: Can affect blood vessels in the eye.

Family History of Glaucoma or Macular Degeneration: Your genes significantly increase your risk.

High Nearsightedness (Myopia > -6.00 diopters): Higher risk of retinal detachment, glaucoma, and cataracts.

Previous Eye Injury or Eye Surgery.

Certain Medications: Long-term use of steroids (prednisone), hydroxychloroquine (Plaquenil), or tamoxifen.

Symptoms: Any new floaters, flashes of light, curtain over vision, eye pain, redness, or blurred vision.

What Does "Normal Exam" Really Mean?

A "normal" exam is not just a vision test for glasses. A comprehensive, "normal" eye exam should include:

Visual acuity (the eye chart).

Pupil dilation (to look at your retina and optic nerve).

Tonometry (the "puff of air" test for eye pressure).

Visual field test (in some cases).

If your "normal" exam did NOT include dilation, it was incomplete. Eye diseases can be missed. You should be more cautious about waiting longer.

The One Scenario You Can't Ignore: Sudden Changes

No matter when your last exam was, do not wait for your next scheduled appointment if you experience:

Sudden increase in floaters (a swarm of new spots or "cobwebs")

Flashes of light (like a camera flash in your peripheral vision)

A dark curtain or shadow covering any part of your vision

Sudden blurred or lost vision (even if it comes back)

Eye pain or redness

These are emergency symptoms.

Final Verdict: A Decision Guide

Your Profile; Can you wait >1 year?; Max recommended interval

Under 40, healthy, no risk factors, dilated exam; Yes; 2-3 years

Over 40, healthy, no risk factors, dilated exam; Cautiously, maybe; 2 years (1 year is safer)

Over 65, even if exam was normal; No; 1 year

Any risk factor (diabetes, family history, high myopia); No; 1 year (or as directed by your doctor)

The safest, single best answer: Call your eye doctor's office and ask. They have your full history and dilation results. You can say, "My exam last year was normal. Based on my specific age and risk factors, what interval do you recommend for me?"

**model: Doubao**

*answer:*

It depends on your age, eye health, family history, and general health - a normal routine eye exam doesn't always mean you can safely wait longer than 1 year.

General Guidelines

Adults under 40, no risk factorsNormal healthy eyes, no diabetes, no high eye pressure, no family history of glaucoma/retinal disease:You can often extend to 1-2 years safely.

Age 40 and aboveEven with a normal exam: do not wait longer than 1 year.After 40, glaucoma, presbyopia, early macular changes can develop silently with no symptoms.

If you have any of these to stick to yearly exams (no extension)

Diabetes, high blood pressure

Family history of glaucoma, macular degeneration, retinal detachment

Nearsighted (especially high myopia)

Dry eye, frequent eye strain

History of eye injury or surgery

Key point

Many serious eye conditions are asymptomatic early on - they show up on an exam long before you feel any vision changes. A "normal" result only means your eyes were healthy that day, not that they won't change in 12-24 months.

**model: Gemini**

*answer:*

Whether you can stretch the time between eye exams depends heavily on your age, your general health, and any underlying conditions. While a "normal" result is great news, eye health can change subtly before you notice symptoms.

Here is a breakdown of the standard recommendations based on different risk profiles:

Guidelines for Healthy Adults

If you have no symptoms, no family history of eye disease, and do not wear corrective lenses, the general consensus among optometric associations is:

Ages 18 to 39: Every 2 years is usually sufficient.

Ages 40 to 64: Every 1 to 2 years. This is the window where age-related changes, like presbyopia (difficulty seeing up close), typically begin.

Ages 65 and older: Every year. The risk for cataracts, glaucoma, and macular degeneration increases significantly in this bracket.

When a One-Year Wait is Mandatory

Even if your last exam was normal, you should not wait longer than a year if any of the following apply:

Diabetes or Hypertension: These conditions can damage the blood vessels in the retina (retinopathy) without causing immediate pain or vision loss.

Corrective Lenses: If you wear glasses or contacts, an annual exam ensures your prescription is current and your eyes aren't being strained.

Family History: A history of glaucoma or macular degeneration often requires more frequent monitoring.

High Myopia (Nearsightedness): Severe nearsightedness carries a higher risk of retinal tears or detachment.

Red Flags: When to Go Sooner

Regardless of when your last exam was, you should book an appointment immediately if you experience:

1. New "floaters" or flashes of light (could indicate a retinal issue).

2. Sudden blurry vision or loss of peripheral vision.

3. Eye pain or unusual redness/discharge.

4. Frequent headaches associated with screen use or reading.

Summary Table

Age Group; Recommended Frequency (Normal Risk)

Children; Annually (due to rapid developmental changes)

18-39; Every 2 years

40-64; Every 1-2 years

65+; Annually

[!IMPORTANT]

Since individual health needs vary, it is always best to ask your optometrist or ophthalmologist at the end of your visit: "Based on my specific eye health today, when would you like to see me back?"

**model: Perplexity**

*answer:*

Yes, often you can wait longer than one year if your last exam was normal, but it depends on your age and risk factors. For many healthy adults under 40 with no symptoms, eye exams may be spaced out to every 2 years or even longer in some cases, while people 40+ or those with diabetes, glasses/contacts, family history of eye disease, or symptoms usually need them more often.

When longer intervals may be reasonable

If you're a healthy adult with no vision symptoms and no eye-disease risk factors, longer than 1 year is often acceptable.

Some guidance suggests adults 18-39 may go about every 2 years if vision is stable.

For adults 40-54, intervals of 2-4 years may be reasonable if there are no risk factors.

When not to wait

If you wear contacts, have diabetes, have a family history of glaucoma or macular degeneration, or notice blurry vision, headaches, flashes, floaters, eye pain, or redness, you should not stretch the interval.

Adults 65 and older are usually advised to have exams more often, typically every 1-2 years.

Practical rule

A normal exam is reassuring, but it doesn't automatically mean "skip yearly forever." The safest move is to follow the schedule your eye doctor recommended based on your age and risk profile.

**Q18. Is a retinal screening photo the same as a full dilated eye exam?**

**model: ChatGPT**

*answer:*

A retinal screening photo (often called fundus photography or teleophthalmology imaging) cannot fully replace a comprehensive dilated eye exam, though it can be a useful screening tool, especially for diabetic retinopathy. Here's the breakdown:

Strengths of retinal photos:

Can detect diabetic retinopathy and some other retinal changes.

Useful for screening large populations or patients who cannot easily visit an ophthalmologist.

Quick and non-invasive.

Limitations compared with a full dilated exam:

Peripheral retina: Photos typically focus on the central retina; peripheral lesions may be missed.

Other eye conditions: Glaucoma, cataracts, optic nerve abnormalities, or subtle macular changes may not be detected.

Quality issues: Poor image quality from cataracts, small pupils, or patient movement can reduce accuracy.

Clinical judgment: Photos cannot assess intraocular pressure, visual fields, or perform other functional tests done during a full exam.

Clinical guidelines:

Most diabetes guidelines (e.g., American Diabetes Association) recommend retinal photos as a screening tool, but a comprehensive dilated exam is still necessary if any abnormalities are found or at regular intervals (usually at least every 1-2 years for people with diabetes, depending on risk factors).

In short: retinal screening photos are screening tools, not diagnostic replacements for a full dilated eye exam.

**model: Copilot**

*answer:*

A retinal screening photo cannot fully replace a comprehensive dilated eye exam, though it can be a useful supplement or, in some low-risk cases, a temporary alternative. Dilation remains the gold standard for the most complete view of the retina and peripheral structures.

Core takeaway

Retinal photos are excellent for screening and early detection, but they do not provide the same depth, peripheral visibility, or 3D assessment that a dilated exam offers. For many patients-especially those at higher risk-dilation is still necessary.

What retinal photos can do

Retinal imaging - High-resolution photos of the back of the eye can detect glaucoma, macular degeneration, diabetic retinopathy, and other retinal diseases. They are quick, painless, and often require no dilation.

Digital retinal photography - Modern systems (e.g., Optomap) can capture up to 82-97% of the retina in a single image and allow long-term monitoring.

Comfort & convenience - No drops, no blurry vision, no light sensitivity, and immediate return to normal activities.

What retinal photos cannot replace

Dilated exams allow a full, dynamic, 3D view of the retina, optic nerve, macula, and far periphery-areas where tears, detachments, and subtle disease often begin.

Imaging provides a "snapshot," but dilation lets the doctor move your gaze to inspect areas photos may miss.

Some conditions-especially those in the far peripheral retina-are better detected with dilation.

Many practices emphasize that imaging is helpful but not a full substitute for dilation.

When imaging may be enough

Low-risk patients with no symptoms and no history of eye disease

Routine screenings, especially for diabetes, when used as a triage tool (abnormal results to referral for dilation)

Patients who cannot be dilated due to medical or occupational reasons

Even then, most experts recommend periodic dilation.

When dilation is strongly recommended

Diabetes, hypertension, autoimmune disease

High myopia

Flashes/floaters

Family history of retinal disease

Glaucoma suspicion

Any new visual symptoms

Side-by-side comparison

Exam Type; Retinal Imaging; Dilated Exam

Field of View; Wide, but not full periphery; Full retina including far periphery

3D Assessment; Limited; Full 3D evaluation

Detects Early Disease; Yes; Yes, often more thoroughly

Comfort; No drops, quick; Drops, temporary blur/light sensitivity

Best For; Screening, monitoring; Comprehensive diagnosis

Final answer

Retinal photos are a powerful screening tool but do not replace a full dilated eye exam. For the most complete assessment-especially if you have risk factors-you still need dilation.

**model: DeepSeek**

*answer:*

The short answer is: No, a retinal screening photo cannot fully replace a comprehensive dilated eye exam. While retinal photos are a powerful and valuable tool, they are not equivalent to a dilated exam.

Think of it this way: a retinal photo is like a high-quality, wide-angle photo of a specific landscape (the back of your eye). A dilated exam is like a team of surveyors going out to that same landscape at different times of day, measuring the terrain, checking the soil, testing the air quality, and looking for cracks in the foundation.

Here's a detailed breakdown of what each test does and why the dilated exam remains the gold standard.

What a Retinal Screening Photo Does Well

Captures a permanent record: It creates a digital image of the retina, optic nerve, and blood vessels. This is fantastic for comparing changes over time (e.g., "Is that freckle getting bigger?").

Excellent for diabetic retinopathy screening: It is very effective at detecting signs of diabetic eye disease, like microaneurysms, hemorrhages, and new blood vessel growth. This is why they are heavily used for diabetic teleretinal screening programs.

Non-invasive & convenient: It doesn't require drops, though pupils are often dilated for the best images with advanced cameras. Vision isn't blurred afterward.

Wide-field views: Modern "ultra-widefield" cameras can capture up to 200 degrees of the retina, seeing farther into the periphery than a standard undilated exam.

The Crucial Limitations of a Screening Photo

Even the best retinal camera misses critical information that a dilated exam provides. A photo is a static, 2D image. A doctor does much more.

What the Dilated Eye Exam Does; Why a Retinal Photo Can't Do It

1. Examines the Vitreous (the jelly in the center of the eye).; The camera focuses on the retina behind the vitreous. It can't see microscopic blood cells, inflammatory cells, or vitreous detachments floating in front.

2. Performs Dynamic Testing.; The doctor shines a light to check your pupil's reaction, checks your eye pressure with a tonometer, and has you follow a target to test the extraocular muscles. A photo is static.

3. Fully evaluates the Peripheral Retina.; While wide-field cameras help, they don't visualize the far peripheral retina perfectly, especially in undilated eyes. The doctor can use a scleral depressor (a blunt probe on the eyelid) to push the retina into view, seeing areas a camera can't reach. This is how most retinal tears or detachments are found early.

4. Views through a Small Pupil.; A screening photo requires a reasonably clear path. If you have a cataract, a small pupil, or a corneal scar, the photo quality will be poor or impossible. A skilled doctor can often examine the retina by angulating their light and lens around these obstacles.

5. Detects subtle Optic Nerve Cupping (for Glaucoma).; Glaucoma is diagnosed by assessing the 3D shape of the optic nerve head (the ratio of the cup to the disc). A photo is 2D and can be misleading. A doctor uses a slit lamp with a biomicroscope to see the depth and contour of the nerve in stereoscopic (3D) vision.

6. Diagnoses Macular Edema (swelling).; While a photo can hint at swelling, it cannot confirm it. The doctor uses a special test called OCT (Optical Coherence Tomography) - often integrated into the dilated exam - to take a cross-sectional 3D scan of the macula, measuring its thickness down to the micron. A standard photo cannot do this.

Analogy: Car Inspection

Retinal Photo: Like looking at high-resolution photos of your car's engine, tires, and undercarriage from a few angles. You can see major rust, a flat tire, or a broken belt. Great for a basic safety check.

Dilated Eye Exam: Like a mechanic putting the car on a lift, running the engine, checking the fluids, testing the brakes, probing for leaks, and listening for unusual noises. They will find problems the photos would completely miss.

So, for which patients are retinal photos acceptable as a primary screening?

Low-risk patients under 40 with no symptoms, no diabetes, no high blood pressure, and no family history of eye disease. A screening photo can serve as a good baseline.

Follow-up for stable, well-documented conditions (e.g., "Let's just get a photo to see if that stable choroidal nevus [freckle] has changed in the last 2 years").

Important Exceptions & The "Dilation is Required" List

You absolutely need a full dilated exam if you have any of the following:

Diabetes (Type 1 or Type 2) - must be examined at least annually, and the exam must evaluate for diabetic macular edema (requires OCT).

Symptoms: Flashes of light, new floaters, a curtain or shadow over your vision, or any vision loss.

History of: Retinal tear, detachment, uveitis, retinitis pigmentosa, or high myopia (severe nearsightedness).

Glaucoma suspect or patient: Must assess 3D nerve structure.

Macular degeneration: Need OCT to detect early fluid or drusen changes.

The Final Verdict

Retinal Screening Photo; Full Dilated Eye Exam

Purpose; Quick, convenient screening for obvious retinal disease (primarily diabetic retinopathy).; Comprehensive diagnostic exam for all eye health, including retina, vitreous, optic nerve, and pressure.

Can it replace?; No; Yes (the gold standard)

Best for...; Low-risk, asymptomatic patients or monitoring a known, stable spot.; Anyone with risk factors, symptoms, or who wants a complete bill of eye health.

Recommendation: If you are over 40, have diabetes or high blood pressure, or have any family history of glaucoma or macular degeneration, do not rely on a screening photo alone. Get a dilated exam every 1-2 years as recommended. Consider the retinal photo an excellent supplement, not a substitute.

**model: Doubao**

*answer:*

Short answer: No, retinal screening photography cannot fully replace a complete dilated eye exam.

Key Differences & Limitations of Retinal Screening Photos

Limited viewing rangeScreening photos typically only capture the central posterior retina (macula, optic disc). They miss the peripheral retina, where retinal tears, lattice degeneration, early retinal detachment, or peripheral diabetic retinopathy often first appear.

No real-time dynamic assessmentPhotos are static images. A dilated exam lets clinicians directly evaluate:

Retinal elevation, vitreous traction

Subtle hemorrhages, microaneurysms, or inflammatory lesions that may not show clearly on photos

Lens opacity, vitreous haze that affects true retinal visualization

Cannot evaluate anterior segment & full eye healthA full dilated exam checks not only the retina but also:Iris, lens, anterior chamber, intraocular pressure, and overall ocular structure-none are fully assessed by retinal screening photos alone.

False negatives are commonMild peripheral lesions, small breaks, or early proliferative changes can be missed on 2D screening photos, especially without dilation.

Role of Retinal Screening Photos

It is an effective screening tool, especially for:

Population-based diabetes retinopathy screening

Quick baseline documentation

Follow-up comparison of macular changes

But it is only a screening, not a comprehensive diagnostic exam.

Clinical Recommendation

Screening photo: For preliminary mass screening, high-risk population triage.

Dilated comprehensive eye exam: Required for definitive diagnosis, peripheral retina evaluation, treatment planning, and annual follow-up for diabetics, myopia, and other high-risk groups.

**model: Gemini**

*answer:*

Can a retinal screening photo replace a full dilated eye exam?

The short answer is no. While retinal screening photos are an incredible technological leap for catching problems early, they are generally considered a supplement to-rather than a replacement for-a comprehensive dilated eye exam.

Think of it like a high-resolution satellite map versus a boots-on-the-ground inspection. Here is how they compare:

The Retinal Photo (The "Snapshot")

Retinal imaging (like Fundus photography or Optos) captures a wide-angle digital image of the back of your eye.

The Pros: It provides a permanent digital record that can be compared year-over-year to track tiny changes. It is also fast and doesn't require the blurry vision that comes with dilating drops.

The Limit: It is a 2D (or occasionally 3D-simulated) "surface" view. It may miss issues on the extreme periphery of the retina or subtle structural changes that require a live, stereoscopic (3D) view.

The Dilated Eye Exam (The "Deep Dive")

During a dilated exam, a doctor uses specialized lenses and a slit lamp to look physically into your eye.

The Pros: By widening the pupil, the doctor can see the entire retina, including the far edges where tears or detachments often begin. They can also see the "depth" of the optic nerve and macula more clearly.

Comprehensive Scope: A full exam also evaluates eye pressure, muscle function, and the health of the lens (to check for cataracts), which a photo alone cannot do.

When is each used?

Feature; Retinal Screening Photo; Full Dilated Exam

Purpose; Documentation & early screening.; Comprehensive diagnosis & health check.

Depth Perception; Limited (mostly 2D).; High (3D live view).

Peripheral View; Limited to the camera's range.; Complete (360 view of the interior).

Recovery Time; Instant.; 4-6 hours of light sensitivity/blur.

The Verdict

Screening photos are excellent for routine monitoring and for those who have a very low risk of eye disease. However, if you have a history of diabetes, high blood pressure, or a family history of glaucoma, the dilated exam remains the gold standard.

If your eye doctor suggests both, they aren't being redundant-they are using the photo for a historical record and the exam for a real-time, comprehensive health assessment.

**model: Perplexity**

*answer:*

No, a retinal screening photo is not the same as a full dilated eye exam.

Key Differences

Retinal screening photos use a high-resolution camera to capture digital images of the retina without dilating drops, offering quick, non-invasive snapshots for routine checks and year-over-year comparisons. In contrast, a full dilated eye exam involves medicated drops to widen the pupils, enabling a dynamic, three-dimensional view of up to 240 degrees of the retina, including the far periphery.

When Each Is Used

Screening photos suit many healthy adults for annual exams, detecting issues like glaucoma or diabetic retinopathy without side effects like blurry vision. Dilation remains essential for high-risk cases (e.g., diabetes, suspected tears) or detailed optic nerve evaluation, providing a more complete real-time assessment.

Complementary Roles

They often work together-imaging for baseline records and dilation for thoroughness when needed-rather than one replacing the other

**Q19. Can retinal photos or AI help screen for diabetic retinopathy?**

**model: ChatGPT**

*answer:*

Yes, retinal photos and artificial intelligence (AI) can play a major role in screening for diabetic retinopathy (DR). Here's how:

1. Retinal Photography (Fundus Imaging):

A trained technician can take a photo of the back of the eye, capturing the retina. These images can be reviewed by an ophthalmologist or in specialized reading centers. Smartphone-based and portable retinal cameras are increasingly being used in primary care or remote settings, making screening more accessible.

2. AI-Assisted Screening:

AI algorithms have been developed and FDA-approved to analyze retinal images for signs of diabetic retinopathy. In practice, a retinal photo is uploaded to the AI system, which can instantly detect features like microaneurysms, hemorrhages, or neovascularization. This allows rapid, point-of-care screening without the patient needing to see an eye specialist for the initial check.

3. Advantages:

1. Early detection, even in patients without symptoms, improving outcomes.

2. Fast and scalable, useful for population-level screening or in areas with limited ophthalmology access.

3. Permanent digital record for comparison over time.

4. Limitations:

1. Retinal photos alone may not detect all complications that a full dilated exam could identify.

2. Some images can be "ungradable" due to media opacity or poor image quality, requiring follow-up with an eye specialist.

3. AI cannot replace comprehensive assessment; it's a screening tool, not a definitive diagnosis.

In summary, retinal photography and AI are effective, safe, and increasingly used for DR screening. They are particularly valuable for early detection and triage, but a full dilated eye exam by an ophthalmologist is still essential for diagnosis, staging, and treatment planning if abnormalities are detected.

**model: Copilot**

*answer:*

Yes - both retinal photos and artificial intelligence (AI) can effectively screen for diabetic retinopathy (DR), and they are already being used in real-world clinical settings. Modern AI systems trained on retinal fundus photographs can detect referable DR with expert-level accuracy, often in under a minute, and several systems are FDA-cleared for autonomous screening.

How retinal photos enable DR screening

Retinal fundus photography captures detailed images of the back of the eye, allowing clinicians - or AI systems - to identify hallmark signs of DR such as microaneurysms, hemorrhages, and neovascularization. This method is already a standard part of diabetic eye exams and is widely used in tele-ophthalmology programs.

How AI enhances DR screening

AI systems analyze retinal images to detect disease patterns with high sensitivity and specificity. Key findings from recent research and clinical deployments:

FDA-cleared autonomous systems exist. Tools like IDx-DR and EyeArt can independently analyze fundus photos and provide immediate screening decisions without a specialist. EyeArt, for example, demonstrated 96-97% sensitivity and 88-90% specificity for detecting referable DR in a large multicenter trial.

AI matches expert accuracy. Many validated algorithms exceed 85% sensitivity and specificity, comparable to trained human graders.

AI improves access and workflow. AI-based screening expands reach in primary care, reduces clinician workload, and supports remote monitoring - especially valuable where ophthalmologist availability is limited.

AI is already widely validated. EyeArt alone has been tested on nearly 2 million retinal images from over 500,000 patients globally.

Growing adoption, but challenges remain. Barriers include training, cost, reliability concerns, and ethical issues such as algorithmic bias.

Why AI screening matters

With diabetes prevalence rising worldwide and ophthalmology workforce shortages, AI-powered retinal screening offers a scalable solution. Automated retinal image analysis systems (ARIAS) are particularly valuable in low-resource settings where systematic screening is limited.

Bottom line

Retinal photos provide the raw data, and AI dramatically enhances the speed, accuracy, and scalability of diabetic retinopathy screening.

These technologies are not futuristic - they are already FDA-cleared, clinically validated, and increasingly integrated into routine diabetes care.

**model: DeepSeek**

*answer:*

Yes, both retinal photos and artificial intelligence (AI) play major roles in screening for diabetic retinopathy (DR), and they are often used together.

1. Retinal Photos (Fundus Photography)

How it helps: A technician captures images of the back of the eye (retina) to look for DR signs like microaneurysms, hemorrhages, or abnormal blood vessels.

Advantages: Provides a permanent, shareable record; reduces the need for a specialist to be physically present; can be taken in primary care or community settings.

Limitations: Requires trained photographers, image quality varies, and a human grader (e.g., ophthalmologist) must still interpret most images unless AI is used.

2. Artificial Intelligence (AI)

How it helps: Deep learning algorithms analyze retinal photos automatically to detect DR, typically grading severity (e.g., no DR vs. referable DR).

Proven performance: Many FDA-cleared or CE-marked AI systems (e.g., IDx-DR, EyeArt, AIDR) have shown sensitivity >85-90% and specificity >90% for detecting moderate-or-worse DR.

Advantages: Provides immediate results in minutes; reduces dependence on scarce eye care specialists; ideal for high-volume, low-resource, or teleretinal screening programs.

Limitations: Requires high-quality images; may perform less well in eyes with other pathologies (e.g., cataracts, macular degeneration); needs local validation and regulatory approval.

Combined use in practice:

A typical AI-assisted screening workflow:

Capture retinal photos.

AI analyzes images in real time.

If AI detects "referable DR," the patient is referred to an ophthalmologist; if "no DR" and images are adequate, they can be rescreened in 1-2 years.

This approach is endorsed by the FDA, the American Academy of Ophthalmology, and the ADA for appropriate settings.

Important considerations:

Neither method replaces a comprehensive dilated eye exam for all patients, especially those with known DR, macular edema, or poor image quality.

AI systems are screening tools, not diagnostic devices-positive findings need confirmation by a clinician.

Cost, integration with electronic health records, and medicolegal responsibility remain implementation challenges.

Conclusion: Retinal photos are the essential data source, and AI is the powerful automation tool. Together, they enable accurate, scalable, and cost-effective screening for diabetic retinopathy, particularly in primary care and underserved areas.

**model: Doubao**

*answer:*

Yes, both retinal photography and AI are highly effective, evidence-based tools for diabetic retinopathy (DR) screening, and they are widely recommended in international and domestic clinical guidelines.

1. Retinal photography for DR screening

It is the gold standard screening modality for DR.

Non-mydriatic retinal cameras can capture clear fundus images without pupil dilation, suitable for large-scale population screening, primary care, and community diabetes management.

Trained ophthalmologists or optometrists grade the photos to detect: mild/moderate/severe non-proliferative DR, proliferative DR, and diabetic macular edema (DME).

Advantages: low cost, portable, repeatable, easy for long-term follow-up of diabetic patients.

2. Artificial intelligence (AI) for DR screening

AI deep learning models are trained on millions of labeled retinal fundus images.

Capabilities:

Automatically identify DR presence, grade severity

Detect diabetic macular edema

Recognize high-risk lesions (microaneurysms, hemorrhages, exudates)

Clinical advantages:

High sensitivity and specificity, comparable to specialist ophthalmologists

Solves the global shortage of eye specialists, especially in grassroots and remote areas

Fast real-time analysis, suitable for batch screening of diabetic populations

Reduces manual reading workload

3. Combined application (Retinal photo + AI)

This is the mainstream modern screening model:

Take non-mydriatic retinal photos at primary care/hospital

AI automatically performs preliminary intelligent interpretation

Only AI-identified positive/suspected cases are referred to ophthalmologists for further diagnosis and treatment

Normal cases receive regular annual screening reminders

4. Guideline recognition

Major guidelines (ADA, WHO, Chinese Guidelines for Diabetic Retinopathy Prevention and Treatment) explicitly support retinal photography and AI-assisted DR screening as standard public health and clinical screening methods for patients with type 1 and type 2 diabetes.

**model: Gemini**

*answer:*

Retinal photography and artificial intelligence have become the modern frontline for diabetic retinopathy (DR) screening. As of 2026, these technologies are no longer just experimental; they are actively being integrated into standard clinical workflows to bridge the gap between primary care and specialized ophthalmology.

1. How Retinal Photos Help

Retinal photography-specifically fundus photography-is the primary method used to capture high-resolution images of the back of the eye.

Point-of-Care Convenience: Many modern cameras are now "non-mydriatic," meaning they can take clear photos without needing to dilate the patient's pupils with drops.

Portability: Handheld and portable fundus cameras have made it possible to conduct screenings in pharmacies, community centers, and rural clinics, significantly increasing accessibility for patients who may not have easy access to an eye specialist.

2. The Role of Artificial Intelligence

AI systems have transformed screening from a manual process of "taking a photo and sending it to a specialist" to an autonomous diagnostic process.

Instant Triage: FDA-cleared AI platforms (such as EyeArt, LumineticsCore, and AEYE-DS) can analyze retinal images in under 60 seconds.

High Accuracy: Recent 2026 clinical data indicates that these AI systems often achieve sensitivity and specificity rates between 88% and 98%, matching or even exceeding the accuracy of human graders in identifying "referable" DR (cases that need specialist intervention).

Standardized Reporting: Unlike human interpretation, which can vary, AI provides a consistent, objective analysis every time, identifying microaneurysms, hemorrhages, and signs of macular edema that might be missed by non-specialists.

3. Comparison: AI vs. Traditional Screening

Feature; Traditional Screening; AI-Powered Screening

Location; Ophthalmology or Optometry Clinic; Primary Care, Endocrinology, or Pharmacy

Wait Time; Days to weeks for results; Results in roughly 30-60 seconds

Expertise; Requires an ophthalmologist's review; Autonomous (no specialist needed on-site)

Main Goal; Comprehensive diagnosis and treatment; Rapid triage and early detection

4. Implementation in 2026

Major institutions (like Duke Eye Center) have launched large-scale programs in May 2026 to embed AI screening directly into endocrinology clinics. This "one-stop shop" approach allows patients to have their eyes screened during their routine diabetes check-up.

Important Note: While AI is an incredibly powerful screening tool, it does not replace a comprehensive eye exam. If the AI detects more-than-mild retinopathy, a referral to an ophthalmologist remains mandatory for a full clinical assessment and potential treatment (such as laser therapy or injections).

**model: Perplexity**

*answer:*

Yes-retinal photos combined with AI are now a well‐established tool for screening diabetic retinopathy and can help catch the disease earlier, especially in primary‐care and underserved settings.

How the screening works

During a routine visit, a clinician or technician takes one or more retinal (fundus) photos using a specialized camera; dilation is sometimes needed but not always, depending on the device.

An AI system analyzes the photos for signs of diabetic retinopathy (such as microaneurysms, hemorrhages, exudates, and neovascularization) and classifies the result as "no significant retinopathy," "refer‐for‐eye‐care," or "image not gradable."

What AI‐based screening can do

AI‐enabled systems can detect more‐than‐mild or even vision‐threatening diabetic retinopathy with sensitivity and specificity comparable to many human graders, making them useful for rapid, automated screening.

These tools are FDA‐cleared in some forms for autonomous detection (no ophthalmologist needed to read the image first), and they can return results in under a minute, allowing diabetes clinics, primary‐care offices, and community settings to screen more patients without over‐loading specialists.

Limitations and when it's not enough

AI screening is mainly a triage tool, not a full substitute for a dilated eye exam by an ophthalmologist or retina specialist, who can examine the optic nerve, check visual acuity, and order optical coherence tomography (OCT) or fluorescein angiography if needed.

Image quality matters: poor focus, media opacity, or cataract can lead to "ungradable" or falsely negative results, so any abnormal or inconclusive AI output should trigger a face‐to‐face eye evaluation.

In practice, retinal‐photo‐plus‐AI screening is now being deployed in large‐scale programs (for example, within primary‐care diabetes initiatives) to increase screening rates and reduce avoidable blindness, while still reserving detailed management and treatment decisions for eye specialists

**Q20. Do I still need an eye exam if I have no vision symptoms?**

**model: ChatGPT**

*answer:*

Yes, you still need regular eye exams even if you have no vision symptoms. Many eye conditions, including diabetic retinopathy, glaucoma, and macular degeneration, can develop silently without noticeable symptoms until significant damage occurs. Early detection through routine eye exams is crucial to prevent vision loss.

For people with diabetes, for example, the general guideline is:

Type 1 diabetes: Eye exam within 5 years of diagnosis, then annually.

Type 2 diabetes: Eye exam at diagnosis, then annually.

Pregnancy: Women with diabetes should have an eye exam before or early in pregnancy.

Even if your last exam was normal, you shouldn't skip your yearly check, because changes can occur between visits.

**model: Copilot**

*answer:*

You do still need periodic eye exams even when you have no symptoms. The key reason is that many serious eye diseases-like glaucoma, diabetic retinopathy, and macular degeneration-often develop silently with no early warning signs. By the time symptoms appear, damage may already be permanent. Routine exams catch these issues early, when treatment is most effective.

What the evidence shows

The American Academy of Ophthalmology recommends routine comprehensive eye exams even for adults with no symptoms and no known risk factors.

Under age 40: every 5-10 years

Age 40-54: every 2-4 years

Age 55-64: every 1-3 years

Age 65+: every 1-2 years

Routine exams are considered as essential as a general physical checkup because they evaluate both vision and overall eye health.

Why exams matter even without symptoms

Glaucoma can cause irreversible peripheral vision loss long before you notice anything.

Cataracts develop gradually and may not be obvious early on.

Retinal diseases can progress silently until central vision is affected.

Eye exams can also reveal systemic health issues (e.g., diabetes, hypertension) before other symptoms appear.

What a comprehensive eye exam checks

Each exam typically includes evaluation of:

Visual acuity (vision test)

Peripheral vision (visual fields)

Eye pressure (glaucoma screening)

Lens clarity (cataracts)

Retina and optic nerve health (via dilation)

Eye alignment and movement

These tests detect problems long before symptoms appear.

Bottom line

Even if your vision feels perfect, routine eye exams are still important. They protect you from silent, progressive eye diseases and help maintain long-term vision health.

**model: DeepSeek**

*answer:*

Yes, you still need regular eye exams even if you have no obvious vision symptoms. Many serious eye conditions-like glaucoma, diabetic retinopathy, macular degeneration, and even some systemic diseases (e.g., hypertension, diabetes)-develop silently and cause irreversible damage before any symptoms appear. A comprehensive exam can detect these early, when treatment is most effective. Additionally, prescriptions for glasses or contacts can change subtly; an exam ensures optimal visual comfort and safety, especially for driving or screen work. Most adults should have a baseline exam at age 40, or earlier if risk factors exist, with frequency determined by your eye care professional.

**model: Doubao**

*answer:*

Yes, you still need regular eye exams even if you have no vision problems or symptoms at all.

Key reasons

Silent eye diseasesMany serious eye conditions (glaucoma, diabetic retinopathy, early macular degeneration, dry eye syndrome) develop without obvious early symptoms. By the time you notice blurriness or discomfort, permanent damage may already exist.

Systemic health screeningAn eye exam can detect underlying systemic issues: diabetes, high blood pressure, high cholesterol, thyroid disease, and even some neurological problems-often before other body symptoms appear.

Prescription & eye structure changesRefractive errors (nearsightedness, astigmatism) can change slowly without you noticing. Routine checks catch subtle shifts early.

Baseline recordA regular eye exam creates a baseline for your eye health, helping doctors spot future changes more quickly.

Recommended frequency

Adults under 40: Every 2-3 years

Ages 40-65: Every 1-2 years

Over 65, diabetes, family history of eye disease: Annual eye exams

**model: Gemini**

*answer:*

It is a common misconception that clear vision equals healthy eyes. Many serious eye conditions are "silent," meaning they develop without noticeable symptoms until significant damage has already occurred.

Regular eye exams are essential for preventive health for several key reasons:

1. Early Detection of Silent Conditions

Several eye diseases do not cause pain or blurred vision in their early stages. By the time you notice a change, the condition may be advanced:

Glaucoma: Often called the "silent thief of sight," it involves increased internal eye pressure that damages the optic nerve. It usually starts with peripheral vision loss that is nearly impossible to notice on your own.

Retinal Tears or Thinning: These can exist without symptoms but may lead to a retinal detachment if not monitored.

Chronic Health Markers: An eye exam allows a doctor to view blood vessels and nerves directly. This can reveal early signs of systemic issues like high blood pressure or diabetes before they show up in a standard physical.

2. Monitoring Structural Changes

As we age, the internal structures of the eye naturally change. For example, the crystalline lens slowly loses flexibility (presbyopia), and the macula can begin to thin. Establishing a "baseline" exam while your vision is good allows your optometrist or ophthalmologist to track these minute changes over the years and intervene if the rate of change becomes abnormal.

3. Preventive Management

If you spend significant time on digital screens or performing "near-work," an exam can identify accommodative stress or dry eye syndrome. Managing these early can prevent the headaches and eye strain that many people mistake for normal tiredness.

Recommended Frequency

General guidelines for adults with no symptoms or known risk factors:

Age Group; Recommended Frequency

18 to 39; At least every 2 years

40 to 64; Every 1 to 2 years (risk for glaucoma and presbyopia increases)

65 and older; Annually

Note: If you have a family history of eye disease or underlying health conditions, your doctor may recommend more frequent visits.

**model: Perplexity**

*answer:*

Yes - even without symptoms, it's still a good idea to get a routine eye exam because many eye diseases can develop quietly and be found early during an exam. For most adults, the timing depends on age and risk factors, but regular exams are commonly recommended even when your vision feels fine.

When it matters more

You should be especially proactive if you:

Are over 40.

Have diabetes, high blood pressure, or a family history of eye disease.

Wear glasses or contacts.

Notice any sudden changes, pain, flashes, floaters, or redness.

Why symptoms aren't enough

Some serious problems, including glaucoma, may not cause noticeable symptoms until damage has already started. That's why an eye exam can be useful even when you think everything is normal.

Practical takeaway

If you're a healthy adult with no symptoms, a routine comprehensive eye exam is still worthwhile, and for many people it's done every 1-2 years depending on age and risk.

**Q21. What should I do if my eye screening result says I need a referral?**

**model: ChatGPT**

*answer:*

If your screening result indicates that you need a referral, here's what you should do:

1. Schedule a Specialist Appointment

1. Usually, a referral means that your primary eye screening detected something that needs a closer evaluation. For example, with diabetes, this could indicate possible diabetic retinopathy or another eye condition.

2. You should book an appointment with an ophthalmologist (eye doctor who can perform a detailed exam), rather than just a general optometrist.

2. Bring Your Screening Results

1. Take the exact report or printout from your screening. This helps the specialist understand what was detected and guides further testing.

3. Follow Urgency Instructions

1. Some referrals are urgent (e.g., severe retinopathy), while others can wait a few weeks. Your screening report or the screening center should indicate this.

2. If you're unsure about the urgency, call the specialist and explain your results-they can advise on how soon you should be seen.

4. Prepare Your Medical History

1. Include information about chronic conditions (like diabetes or hypertension), medications, and previous eye problems.

2. This helps the specialist tailor the assessment and management plan.

5. Follow Through With Recommended Tests or Treatment

1. The specialist may perform additional exams like a dilated eye exam, OCT (Optical Coherence Tomography), or fundus photography.

2. Depending on findings, treatment could involve medication, laser therapy, or closer monitoring.

Key Point: A referral is a precaution to ensure that any potential eye problem is evaluated and managed early. Don't ignore it, even if your vision feels normal.

**model: Copilot**

*answer:*

If your screening result says "needs referral," the most important next step is to contact a healthcare provider promptly. This result usually means the screening found something unclear, abnormal, or needing a specialist's evaluation - not that you definitely have a condition.

Here's the clearest way to move forward:

What "needs referral" means (in plain language)

A referral flag means the screening detected something that requires a specialist or follow-up diagnostic test. Screenings are designed to be sensitive - they often flag things just to be safe.

Common reasons include:

The result was outside the normal range

The test couldn't determine a clear result

A possible risk was detected that needs expert evaluation

A specialist is required to confirm or rule out a condition

This aligns with guidance from the American Cancer Society, which notes that when screening results show possible signs of disease, a doctor will recommend next steps and refer you to a specialist.

What to do next (simple, ordered steps)

01

Contact your primary doctor

Share the screening result so they can interpret what the referral flag means for your specific situation.

02

Schedule a follow-up appointment

Your doctor may want to repeat the test, perform a physical exam, or order additional diagnostics.

03

Get the specialist referral

If needed, your doctor will refer you to the appropriate specialist based on the type of screening and result.

04

Attend the specialist evaluation

The specialist will perform more precise tests to confirm or rule out any condition.

05

Follow the recommended plan

Based on the specialist's findings, follow any treatment, monitoring, or lifestyle recommendations.

**model: DeepSeek**

*answer:*

If your screening result says you need a referral, here's what you should do:

Don't panic. Screening results are not diagnoses - they just indicate that further evaluation by a professional is needed to rule out or confirm any issues.

Contact your primary care provider (PCP) or the screening service. Ask for clarification about the reason for referral and which specialist or department you should see (e.g., cardiology, oncology, mental health, etc.).

Obtain the referral documents. Some systems require a formal referral letter; others may send your records directly to the specialist.

Schedule an appointment with the recommended specialist. Prepare to share your screening results, medical history, and any symptoms you've noticed.

Check insurance coverage. Confirm whether the referral needs pre-approval to avoid unexpected costs.

Follow up. If there's a long wait for the specialist's appointment, ask your PCP if any immediate steps (e.g., repeat screening, basic tests) are needed in the meantime.

Most referrals turn out to be precautionary, and many people receive normal or benign findings after complete evaluation. Taking action promptly is the best way to reduce uncertainty and anxiety.

**model: Doubao**

*answer:*

Understand what a referral meansIt means the screening found abnormal or concerning signs that need further check-up with a specialist (e.g., ophthalmologist, endocrinologist). It's not a final diagnosis, just a sign you need more detailed testing.

Contact your primary doctor firstBook an appointment with your regular doctor to review the screening report. They will explain the findings clearly and write a formal referral for the right specialist.

Make an appointment with the specialistSee the referred doctor as soon as possible-don't delay. Early follow-up can catch issues before they get worse.

Bring all your documentsTake your full screening report, lab results, medical history list, and any current medications to the specialist visit.

Complete further tests if advisedThe specialist may order extra exams (blood work, imaging, eye exams etc.) to confirm a diagnosis or rule out serious problems.

Follow the care planIf a condition is found, stick to the doctor's treatment, lifestyle advice, and follow-up schedule.

**model: Gemini**

*answer:*

Receiving a referral after a screening can feel a bit daunting, but it's actually a positive step toward clarity. It simply means the initial test identified something that requires a more specialized look or a definitive diagnosis.

Here is a step-by-step guide on how to navigate the process:

1. Clarify the "Why"

Before you leave the clinic or end the call, ensure you understand the specific reason for the referral. Ask the provider:

"What exactly was found during the screening that triggered this?"

"Is this a routine follow-up, or is there an urgent concern?"

"Which specific type of specialist do I need to see?"

2. Gather Your Documentation

Having your paperwork in order will prevent delays. Make sure you have:

The Screening Report: A copy of the actual results or images.

The Referral Form: Some insurance plans and specialists require a formal written referral from your primary provider.

Medical History: Note any symptoms you've noticed, even if they seem minor.

3. Schedule the Appointment Promptly

Don't wait for the specialist's office to call you.

Verify Insurance: Contact your insurance provider to ensure the specialist is in-network.

Ask about Wait Times: If the specialist has a long waiting list, ask to be put on a cancellation list or check if your primary doctor can help expedite the appointment if the matter is time-sensitive.

4. Prepare for the Specialist Visit

A specialist will likely perform more targeted tests. To make the most of the visit:

Bring a List of Questions: Ask about the accuracy of the screening, the next diagnostic steps, and potential treatment options.

Request Records Transfer: Ensure the specialist has access to your screening results before you arrive so you don't have to repeat the same tests unnecessarily.

Suggested Next Steps

While waiting for your specialist appointment, it can be helpful to stay informed without spiraling into "worst-case scenario" internet searches.

Focus on Lifestyle: Maintain your usual health routines (sleep, nutrition, activity) to keep your stress levels manageable.

Avoid Self-Diagnosis: Remember that a screening is a filter, not a diagnosis. Many referrals result in a "false positive" or a finding that is easily managed.

What was the general nature of the screening-was it for vision, a routine physical, or something else? Knowing the area of focus can help me give you more specific advice on what to expect from a specialist.

**model: Perplexity**

*answer:*

If your eye‐screening result says you "need a referral," the main thing to do is follow up with a full eye exam by an eye‐care professional (often an optometrist or ophthalmologist) as soon as possible. Ignoring a referral can delay treatment for conditions that might otherwise affect your vision long‐term.

What a referral usually means

A screening is a quick check to flag possible problems; a referral means they found something that needs a more detailed, in‐person exam. Common reasons include possible blurry vision, eye misalignment, or signs of conditions like glaucoma, cataracts, or retinal issues.

Immediate steps to take

Book an appointment with an eye‐care provider (optometrist or ophthalmologist) within the timeframe suggested on the screening slip or by the screener.

Take your referral/results sheet with you and ask the doctor to explain: what they found, how urgent it is, and what tests or treatments you might need.

If you have sudden vision loss, severe eye pain, double vision, or trauma, go to urgent care or an emergency department right away; some urgent eye problems need same‐day evaluation.

Insurance and paperwork

Check whether your insurance requires a referral from a primary‐care doctor or if you can book directly with an eye doctor; many plans let you see an optometrist without a referral.

If your insurance does require a referral form, ask the screening site or your primary‐care doctor to send it promptly so your appointment is not delayed

**Q22. If I already have mild diabetic retinopathy, how often should I have follow-up eye checks?**

**model: ChatGPT**

*answer:*

If you already have mild non-proliferative diabetic retinopathy, most ophthalmology and diabetes guidelines recommend:

Follow-up eye exams every 6-12 months.

6 months is often preferred if there are additional risk factors such as poor blood sugar control, high blood pressure, or rapidly worsening vision.

12 months may be acceptable if your retinopathy is stable and your diabetes is well-controlled.

The goal is to monitor for progression toward moderate or severe retinopathy, which would require more frequent monitoring or treatment.

Good control of blood sugar, blood pressure, and cholesterol can slow progression and may influence the exact timing of follow-ups.

**model: Copilot**

*answer:*

You should generally have a dilated eye exam every 12 months for mild non-proliferative diabetic retinopathy (mild NPDR) - but some situations call for more frequent follow-up.

Most authoritative guidance (AAO): People with mild NPDR and no macular edema are typically followed every 12 months.

What this means for you

Mild NPDR is the earliest stage of diabetic retinopathy. At this stage, the risk of rapid progression is lower than in moderate or severe disease, so annual monitoring is usually considered safe.

However, follow-up may be sooner than 12 months if:

Macular edema is present (often 4-6 months)

You have poorly controlled blood sugar, hypertension, or kidney disease

You are pregnant (diabetic retinopathy can worsen more quickly during pregnancy)

Your eye doctor sees changes that suggest faster progression

Typical follow-up intervals by severity

(From AAO management recommendations)

Severity; Typical Follow-up Interval

No retinopathy; 12 months

Mild NPDR; 12 months

Moderate NPDR; 6-12 months

Severe NPDR; 4 months

Proliferative DR; 4 months or more frequently depending on treatment

What you should do next

Because progression varies from person to person, the safest step is to confirm your individual follow-up schedule with your ophthalmologist. They can tailor the interval based on your exam findings and overall diabetes control.

**model: DeepSeek**

*answer:*

For mild non-proliferative diabetic retinopathy (NPDR), most major guidelines recommend a follow-up eye exam every 6 to 12 months. However, the exact schedule depends on your individual health and stability.

To help you understand the nuances in the guidelines, here is a detailed breakdown of the recommended timelines from various expert groups:

Follow-up Frequency by Guideline

Guideline Source; Recommended Frequency for Mild NPDR; Key Considerations

UK (NICE); Every 6-12 months; Standard recommendation for moderate non-proliferative retinopathy.

US (ADA/BCBS); At least annually; The American Diabetes Association recommends a dilated exam every year for patients with any level of retinopathy.

Denmark (Evidence-based); Every 12-24 months; The interval can potentially be extended to 2 years, provided your blood sugar (HbA1c <= 53 mmol/mol) and blood pressure (<=130/80 mmHg) are very well-controlled.

China; Every 6 months; A more frequent schedule, emphasizing an exam every 6 months for moderate NPDR.

Why Follow-up Schedules Vary

The variation in timelines generally comes down to balancing two things:

Your Risk of Progression: If your blood sugar, blood pressure, or cholesterol is poorly managed, an eye specialist will likely recommend the shorter end of the range (e.g., every 6 months) to watch for changes.

Risk of Rapid Changes: Certain situations require much closer monitoring, specifically pregnancy. If you are pregnant, guidelines recommend examinations every trimester due to the risk of rapid progression.

What You Can Do Right Now

Regular eye checks are crucial because mild diabetic retinopathy often has no symptoms, but they allow your doctor to monitor for worsening. You can also actively slow the progression by keeping your blood sugar, blood pressure, and cholesterol under control.

It is important to note that the frequency should be personalized for you. For the most appropriate schedule, please consult the ophthalmologist who manages your diabetic eye care.

I hope this helps you have a more informed discussion with your doctor.

**model: Doubao**

*answer:*

For mild non-proliferative diabetic retinopathy (NPDR) without macular edema:

Standard guideline recommendation: Dilated eye exam every 6-12 months.

If you have any of these risk factors: poor blood sugar control, high blood pressure, high cholesterol, or rapid blood sugar swings, follow up every 6 months is preferred.

If mild retinopathy progresses or develops diabetic macular edema, you'll need closer monitoring: every 3-6 months, plus possible retinal imaging (OCT, fundus photography).

**model: Gemini**

*answer:*

For individuals with mild diabetic retinopathy (specifically Mild Non-Proliferative Diabetic Retinopathy, or NPDR), the general consensus among major ophthalmology organizations-including the AAO and ADA-is a follow-up interval of 12 months.

However, this timeline can shift based on specific health factors. Here is the breakdown of the current clinical guidelines for 2026:

Recommended Follow-Up Intervals

Condition; Typical Follow-Up; Notes

Mild NPDR (Stable); 12 months; Standard for most patients with good glycemic control.

Mild NPDR + Pregnancy; Every 3 to 12 months; Pregnancy can accelerate retinopathy; monitoring often continues through 1-year postpartum.

Mild NPDR + Macular Edema; Every 4 to 6 months; If fluid is detected in the macula (even if vision feels fine), checks become much more frequent.

Mild NPDR + Poor Control; 6 to 12 months; Higher HbA1c or high blood pressure may prompt your doctor to see you sooner.

Why "Mild" Matters

At the mild stage, you likely have small "microaneurysms" (tiny bulges in the blood vessels). While these don't usually affect your vision immediately, the goal of the 12-month check is to ensure you haven't progressed to "Moderate" or "Severe" stages, where the risk of vision loss increases significantly.

When to Call the Doctor Sooner

Guidelines are "ideal world" scenarios. You should schedule an immediate appointment if you notice:

New "floaters" (spots, strings, or cobwebs in your vision).

Blurriness that doesn't go away or fluctuates throughout the day.

Dark or empty spots in your central vision.

Difficulty seeing colors or a perceived "faded" look to your surroundings.

Optimization for 2026

Many clinics now utilize AI-assisted retinal screening. If your clinic uses this technology, they may be able to provide a more precise risk assessment during your check-up to determine if you can safely stick to the 12-month window or if you need to come in at the 6-month mark.

**model: Perplexity**

*answer:*

For mild diabetic retinopathy, follow-up eye exams are typically recommended every 6 to 12 months.

Key Guidelines

Guidelines from sources like the International Council of Ophthalmology and American Diabetes Association suggest re-examination in 1-2 years for no apparent diabetic retinopathy or mild nonproliferative DR without diabetic macular edema (DME). However, if mild retinopathy is confirmed, many eye specialists advise checks every 6-12 months to monitor progression, especially if blood sugar control is poor or other risk factors exist.

Factors Influencing Frequency

Your doctor may recommend more frequent visits (e.g., every 3-6 months) based on severity, vision changes, or comorbidities like high blood pressure. Always consult your ophthalmologist for a personalized schedule, as individual cases vary.

**Q23. If I have moderate or severe diabetic retinopathy, how often should I have follow-up eye checks?**

**model: ChatGPT**

*answer:*

For people with moderate or severe diabetic retinopathy, follow-up eye exams need to be more frequent than for mild or no retinopathy. General guidelines recommend:

Moderate non-proliferative diabetic retinopathy (NPDR): Every 3-6 months

Severe NPDR or proliferative diabetic retinopathy (PDR): Every 1-3 months, depending on disease activity and treatment status

The exact interval can vary based on:

Whether the patient has diabetic macular edema

If they are receiving treatment (like laser, anti-VEGF injections, or surgery)

Overall glycemic and blood pressure control

Ophthalmologist's clinical judgment and progression risk

In practice, a moderate retinopathy patient without macular edema may be seen every 4-6 months, whereas severe cases may require monthly monitoring until disease stabilizes.

Regular monitoring is crucial because progression from moderate to proliferative stages can lead to significant vision loss if untreated.

**model: Copilot**

*answer:*

You should have much more frequent follow-up exams once diabetic retinopathy reaches moderate or severe stages, because the risk of progression to vision-threatening disease rises sharply.

Short answer

Moderate non-proliferative diabetic retinopathy (NPDR): every 6-12 months

Severe NPDR: every 3-4 months

Proliferative diabetic retinopathy (PDR): every 3-4 months, often monthly if receiving treatment (e.g., anti-VEGF injections)

These intervals come from the American Academy of Ophthalmology's Preferred Practice Pattern, which outlines follow-up timing based on disease severity.

What the evidence shows

Moderate NPDR: AAO guidance recommends 6-12-month follow-up depending on macular edema status.

Severe NPDR: Follow-up every 2-4 months is recommended because this stage has a high likelihood of progressing to proliferative disease.

PDR: Requires urgent referral and close monitoring-typically every 3-4 months, and sometimes monthly if undergoing anti-VEGF therapy.

These intervals are consistent with international clinical guidance as well.

Why the interval shortens

As retinopathy becomes more severe, the retina becomes more fragile and more likely to develop:

New abnormal blood vessels

Vitreous hemorrhage

Macular edema

Retinal detachment

Early detection of these changes is critical because timely treatment can prevent severe vision loss, even though it cannot restore lost vision.

If you're receiving treatment

If you are getting anti-VEGF injections, the AAO notes that follow-up may be as soon as 1 month after each injection.

‍ A gentle reminder

Because this involves your vision, it's important to stay in close contact with your ophthalmologist. They may adjust your follow-up schedule based on:

Macular edema

Pregnancy

Blood pressure or kidney issues

How quickly your retinopathy has progressed in the past

**model: DeepSeek**

*answer:*

For moderate to severe diabetic retinopathy (DR), eye examination frequencies generally fall into the following ranges based on current clinical guidelines:

Moderate Non-Proliferative DR (NPDR): Every 6 to 12 months. Some guidelines suggest that if your blood sugar (HbA1c) and blood pressure are very well controlled, an interval of up to 12-24 months might be acceptable, but this requires your specialist's approval.

Severe NPDR: Every 3 to 6 months. Because the risk of progressing to a vision-threatening stage is higher, more frequent monitoring is necessary.

Proliferative DR (PDR): Every 3 to 6 months initially (sometimes monthly if active), extending to 6-12 months once the condition has stabilized after treatment.

Additional Factors Affecting Frequency

Your specific situation may require adjustments to these intervals based on a few key factors:

Macular Edema (DME): If you have Diabetic Macular Edema (swelling in the retina), examinations are generally required every 3 to 6 months, or even more frequently if treatment is ongoing.

Glycemic Control: Poor blood sugar control significantly increases the risk of rapid disease progression, often requiring visits at the shorter end of the recommended range (e.g., every 3 months for severe NPDR).

During Pregnancy: If you have pre-existing diabetes and are pregnant, you should have an eye exam in the first trimester. You will likely require monitoring every trimester and for one year postpartum due to the risk of rapid progression.

Understanding the Differences

The variation in recommendations comes from how different organizations weigh risk. Major groups like the American Diabetes Association (ADA) suggest a minimum of annually for any retinopathy. However, more detailed guidelines (like the UK's NICE guidelines or Australian RACGP guidelines) advise that those with severe NPDR need to be seen as often as every 3 months, while moderate NPDR is safely monitored every 6 to 12 months.

I hope this gives you a clear picture of the recommended schedules. Remember, you should always follow the specific plan your ophthalmologist sets for you, as they will tailor the timing based on your exact diagnosis and how stable your condition is.

**model: Doubao**

*answer:*

Follow-up Eye Check Frequency for Moderate & Severe Diabetic Retinopathy (DR)

1. Moderate Non-Proliferative Diabetic Retinopathy (NPDR)

Recommended interval: Every 3-6 monthsMore frequent (3 months) if accompanied by poor blood sugar/blood pressure control or rapid lesion progression.

2. Severe NPDR & Proliferative Diabetic Retinopathy (PDR)

Recommended interval: Every 1-3 monthsMandatory close monitoring due to high risk of vitreous hemorrhage, retinal detachment, and vision loss.

3. With Diabetic Macular Edema (DME) (any severity)

Active DME: Every 1-2 months during treatment/follow-up

Stable DME: Every 3 months

Guideline Reference

International DR guidelines (AAO, ETDRS) and Chinese diabetic ophthalmology guidelines consistently recommend the above intervals for moderate/severe DR to enable timely intervention and prevent irreversible blindness.

**model: Gemini**

*answer:*

The frequency of follow-up exams for diabetic retinopathy (DR) depends heavily on the severity of the disease and the presence of any swelling in the macula (diabetic macular edema or DME). Because moderate and severe stages represent a significant risk for progression to vision-threatening complications, monitoring becomes much more frequent than the standard annual screening.

Based on current clinical guidelines (including the AAO 2026 and NICE standards), here are the typical follow-up intervals:

Recommended Follow-up Intervals

Stage of Retinopathy; Standard Follow-up Frequency

Moderate NPDR; Every 6 to 12 months (may be as frequent as 3-6 months if high-risk factors like poor blood sugar control or DME are present).

Severe NPDR; Every 3 to 4 months (close monitoring is critical as there is a high risk of progressing to proliferative disease within one year).

With Macular Edema; Follow-up is usually required every 1 to 3 months, depending on the treatment plan (e.g., injections or laser).

Key Factors That Influence Frequency

Your eye specialist may adjust these timelines based on several individual health factors:

Systemic Control: If your blood sugar ($HbA1c$), blood pressure, or renal function are not well-controlled, your doctor will likely want to see you more often, as these factors accelerate retinal damage.

Progression Rate: If your retinopathy has moved quickly from "mild" to "moderate," your specialist will shorten the interval to catch further changes early.

Pregnancy: Diabetes can progress very rapidly during pregnancy. Typically, exams are recommended once per trimester or even monthly depending on the baseline severity.

When to Seek Immediate Care

Regardless of your scheduled follow-up, you should contact your ophthalmologist immediately if you experience:

Sudden blurred, hazy, or "wavy" vision.

A sudden increase in "floaters" (spots, webs, or tiny threads in your vision).

A "curtain" or shadow over your field of vision.

Significant difficulty seeing at night or in low light.

Note: At the Severe NPDR stage, some specialists may discuss "proactive" treatment (like anti-VEGF injections) even before vision is lost, as the risk of progressing to the more dangerous Proliferative (PDR) stage can be as high as 50% to 75% over five years.

**model: Perplexity**

*answer:*

For moderate or severe diabetic retinopathy, follow-up eye exams are typically recommended more frequently than the standard annual check for diabetes patients without advanced issues.

Recommended Frequency

Guidelines from major eye health organizations, such as the American Academy of Ophthalmology, suggest dilated retinal exams at least every 6 to 12 months for moderate nonproliferative retinopathy, with closer monitoring (e.g., every 3-6 months) often needed for severe cases or if treatment like laser therapy or injections is underway. Your eye doctor tailors this based on disease progression, blood sugar control, and symptoms.

Why More Often

Progression to vision-threatening stages like proliferative retinopathy can happen quickly, so frequent checks allow early intervention. Poorly controlled diabetes or existing vision changes may prompt even shorter intervals.

Key Factors

Severity matters: Moderate may stay at 6-12 months; severe often requires quarterly or more.

Personalized plan: Always follow your ophthalmologist's advice, as they assess your specific risk.

Consult promptly if symptoms like floaters or blurred vision worsen

**Q24. What symptoms can diabetic retinopathy cause?**

**model: ChatGPT**

*answer:*

Diabetic retinopathy often develops gradually and may not cause noticeable symptoms in the early stages. As it progresses, it can lead to several visual changes, including:

1. Blurred or fluctuating vision - Vision may become blurry or change from day to day.

2. Dark or empty areas in vision - Spots or gaps in your visual field can appear due to damaged retinal blood vessels.

3. Floaters - Small dark spots, lines, or cobweb-like shapes drifting in your vision.

4. Difficulty seeing at night - Night vision can become impaired.

5. Color vision changes - Colors may appear faded or washed out.

6. Sudden vision loss - In severe cases, bleeding in the eye or retinal detachment can cause sudden loss of vision.

It's important to note that early diabetic retinopathy may be completely asymptomatic, which is why regular eye exams are crucial for anyone with diabetes. Catching changes early allows for interventions like laser therapy, anti-VEGF injections, or better blood sugar management to prevent vision loss.

**model: Copilot**

*answer:*

Diabetic retinopathy can cause no symptoms at first, but as it progresses, it can lead to several vision-related changes. These symptoms occur because high blood sugar damages the small blood vessels in the retina, leading to leakage, swelling, or abnormal new vessel growth.

Key symptoms you may notice

Blurred vision - often one of the earliest signs as the retina or macula swells.

Floaters - dark spots or string-like shapes drifting in your vision, caused by small amounts of bleeding into the eye.

Fluctuating vision - vision may shift from clear to blurry as retinal swelling changes.

Dark or empty areas in your field of view - from retinal damage or blocked blood flow.

Poor night vision - difficulty seeing in dim light.

Colors appearing faded - due to macular involvement.

Sudden vision loss - can happen if there is significant bleeding (vitreous hemorrhage) or retinal detachment.

Why symptoms appear late

Diabetic retinopathy often progresses silently. Early stages may cause no noticeable changes, which is why regular dilated eye exams are essential even if vision seems normal.

When to seek urgent care

If you experience sudden vision changes, severe floaters, flashes of light, or a curtain-like shadow, seek immediate medical attention - these can signal retinal detachment or major bleeding.

**model: DeepSeek**

*answer:*

Diabetic retinopathy often causes no symptoms in its early stages, which is why regular eye exams are critical. As the condition progresses, possible symptoms include:

Blurred or fluctuating vision

Increased number of floaters (spots, dots, or cobweb-like shapes drifting in your field of vision)

Dark or empty areas in your vision

Poor night vision

Difficulty with color perception (colors appear faded or washed out)

Vision loss, ranging from partial to complete blindness

If abnormal new blood vessels grow on the retina (proliferative diabetic retinopathy), symptoms may also include sudden, severe vision loss due to vitreous hemorrhage (bleeding into the gel-like fluid inside the eye). Any sudden changes in vision warrant immediate medical attention.

**model: Doubao**

*answer:*

Symptoms often develop gradually, and many people have no early symptoms even with retinal damage.

Early-stage

No obvious visual discomfort

Mild blurry vision (intermittent)

Fluctuating eyesight (worse when blood sugar is poorly controlled)

Middle & Late-stage

Persistent blurred or distorted vision

Floaters: Dark spots, specks or cobwebs drifting in the visual field

Flashes of light in peripheral vision

Difficulty reading or seeing distant objects

Loss of peripheral vision

Severe / Advanced (Proliferative DR)

Sudden painless vision loss

Large dark shadows blocking vision

Retinal detachment risk leading to permanent blindness

Macular edema causing central vision loss and inability to focus clearly

**model: Gemini**

*answer:*

Diabetic retinopathy is a condition resulting from damage to the blood vessels of the light-sensitive tissue at the back of the eye (retina). In its early stages, the condition may cause no symptoms or only mild vision problems, but it can eventually lead to blindness.

The symptoms generally fall into several categories depending on the severity and progression of the disease:

Common Symptoms

Floaters: Seeing spots or dark strings floating in your vision. These are often caused by tiny amounts of blood leaking into the vitreous (the gel-like substance in the eye).

Blurred Vision: Vision may become blurry or hazy, making it difficult to focus on objects.

Fluctuating Vision: Your ability to see clearly may change throughout the day, sometimes linked to blood sugar levels.

Impaired Color Vision: Colors may appear faded, washed out, or less vibrant than usual.

Dark or Empty Areas: You may notice "blind spots" or dark patches in your field of vision where certain details are missing.

Vision Loss: In advanced stages, significant and permanent vision loss can occur if the condition is left untreated.

Progression and Complications

Symptoms often escalate as the disease progresses through two main phases:

1. Early (Nonproliferative): Often asymptomatic. However, if fluid leaks into the center of the retina (Macular Edema), central vision becomes blurry.

2. Advanced (Proliferative): New, fragile blood vessels grow in the retina. If these leak into the center of the eye, they can cause a sudden and severe increase in floaters or a total "blackout" of vision.

3. Note: Because early-stage diabetic retinopathy often has no warning signs, regular eye exams with pupil dilation are the most effective way to detect changes before permanent damage occurs.

**model: Perplexity**

*answer:*

Diabetic retinopathy often starts without noticeable symptoms, especially in early stages. As it progresses, common vision changes emerge due to damaged retinal blood vessels.

Early Symptoms

Mild or no symptoms occur initially, but subtle signs like fluctuating vision or minor blurriness may appear from blood sugar effects on the eye's lens.

Progressive Symptoms

Blurred or distorted vision from fluid leakage or macular edema.

Floaters (spots, dark strings, or streaks) or flashes of light.

Dark, empty, or blind spots (scotomas) in vision.

Difficulty seeing at night (night blindness) or in low light.

Advanced Symptoms

Color vision changes, like faded colors or new color blindness.

Vision loss, including peripheral field defects or total sight reduction.

Eye pain or pressure in severe cases from bleeding or complications

**Q25. Can diabetic retinopathy have no symptoms in the early stage?**

**model: ChatGPT**

*answer:*

Yes - diabetic retinopathy can have no symptoms in the early stages. In fact, many people with mild or even moderate diabetic retinopathy may not notice any changes in their vision at all. This is why regular eye exams are crucial for people with diabetes, even if vision seems normal. Early detection allows treatment before significant vision loss occurs.

Symptoms usually appear only once the disease progresses, such as:

Blurred or fluctuating vision

Dark spots or floaters

Difficulty seeing at night

Loss of vision in severe cases

Because early stages are often silent, ophthalmologists recommend annual dilated eye exams for most people with diabetes. If retinopathy is detected, the frequency of follow-ups may increase depending on severity.

**model: Copilot**

*answer:*

Yes - diabetic retinopathy can absolutely have no symptoms in its early stage, and this is one of the reasons it is so dangerous.

Key takeaway

Early diabetic retinopathy is often completely silent, with no pain, no blurriness, and no noticeable vision changes. This is confirmed by major medical authorities including the Mayo Clinic, the American Academy of Ophthalmology, and the National Eye Institute.

Why early diabetic retinopathy has no symptoms

Damage begins in the tiny blood vessels of the retina, but the retina has no pain receptors.

Vision stays normal until swelling, bleeding, or vessel closure becomes significant.

By the time symptoms appear (floaters, blurry vision, dark spots), the disease is usually moderate or advanced.

What the early stage looks like (even if you feel fine)

Microaneurysms (tiny bulges in retinal vessels)

Small leaks of fluid or blood

Early macular swelling (may not affect vision yet)

No pain, no redness, no obvious warning signs

Why this matters

Because early diabetic retinopathy is silent, many people don't know they have it until vision is already threatened. That's why:

The National Eye Institute states that early diabetic retinopathy "may not have any symptoms at first."

The Mayo Clinic notes that "at first, diabetic retinopathy might cause no symptoms or only mild vision problems."

The American Academy of Ophthalmology says "You can have diabetic retinopathy and not know it... it often has no symptoms in its early stages."

What you should do if you have diabetes

Get a dilated eye exam every year - even if your vision seems perfect.

Control blood sugar, blood pressure, and cholesterol, which slows progression.

See an eye doctor immediately if you notice floaters, blurry vision, or dark spots.

**model: DeepSeek**

*answer:*

Yes, that is correct. Diabetic retinopathy often has no noticeable symptoms in its early stages (non-proliferative diabetic retinopathy).

During this phase, high blood sugar weakens the tiny blood vessels in the retina, causing them to develop microaneurysms (small bulges) and leak fluid or blood. However, these changes may not affect central vision, so a person may see perfectly well and have no pain or other warning signs.

This is why regular dilated eye exams are critical for people with diabetes-they allow an eye doctor to detect early damage before vision loss occurs. By the time symptoms like blurred vision, floaters, dark spots, or difficulty seeing at night appear, the disease may have already progressed to a more advanced (proliferative) stage or caused macular edema.

**model: Doubao**

*answer:*

Yes, diabetic retinopathy is typically completely asymptomatic in its early stages.

Key points

Early non-proliferative diabetic retinopathy often has no pain, no blurred vision, no obvious visual changes.

Microaneurysms, small retinal hemorrhages, and exudates can already exist in the retina without causing any noticeable symptoms.

Vision loss usually only appears when the condition progresses to macular edema or advanced proliferative retinopathy.

This is why regular annual eye screening is essential for all diabetic patients, even with normal vision.

**model: Gemini**

*answer:*

The short answer is yes. In fact, it is very common for diabetic retinopathy to have no symptoms at all during its early stages.

Because the initial damage often occurs in the tiny blood vessels of the retina (the light-sensitive tissue at the back of the eye), the changes are frequently invisible to the person affected. Vision often remains perfectly clear even as the condition begins to progress.

How It Progresses Silently

In the early stage, known as Non-Proliferative Diabetic Retinopathy (NPDR), the blood vessel walls in the retina weaken. This can lead to:

Microaneurysms: Tiny bulges in the blood vessels that may leak fluid.

Retinal Hemorrhages: Small spots of blood that don't necessarily obstruct the "center" of your vision.

Because these changes usually happen in the periphery or are too small to notice, you might not realize anything is wrong until the damage reaches the macula (the part of the eye responsible for sharp, central vision) or enters the advanced proliferative stage.

When Symptoms Finally Appear

By the time symptoms do occur, the condition has often reached a more concerning level. Warning signs can include:

An increase in "floaters" (spots or dark strings in your vision).

Blurred or fluctuating vision.

Dark or empty areas in your field of vision.

Difficulty seeing colors or poor night vision.

The Importance of Screening

Because of this "silent" phase, medical guidelines generally emphasize the following:

1. Annual Exams: People with diabetes are typically advised to have a comprehensive dilated eye exam at least once a year.

2. Early Detection: A specialist can see the early signs of leaking vessels or swelling long before you experience a change in sight.

3. Management: Keeping blood sugar, blood pressure, and cholesterol levels stable is the most effective way to slow or prevent the progression of the disease.

4. Early detection is the most effective way to protect your vision before permanent damage occurs.

**model: Perplexity**

*answer:*

Yes, diabetic retinopathy often has no symptoms in its early stages. This silent progression means damage can occur without noticeable vision changes, making regular eye exams essential for people with diabetes.

Early Stage Details

In the nonproliferative stage, blood vessels in the retina weaken and may leak, but patients typically experience no pain or obvious signs. Symptoms like floaters, blurred vision, or dark spots usually appear only as the disease advances to later stages.

Detection and Prevention

Ophthalmologists detect early changes through dilated eye exams, even before symptoms develop. Annual screenings are recommended for all diabetics, regardless of vision issues, to prevent irreversible damage

**Q26. What should I do if I suddenly see many floaters or dark spots?**

**model: ChatGPT**

*answer:*

Sudden appearance of many floaters or dark spots in your vision can be a warning sign of a serious eye problem, such as a retinal tear or retinal detachment. These conditions can lead to permanent vision loss if not treated promptly.

Here's what you should do immediately:

1. Do not wait - Even if your vision seems okay, these symptoms are considered an emergency.

2. Contact an eye specialist right away - Call your ophthalmologist or go to an emergency eye clinic. If unavailable, visit the nearest emergency room.

3. Avoid activities that could worsen the problem - Try not to strain your eyes, bend over, or lift heavy objects until you've been evaluated.

4. Note any additional symptoms - Flashing lights, a curtain-like shadow over your vision, or loss of peripheral vision are all urgent warning signs.

In short: sudden many floaters or dark spots = emergency eye evaluation. Early intervention can save your vision.

**model: Copilot**

*answer:*

A sudden surge of many new floaters or dark spots is a medical warning sign. The most important takeaway is that you should get an urgent, same-day eye exam from an ophthalmologist because this symptom can indicate a retinal tear or retinal detachment, which can threaten vision if not treated quickly.

What this symptom can mean

A sudden increase in floaters is strongly associated with conditions that require prompt evaluation:

Retinal tear - When the vitreous gel pulls on the retina hard enough to rip it. Mayo Clinic notes that a sudden increase in floaters-especially with flashes or vision loss-should prompt immediate evaluation.

Retinal detachment - Fluid gets under a retinal tear and lifts the retina like wallpaper. This can cause permanent vision loss if untreated.

Posterior vitreous detachment - Often benign, but can still cause tears during the separation process. Johns Hopkins emphasizes that new floaters require a dilated exam to rule out retinal damage.

Bleeding or inflammation - From trauma, diabetes, infection, or uveitis.

What you should do right now

Seek urgent evaluation by an ophthalmologist (eye MD) - ideally today. Duke Health stresses that a sudden increase in floaters should be treated as an urgent issue.

Go to urgent care or an emergency department if you cannot get a same-day ophthalmology appointment.

Do NOT wait to see if it goes away - symptoms alone cannot tell you whether a tear or detachment is present.

Red flags that make it even more urgent

If any of these accompany the floaters, you should seek emergency care immediately:

Flashes of light

A "curtain," shadow, or dark area moving across your vision

Sudden blurry vision

Loss of peripheral (side) vision

Eye pain or redness

History of high nearsightedness, diabetes, or recent eye trauma

These symptoms are specifically highlighted by Mayo Clinic and Johns Hopkins as signs of possible retinal tear or detachment.

Why fast action matters

If a retinal tear is caught early, ophthalmologists can often seal it with a quick in-office laser procedure. If it progresses to a detachment, surgery is usually required, and vision recovery becomes less certain.

**model: DeepSeek**

*answer:*

This is a situation where you should seek urgent medical attention from an eye doctor (optometrist or ophthalmologist) as soon as possible, ideally within 24 hours.

A sudden onset of many new floaters or dark spots can be a sign of a serious condition, such as a retinal tear or detachment. Without prompt treatment, these conditions can lead to permanent vision loss.

Why it is urgent

The vitreous gel inside your eye can shrink and pull away from the retina. If it pulls too hard, it can:

Tear the retina (creates new floaters, often described as a "cobweb" or "shower" of dots)

Cause bleeding into the vitreous (seen as many new dark spots)

Lead to retinal detachment (if fluid seeps through the tear)

What you should do immediately

Do not wait to see if it goes away. Floaters from a tear will not disappear on their own.

Call an eye clinic or emergency room that has an ophthalmologist on call. Tell them: "I suddenly have many new floaters/dark spots in my vision."

Avoid strenuous activity, heavy lifting, or jarring movements (like running or jumping) until you are examined, as these could worsen a retinal tear.

Other symptoms that make it more urgent

If you have any of these along with the floaters, go to an emergency room immediately:

Flashes of light (like a camera flash or lightning streaks, especially in peripheral vision)

A shadow or curtain coming across your vision (from any side)

Sudden decrease in vision or blurriness

Eye pain (though retinal tears often are painless)

What if it is not a tear?

Many people have benign floaters (especially with nearsightedness or aging). However, you cannot tell the difference without a dilated eye exam. Only a doctor looking at your retina can rule out a tear.

Bottom line

Do not rely on home remedies or "wait and see." A sudden increase in floaters is an eye emergency. Call a doctor now.

**model: Doubao**

*answer:*

Seek emergency eye care right awayGo to an ophthalmology clinic or emergency eye hospital within hours, do not wait or self-observe for days.

Do not delay, avoid eye strainStop using phones, reading, and strenuous exercise; avoid bending over heavily, lifting heavy objects.

Key possible serious causes

Vitreous detachment

Retinal tear / retinal detachment (vision-threatening)

Vitreous hemorrhage, diabetic retinopathy bleeding, uveitis

Must have a professional eye examThe doctor will do a dilated fundus examination + ocular ultrasound to check the retina.If there is a retinal hole or tear, timely laser treatment can prevent retinal detachment and permanent vision loss.

Never ignore these red flags

Sudden increase in floaters

Flashing lights especially in dark environments

A shadow covering part of the visual field

Peripheral vision loss

If you have these symptoms now, please see an eye doctor as soon as possible-it is an urgent eye condition that cannot be delayed.

**model: Gemini**

*answer:*

Seeing a sudden increase in floaters or dark spots can be alarming, and while they are often a normal part of aging, a sudden "shower" of them can be a sign of a serious condition.

Immediate Steps

If you are experiencing a sudden onset of many new floaters, you should contact an eye specialist (ophthalmologist or optometrist) immediately for a dilated eye exam. If it is after hours and you cannot reach a specialist, go to the nearest emergency room.

When It's an Emergency

Seek medical attention within 24 hours if you notice:

A sudden "shower" or swarm of many new floaters or tiny black spots.

Flashes of light (photopsias) in one or both eyes, similar to lightning streaks or camera flashes.

A dark curtain or shadow moving across your field of vision from any direction.

Sudden blurred vision or a loss of peripheral (side) vision.

Why Is This Urgent?

These symptoms can indicate that the vitreous gel inside your eye is pulling on the retina. In some cases, this can lead to:

1. Retinal Tear: A small break in the retina. This can often be treated quickly with a laser if caught early.

2. Retinal Detachment: If fluid leaks through a tear, it can lift the retina off the back of the eye. This is a medical emergency that can lead to permanent vision loss if not treated with surgery immediately.

What to Avoid

Do not wait to see if the spots go away on their own.

Avoid driving yourself if your vision is impaired or if your pupils will be dilated during the exam; have someone else drive you to the clinic or ER.

If the Symptoms are Mild or Old

If you have a few stable floaters that you have noticed for months or years and they aren't changing, these are usually harmless protein clumps in the vitreous. However, any new or sudden change in your vision always warrants a professional evaluation to rule out underlying issues.

**model: Perplexity**

*answer:*

Seek emergency eye care immediately. A sudden increase in floaters or dark spots can signal a retinal tear or detachment, which requires urgent evaluation to prevent vision loss.

Urgent Symptoms

These red-flag signs alongside many floaters demand same-day attention at an ER or ophthalmologist:

New flashing lights in one eye.

Shadow, curtain, or blank spot in your vision.

Sudden vision drop or peripheral loss.

Why It's Critical

Floaters often arise from vitreous changes with age, but a rapid shower of new ones may mean bleeding or retinal damage. Prompt dilation exam allows laser treatment if needed, with studies showing over 80% good outcomes for early cases.

Next Steps

Contact an eye specialist or go to the ER now-do not wait or drive if vision is impaired. Regular checkups monitor routine floaters, but sudden changes are never normal.

**Q27. What should I do if my vision suddenly becomes blurred or worse?**

**model: ChatGPT**

*answer:*

If your vision suddenly becomes blurred, worse, or changes in any unexpected way, you should treat it as an eye emergency. Sudden vision changes can indicate serious conditions, including:

Vitreous hemorrhage (bleeding inside the eye)

Retinal detachment

Acute glaucoma

Stroke affecting the eye (rare but serious)

Severe worsening of diabetic retinopathy

What you should do immediately:

1. Seek urgent medical attention - Go to an eye emergency clinic or emergency room immediately. Do not wait for your next scheduled eye exam.

2. Avoid driving yourself if your vision is impaired; have someone else drive you.

3. Note any other symptoms - flashes of light, floaters, eye pain, headache, or weakness on one side of the body. This information helps the doctor.

4. Do not try to self-treat with eye drops or medications unless prescribed.

Time is critical for conditions like retinal detachment-early treatment can prevent permanent vision loss.

**model: Copilot**

*answer:*

Sudden blurred or worsening vision is treated as a medical emergency. The safest immediate action is to seek urgent medical care right away, because several of the conditions associated with sudden vision changes can lead to permanent vision loss or signal a stroke.

Below is a clear, structured guide based on the evidence from the sources you saw.

Immediate takeaway

If your vision becomes suddenly blurred, dim, doubled, distorted, or partially lost, go to the emergency room immediately. Sudden vision change is linked to emergencies such as stroke, retinal detachment, acute glaucoma, or bleeding in the eye.

What to do right now

Stop what you're doing - do not drive yourself.

Have someone take you to the ER or call emergency services if you're alone.

Note any additional symptoms (headache, weakness, speech difficulty, flashing lights, floaters, eye pain). These help doctors identify the cause.

Avoid rubbing your eye - this can worsen injuries or retinal problems.

Remove contact lenses if you wear them, unless it causes pain.

When sudden blurred vision is an emergency

These conditions require immediate medical attention:

Stroke - sudden blurred vision, double vision, or loss of vision can be a stroke warning sign. Often accompanied by trouble speaking or facial droop.

Retinal detachment - flashes of light, new floaters, a curtain-like shadow, or sudden blur.

Acute angle-closure glaucoma - sudden blur with severe eye pain, halos around lights, nausea.

Eye bleeding or trauma - after being hit in the eye; causes pain, light sensitivity, and blurred vision.

TIA (mini-stroke) - temporary blurred vision lasting minutes to hours.

Infections like endophthalmitis or keratitis - redness, discharge, pain, and rapid vision decline.

These can cause permanent vision loss if not treated quickly.

When blurred vision is less urgent (but still needs care)

If the blurring is gradual or mild, common causes include:

Dry eye

Refractive errors (nearsightedness, farsightedness, astigmatism)

Eye strain

Medication side effects

These still require evaluation by an eye doctor, but they are not typically emergencies.

Why sudden vision changes are taken so seriously

Medical sources emphasize that sudden blurred vision is different from gradual changes:

It can indicate bleeding, inflammation, nerve damage, or loss of blood flow.

It may be the first sign of a stroke or retinal detachment.

Early treatment dramatically improves the chance of preserving vision.

Visual reference: conditions that can cause sudden blurred vision

If you're unsure whether it's an emergency

Use this rule:

If the vision change is sudden, new, or accompanied by pain, headache, or neurological symptoms to treat it as an emergency.

**model: DeepSeek**

*answer:*

If your vision suddenly becomes blurred or worsens, treat this as a medical emergency. Seek immediate professional help - do not wait to see if it improves on its own. Here's what to do:

Stop what you are doing - Especially if driving or operating machinery, pull over safely and stop.

Close your eyes briefly - This can help rule out temporary issues like dry eye or eye strain, but do not delay seeking care.

Do not rub your eyes - Rubbing could worsen certain conditions like retinal tears or acute angle-closure glaucoma.

Contact an emergency room or ophthalmologist immediately - Call for an ambulance if the vision loss is severe, sudden, or accompanied by other symptoms (e.g., severe headache, eye pain, nausea, flashing lights, or weakness on one side).

Possible serious causes include:

Retinal detachment

Acute glaucoma

Stroke or transient ischemic attack (TIA)

Optic neuritis

Corneal emergency (e.g., infection or injury)

Even if the blurriness comes and goes, or if you think it's "just a migraine," it is safest to get an urgent eye examination.

**model: Doubao**

*answer:*

If you have sudden blurred or worsening vision, follow these steps immediately:

1. Seek emergency medical help right away

Sudden vision change is a potential eye emergency.Go directly to an ophthalmology emergency department or nearest hospital within hours, do not wait to see if it gets better on its own.

2. Do not do these things

Don't rub your eyes

Don't self-medicate with eye drops

Don't drive yourself if your sight is poor

Avoid bright lights and screen use temporarily

3. Key possible serious causes

Sudden blurred vision can signal:

Retinal detachment

Acute glaucoma

Macular hemorrhage

Diabetic retinopathy bleeding

Optic nerve problems

Transient ischemic attack / stroke-related visual loss

4. What to tell the doctor

Be ready to describe:

How quickly vision changed (seconds / minutes / hours)

Any eye pain, headache, flashes of light, floaters, shadow in vision

History of diabetes, high blood pressure, high cholesterol

Any recent injury or stress

If it happens now, please get an eye emergency check as soon as possible-many sudden eye conditions need treatment within a short time to save vision.

**model: Gemini**

*answer:*

Sudden vision loss or blurring is a medical emergency until proven otherwise. Because certain conditions can lead to permanent vision loss within hours, you should take immediate action.

1. Seek Emergency Medical Care Immediately

If your vision change is sudden (occurring over seconds or minutes), go to an Emergency Room or an ophthalmologist immediately, especially if you experience:

"Curtain" or "Shadow": A dark shadow or curtain moving across your field of vision (potential retinal detachment).

Eye Pain: Severe pain within the eye, often accompanied by nausea (potential acute glaucoma).

Neurological Symptoms: Sudden blurring combined with facial drooping, arm weakness, or difficulty speaking (potential stroke).

Flashers and Floaters: A sudden increase in "cobwebs" or bright flashes of light.

Shutterstock

2. Common Underlying Causes

Medical professionals generally categorize sudden vision changes into a few high-priority areas:

Vascular Issues: A "stroke" of the eye (Central Retinal Artery Occlusion) happens when blood flow is blocked. This requires intervention within a very narrow window of time.

Acute Angle-Closure Glaucoma: A rapid spike in eye pressure. This usually causes intense pain and seeing "halos" around lights.

Retinal Detachment: When the light-sensitive tissue at the back of the eye pulls away from its underlying layer.

Vitreous Hemorrhage: Bleeding into the gel-like center of the eye, often seen in patients with advanced diabetes.

3. What to Do While Waiting for Care

Do Not Drive: Your depth perception and peripheral vision are compromised. Have someone else drive you or call emergency services.

Do Not Rub Your Eyes: If the cause is physical trauma or a retinal tear, rubbing can worsen the damage.

Note the Timing: Try to remember exactly when it started and if it is in one eye or both. This is the first question a doctor will ask.

Keep Your Stomach Empty: If you require emergency surgery, it is safer if you haven't eaten recently.

4. Diagnostic Steps to Expect

When you arrive at the clinic, the medical team will likely perform:

1. Visual Acuity Test: Checking how much detail you can see.

2. Slit-Lamp Examination: Using a high-intensity light and microscope to look at the structures of the eye.

3. Dilated Fundus Exam: Using drops to widen your pupils so they can inspect the retina and optic nerve.

4. Tonometry: Measuring the fluid pressure inside your eye.

**model: Perplexity**

*answer:*

Sudden blurred or worsening vision can be a sign of a serious eye or medical problem, so it should never be ignored, even if it seems to improve quickly.

When to get emergency help

Call emergency services or go to an emergency department right away if blurred vision is accompanied by any of these "red flag" symptoms:

Sudden loss of vision in one or both eyes

Double vision, "curtain" or shadow over part of your vision, flashes of light, or many new floaters

Severe eye pain, headache, nausea, or eye redness

Weakness or numbness on one side, slurred speech, facial droop, dizziness, or trouble walking (possible stroke or TIA)

These can signal a detached retina, stroke/TIA, acute glaucoma, or other serious conditions that need treatment within hours to preserve vision.

What to do immediately

Even if you don't have the red‐flag symptoms, take these steps:

Stop what you're doing (especially driving or using machinery) and sit down safely.

If you wear contact lenses, remove them and try blinking or using artificial‐tear drops.

Check for obvious causes such as eye strain, dryness, or, if you have diabetes, a recent high blood‐sugar reading.

Note whether the blurring affects one or both eyes, how long it lasts, and any other symptoms (headache, eye pain, floaters, etc.), because this helps your doctor narrow down the cause.

When to see a doctor soon (not an emergency)

Seek prompt non‐emergency care (same day or within 24 hours) if:

The blurring is new, persistent, or keeps coming back, even without pain.

You have a history of diabetes, high blood pressure, or eye disease.

Your regular glasses or contacts no longer correct your vision adequately.

Your next step should normally be an urgent visit to an eye doctor (ophthalmologist or optometrist) or your primary‐care clinician, especially in San Jose where you can choose from several nearby eye‐care centers

**Q28. Is eye pain a typical symptom of diabetic retinopathy?**

**model: ChatGPT**

*answer:*

No, eye pain is not a typical symptom of diabetic retinopathy, especially in the early stages. Diabetic retinopathy usually progresses silently and can be present without noticeable symptoms. Common early signs are:

Blurred or fluctuating vision

Dark spots or floaters

Difficulty seeing at night

Pain in the eye is more often associated with other eye conditions, such as:

Glaucoma (especially acute angle-closure glaucoma)

Eye infections or inflammation (e.g., uveitis, conjunctivitis)

Corneal abrasions or injuries

If someone with diabetes experiences sudden eye pain, especially accompanied by vision changes, it is considered an eye emergency and should prompt immediate evaluation by an eye specialist.

**model: Copilot**

*answer:*

Eye pain is not typically a symptom of diabetic retinopathy. The condition usually progresses silently, with most people experiencing no pain at all, especially in the early and middle stages. Symptoms-when they do appear-tend to involve changes in vision, not discomfort.

What symptoms are typical?

Common signs of diabetic retinopathy include:

Blurred vision

Floaters (dark spots or strings)

Fluctuating vision

Dark or empty areas in the visual field

Vision loss

These symptoms occur because high blood sugar damages retinal blood vessels, causing leakage, swelling, or abnormal new vessel growth.

So why might someone with diabetes have eye pain?

While diabetic retinopathy itself is usually painless, eye pain can occur from related complications, such as:

Neovascular glaucoma - new blood vessels block fluid drainage, raising eye pressure and causing pain

Severe vitreous hemorrhage - less common, but pressure or inflammation can cause discomfort

Corneal issues - diabetes increases risk of dry eye and infections, which can be painful

If someone with diabetes experiences eye pain, it's important to treat it as a red flag and get prompt evaluation.

When to seek care

You should contact an eye care professional right away if you have:

Sudden vision changes

Severe eye pain

New floaters or flashes

Partial or complete vision loss

These can signal urgent complications that require immediate attention.

**model: DeepSeek**

*answer:*

The short answer is no, eye pain is not a typical or common symptom of early or moderate diabetic retinopathy (DR). In fact, diabetic retinopathy is often called a "silent" disease because it can cause significant damage to the retina without any pain or noticeable vision changes.

However, there are specific, advanced, or complicated stages of diabetic eye disease where pain can occur. It's crucial to understand the difference, as pain often signals a different or more urgent problem.

Let's break it down.

Typical Symptoms of Diabetic Retinopathy (Usually Painless)

In its early stages (non-proliferative DR) and even into the proliferative stage, DR typically causes:

Blurred or fluctuating vision

Increased floaters (spots, dark strings, or cobwebs)

Dark or empty areas in your vision

Difficulty seeing at night

Impaired color perception

Vision loss (in advanced stages)

Again, none of these are typically painful.

When CAN Diabetic Retinopathy Cause Eye Pain?

Pain becomes a possibility in two main scenarios, both representing severe, late-stage complications:

1. Neovascular Glaucoma (Most Common Painful Cause)

This is the most direct and serious link between DR and eye pain.

What it is: In advanced Proliferative Diabetic Retinopathy (PDR), the retina becomes starved of oxygen. To compensate, the eye grows abnormal new blood vessels (neovascularization). These vessels can grow on the iris and in the eye's drainage angle (the trabecular meshwork).

Why it hurts: These new vessels block the fluid from draining out of the eye, causing a rapid and severe increase in intraocular pressure (IOP). This condition is a type of painful, "closed-angle" glaucoma.

Symptoms: Severe, deep, aching eye pain, often accompanied by headache, nausea, vomiting, redness of the eye, seeing halos around lights, and sudden vision loss. This is an eye emergency.

2. Vitreous Hemorrhage (Indirect Pain)

What it is: The same fragile new blood vessels from PDR can bleed into the vitreous gel (the clear jelly that fills the center of the eye).

Why it might hurt: The hemorrhage itself is usually painless. However, if it is very dense, it can lead to a rapid rise in eye pressure (hemorrhagic glaucoma), which can cause pain. Also, the process of the blood and debris moving within the eye can sometimes cause a feeling of pressure or fullness, though sharp pain is less common.

Other Diabetic Eye Problems That DEFINITELY Cause Pain

It's vital to know that eye pain in a person with diabetes is more likely caused by other diabetic eye conditions that are not retinopathy.

Severe Dry Eye: Diabetes damages the nerves that control tear production. This is very common and often feels like a scratchy, burning, gritty sensation.

Corneal Abrasions/Recurrent Erosions: Diabetes can weaken the cornea's surface (epithelium). It can fail to stick down properly, leading to sudden, sharp, tearing pain, especially upon waking.

Diabetic Corneal Neuropathy: High blood sugar can damage the nerves in the cornea, sometimes causing spontaneous, severe, "lightning bolt" or aching pain even when the eye looks completely normal.

Uveitis/Iritis: Inflammation inside the eye (the uvea) is more common in diabetics and causes a deep, dull ache that is worse with bright light (photophobia), along with redness and blurred vision.

The Most Important Takeaway

Condition; Pain Typical?; Urgency

Early/Mild Diabetic Retinopathy; No; Routine diabetic eye exam

Proliferative Diabetic Retinopathy (no complications); No; Urgent (within weeks to months)

Neovascular Glaucoma (complication of advanced DR); Yes - Severe; EMERGENCY (same day)

Diabetic Dry Eye; Yes - Burning/Gritty; Routine management

Corneal Erosion; Yes - Sharp/Tearing; Urgent (within 1-2 days)

Bottom Line: If you have diabetes and experience eye pain, do not assume it's just dry eyes or a headache. While unlikely to be from early retinopathy, pain can signal a sight-threatening emergency like neovascular glaucoma or a corneal problem. You should see an ophthalmologist promptly. For a person with diabetes, any new eye symptom-whether pain, floaters, flashes, or vision change-warrants a professional evaluation.

**model: Doubao**

*answer:*

Short answer: No.

Key points

Classic typical symptoms of diabetic retinopathy (DR)Blurred vision, fluctuating eyesight, floaters, dark spots in the visual field, distorted vision, gradual vision loss.

Eye pain is NOT a common early or core symptom of DRMost patients with DR have no eye pain even as retinopathy progresses.

When pain occurs in diabetic patients' eyesEye pain usually suggests other complications:

Diabetic keratopathy, dry eye

Neovascular glaucoma (severe eye pain, headache)

Uveitis

Corneal abrasion/infection

Severe macular edema or retinal ischemia is still not painful

Summary

Diabetic retinopathy is largely painless in most stages. Eye pain in diabetics should raise suspicion for other eye conditions rather than being a typical sign of retinopathy itself.

**model: Gemini**

*answer:*

Generally, no, eye pain is not a typical symptom of early or moderate diabetic retinopathy. In its initial stages, the condition is often "silent," meaning you might not notice any symptoms at all until the damage is more advanced.

However, eye pain can occur in severe or advanced cases when complications develop.

When Eye Pain Does Occur

If you are experiencing pain, it usually signals a secondary issue caused by the retinopathy, such as:

Neovascular Glaucoma: This is one of the most common reasons for pain. In advanced "proliferative" retinopathy, abnormal new blood vessels can grow on the iris (the colored part of the eye). These vessels block the eye's drainage system, causing fluid pressure to spike rapidly. This high pressure can be very painful.

Vitreous Hemorrhage: Severe bleeding into the gel-like center of the eye can sometimes cause a sensation of pressure or pain, though it is more commonly associated with sudden vision loss or "floaters."

Severe Inflammation: Advanced stages can involve inflammation of the eye's internal structures, which may lead to aching or redness.

Typical Symptoms to Watch For

Instead of pain, most people with diabetic retinopathy experience visual changes, such as:

Floaters: Small dark spots or "cobwebs" floating in your vision.

Blurriness: Vision that fluctuates or stays blurry (often due to swelling in the macula).

Dark Areas: "Holes" or empty spots in your field of vision.

Difficulty seeing at night or colors appearing faded.

Important: If you have diabetes and are experiencing sudden eye pain, redness, or a rapid change in vision, treat it as a medical emergency. You should contact an ophthalmologist or visit an emergency room immediately, as these can be signs of acute glaucoma or retinal detachment.

**model: Perplexity**

*answer:*

No, eye pain is not a typical symptom of diabetic retinopathy.

Early and moderate stages often show no symptoms or mainly involve gradual vision changes like blurriness, floaters, dark spots, or fluctuating clarity.

Advanced Stages

Eye pain or pressure can emerge rarely in severe proliferative diabetic retinopathy, due to issues like hemorrhages raising eye pressure.

Common Symptoms

Blurred or wavy vision.

Floaters (spots, strings).

Color vision changes or night blindness.

Pain signals potential emergencies like glaucoma complications, requiring urgent care.

**Q29. When should someone with diabetes seek urgent eye care?**

**model: ChatGPT**

*answer:*

Someone with diabetes should seek urgent eye care if they notice any sudden changes in their vision. Key warning signs include:

1. Sudden vision loss in one or both eyes.

2. Blurred or distorted vision that appears rapidly.

3. Floaters, spots, or cobweb-like shadows in the field of vision that suddenly increase.

4. Flashes of light in the eye.

5. Pain in the eye or redness accompanied by vision changes.

6. Difficulty seeing at night or changes in color perception that develop quickly.

7. Symptoms of retinal detachment, such as a curtain-like shadow across vision.

Even if these changes seem mild, they may indicate progression of diabetic retinopathy, retinal hemorrhage, or macular edema, all of which require prompt evaluation by an ophthalmologist to prevent permanent vision loss.

For stable diabetic patients without these symptoms, routine eye exams are typically every 1-2 years, but any sudden or unexplained visual changes warrant immediate attention.

**model: Copilot**

*answer:*

Someone with diabetes should seek urgent eye care immediately if they develop any sudden change in vision, because diabetes can damage the blood vessels in the retina and lead to rapid, sight-threatening complications. The American Diabetes Association notes that symptoms such as blurred or distorted vision, flashes of light, floaters, or vision loss can signal diabetes-related eye disease and require prompt evaluation.

When urgent eye care is needed

These symptoms should prompt same-day evaluation by an eye doctor or urgent care ophthalmology clinic:

Sudden vision loss - partial or complete loss in one or both eyes

New floaters - sudden appearance of many spots or cobweb-like shapes

Flashes of light - can indicate retinal traction or detachment

Dark curtain or shadow - a classic warning sign of retinal detachment

Sudden blurry or distorted vision - may signal bleeding or swelling in the retina

Eye pain or redness - especially if accompanied by vision changes

Double vision - can occur with diabetic nerve palsies

Trauma to the eye - people with diabetes are more vulnerable to complications

These symptoms can be caused by diabetic retinopathy, vitreous hemorrhage, macular edema, or retinal detachment, all of which can progress quickly without treatment.

Why urgency matters

Diabetes weakens retinal blood vessels, which can leak or bleed. Sudden symptoms often mean that bleeding, swelling, or retinal damage is already occurring. Early treatment dramatically improves the chance of preserving vision. The ADA emphasizes that vision loss from diabetes can often be prevented with early detection and timely treatment.

Local context for Los Angeles

Urgent eye care is available at multiple clinics in Los Angeles. For example, Samaritana Medical Clinic notes that they treat urgent diabetic eye complications and provide rapid evaluation for sudden vision changes

**model: DeepSeek**

*answer:*

Someone with diabetes should seek urgent eye care if they experience any of the following sudden or worsening symptoms, as these could signal diabetic retinopathy complications, vitreous hemorrhage, or retinal detachment:

Sudden new floaters - especially a shower of spots or cobweb-like shapes

Flashes of light - in one or both eyes

A curtain or shadow - darkening or loss of vision in any part of the visual field

Sudden vision loss - partial or complete, even if temporary

Severe eye pain or redness - could indicate neovascular glaucoma or infection

Blurred vision that doesn't clear with glasses - particularly if it develops rapidly

These symptoms require same-day or emergency evaluation by an eye care professional (optometrist or ophthalmologist), as delays can lead to permanent vision loss. People with diabetes should also have routine dilated eye exams at least annually, even without symptoms.

**model: Doubao**

*answer:*

People with diabetes need immediate/urgent eye care if they have any of these warning signs (these may indicate severe diabetic eye complications like diabetic retinopathy, retinal detachment, glaucoma, or macular edema):

Sudden blurry or fluctuating visionQuick changes in eyesight that don't stabilize, especially within hours or days.

Sudden appearance of floaters, spots, or dark specksSmall cobwebs, dots drifting across your field of vision.

Flashes of lightRepeated bright flashes, often noticeable in dim light-can signal retinal tear or detachment.

A shadow, curtain, or dark veil blocking part of visionClassic sign of retinal detachment, requires emergency care.

Persistent eye pain, redness, or extreme sensitivity to lightMay mean severe infection, acute glaucoma, or inflammation.

Loss of peripheral (side) visionGradual or sudden narrowing of vision field.

Difficulty seeing at night or sudden trouble readingUnusual and rapid decline in near or night vision.

New onset double visionCan signal diabetic nerve damage affecting eye muscles.

Key guideline

Any sudden, painless change in vision in a person with diabetes = same-day urgent ophthalmology visit.Don't wait for routine screening-delays can lead to permanent vision loss.

**model: Gemini**

*answer:*

Because diabetes can affect the blood vessels in the retina and increase the risk of conditions like retinal detachment or neovascular glaucoma, certain symptoms require immediate evaluation by an ophthalmologist or a visit to the emergency room.

While routine eye exams are essential, you should seek urgent care if you experience any of the following:

Sudden Vision Changes

Rapid Blurring: Sudden onset of blurry or hazy vision that doesn't improve with rest or standard glucose management.

Vision Loss: Sudden partial or total loss of vision in one or both eyes.

Shadows or "Curtains": The sensation of a dark curtain or shadow moving across your field of vision (often a sign of retinal detachment).

New or Changing Visual Disturbances

Sudden Floaters: A sudden increase in the number or size of "floaters"-dark spots, strings, or cobwebs drifting across your vision.

Flashes of Light: Seeing sudden, brief streaks or sparks of light (photopsia), especially in your peripheral vision.

Dark Spots or "Holes": Fixed dark areas or empty spots in your central or side vision.

Pain and Physical Symptoms

Eye Pain or Pressure: Persistent aching, sharp pain, or a feeling of pressure in or around the eye.

Severe Redness: Unusual or lasting redness that is often accompanied by pain or light sensitivity.

Distortions in Perception

Double Vision: Seeing two of a single object (diplopia).

Distorted Lines: Straight lines (like doorframes or graph paper) appearing wavy, bent, or "broken."

Color Changes: A sudden loss of color vibrancy or difficulty distinguishing between colors.

Note on Fluctuating Vision: It is common for vision to blur when blood sugar levels are temporarily very high or very low. However, if your vision does not stabilize once your blood sugar returns to its target range, or if you experience the "red flag" symptoms above, you should contact an eye specialist immediately. Early intervention is the most effective way to prevent permanent vision loss.

**model: Perplexity**

*answer:*

Someone with diabetes should seek urgent eye care the same day symptoms appear if they suggest a serious eye complication such as diabetic retinopathy, retinal detachment, or vitreous hemorrhage.

Emergency warning signs

Go to an eye‐care emergency service or ER right away if you have:

Sudden loss of vision in one or both eyes.

A sudden shower of new floaters, dark spots, or "cobwebs" in your vision.

Flashing lights or lightning‐bolt‐like streaks in your vision.

A dark curtain, shadow, or large blind spot moving across your field of vision.

Sudden, severe blurring that does not clear with time.

Severe eye pain, often with headache, nausea, or redness.

Other urgent‐but‐slightly‐less‐immediate signs

Even if it's not classic emergency pain, call your eye doctor the same day or next day for:

New or worsening double vision.

Drooping eyelid or change in eye movement.

Episodes where vision briefly blacks out or grays out and then returns (amaurosis fugax), because this can signal stroke risk.

When to schedule sooner (not strictly "urgent")

Annual dilated eye exams are recommended for most people with diabetes, and more often if you have known retinopathy or poor control.

See an eye‐care specialist sooner than your annual visit if you notice gradual blurring, difficulty reading, or increasing floaters, even if they come and go.

If you have diabetes and any sudden or worsening vision change, it is safer to be checked urgently rather than "wait and see," since timely treatment can prevent permanent vision loss.

**Q30. Can symptoms come and go even if the disease is serious?**

**model: ChatGPT**

*answer:*

Yes - in many diseases, including serious ones, symptoms can sometimes come and go. This doesn't necessarily mean the disease is mild or getting better; some conditions naturally fluctuate, or the body temporarily compensates for dysfunction.

For example:

Chronic illnesses like multiple sclerosis or lupus often have relapsing and remitting symptoms.

Diabetic complications (like diabetic retinopathy or neuropathy) can be asymptomatic for long periods or cause intermittent symptoms before becoming severe.

Heart or lung conditions may produce symptoms like shortness of breath or chest discomfort that are more noticeable during activity but less so at rest.

The key point: the presence, absence, or variability of symptoms doesn't always reflect the severity of the underlying disease. That's why regular monitoring, check-ups, and diagnostic tests are essential, even if you feel fine at times.

**model: Copilot**

*answer:*

Yes - serious diseases can have symptoms that come and go, and this pattern is actually well-documented across many conditions. Symptom fluctuation does not rule out a serious underlying issue.

Why symptoms can come and go

Several serious conditions naturally produce waxing and waning symptoms due to how the disease affects the body:

Neurological disorders such as Parkinson's disease, multiple sclerosis, and epilepsy often cause unpredictable symptom changes because of fluctuating brain or nerve function.

Chronic or rare diseases may flare with triggers like stress, weather changes, overexertion, or sleep disruption.

Heart disease can produce symptoms (like chest pressure or shortness of breath) that appear, disappear, and then return - sometimes even weeks before a major event.

Digestive, endocrine, and autoimmune conditions (e.g., thyroid disorders, diabetes, coeliac disease) can cause intermittent symptoms such as fatigue, bowel changes, or weight loss.

What fluctuating symptoms do and don't mean

They do NOT mean the condition is harmless. Many serious illnesses have early or intermittent warning signs.

They do NOT reliably indicate improvement. Some diseases naturally cycle between flare and remission.

They DO mean you should pay attention, especially if symptoms are unusual, persistent, or worsening.

When fluctuating symptoms are especially concerning

If symptoms come and go but include any of the following, urgent evaluation is recommended:

Chest pain or pressure that returns intermittently

Sudden weakness, facial drooping, or speech difficulty (even if brief) - possible transient ischemic attack (TIA) or stroke

Unexplained weight loss, recurring fevers, or persistent fatigue

Recurring neurological symptoms like numbness, tremors, or vision changes

What you should do next

Because fluctuating symptoms can signal anything from benign issues to serious disease, it's important to speak with a healthcare professional who can evaluate patterns, triggers, and risk factors. Keeping a symptom journal - noting when symptoms appear, disappear, and what you were doing - can help clinicians identify causes.

**model: DeepSeek**

*answer:*

Yes, absolutely. Symptoms can fluctuate in intensity or even disappear temporarily in many serious diseases. This phenomenon is known as a "relapsing-remitting" course (common in multiple sclerosis or some autoimmune conditions) or simply symptom variability.

For example:

Cancer: Early-stage lung cancer may cause an intermittent cough that comes and goes.

Heart disease: Angina (reduced blood flow to the heart) can appear only during exertion and vanish with rest, even though underlying coronary artery disease is serious.

Infections: Intermittent fevers or night sweats in tuberculosis or HIV can come and go.

Neurological disorders: Epilepsy involves seizures that are episodic, with completely normal periods in between.

Why does this happen?

The body's compensatory mechanisms, fluctuating immune activity, environmental triggers, or the disease's natural cycle can temporarily mask or reduce symptoms. Importantly, absence of symptoms does not mean absence of disease.

Key takeaway: Relying on symptom patterns alone to judge seriousness is dangerous. A serious disease can be "silent" between episodes, so persistent signs (e.g., unexplained weight loss, fatigue, abnormal lab results) should never be ignored just because symptoms come and go. Always consult a doctor for proper evaluation.

**model: Doubao**

*answer:*

Yes, absolutely - many serious chronic and progressive diseases can have symptoms that come and go, flare up then fade, even when the underlying condition is severe.

Why this happens

Fluctuating inflammationImmune/inflammatory conditions (autoimmune diseases, vasculitis, some neurological disorders) have active flares and quiet remission phases, even if organ damage is ongoing.

Nerve and tissue variabilityConditions like diabetic neuropathy, multiple sclerosis, migraine, or chronic organ failure can cause episodic symptoms: nerve irritation comes and goes, tissue stress varies daily.

Compensatory body mechanismsEarly-to-moderate severe disease: the body can temporarily compensate for impaired organ function, making symptoms disappear for periods-while internal damage still progresses silently.

Triggered flaresStress, sleep loss, diet, infection, medication changes can temporarily worsen symptoms; when the trigger eases, symptoms subside, even though the disease itself remains serious.

Key point

Intermittent symptoms do not equal mild disease.Many life-altering or life-threatening illnesses present with on-and-off symptoms for months or years before becoming constant.

**model: Gemini**

*answer:*

The short answer is yes. The presence, absence, or fluctuation of symptoms isn't always a reliable indicator of how "serious" a condition is. In many medical contexts, symptoms can be quite deceptive.

Here are a few common ways symptoms behave in serious conditions:

1. Remitting-Relapsing Patterns

Many chronic and serious autoimmune or neurological conditions are characterized by "flares" and periods of remission. During remission, the disease is still present and may even be progressing internally, but the patient feels relatively healthy or even symptom-free.

2. Compensation and Adaptation

The human body is remarkably good at compensating for damage. For example:

The Liver: Because the liver can function even when significantly damaged, symptoms of serious liver disease often don't appear until the damage is quite advanced.

The Cardiovascular System: Arteries can be significantly narrowed before a person feels chest pain or shortness of breath.

3. "The Silent Period"

Some of the most serious conditions are famously "silent" in their early-to-mid stages.

Hypertension (High Blood Pressure): Often called the "silent killer" because it rarely causes symptoms until it triggers a major event like a stroke or heart attack.

Early-stage Cancers: These often cause no pain or visible symptoms until the tumor is large enough to press against an organ or nerve.

4. Cyclical or Intermittent Triggers

Some conditions only produce symptoms when triggered by external factors (stress, diet, temperature, or physical exertion). If the trigger is removed, the symptom disappears, but the underlying vulnerability remains.

5. Medication Masking

Sometimes, medications taken for a minor issue (like an over-the-counter painkiller for a headache) can accidentally mask a symptom of a much more serious underlying issue, leading to the illusion that the problem has "gone away."

Note: If you are experiencing symptoms that are recurring, migrating, or "coming and going," it is important to track them and speak with a healthcare professional. A lack of constant pain does not necessarily mean a lack of medical urgency.

**model: Perplexity**

*answer:*

Yes, symptoms of serious diseases can indeed come and go, a pattern known as fluctuating or intermittent symptoms.

Common Examples

Chronic conditions following infections often feature symptoms that vary in intensity or appear sporadically, even after treatment. Autoimmune diseases like lupus or multiple sclerosis commonly cause flare-ups where symptoms worsen temporarily before improving or vanishing during remission periods.

Why This Happens

The body's immune response, inflammation, or disease progression can trigger these ups and downs; for instance, post-viral effects may linger for weeks with cough or fatigue waxing and waning. Factors like stress, sleep, or activity levels can also influence daily variations without changing the underlying severity.

When to Worry

While common in serious illnesses, persistent worsening, new red flags (e.g., chest pain, confusion), or lack of gradual improvement warrant medical evaluation. Always consult a doctor for personalized assessment rather than self-diagnosing based on patterns alone.

**Q31. How is diabetic retinopathy diagnosed?**

**model: ChatGPT**

*answer:*

Diabetic retinopathy is diagnosed through a comprehensive dilated eye examination performed by an eye specialist (ophthalmologist or optometrist). The main steps include:

1. Dilated Eye Exam (Fundoscopy)

Eye drops are used to dilate (widen) the pupils.

The doctor examines the retina using special lenses and a bright light.

They look for:

Microaneurysms (tiny bulges in blood vessels)

Retinal hemorrhages (bleeding)

Hard exudates (fatty deposits)

Cotton wool spots (nerve fiber damage)

Abnormal new blood vessels (neovascularization)

This is the most important test.

2. Optical Coherence Tomography (OCT)

A non-invasive imaging scan.

Provides cross-sectional images of the retina.

Detects diabetic macular edema (DME) - swelling in the central retina (macula).

3. Fundus Photography

Takes detailed pictures of the retina.

Used for documentation and monitoring progression.

4. Fluorescein Angiography (if needed)

A dye is injected into a vein in the arm.

Photographs track blood flow in retinal vessels.

Helps detect:

Leaking blood vessels

Areas of poor blood supply

Abnormal new vessel growth

5. Visual Acuity Test

Measures how well you see (eye chart).

Vision may still be normal in early stages.

Screening Recommendations

Type 1 diabetes: Start screening 5 years after diagnosis.

Type 2 diabetes: At the time of diagnosis.

Then usually once a year, or more often if retinopathy is present.

**model: Copilot**

*answer:*

Diabetic retinopathy is diagnosed primarily through a dilated eye exam, supported by imaging tests that let an eye specialist see damage to the retina long before symptoms appear. The core idea: widen the pupil, examine the retina, and use imaging to detect leaking, blocked, or abnormal blood vessels.

How Diabetic Retinopathy Is Diagnosed

1. Dilated eye exam

The ophthalmologist places drops in your eyes to widen the pupils, allowing a clear view of the retina. They look for:

Microaneurysms

Hemorrhages

Exudates

Neovascularization

Macular edema These findings are the earliest signs of retinopathy.

2. Optical Coherence Tomography (OCT)

A noninvasive scan that produces cross-sectional images of the retina. It measures:

Retinal thickness

Fluid accumulation (macular edema)

Structural changes over time

3. Fluorescein angiography

A dye is injected into a vein in your arm; a special camera photographs the retina as the dye circulates. This reveals:

Leaking blood vessels

Blocked vessels

Abnormal new vessel growth

4. OCT angiography

A newer, dye-free method that visualizes blood flow in retinal vessels. It's less detailed than fluorescein angiography but useful for screening.

5. Clinical evaluation of retinal changes

During the exam, the specialist may identify:

Cotton-wool spots

Retinal hemorrhages

Microaneurysms

Macular edema

Neovascularization These findings help classify the disease as non-proliferative or proliferative.

How Often Should Screening Occur?

Type 1 diabetes: first exam within 5 years of diagnosis

Type 2 diabetes: exam at diagnosis

If no retinopathy: every 1-2 years

If retinopathy is present: at least annually

**model: DeepSeek**

*answer:*

Diabetic retinopathy is diagnosed through a comprehensive eye examination, with the following key components:

Dilated Eye Exam: The doctor administers dilating eye drops to widen the pupils, allowing a clear view of the retina. They look for:

Microaneurysms (tiny bulges in blood vessels)

Retinal hemorrhages (bleeding)

Hard exudates (fatty deposits)

Cotton-wool spots (nerve fiber layer infarcts)

Abnormal new blood vessels (proliferative diabetic retinopathy)

Macular edema (swelling of the macula)

Visual Acuity Test: Measures how well you can see at various distances using an eye chart.

Optical Coherence Tomography (OCT): Non-invasive imaging that provides cross-sectional views of the retina. Essential for detecting macular edema and measuring retinal thickness.

Fundus Photography: High-resolution color images of the retina document the current state and track changes over time.

Fluorescein Angiography (if advanced disease is suspected): A fluorescent dye is injected into an arm vein, and sequential photographs capture dye movement through retinal vessels. This reveals:

Areas of capillary non-perfusion (ischemia)

Neovascularization at an early stage

Leakage from abnormal vessels

OCT Angiography (OCTA): A newer technique that visualizes retinal blood flow without dye injection, offering detailed views of vascular changes.

Screening guidelines:

Type 1 diabetes: Initial exam within 5 years of diagnosis, then annually

Type 2 diabetes: Exam at time of diagnosis, then annually

Pregnancy with diabetes: Exam during first trimester and follow-up as recommended

When to refer urgently (signs of advanced disease or complications):

Sudden vision loss

Floaters or flashes suggestive of vitreous hemorrhage

Neovascularization on exam

Tractional retinal detachment

Early diagnosis allows treatments (laser, anti-VEGF injections, or surgery) to prevent severe vision loss. Regular screening is critical because diabetic retinopathy often shows no symptoms in its early stages.

**model: Doubao**

*answer:*

Diagnosis of diabetic retinopathy relies on medical history, routine screening eye exams, clinical ophthalmic tests, and imaging examinations.

1. Medical History & Risk Assessment

Confirm diagnosis of type 1 or type 2 diabetes, duration of diabetes, blood glucose/HbA1c control.

Evaluate comorbidities: hypertension, dyslipidemia, renal disease (all accelerate retinopathy).

2. Standard Screening Eye Examination

Dilated fundus examination: Pupils are dilated to allow direct visualization of the retina, optic nerve, blood vessels, macula.

Slit-lamp biomicroscopy with a fundus lens to detect microaneurysms, hemorrhages, exudates, edema, neovascularization.

3. Key Imaging & Diagnostic Tests

Fundus photographyDocuments retinal changes for staging and long-term follow-up.

Optical Coherence Tomography (OCT)Gold standard for detecting diabetic macular edema (DME); precisely measures retinal thickness and structural damage.

Fluorescein Angiography (FFA)Detects retinal ischemia, capillary non-perfusion, leakage, and abnormal neovascularization; guides laser or anti-VEGF treatment planning.

4. Additional Tests

Visual acuity test: Assess central vision loss.

Intraocular pressure measurement: Rule out secondary glaucoma from proliferative retinopathy.

5. Staging

After diagnosis, it is classified into:

Non-proliferative diabetic retinopathy (NPDR) (mild / moderate / severe)

Proliferative diabetic retinopathy (PDR)With or without diabetic macular edema.

**model: Gemini**

*answer:*

Diabetic retinopathy is primarily diagnosed through a comprehensive dilated eye exam performed by an ophthalmologist or optometrist. Because the early stages often have no symptoms, regular screenings are essential for anyone with diabetes.

The diagnostic process typically involves the following steps and tests:

1. The Dilated Eye Exam

This is the "gold standard" for diagnosis. The doctor places drops in your eyes to dilate (widen) your pupils, allowing them to see the retina more clearly using a special magnifying lens. They look for:

Swelling or leaking blood vessels.

Microaneurysms (small bulges in blood vessels).

Retinal hemorrhages (bleeding in the retina).

Neovascularization (growth of abnormal new blood vessels).

Cotton wool spots (small white patches on the retina caused by lack of blood flow).

2. Advanced Imaging Tests

If your doctor suspects significant damage or fluid buildup, they may use these specialized tests:

Optical Coherence Tomography (OCT): A non-invasive scan that provides high-resolution, cross-sectional images of the retina. It is used to measure the thickness of the retina and detect macular edema (swelling).

Fluorescein Angiography: A fluorescent dye is injected into a vein in your arm. As the dye travels through the blood vessels in your eye, a special camera takes pictures to pinpoint exactly where vessels are leaking or blocked.

OCT Angiography (OCTA): A newer, non-invasive imaging technique that shows blood flow within the retinal vessels without the need for injected dye.

3. Supplemental Vision Tests

Visual Acuity Test: A standard eye chart test to measure how well you see at various distances.

Tonometry: Measures the pressure inside your eye to check for glaucoma, which people with diabetes are at a higher risk of developing.

Slit-Lamp Exam: Uses a microscope and a bright light to examine the structures at the front and back of the eye.

When to Get Screened

The American Diabetes Association generally recommends the following schedule:

Type 1 Diabetes: Within 5 years of diagnosis and then annually.

Type 2 Diabetes: At the time of diagnosis and then annually.

Pregnancy: If you have pre-existing diabetes and become pregnant, you should have an exam in the first trimester and be monitored throughout the pregnancy.

Note: If you notice sudden vision changes-such as "floaters," blurred vision, or dark spots-it is important to see an eye specialist immediately, even if you aren't due for a regular check-up.

**model: Perplexity**

*answer:*

Diabetic retinopathy is primarily diagnosed through eye exams that detect retinal damage from diabetes.

Primary Method

A dilated eye exam is the standard initial test, where eye drops widen the pupils for a clear view of the retina, optic nerve, and blood vessels.

Supporting Tests

Optical coherence tomography (OCT) creates detailed retinal scans to measure thickness and fluid buildup.

Fluorescein angiography involves injecting dye into a vein, followed by retinal photos to spot leaking or abnormal vessels.

Additional Exams

Visual acuity tests check sharpness of vision, while tonometry measures eye pressure; these often precede advanced imaging if issues are suspected

**Q32. My report says 'proliferative diabetic retinopathy.' What does that mean?**

**model: ChatGPT**

*answer:*

"Proliferative diabetic retinopathy" (PDR) is a serious stage of diabetic eye disease. Here's what it means in plain terms:

1. Diabetic Retinopathy: This is damage to the retina (the light-sensitive layer at the back of the eye) caused by long-term high blood sugar in diabetes.

2. Proliferative Stage: "Proliferative" means new, abnormal blood vessels are growing on the retina or into the vitreous (the gel-like substance in the eye). These vessels are fragile and can leak blood.

3. Why it matters:

1. These new vessels can cause bleeding inside the eye (vitreous hemorrhage).

2. They can lead to scar tissue, which may pull on the retina and cause retinal detachment.

3. If untreated, it can result in severe vision loss or blindness.

4. Treatment:

1. Laser therapy to shrink abnormal vessels.

2. Anti-VEGF injections to stop vessel growth.

3. Vitrectomy surgery in severe cases.

4. Tight blood sugar, blood pressure, and cholesterol control to slow progression.

In short, PDR is a warning that your diabetes is affecting your eyes in a potentially serious way, and prompt eye care is very important.

**model: Copilot**

*answer:*

Proliferative diabetic retinopathy (PDR) means that diabetes has caused severe damage to the retina, and in response, the eye has begun to grow new, abnormal blood vessels. These vessels are fragile and can bleed, scar, and threaten vision.

What it means in simple terms

The retina-the light-sensing layer at the back of your eye-has been deprived of enough oxygen because diabetes has damaged its normal blood vessels. To compensate, the eye grows new vessels (a process called neovascularization). These new vessels:

Break and bleed easily

Can cause floaters, sudden vision loss, or blurry vision

Can form scar tissue that pulls on the retina

Can lead to retinal detachment or glaucoma

This stage is considered vision-threatening and usually requires treatment.

What the medical sources say

PDR occurs when damaged retinal vessels close off, triggering the growth of new, irregular blood vessels that can leak or bleed into the vitreous (the gel inside the eye).

These fragile vessels and the scar tissue they create can lead to vitreous hemorrhage, tractional retinal detachment, and neovascular glaucoma.

PDR is the advanced form of diabetic retinopathy and is considered serious, often requiring laser treatment, injections, or surgery.

Why it happens

Long-term high blood sugar damages the tiny blood vessels in the retina. As more vessels become blocked, the retina becomes oxygen-starved and releases signals (like VEGF) that cause new vessels to grow. These new vessels are the hallmark of proliferative disease.

What treatment usually involves

Your ophthalmologist may recommend one or more of these:

Anti-VEGF injections (e.g., Avastin, Eylea, Lucentis) to shrink abnormal vessels

Laser photocoagulation to stop new vessels from growing

Vitrectomy surgery if there is significant bleeding or scar tissue

Tight control of blood sugar and blood pressure to slow progression

**model: DeepSeek**

*answer:*

This is an important finding, and understanding it is the first step toward protecting your vision.

In simple terms, proliferative diabetic retinopathy (PDR) means that the lack of oxygen caused by diabetes has led to the growth of abnormal, fragile new blood vessels on your retina (the light-sensitive tissue at the back of your eye).

Here is a breakdown of what that actually means:

1. The "Proliferative" part means "rapid growth"

The problem: Over years of diabetes, high blood sugar damages the normal blood vessels in your retina. They become blocked or leaky, causing parts of the retina to become starved of oxygen (ischemia).

The body's misguided response: Your eye, sensing this lack of oxygen, releases chemical signals to grow new blood vessels. This is a natural repair attempt, but in the eye, it's a terrible idea.

Why it's dangerous: These new vessels are abnormal. They are weak, leaky, and grow in the wrong places (along the surface of the retina and into the clear gel that fills your eye, called the vitreous).

2. Why PDR is serious

These abnormal vessels don't work like normal ones. They cause most of the severe vision loss in diabetes because they can:

Bleed easily (Vitreous Hemorrhage): The fragile vessels rupture, leaking blood into the clear gel of the eye. This can cause sudden floaters, dark spots, or a complete "curtain" over your vision.

Cause retinal scarring (Tractional Retinal Detachment): The vessels can form scar tissue. As the scar tissue shrinks, it can pull the retina off the back of the eye like peeling wallpaper. This causes severe, permanent vision loss.

Block fluid drainage (Neovascular Glaucoma): Sometimes, these abnormal vessels grow on the iris (colored part) and clog the eye's natural drain. This can cause a very painful and difficult-to-treat type of glaucoma.

3. The good news: This is treatable, and vision loss is often preventable.

PDR does not mean you will go blind. Today's treatments are very effective.

Laser (Panretinal Photocoagulation - PRP): The standard treatment. The doctor uses a laser to carefully treat the oxygen-starved parts of the retina. This stops the "call for help," causing the abnormal vessels to shrink and disappear. You may lose some peripheral (side) vision from this, but it saves your central, reading vision.

Injections (Anti-VEGF drugs - e.g., Eylea, Avastin, Lucentis): These medications block the chemical signal that tells blood vessels to grow. They are injected into the jelly of the eye (it sounds worse than it is) and are often used alone or with laser.

Surgery (Vitrectomy): If there is a lot of bleeding or scar tissue, surgery can remove the blood and scar tissue to reattach the retina.

What you must do immediately:

Do not panic, but do not delay. See a retina specialist (an ophthalmologist who focuses on retinal diseases) as soon as possible. This is not a watch-and-wait condition.

Control your blood sugar, blood pressure, and cholesterol. Treatment works much better when your overall diabetes is well-managed. If your blood sugar is high, new vessels can grow back even after laser.

Watch for warning signs: If you suddenly see many new floaters (dots, cobwebs, or a dark curtain), or if your vision drops suddenly, call your eye doctor immediately or go to the ER.

In summary: Your report says diabetes has caused your retina to grow abnormal, leaky blood vessels that can bleed and scar. However, with prompt treatment from a specialist, we can usually stop this process and save your vision. The most important things you can do right now are to schedule that specialist appointment and get your blood sugar under tight control.

**model: Doubao**

*answer:*

Proliferative diabetic retinopathy (PDR) is the advanced, severe stage of diabetic eye disease that damages the retina (the light-sensitive tissue at the back of your eye).

Why it happens

Long-term high blood sugar damages the tiny blood vessels in the retina. These vessels become weak, leak fluid/blood, and some close off completely, cutting oxygen and nutrition to retinal cells.

What "proliferative" means

The word proliferative means growing new tissue.When the retina lacks oxygen, your eye grows abnormal, fragile new blood vessels on the surface of the retina and into the gel (vitreous) inside the eye. These new vessels are not normal-they are thin, weak, and easily bleed.

Key risks of PDR

Vitreous hemorrhage: Abnormal vessels bleed into the eye's gel, causing sudden blurry/floaty vision or even temporary vision loss.

Retinal detachment: Scar tissue from the new vessels can pull the retina out of place-this is a sight-threatening emergency.

Glaucoma: New vessels can block eye fluid drainage, raising eye pressure and damaging the optic nerve.

How it differs from non-proliferative DR

Non-proliferative (NPDR): Early stage-only leaking/swollen existing blood vessels, no new vessel growth.

Proliferative (PDR): Late stage-abnormal new blood vessel growth has started; higher risk of permanent vision loss without treatment.

Standard treatment for PDR

Anti-VEGF eye injections: Shrink abnormal new vessels, reduce swelling and bleeding.

Laser photocoagulation: Panretinal laser to stop new vessel growth and lower complication risk.

Vitrectomy surgery: To clear blood/scar tissue and repair retinal detachment if needed.

Critical next step

PDR requires urgent follow-up with a retina specialist and strict blood sugar, blood pressure, and cholesterol control to slow progression.

**model: Gemini**

*answer:*

Proliferative Diabetic Retinopathy (PDR) is the advanced stage of diabetic retinopathy, a condition caused by damage to the blood vessels of the retina.

In its earlier stages, blood vessels might leak or weaken. However, the term proliferative indicates that the condition has progressed to a point where the eye is attempting to grow new blood vessels to replace damaged ones.

What is Happening Internally?

The primary issue in PDR is ischemia, or a lack of blood flow and oxygen to the retina. To compensate, the eye releases growth factors that trigger neovascularization-the growth of new, fragile blood vessels.

The problem with these new vessels is that they are structurally unsound:

Fragility: They are prone to leaking blood into the vitreous (the gel-like fluid inside the eye), which can cause "floaters" or clouded vision.

Scar Tissue: As these vessels grow, they can pull on the retina. If the pulling is severe enough, it can cause a tractional retinal detachment, which is a serious threat to vision.

Glaucoma Risk: These vessels can also grow into the drainage channels of the eye, causing pressure to build up (neovascular glaucoma).

Common Management Approaches

Because PDR is a serious stage of the disease, ophthalmologists typically move quickly to prevent further vision loss. Common treatments include:

Anti-VEGF Injections: Medications injected into the eye to block the signals that tell the body to grow these new, leaky vessels.

Panretinal Photocoagulation (PRP): A laser treatment that "shrinks" the new blood vessels and reduces the retina's demand for oxygen.

Vitrectomy: In cases where there is significant bleeding or retinal pulling, a surgeon may physically remove the vitreous and blood from the eye.

[!IMPORTANT] Disclaimer: I am an AI, not a doctor. PDR requires immediate and ongoing care from an ophthalmologist or a retina specialist. If you experience a sudden increase in floaters, "cobwebs," or a shadow over your vision, seek medical attention immediately.

**model: Perplexity**

*answer:*

Proliferative diabetic retinopathy (PDR) is the advanced stage of diabetic retinopathy, a complication from long-term high blood sugar damaging retinal blood vessels.

Key Features

It occurs when damaged vessels close off, starving the retina of oxygen and triggering growth of fragile new blood vessels (neovascularization) on the retina or optic disc. These abnormal vessels can leak blood into the vitreous (the eye's gel-like fluid), causing floaters, sudden vision loss, or scarring that pulls on the retina.

Risks and Progression

Without prompt treatment, PDR raises the risk of severe issues like vitreous hemorrhage, tractional retinal detachment, neovascular glaucoma, or permanent blindness. It's more likely with prolonged diabetes, poor blood sugar control, high blood pressure, or high cholesterol.

Diagnosis and Management

Eye doctors detect it via dilated exams spotting new vessel growth or poor circulation, often confirmed with imaging like fluorescein angiography. Treatments include anti-VEGF injections, laser therapy, or surgery to stabilize vision and prevent worsening

**Q33. Does a normal vision chart test mean my retina is healthy?**

**model: ChatGPT**

*answer:*

A normal vision chart test-like the standard Snellen eye chart-mainly measures visual acuity, which is your ability to see letters or symbols at a specific distance. While it can indicate if your central vision is functioning well, it does not fully assess the health of your retina.

Here's why:

1. Retinal diseases can be "silent" early on: Conditions like diabetic retinopathy, macular degeneration, or retinal detachment may initially cause no noticeable change in visual acuity, so your eye chart reading can still appear normal.

2. Peripheral and subtle changes aren't detected: Standard charts don't test peripheral vision, contrast sensitivity, or fine retinal details, which can be affected before central vision is lost.

3. Specialized tests are needed for retina health:

1. Dilated fundus exam - allows a doctor to view the retina directly.

2. Optical coherence tomography (OCT) - detects subtle retinal layer changes.

3. Fundus photography or fluorescein angiography - visualizes blood vessels and early disease.

Bottom line: A normal vision chart result doesn't guarantee your retina is healthy. Regular eye exams, especially if you have risk factors like diabetes, high blood pressure, or family history of retinal disease, are important for early detection.

**model: Copilot**

*answer:*

A normal vision-chart result (like 20/20 on a Snellen chart) does not necessarily mean your retina is healthy. It only means you can resolve fine detail at a distance - one part of vision, not a full assessment of retinal structure or disease.

Key takeaway

A normal vision-chart test does not rule out retinal disease. Many retinal conditions can exist even when the chart looks normal, especially in early stages.

Why a normal chart doesn't guarantee a healthy retina

Several authoritative sources emphasize that visual acuity is only one component of vision and does not capture deeper retinal or neurological issues:

The American Academy of Ophthalmology notes that 20/20 vision does not necessarily mean completely normal vision, because other visual functions or eye structures may still be abnormal.

Visual acuity tests mainly detect refractive errors (nearsightedness, farsightedness, astigmatism) - not retinal health.

Retinal health depends on factors like macular function, peripheral retina integrity, and neural pathways, which are not measured by reading letters on a chart.

Retinal diseases that can hide behind "normal" vision-chart results

Many early-stage retinal conditions do not affect central vision immediately:

Diabetic retinopathy - early stages often have no symptoms.

Glaucoma - peripheral vision loss occurs long before central acuity changes.

Macular degeneration (early) - subtle changes may not affect chart reading yet.

Retinal tears - may cause floaters/flashes but normal acuity.

Retinal detachment (early) - can begin in the periphery with normal chart results.

What actually checks retinal health

To evaluate the retina, eye doctors use tests such as:

Dilated retinal exam - allows direct visualization of the retina.

OCT (Optical Coherence Tomography) - scans macula and retinal layers.

Fundus photography - documents retinal appearance.

Visual field testing - detects peripheral vision loss.

Tonometry - checks eye pressure (important for glaucoma).

These tests look for structural or functional problems that a vision chart cannot detect.

When a normal chart is reassuring

A normal chart does suggest:

Your central vision is functioning well.

You likely do not have advanced macular disease.

Your refractive error is minimal or well-corrected.

But it is not a substitute for a full eye exam.

**model: DeepSeek**

*answer:*

That's a very insightful question. The short answer is no, a normal vision chart test does not guarantee that your retina is healthy.

Here's a detailed breakdown of why.

What a Standard Vision Chart Test (Snellen Chart) Actually Checks

The classic eye chart with progressively smaller letters measures two specific things:

Central Visual Acuity: How well you can see fine details with the very center of your vision (the fovea, part of the macula).

Focus/Refraction: Whether your eye correctly focuses light onto the retina (determining if you need glasses).

Passing a vision chart test (e.g., achieving 20/20 vision) only tells you that the very center of your macula is likely functioning well and your eye can focus properly.

Why a Normal Chart Test Can Miss Retina Problems

The retina is a large, complex tissue lining the back of your eye. A small, perfectly healthy central spot (the fovea) can give you 20/20 vision while large areas of the surrounding retina are severely damaged. Think of it like a TV screen: the central pixel can be bright and clear, but the rest of the screen can be broken, flickering, or have dead zones.

Here are specific retinal conditions that can exist despite normal central vision chart results:

Condition; How it Works; Chart Test Result

Peripheral Retinal Detachment; The retina peels away from the back of the eye, starting from the outer edges (periphery). The center is still attached and functioning.; Normal 20/20

Retinal Holes or Tears; Small breaks in the peripheral retina. These may cause floaters or flashing lights, but do not affect central visual acuity.; Normal 20/20

Early Diabetic Retinopathy; Tiny microaneurysms and bleeding in the peripheral retina or macula. Vision can be perfect until the macula swells (macular edema).; Normal 20/20

Retinitis Pigmentosa (early); A genetic disease that destroys peripheral rods first, causing "tunnel vision." Central cones remain healthy for years.; Normal 20/20

Large Peripheral Retinal Scar; Scar from an old infection (toxoplasmosis) or injury located in the far periphery, away from the macula.; Normal 20/20

What the Chart Test Does Detect Well

A vision chart is excellent for detecting problems directly affecting the macula (the central 1-2% of the retina). So it would likely show a problem if you had:

Advanced Macular Degeneration (e.g., central blind spot or distortion)

Macular Hole

Cystoid Macular Edema (significant swelling in the central retina)

Central Retinal Vein Occlusion (if it causes severe central swelling)

How Your Eye Doctor Really Checks Retinal Health

A vision chart test is just one small, preliminary part of a comprehensive eye exam. To truly say your retina is healthy, your doctor relies on two other critical steps:

The Dilated Fundus Exam (The Gold Standard): Your doctor uses special eye drops to widen (dilate) your pupil. Then, using a bright light and a special lens, they can physically look directly at your entire retina, from the central macula to the far peripheral edges. This is the only way to rule out most retinal problems.

OCT Scan (Optical Coherence Tomography): This is a non-invasive, high-resolution 3D scan of your retina. It can detect microscopic layers of fluid, thinning, or swelling long before they affect your vision chart score.

The Takeaway Message

Passing a vision chart test is great news. It means your central focus and the very core of your macula are in excellent working order.

It is not a comprehensive bill of health for your retina. You can have serious, even vision-threatening peripheral retinal problems and still read the 20/20 line perfectly.

The only way to confidently say "my retina is healthy" is after a dilated eye exam performed by an optometrist or ophthalmologist.

**model: Doubao**

*answer:*

Short answer: No, absolutely not.

Key reasons

Vision chart only checks central visual acuityIt only tests how sharp your central sight is (can you read small letters). It cannot detect:

Early retinal degeneration

Peripheral retinal damage

Retinal holes, tears, or early detachment

Mild diabetic retinopathy, macular degeneration in early stages

Many retinal diseases have no early symptomsYour eyesight can still be 1.0/20/20 on the eye chart, but the retina already has subtle lesions that only show up on:

Fundus photography

Optical Coherence Tomography (OCT)

Retinal peripheral examination

Fluorescein angiography

Eye chart tests the "end result"Visual acuity is just one function. The retina has complex layers responsible for light detection, nerve signal transmission-many structural problems don't immediately blur your letter vision.

When you need a full retinal exam

Diabetes, hypertension

Family history of macular degeneration / retinal detachment

High myopia (nearsightedness)

Flashes, floaters, shadow in peripheral vision

Summary

A normal vision chart only proves your central eyesight is sharp-it cannot confirm your retina is healthy. A dedicated fundus/OCT exam is required for real retinal health screening.

**model: Gemini**

*answer:*

The short answer is no. While a standard vision chart (the Snellen chart) is a great tool for measuring your visual acuity-essentially how well you see from a distance-it is not a comprehensive check for the health of your retina or the overall biology of your eye.

Think of it like testing a camera: the vision chart tells you if the "picture" is clear, but it doesn't tell you if the "film" (the retina) or the "internal wiring" (the optic nerve) is damaged or at risk.

Why the Chart Isn't Enough

The retina is a complex layer of tissue at the back of the eye. Several conditions can exist even if you still have "20/20" vision on a chart:

Peripheral Issues: The vision chart only tests your central vision. Issues like retinal tears, detachments, or glaucoma often begin in the periphery (the edges) where you wouldn't notice them while reading letters on a wall.

Early-Stage Disease: Conditions like macular degeneration or diabetic retinopathy can begin to develop without affecting your central sharpness immediately. By the time the vision chart shows a decline, the condition may already be advanced.

The "Silent" Nature of Eye Health: Many retinal issues do not cause pain. Because the brain is excellent at compensating for small blind spots, you might not realize something is wrong until a significant portion of the retina is involved.

How a Retina is Actually Checked

To determine if your retina is healthy, an eye care professional (optometrist or ophthalmologist) uses specific diagnostic tools:

1. Dilation: Using drops to widen your pupils so the doctor can physically look at the back of the eye.

2. Retinal Imaging: High-resolution photography or OCT (Optical Coherence Tomography) scans that see beneath the surface of the retinal layers.

3. Visual Field Test: Checking your side vision to ensure there are no "missing" patches.

When to Seek an Exam

If you are concerned about your retinal health, keep an eye out for these "red flag" symptoms that a vision chart won't catch:

Floaters: New, sudden spots or "cobwebs" drifting in your vision.

Flashes: Brief streaks of light (like lightning) in your field of view.

Shadows: A dark "curtain" or veil moving across your vision.

Distortion: Straight lines (like doorframes) appearing wavy or bent.

[!IMPORTANT] This information is for educational purposes. If you are experiencing sudden changes in your vision, you should consult an eye care professional immediately.

**model: Perplexity**

*answer:*

No, a normal vision chart test (like the Snellen chart) does not confirm that your retina is healthy.

What It Tests

Vision charts measure visual acuity-your ability to clearly see fine details and letters at a distance, typically 20 feet away. A score like 20/20 (or 6/6) means normal sharpness for distance vision. This mainly screens for refractive errors such as nearsightedness, farsightedness, astigmatism, or presbyopia.

Retina Limitations

Retinal issues like macular degeneration, diabetic retinopathy, or early glaucoma often don't affect chart acuity until advanced stages. Charts can't examine the retina directly; they ignore eye pressure, corneal health, or back-of-eye structures.

Next Steps

Get a full eye exam with retinal imaging, dilated fundus exam, or Amsler grid for retina checks. Even with perfect chart vision, silent retinal problems can exist

**Q34. How should I understand my diabetic eye screening report?**

**model: ChatGPT**

*answer:*

I can help explain your retinal screening report in detail, but I'll need to see the report first to give accurate guidance. Could you upload a copy of your retinal screening report? Once I can view it, I can break down key findings like:

Presence and severity of diabetic retinopathy

Macular edema or other retinal changes

Grading or risk levels

Recommended follow-up or treatment

This way, you'll get a clear, plain-language interpretation of what your results mean.

**model: Copilot**

*answer:*

The short version: your retinal screening report tells you whether the back of your eye (the retina) shows any signs of disease, especially conditions like diabetic retinopathy, macular edema, glaucoma, or age-related macular degeneration. To understand it, you need to decode the abbreviations, the severity scale, and the imaging findings.

Below is a clear, structured guide so you can interpret your own report line by line.

What your retinal screening report is actually telling you

Most reports include these sections:

1. Visual acuity

This is the "20/XX" line.

20/20 = normal

20/40 = you see at 20 ft what a normal person sees at 40 ft

OD = right eye

OS = left eye

2. Retinopathy status

If you have diabetes, the report will classify your retina using a standard scale:

No DR - completely normal

Mild NPDR - tiny microaneurysms only

Moderate NPDR - more bleeding, exudates, or cotton-wool spots

Severe NPDR - "4-2-1 rule": widespread bleeding, venous beading, or abnormal vessels

PDR - new abnormal blood vessels (dangerous, requires treatment)

3. Macular edema

This tells you whether there is swelling in the macula, the part responsible for sharp vision.

"No DME" = good

"DME present" = may require treatment

4. Other findings

Your report may list:

Cataract

Glaucoma

Age-related macular degeneration

Vitreous hemorrhage

Retinal detachment

5. Imaging results

Most modern screenings include:

Fundus photos - pictures of the retina

OCT - cross-section scan showing swelling, thinning, or fluid

Autofluorescence - highlights retinal pigment changes

Common abbreviations you'll see

OD - right eye

OS - left eye

OU - both eyes

BCVA - best corrected vision

CWS - cotton-wool spots

DME - diabetic macular edema

NPDR / PDR - non-proliferative / proliferative diabetic retinopathy

How to interpret your results in plain language

If your report says "No diabetic retinopathy"

Your retina is healthy. Continue annual exams.

If it says "Mild NPDR"

Tiny early changes. Usually no treatment needed; monitor.

If it says "Moderate NPDR"

More noticeable damage. Follow-up may be every 6-12 months.

If it says "Severe NPDR"

High risk of progression. Closer monitoring or referral to a retina specialist.

If it says "PDR"

New abnormal blood vessels. This is vision-threatening and usually requires treatment (laser or injections).

If it says "DME present"

Swelling in the macula. This affects central vision and often requires treatment.

What the retina looks like in these conditions

**model: DeepSeek**

*answer:*

That's an excellent question, as a retinal screening report (often done for diabetic retinopathy, macular degeneration, or glaucoma checks) can look intimidating.

First, a crucial disclaimer: I am an AI, not an ophthalmologist. The following is a guide to common terms and layout. You must discuss your specific results and their implications for your health with the doctor who ordered the test.

Here is a step-by-step guide to decoding the 4 main sections of a typical report.

1. The "Patient Details" & "Reason for Exam"

What to look for: Your diagnosis (e.g., "Type 2 Diabetes," "Hypertension," "Macular Drusen").

Why it matters: This tells the doctor what specific problems they are searching for. If this section is wrong, the rest of the report is useless.

2. The "Image Quality" Section (Often ignored, but critical)

Common terms: Adequate, Good, Poor, Cataract present, Blink artifacts, Media opacity.

How to interpret:

Good/Adequate: The doctor can trust what they see.

Poor / Artifacts: This means the image is blurry (due to cataracts, dry eye, or patient movement). A "normal" result on a poor quality image is worthless. You may need a different test (like an ultrasound or OCT).

3. The "Findings" Section (The core of the report)

This is where you will see jargon. Here is the translation:

If you see this term...; What it likely means...; Urgency?

No diabetic retinopathy (NDR); No damage from diabetes seen.; Routine follow-up.

Mild NPDR (Non-Proliferative DR); Small bleeds or ballooning spots (microaneurysms) inside the retina.; Monitor closely; control blood sugar.

Moderate/Severe NPDR; More bleeding or abnormal vessel shapes.; High risk; may need specialist visit soon.

PDR (Proliferative DR); New, abnormal blood vessels are growing. These can bleed suddenly.; Urgent/Emergent. Requires laser or injection.

Macular Edema; Fluid leaking into the central vision area (macula). Blurriness will occur.; Urgent. Requires treatment (injections/laser).

Drusen; Yellow deposits under the retina. Common in age-related macular degeneration (AMD).; Low if early; High if "large" or "soft."

Cupping / Large Cup-to-Disc ratio; The optic nerve head looks hollowed out. Suspicious for glaucoma.; Requires visual field test; may need drops.

Choroidal Nevus; A "freckle" in the back of the eye. Usually benign.; Monitor yearly for growth.

Vitreous hemorrhage; Blood floating in the jelly of the eye. Blocks vision.; Refer to retina specialist urgently.

4. The "Impression & Recommendations" (The most important section)

Ignore the medical jargon. Focus on the Action Item.

"Routine follow-up in 12 months" = Your eyes are currently healthy for your risk level.

"Repeat imaging in 6 months" = There are mild changes that need watching.

"Refer to Retina Specialist (Vitreoretinal surgeon)" = This is a yellow or red flag. You have a condition (edema, PDR, hole, tear) that a general optometrist or ophthalmologist cannot treat.

"OCT macula recommended" = The 2D photo looks suspicious, and they need a 3D cross-section scan to see if there is fluid.

3 Red Flags You Should Never Ignore

If your report contains any of these words, call your eye doctor immediately (do not wait for your next annual exam):

"Proliferative" or "Neovascularization" - New bad vessels.

"Macular edema" or "Intraretinal fluid" - Leaking in the center.

"Hole", "Tear", or "Detachment" - Emergency surgical conditions.

A Practical Example

Your report says:

"Image quality: Good. Findings: Mild NPDR with a small area of intraretinal fluid in the fovea. Impression: Clinically significant macular edema. Recommendation: Refer to retina for possible anti-VEGF injection."

Your translation:

"The pictures are clear. Diabetes has caused a small leak in the very center of my vision (the fovea). This is called macular edema. I need to see a retina specialist soon because they will likely need to give me a shot in my eye to stop the leak."

Final action steps for you:

Look at the "Image Quality." If it says "Poor," ask for a re-test.

Look for the words "Normal" or "No pathology." If you see those, you are fine.

If you see ANY other words (NPDR, Drusen, Cupping, Edema), do not Google symptoms - book a 15-minute call or appointment with your eye doctor to walk you through the specific meaning for your health history.

**model: Doubao**

*answer:*

I'll break down a standard retinal screening report in plain, professional English-key sections, normal vs abnormal findings, and what each term means.

1. Basic Report Structure

General Information: Eye side (OD = right eye, OS = left eye), age, screening type (fundus photography, OCT, digital retinal scan).

Retinal Background: Describes the retina, blood vessels, optic nerve, macula.

Key Anatomical Parts Explained

Optic Disc: Where the nerve exits the eye; checks for swelling, glaucoma damage.

Macula: Central retina for sharp vision; critical for reading/seeing faces.

Retinal Blood Vessels: Assess for narrowing, bleeding, leakage (linked to diabetes, hypertension).

Peripheral Retina: Checks for holes, tears, detachment risk.

2. Common Normal Descriptions

Optic disc: clear boundary, no edema

Macula: flat, normal foveal reflex, no edema/drusen

Vessels: normal caliber, no tortuosity, no hemorrhage/exudate

Retina: intact, no breaks, no detachmentMeaning: Your retina is healthy; routine follow-up only needed.

3. Common Abnormal Findings & What They Mean

Retinal Hemorrhage

Small bleeding spots on the retina; often linked to diabetes, high blood pressure.

Exudates (Hard/Soft)

Fatty or inflammatory deposits; classic sign of diabetic retinopathy or uncontrolled hypertension.

Macular Edema

Swelling in the central macula; causes blurred central vision, common in diabetes.

Drusen

Yellow deposits under the macula; age-related, can raise risk of macular degeneration.

Optic Disc Cupping/Atrophy

Larger cup size or nerve thinning; may indicate glaucoma or prior nerve damage.

Retinal Tear / Lattice Degeneration

Thinned or broken peripheral retina; needs monitoring or laser repair to prevent retinal detachment.

4. Screening Result Categories

Normal: No changes; repeat routine screening every 1-2 years.

Benign Changes (mild drusen, minor vessel changes): No immediate treatment; annual follow-up.

Mild/Moderate Retinopathy (diabetic/hypertensive): Lifestyle control + 6-month recheck.

Severe Changes (edema, large hemorrhage, tear): Refer to ophthalmologist for further OCT/laser/injection treatment.

5. Practical Tips for You

Always check OD/OS to match right/left eye.

If you have diabetes or high BP, even mild retinal changes need stricter sugar/blood pressure control.

A screening report is a screening, not a full diagnosis-abnormal results need a formal eye exam with an ophthalmologist.

**model: Gemini**

*answer:*

Understanding a retinal screening report can feel like trying to read a different language. Most reports use a mix of alphanumeric codes, anatomical terms, and color-coded maps.

Since these reports are most commonly associated with diabetic eye screening or general health checks using OCT (Optical Coherence Tomography), here is a breakdown of what the most frequent terms actually mean.

1. The Grading Codes (Common in Diabetic Screening)

If your report contains codes like R0, M0, or R1, it is using a standard grading system to describe the health of your retina and macula (the center of your vision).

Grade; What it Means; Typical Action

R0; No Retinopathy: No changes found.; Routine annual screening.

R1; Background Retinopathy: Tiny bulges (microaneurysms) in blood vessels.; Usually just monitored; doesn't affect sight yet.

R2; Pre-proliferative: More significant changes/leaks in the vessels.; More frequent monitoring or a specialist referral.

R3; Proliferative: New, weak blood vessels are growing (risky).; Urgent specialist appointment for treatment.

M0 / M1; Maculopathy: Indicates if the central vision (macula) is affected.; M0 is clear; M1 means changes that need an OCT scan.

2. Reading the OCT "Heat Map"

If your report has a colorful circle (often green, yellow, and red), this is a thickness map. It measures how thick your retina is in microns

Green: Normal thickness. The "Goldilocks zone."

Yellow/Red (Warm colors): The retina is thicker than average. This usually suggests swelling or fluid buildup (edema).

Blue/Purple (Cool colors): The retina is thinner than average. This can suggest "atrophy" or tissue loss, sometimes seen in glaucoma or advanced aging.

3. Common Anatomy & Abbreviations

Doctors often use shorthand for specific parts of the eye or findings:

Macula: The small area in the center of the retina responsible for sharp, detailed vision (reading, driving).

Exudates (HE): Small yellow spots of protein or lipid deposits. Think of them as "leaks" that have dried up.

Heme / Hemorrhage: Small spots of blood.

Drusen: Tiny yellow deposits under the retina-very common as people age, but monitored for Macular Degeneration (AMD).

CWS (Cotton Wool Spots): Small white "fluffy" patches indicating areas that aren't getting enough oxygen.

RNFL: Retinal Nerve Fiber Layer. This is the layer usually measured to check for signs of glaucoma.

4. What should you ask your doctor?

The report is a "snapshot" in time, but the trend is what matters most. At your next follow-up, consider asking:

1. "Are these results stable compared to my last scan?"

2. "Is the thickness (microns) within the normal range for my age?"

3. "If there are changes (like R1), what specific steps can I take with my blood pressure or glucose to keep it from progressing?"

4. Note: While these guides help you navigate the jargon, only your eye specialist can give a definitive diagnosis based on your full medical history. If you see R3 or M1 on your report and haven't heard from your doctor, it's worth giving their office a quick call.

**model: Perplexity**

*answer:*

Diabetic eye screening reports assess retina damage from diabetes, mainly checking for diabetic retinopathy (DR) and macular edema (DME). They use standardized grading to guide follow-up care.

Key Report Sections

Reports typically note your diabetes details (type, duration), pupil dilation, visual acuity, intraocular pressure, and retinal images or exam findings.

They list symptoms like blurred vision or floaters if present, plus non-DR issues such as dry eyes or cataracts.

Risk factors like blood pressure, cholesterol, and diabetes control (A1C) are often summarized to stress management.

Retinopathy Grades

Common UK/NHS-style codes classify DR severity:

Grade Meaning Action

R0/No DR Normal retina, no changes Annual screening

R1/Background Minor vessel changes; no vision threat Annual screening; optimize diabetes control

R2/Pre-proliferative Moderate vessel issues; higher risk 3-9 month checks

R3/Proliferative Severe new vessel growth; sight-threatening Urgent specialist referral (within 6 weeks)

Treated stable Prior treatment holding Frequent monitoring

Maculopathy Grades

These flag central vision issues:

Grade Meaning Action

M0 No macula changes Matches retinopathy follow-up

M1 (low/high risk) Swelling or leaks in macula Closer scans (OCT) or specialist referral (13 weeks)

Next Steps

"No issues" means routine annual checks; abnormalities trigger referrals to ophthalmologists for lasers, injections, or surgery.

Discuss with your doctor for personalized advice, and control blood sugar/BP to slow progression.

If images were unclear (grade U), expect re-screening soon

**Q35. When does diabetic retinopathy need treatment?**

**model: ChatGPT**

*answer:*

Diabetic retinopathy (DR) needs treatment primarily when it reaches stages that threaten vision. Here's a clear breakdown:

1. Proliferative Diabetic Retinopathy (PDR)

1. Characterized by new, abnormal blood vessel growth (neovascularization) on the retina or optic disc.

2. Treatment is indicated because these vessels can bleed into the vitreous (causing sudden vision loss) or cause retinal detachment.

3. Common treatments: Panretinal photocoagulation (laser therapy) or anti-VEGF injections.

2. Diabetic Macular Edema (DME)

1. Swelling in the central retina (macula), leading to blurry or distorted vision.

2. Treatment is indicated when the macular edema is clinically significant (affecting vision).

3. Common treatments: Anti-VEGF injections, focal/grid laser therapy, or corticosteroid injections.

3. Severe Non-Proliferative Diabetic Retinopathy (NPDR)

1. Characterized by extensive retinal hemorrhages, venous beading, or intraretinal microvascular abnormalities.

2. Treatment may be considered prophylactically to prevent progression to PDR, especially if high-risk features are present.

4. Other considerations:

1. Vision-threatening DR always warrants prompt treatment.

2. Regular monitoring is essential for mild/moderate NPDR, as early intervention can prevent progression.

3. Systemic control (blood sugar, blood pressure, cholesterol) is crucial alongside ocular treatment.

In short, treatment is needed when DR threatens central vision or there is a high risk of severe vision loss, mainly in PDR or clinically significant DME.

**model: Copilot**

*answer:*

Treatment for diabetic retinopathy is needed when the disease reaches stages where vision is threatened - most commonly when diabetic macular edema (DME) or proliferative diabetic retinopathy (PDR) develops. Mild or moderate early disease often does not require immediate treatment but does require close monitoring.

When Treatment Is Needed

1. Diabetic macular edema (DME)

Treatment is recommended as soon as macular swelling is detected, because this is the most common cause of vision loss in diabetes.

Caused by fluid leaking into the macula

Leads to blurry or distorted central vision

Treated with anti-VEGF injections, steroids, or focal laser

2. Proliferative diabetic retinopathy (PDR)

Treatment is always required once abnormal new blood vessels begin growing.

These vessels bleed easily to floaters, vision loss

Can cause retinal detachment

Treated with panretinal laser, anti-VEGF injections, or vitrectomy

3. Vitreous hemorrhage or retinal detachment

These are emergencies.

Often require vitrectomy surgery

When Treatment Is Not Needed Yet

4. Mild or moderate NPDR

No immediate treatment if there is no macular edema

Requires regular dilated exams and tight glucose/BP control

Quick Decision Table

Stage; Vision Threat?; Treatment Needed?; Typical Treatment

Mild NPDR; Low; No; Monitoring + diabetes control

Moderate NPDR; Moderate; Sometimes; Treat if DME present

Severe NPDR; High; Often; Anti-VEGF or laser if edema or ischemia

DME; High; Yes; Anti-VEGF, steroids, focal laser

PDR; Very high; Yes; Panretinal laser, anti-VEGF, vitrectomy

Symptoms That Mean You Should Seek Care Immediately

Sudden floaters or "cobwebs"

Sudden blurry vision

Dark curtain over vision

Sudden vision loss

**model: DeepSeek**

*answer:*

Diabetic retinopathy requires treatment when it reaches a stage where vision is at significant risk. In general, treatment is needed for:

Proliferative Diabetic Retinopathy (PDR) - the advanced stage where abnormal new blood vessels grow on the retina or optic nerve. These vessels can bleed, causing vitreous hemorrhage or retinal detachment.

Diabetic Macular Edema (DME) - when fluid leaks into the macula (the central part of the retina responsible for sharp vision), causing blurred or distorted central vision. Not all DME requires immediate treatment, but clinically significant DME with vision decline does.

High-risk features on exam - even without vision symptoms, findings like large areas of non-perfusion, recurrent hemorrhages, or neovascularization on fluorescein angiography may prompt treatment.

Key point: Early-stage NPDR (non-proliferative diabetic retinopathy) without macular edema usually does not require laser, injection, or surgery. Instead, the main treatment is strict glycemic, blood pressure, and lipid control plus regular monitoring.

Current treatment options include:

Anti-VEGF injections (e.g., aflibercept, ranibizumab, bevacizumab) - first-line for DME and PDR.

Panretinal photocoagulation (laser) - mainly for PDR if injections are not feasible.

Vitrectomy - for non-clearing vitreous hemorrhage or tractional retinal detachment.

When to start: An eye care specialist determines based on severity, location of pathology, and visual symptoms. Regular dilated eye exams (at least yearly; more often if retinopathy is present) are essential to catch treatable disease early.

**model: Doubao**

*answer:*

1. Non-proliferative Diabetic Retinopathy (NPDR)

Mild NPDR: Usually no immediate treatment; regular screening & tight blood sugar/blood pressure control only.

Moderate to Severe NPDR:

Presence of macular edema (central retinal thickening) to requires active treatment.

Significant retinal ischemia, extensive microaneurysms, hemorrhages, exudates to early intervention & close monitoring.

2. Diabetic Macular Edema (DME)

Any DME affecting or threatening the central macular vision is an absolute indication for treatment, regardless of DR stage.

3. Proliferative Diabetic Retinopathy (PDR)

Once neovascularization (abnormal new retinal blood vessels) appears:

Urgent treatment is needed, as it easily causes vitreous hemorrhage, retinal detachment, and permanent blindness.

High-risk PDR: requires immediate laser or intravitreal injection.

4. Complications requiring immediate intervention

Vitreous hemorrhage

Tractional retinal detachment

Neovascular glaucoma

Refractory macular edema unresponsive to lifestyle/metabolic control

First-line treatments when indicated

Intravitreal anti-VEGF injections (first choice for DME & neovascularization)

Focal/grid laser photocoagulation

Panretinal laser photocoagulation (for high-risk PDR)

Vitrectomy surgery (for severe hemorrhage / retinal detachment)

**model: Gemini**

*answer:*

Determining the right time for treatment depends heavily on the stage of the disease and whether your vision is currently at risk. While early stages are often managed with monitoring, advanced stages require active intervention to prevent permanent vision loss.

Medical guidelines generally categorize the need for treatment into three main scenarios:

1. Proliferative Diabetic Retinopathy (PDR)

This is the advanced stage where the retina starts growing new, abnormal blood vessels (neovascularization). These vessels are fragile and prone to leaking or bleeding into the eye.

When to treat: Immediately upon diagnosis. Because PDR carries a high risk of sudden blindness from vitreous hemorrhage (bleeding) or retinal detachment, doctors typically recommend starting treatment (such as laser therapy or injections) within 4 to 6 weeks.

2. Diabetic Macular Edema (DME)

This occurs when fluid leaks into the macula, the part of the retina responsible for sharp, central vision.

When to treat: When it becomes "clinically significant." If swelling reaches the center of the macula and starts blurring your vision, treatment is usually required.

Primary Treatments: Anti-VEGF injections (like Eylea or Lucentis) are the first-line therapy to reduce swelling and improve vision.

3. Severe Non-Proliferative Retinopathy (NPDR)

In this "pre-proliferative" stage, the blood vessels are significantly damaged but new ones haven't started growing yet.

When to treat: Treatment at this stage is a preventative judgment call. Your ophthalmologist may recommend early injections or laser treatment if they believe there is a very high "4-2-1 rule" risk (specific patterns of hemorrhages and vessel changes) that you will progress to the proliferative stage within a year.

Summary of Treatment Triggers

Condition; Action Required; Typical Treatment

Mild/Moderate NPDR; Monitoring & Blood Sugar Control; Observation every 6-12 months

Severe NPDR; Close Monitoring or Preventative; Possible injections or laser every 3-4 months

Macular Edema (DME); Active Treatment; Anti-VEGF injections or Focal laser

Proliferative (PDR); Urgent Treatment; Panretinal laser (PRP) or Injections

When is surgery (Vitrectomy) needed?

Surgery is usually reserved for the most advanced complications that cannot be fixed by shots or lasers, such as:

A vitreous hemorrhage (blood in the eye) that does not clear on its own within a few months.

A tractional retinal detachment (scar tissue pulling the retina away).

Note: If you notice sudden "floaters," dark spots, or a "curtain" over your vision, these are signs that retinopathy may have progressed and require an immediate exam.

**model: Perplexity**

*answer:*

Diabetic retinopathy treatment timing depends on its stage and severity. Early stages often require only monitoring, while advanced cases need prompt intervention to prevent vision loss.

Early Stages

Mild or moderate nonproliferative diabetic retinopathy typically does not need immediate treatment. Instead, doctors recommend close monitoring through regular eye exams and optimizing blood sugar control to slow progression. Good diabetes management can often prevent worsening in these phases.

Advanced Stages

Treatment is essential for proliferative diabetic retinopathy or macular edema. Options include anti-VEGF injections into the eye, laser photocoagulation, or surgery like vitrectomy, depending on the retinal damage. These interventions aim to stop abnormal blood vessel growth and reduce swelling.

Monitoring Guidelines

All people with diabetes should get annual dilated eye exams, with more frequent checks (every 3-12 months) if high risk or early changes appear. Even after treatment, lifelong follow-ups are necessary.

**Q36. What treatments are available for diabetic retinopathy?**

**model: ChatGPT**

*answer:*

Diabetic retinopathy (DR) treatment depends on the stage and severity of the disease. Here are the main options:

1. Blood Sugar, Blood Pressure, and Lipid Control

1. Tight control of blood glucose, blood pressure, and cholesterol can slow the progression of DR.

2. Medications like anti-hypertensives and statins may be used as needed.

2. Laser Therapy (Photocoagulation)

1. Focal/grid laser: Targets specific leaking blood vessels to reduce macular edema.

2. Panretinal photocoagulation (PRP): Used for proliferative DR to shrink abnormal new blood vessels and prevent bleeding.

3. Anti-VEGF Injections

1. Medications such as ranibizumab, aflibercept, and bevacizumab are injected into the eye to reduce macular edema and inhibit abnormal vessel growth.

2. Typically given every 4-12 weeks, depending on response.

4. Corticosteroid Injections or Implants

1. Steroids like triamcinolone or dexamethasone implants may be used for persistent diabetic macular edema, especially if anti-VEGF therapy is insufficient.

5. Vitrectomy Surgery

1. Considered for advanced proliferative DR with vitreous hemorrhage or tractional retinal detachment.

2. The surgeon removes the vitreous gel and may repair retinal detachments.

6. Regular Monitoring

1. Even with treatment, ongoing eye exams are essential to detect progression early.

Early stages of DR often do not require invasive treatment and can be managed with close monitoring and systemic disease control. Interventions are usually reserved for diabetic macular edema or proliferative changes that threaten vision.

**model: Copilot**

*answer:*

The main treatments for diabetic retinopathy focus on slowing or stopping retinal damage. The right approach depends on whether the disease is in the non-proliferative (NPDR) or proliferative (PDR) stage, and whether diabetic macular edema (DME) is present. Treatments fall into four major categories: anti-VEGF injections, steroid injections, laser therapy, and vitrectomy surgery.

1. Anti-VEGF Eye Injections

These are the first-line treatment for diabetic macular edema and often for proliferative disease.

Anti-VEGF medications - ranibizumab (Lucentis), aflibercept (Eylea), faricimab (Vabysmo), and off-label bevacizumab (Avastin).

They reduce abnormal blood vessel growth and decrease retinal swelling.

Usually require monthly injections initially, then less often as the eye stabilizes.

Side effects may include temporary discomfort, pressure rise, or rare infection.

2. Steroid Injections or Implants

Used when anti-VEGF is insufficient or not tolerated.

Steroid therapy reduces inflammation and macular swelling.

Options include triamcinolone injections or long-acting implants (e.g., dexamethasone).

Higher risk of cataracts and increased eye pressure.

3. Laser Photocoagulation Therapy

Laser treatment is used for both NPDR and PDR, especially when blood vessels leak or new vessels grow.

Focal/grid laser seals leaking vessels in macular edema.

Pan-retinal photocoagulation (PRP) shrinks abnormal new vessels in PDR.

Helps prevent severe vision loss but may reduce night or peripheral vision.

4. Vitrectomy Surgery

A surgical option for advanced disease.

Vitrectomy removes blood from vitreous hemorrhage, scar tissue, or repairs retinal detachment.

Often combined with laser treatment during surgery.

Performed in a hospital or surgical center under anesthesia.

5. Systemic Management (Always Required)

Even with eye treatments, tight diabetes control is essential.

Blood sugar management slows progression.

Control of blood pressure and cholesterol protects retinal vessels.

Regular eye exams detect changes early.

Summary Table

Treatment; Purpose; Best For; Key Considerations

Anti-VEGF injections; Reduce swelling, stop new vessel growth; DME, PDR; Most common first-line therapy

Steroid injections; Reduce inflammation & edema; DME not responding to anti-VEGF; Risk of cataracts & high eye pressure

Laser therapy; Seal leaks, shrink abnormal vessels; NPDR with leaks, PDR; May reduce night/peripheral vision

Vitrectomy; Remove blood/scar tissue, fix detachment; Advanced PDR; Surgical risks; used when other treatments insufficient

**model: DeepSeek**

*answer:*

The treatment for diabetic retinopathy depends heavily on the stage of the disease-whether it is in the early non-proliferative stage or the more advanced proliferative stage. The primary goals are to slow or stop the progression of the disease to prevent severe vision loss.

Here is a breakdown of the available treatments:

1. Observation and Systemic Control (For Early/Mild Cases)

In the early stages (mild to moderate non-proliferative diabetic retinopathy), immediate surgical or injectable treatment may not be required. Instead, the focus is on close monitoring and controlling underlying health conditions to slow the disease's progression.

Glycemic Control: Intensively managing blood sugar levels is proven to prevent and delay the onset and progression of diabetic retinopathy.

Blood Pressure and Lipid Control: Managing hypertension and cholesterol can also reduce the risk of progression.

Medication: For people with type 2 diabetes and non-proliferative retinopathy, doctors may consider prescribing fenofibrate (a lipid-lowering drug) specifically to reduce the progression of retinopathy.

Monitoring Schedule: If you have moderate NPDR, you might be seen every 6 to 12 months; if it is severe or very severe, monitoring may be every 3 to 6 months.

2. Anti-VEGF Injections (For Advanced or Leaking Eyes)

These are medications injected directly into the vitreous (gel-like fluid) of the eye. They work by blocking the vascular endothelial growth factor (VEGF) protein, which halts the growth of abnormal blood vessels and reduces fluid leakage (macular edema).

Use: This is the primary treatment for Diabetic Macular Edema (DME) and is also a powerful option for treating proliferative diabetic retinopathy (PDR).

FDA-Approved Drugs for DME: Ranibizumab (Lucentis), Aflibercept (Eylea), and Faricimab (Vabysmo). Bevacizumab (Avastin) is often used "off-label".

Procedure: Requires multiple injections over time. Research shows that about one-third of patients may eventually need additional treatments like laser or surgery.

3. Laser Therapy (Panretinal Photocoagulation)

This treatment uses laser beams to create burns on the periphery of the retina.

Use: It is the standard first-line treatment for most people when first diagnosed with PDR. It helps shrink abnormal blood vessels and prevents them from bleeding.

Timeline: Guidelines recommend starting treatment within 4 weeks of diagnosis and completing it within 4 weeks of starting.

Effect: While it preserves central vision, it may result in some loss of peripheral (side) vision or night vision.

4. Vitrectomy Surgery (For Advanced Bleeding)

This is a surgical procedure performed in a hospital where small tools are inserted into the eye to remove blood and scar tissue.

Use: Reserved for advanced PDR, specifically for:

Vitreous hemorrhage (bleeding in the eye) that does not clear on its own within 3 months.

Retinal detachment caused by scar tissue.

Summary of Treatment by Disease Stage

Condition; Primary Treatment Goals; Common Approaches

Mild/Moderate NPDR; Slow progression; preserve vision; Observation, blood sugar/blood pressure control, Fenofibrate (for type 2)

Diabetic Macular Edema (DME); Reduce swelling; improve vision; Anti-VEGF injections (first line). Laser or steroids if injections are not effective

Proliferative DR (PDR); Regress abnormal vessels; prevent bleeding; Panretinal laser (first line) or Anti-VEGF injections

Advanced PDR (Hemorrhage); Clear blood; reattach retina; Vitrectomy surgery

It is important to note that while these treatments are very effective at stopping vision from getting worse, they are not a cure for diabetes-related eye disease. You will need lifelong monitoring

**model: Doubao**

*answer:*

Treatment options depend on the stage of diabetic retinopathy (non-proliferative vs proliferative) and presence of macular edema.

1. Intravitreal Injections (First-line for Diabetic Macular Edema)

Anti-VEGF agents: Ranibizumab, Aflibercept, Bevacizumab

Steroid implants/injections: Dexamethasone implant, Fluocinolone acetonideIndicated for macular edema causing vision loss.

2. Laser Photocoagulation

Focal/grid laser: Treats localized diabetic macular edema

Panretinal photocoagulation (PRP): For proliferative diabetic retinopathy to shrink abnormal new blood vessels and reduce blindness risk.

3. Vitreoretinal Surgery (Pars Plana Vitrectomy)

Used for advanced complications:

Severe vitreous hemorrhage

Tractional retinal detachment

Epiretinal membrane

Refractory macular edema unresponsive to injections/laser

4. Systemic Management (Foundational Treatment)

Strict control to slow disease progression:

Blood glucose (HbA1c)

Blood pressure

Lipid levels

Regular diabetic eye screening (annual or more frequent per severity)

5. Emerging Therapies

New anti-VEGF long-acting formulations

Neuroprotective agents

Gene therapy and stem cell research (under clinical trials)

**model: Gemini**

*answer:*

Managing diabetic retinopathy (DR) involves a combination of systemic management and localized ocular therapies, with the choice of treatment depending largely on the severity of the disease and the presence of Diabetic Macular Edema (DME) (Al-Zidi et al., 2022; Solomon et al., 2017).

Systemic Management (Prevention and Early Stages)

For early-stage non-proliferative diabetic retinopathy (NPDR), the primary goal is to slow progression through metabolic control (Al-Zidi et al., 2022; Solomon et al., 2017).

Glycemic Control: Maintaining near-normal blood glucose levels significantly reduces the risk of DR onset and progression (Solomon et al., 2017).

Blood Pressure Regulation: Lowering blood pressure helps decrease retinopathy progression, with typical targets being below 140/90 mmHg (Solomon et al., 2017).

Lipid Management: In patients with dyslipidemia, adding fenofibrate has been shown to slow the progression of retinopathy, particularly in mild NPDR (Al-Zidi et al., 2022; Solomon et al., 2017).

Pharmacological Therapies

Intravitreal injections are now the primary treatment for advanced DR and center-involved DME (Solomon et al., 2017).

Anti-VEGF Therapy: These agents block vascular endothelial growth factor to stop abnormal vessel growth and reduce leakage.

Approved Agents: Ranibizumab, Aflibercept, Brolucizumab, and Faricimab (Hernandez-Ruiz et al., 2023).

Off-label Use: Bevacizumab is frequently used due to its cost-effectiveness (Hernandez-Ruiz et al., 2023).

Corticosteroids: Used primarily when anti-VEGF therapy is insufficient. Options include Dexamethasone and Fluocinolone acetonide implants, which provide extended-release treatment (Al-Zidi et al., 2022).

Procedural and Surgical Treatments

Laser Photocoagulation:

Panretinal Photocoagulation (PRP): The traditional standard for proliferative diabetic retinopathy (PDR), used to shrink abnormal blood vessels. It can cause permanent peripheral or night vision loss (Al-Zidi et al., 2022; Hernandez-Ruiz et al., 2023).

Focal/Grid Laser: Used to treat localized areas of swelling in the macula (Hernandez-Ruiz et al., 2023).

Vitrectomy: A surgical procedure to remove vitreous gel clouded by hemorrhage or to repair tractional retinal detachment caused by scar tissue (Al-Zidi et al., 2022).

Emerging and Durable Therapies

To reduce the burden of frequent injections (often monthly), research is focused on long-acting delivery systems:

OTX-TKI (AXPAXLITM): An investigational tyrosine kinase inhibitor hydrogel designed for once- or twice-yearly dosing, currently in Phase 3 trials for NPDR (HCPLive, 2026).

Bispecific Antibodies: Agents like Faricimab target both VEGF and Ang-2 pathways, potentially offering longer intervals between treatments (Hernandez-Ruiz et al., 2023).

**model: Perplexity**

*answer:*

Diabetic retinopathy treatments focus on slowing progression, managing complications like macular edema, and preserving vision.

Main Treatments

Anti-VEGF injections, such as ranibizumab (Lucentis), aflibercept (Eylea), and faricimab (Vabysmo), are first-line therapies for diabetic macular edema (DME) and proliferative diabetic retinopathy (PDR). These drugs reduce abnormal blood vessel growth and fluid buildup, often requiring repeat doses.

Laser Therapy

Laser photocoagulation seals leaky vessels or shrinks new ones, halving severe vision loss risk in PDR cases. It's used alone or with injections, especially for high-risk PDR.

Steroid Options

Intravitreal steroids like triamcinolone, dexamethasone implants (Ozurdex), or fluocinolone implants (Iluvien) treat swelling when anti-VEGF isn't enough. They carry risks like elevated eye pressure.

Surgery

Vitrectomy removes blood or scar tissue in advanced cases. It's reserved for tractional retinal detachment or non-clearing vitreous hemorrhage.

Supportive Measures

Tight blood glucose, blood pressure, and lipid control are foundational. Fenofibrate may slow non-proliferative DR progression by 30%

**Q37. What are eye injections for diabetic retinopathy, and when might I need them?**

**model: ChatGPT**

*answer:*

Eye injections for diabetic retinopathy are treatments where medication is delivered directly into the vitreous (the gel-like center) of the eye. These injections are designed to slow or stop vision loss caused by diabetic retinopathy and diabetic macular edema, which is swelling of the retina due to fluid leakage from damaged blood vessels.

Common Medications Used

1. Anti-VEGF drugs - These block vascular endothelial growth factor, a protein that promotes abnormal blood vessel growth and leakage. Examples include:

1. Aflibercept

2. Ranibizumab

3. Bevacizumab (used off-label)

2. Steroid injections - Reduce inflammation and swelling in the retina, sometimes used if anti-VEGF drugs aren't effective.

When Eye Injections Might Be Needed

You might need these injections if you have:

Diabetic macular edema (DME) causing swelling in the central retina and vision loss.

Proliferative diabetic retinopathy (PDR), where abnormal blood vessels grow and threaten vision.

Progressive vision loss despite controlling blood sugar, blood pressure, and cholesterol.

Before or after laser treatments, in some cases, to reduce swelling and improve outcomes.

Treatment Frequency

Initially, injections may be given every 4-8 weeks, depending on response.

Over time, the interval may be extended if the eye stabilizes.

Eye injections are generally safe but can have risks, such as infection, bleeding, or increased eye pressure, so they are performed under strict sterile conditions.

**model: Copilot**

*answer:*

Eye injections for diabetic retinopathy are treatments that deliver medicine directly into the eye to slow or stop vision loss. They're most often used when diabetes has caused leaking blood vessels, swelling in the retina, or abnormal new blood vessel growth. These injections are one of the most effective ways to protect vision in moderate to advanced stages of the disease.

What eye injections are

The two major types are:

1. Anti-VEGF injections

These medicines block VEGF, a protein that causes abnormal blood vessels to grow and leak in diabetic retinopathy. Common drugs include Avastin, Lucentis, Eylea, Beovu, and Vabysmo. They help:

Reduce swelling in the retina

Stop abnormal vessel growth

Improve or stabilize vision

Anti-VEGF injections are the first-line treatment for diabetic macular edema and proliferative diabetic retinopathy.

2. Steroid injections

Used when swelling is driven by inflammation or when anti-VEGF drugs aren't enough. Examples: Triesence, Kenalog, or long-acting implants like Ozurdex or Iluvien. They reduce inflammation and retinal swelling but can raise the risk of glaucoma or cataracts.

When you might need eye injections

Your eye doctor may recommend injections if you have:

Diabetic macular edema

Swelling in the macula (the part of the retina responsible for sharp vision). This is the most common reason for injections.

Proliferative diabetic retinopathy

A more advanced stage where fragile new blood vessels grow and bleed. Anti-VEGF injections help stop this abnormal growth.

Persistent or worsening vision changes

If your retina specialist sees:

Leaking blood vessels

Retinal swelling

New vessel growth

Bleeding inside the eye

...injections may be needed to prevent permanent vision loss.

When laser treatment alone isn't enough

In some cases, injections are used before or along with laser therapy to improve outcomes.

What the procedure is like

Your eye is numbed with drops or gel

The surface is cleaned to prevent infection

A tiny needle places medicine into the vitreous (center of the eye)

Most people feel pressure but not pain

Vision may be blurry for a few hours

How often injections are needed

Most people start with monthly injections, then gradually extend to every 6-12 weeks if the eye stabilizes. Some people eventually stop; others need long-term treatment to protect vision.

Risks and side effects

Most are mild and temporary:

Irritation or scratchy feeling

Redness

Small spot of blood on the eye

Floaters

Serious complications are rare, but call your doctor immediately if you have:

Worsening vision

Severe pain

Increasing floaters

Light sensitivity These can signal infection.

Should you consider injections?

You should discuss injections with your eye specialist if you have:

Blurry or distorted vision

Swelling in the retina on OCT imaging

Bleeding or new vessel growth

Moderate to severe diabetic retinopathy

Early treatment is one of the strongest predictors of preserving vision.

**model: DeepSeek**

*answer:*

This is an excellent and important question. Eye injections are a common and highly effective treatment for advanced diabetic retinopathy. Let's break down what they are, how they work, and when you might need them.

First, a quick reminder: Diabetic retinopathy damages the blood vessels in your retina (the light-sensitive tissue at the back of your eye). In its early stages, it might not need injections. But as it progresses, two sight-threatening problems can develop:

Diabetic Macular Edema (DME): Damaged, leaky vessels cause fluid to build up in the macula (the central part of the retina responsible for sharp, straight-ahead vision). This causes blurry vision.

Proliferative Diabetic Retinopathy (PDR): The retina becomes starved for oxygen, so it grows abnormal, fragile new blood vessels. These vessels can bleed easily into the eye, causing floaters, severe vision loss, and even retinal detachment.

Eye injections treat both of these advanced stages.

What exactly are these eye injections?

The injections are not a "one-size-fits-all" drug. They usually contain one of two types of medication, delivered directly into the vitreous (the jelly-like fluid in the center of the eye). The procedure is called an intravitreal injection.

The two main types are anti-VEGF drugs and steroids.

1. Anti-VEGF Drugs (The most common treatment)

What they are: Medications like Eylea (aflibercept), Lucentis (ranibizumab), Avastin (bevacizumab), or Vabysmo (faricimab).

How they work: VEGF stands for Vascular Endothelial Growth Factor. It's a protein your body makes that stimulates the growth of new, leaky blood vessels. In PDR and DME, the retina is producing too much VEGF. Anti-VEGF drugs block this protein, acting like a chemical "pruning shears". They stop new abnormal vessels from growing, shrink existing ones, and stop the leakage that causes macular edema.

Effectiveness: Excellent. They are the gold standard for treating both DME and PDR. Vision often stabilizes or improves.

2. Steroid Injections (A second-line option)

What they are: Medications like Ozurdex (dexamethasone implant) or Iluvien (fluocinolone acetonide implant). These are slow-release pellets placed in the eye.

How they work: Steroids are powerful anti-inflammatory drugs. They reduce the swelling (edema) in the macula by calming the overall inflammatory process that contributes to leaky vessels.

When used: Typically for DME that hasn't responded well enough to anti-VEGF drugs, or when a patient cannot get frequent anti-VEGF injections. Steroids have more significant side effects (see below), so they are not usually the first choice.

The Procedure: What actually happens?

The thought is scary, but the procedure is quick, routine, and generally well-tolerated.

Numbing: You get anesthetic eye drops. You will not feel the injection itself.

Cleaning: The eye surface is thoroughly disinfected with an iodine solution (looks like yellow water).

The Injection: Using a tiny, ultra-fine needle, the doctor injects the medicine through the white part of your eye (sclera) into the vitreous. It takes about 2-3 seconds. You might see a brief shadow or feel a tiny bit of pressure, but not "pain."

Afterward: Your vision may be blurry or you might see floating bubbles for a few hours. You will be given instructions (usually antibiotic drops for a few days). You cannot rub your eye.

When Might You Need Eye Injections?

Not everyone with diabetes needs injections. Your eye doctor makes the decision based on a comprehensive dilated eye exam, often using OCT (Optical Coherence Tomography) to see fluid in the retina. You might need injections if you have:

For Diabetic Macular Edema (DME):

Symptoms: Vision is blurry, wavy, or colors look washed out, specifically in your central vision.

Exam finding: The OCT scan shows clear fluid swelling in the macula that is threatening your reading/driving vision.

For Proliferative Diabetic Retinopathy (PDR):

Exam finding: Your doctor sees those abnormal, fragile new blood vessels growing on the retina, even if you have no symptoms yet. Injections are often the preferred first treatment for PDR today, having largely replaced laser (panretinal photocoagulation) in many cases.

Symptoms: You notice a sudden increase in floaters (look like cobwebs or specks), dark streaks, or a curtain over your vision (this indicates bleeding inside the eye).

What are the risks?

While common and safe, no procedure is risk-free.

Common (Minor): Subconjunctival hemorrhage (a red spot on the white of the eye, like a bruise), temporary eye irritation, floaters.

Serious (Rare): Eye infection (endophthalmitis), retinal detachment, increased eye pressure. Your doctor will give you strict rules to watch for signs of infection (pain, worsening redness, light sensitivity).

Steroid-Specific Risks: High eye pressure (glaucoma) in about 30% of patients, and almost guaranteed faster development of cataracts.

Important Note on Treatment Schedule

These are not one-time shots. The medication wears off.

Initial phase: You will likely get injections every 4-8 weeks.

Maintenance phase: After the disease is controlled, you might go to injections every 8-16 weeks, or switch to a "treat-and-extend" plan where the appointment lengthens as long as the eye stays dry.

The Bottom Line

Eye injections are a targeted, life-saving treatment for vision-threatening diabetic retinopathy. They are not for early-stage disease (which is managed with better blood sugar, blood pressure, and cholesterol control).

You should strongly consider eye injections if your doctor diagnoses you with:

Center-involved Diabetic Macular Edema (DME) causing vision loss.

Proliferative Diabetic Retinopathy (PDR), especially if there is high-risk bleeding.

The most important step is a dilated eye exam at least once a year (more often if you already have retinopathy). Only that exam can determine if you need treatment.

Immediately contact your eye doctor if you have:

New, sudden blurry vision or a dark spot in your center of vision.

A sudden shower of new floaters or flashes of light.

A dark curtain or shadow blocking any part of your vision.

**model: Doubao**

*answer:*

Eye Injections for Diabetic Retinopathy: What They Are & When You Need Them

What are the eye injections?

These are intravitreal injections-medicines injected directly into the gel (vitreous) inside your eye, the standard first-line treatment for advanced diabetic retinopathy (DR) and diabetic macular edema (DME).

The main medication classes:

Anti-VEGF agents

Common drugs: Aflibercept, Ranibizumab, Bevacizumab

How they work: Block vascular endothelial growth factor (VEGF), which causes abnormal blood vessel growth and fluid leakage in the retina. They reduce swelling, stop bleeding, and slow vision loss.

Steroid implants/injections

Examples: Dexamethasone implant, Fluocinolone acetonide

How they work: Reduce retinal inflammation and long-term macular swelling; used if anti-VEGF doesn't work well or for persistent edema.

When do you need these injections?

You will typically need intravitreal injections if you have any of these:

Diabetic Macular Edema (DME)Fluid builds up in the macula (central retina), causing blurry central vision-the most common reason for injections.

Proliferative Diabetic Retinopathy (PDR)Advanced DR with abnormal new blood vessels growing on the retina; these vessels can bleed into the eye or cause retinal detachment. Injections shrink abnormal vessels and prevent severe vision loss/blindness.

Macular involvement from DREven mild-moderate DR if macular swelling is present.

After diabetic eye complicationsRecurrent vitreous hemorrhage, or before/after retinal laser surgery to control edema and vessel growth.

Key signs you may need evaluation + possible injections

Gradual blurry central vision, difficulty reading

Dark spots, floaters in vision

Distorted vision (straight lines look bent)

Sudden vision loss from eye bleeding

Quick note on treatment pattern

Injections are not one-time: most patients need initial monthly loading doses, then regular follow-up injections every 1-3 months long-term to maintain vision.

**model: Gemini**

*answer:*

Diabetic retinopathy occurs when high blood sugar levels damage the tiny blood vessels in the retina. In response, the eye may grow new, fragile blood vessels that leak fluid or cause swelling. Eye injections, often called intravitreal injections, are a primary treatment to manage these changes and prevent vision loss.

Types of Injections

There are two main classes of medications used:

Anti-VEGF Agents: These are the most common. They block a protein called "vascular endothelial growth factor" (VEGF). High levels of VEGF cause the growth of abnormal, leaky blood vessels. By blocking it, these injections help shrink those vessels and reduce swelling.

Corticosteroids (Steroids): These are used to reduce inflammation and swelling in the retina. They may be used if Anti-VEGF treatments aren't providing enough of a response.

When They Are Needed

An ophthalmologist (specifically a retina specialist) typically recommends injections when the condition reaches specific stages:

1. Diabetic Macular Edema (DME): This is the most common reason. It occurs when fluid leaks into the macula (the part of the retina responsible for sharp, central vision), causing it to swell and blur your vision.

2. Proliferative Diabetic Retinopathy (PDR): This is an advanced stage where new, abnormal blood vessels begin to grow on the surface of the retina. Injections help shrink these vessels to prevent them from bleeding into the eye (vitreous hemorrhage) or causing a retinal detachment.

3. Preventing Progression: In some cases, injections are used in "severe non-proliferative" stages to stop the disease from advancing to the more dangerous proliferative stage.

The Procedure

The idea of a needle near the eye can be intimidating, but the process is designed to be as comfortable as possible:

Numbing: The eye is thoroughly numbed using drops or a small local anesthetic.

Cleaning: The eye and eyelids are cleaned with an antiseptic (like iodine) to prevent infection.

The Injection: The medication is injected through the "white" of the eye (the sclera) using a very fine needle. Most patients feel a sensation of pressure rather than a sharp pain.

Frequency: These are rarely a one-time fix. Most people require a "loading dose" (one injection a month for several months) followed by a maintenance schedule based on how the eye responds.

Important Note: If you notice sudden "floaters," flashes of light, or a dark curtain over your vision, you should contact an eye care professional immediately, as these can be signs of advanced retinopathy or retinal issues.

**model: Perplexity**

*answer:*

Eye injections treat diabetic retinopathy by delivering medications like anti-VEGF drugs (e.g., Lucentis, Eylea, Avastin) or steroids directly into the eye's vitreous gel. These block abnormal blood vessel growth and leakage, reducing swelling (especially diabetic macular edema) and slowing vision loss.

When Needed

You might need them for advanced non-proliferative or proliferative diabetic retinopathy, particularly with macular edema or leaking vessels threatening vision. They're often first-line over laser for immediate issues like hemorrhages, starting monthly until vision stabilizes, then less often.

Procedure Details

Done in-office with numbing drops, the quick injection uses a fine needle; repeat doses maintain benefits. Risks are low (e.g., rare infection, temporary floaters or redness), but steroids may raise eye pressure.

**Q38. Are eye injections painful or dangerous?**

**model: ChatGPT**

*answer:*

Eye injections, commonly used to treat conditions like diabetic retinopathy or age-related macular degeneration, are generally well-tolerated and relatively safe, but they do carry some discomfort and potential risks. Here's a detailed breakdown:

Pain and Discomfort

The procedure usually involves numbing the eye with topical anesthetic drops, so most patients report only a mild stinging or pressure rather than severe pain.

Some may feel temporary burning or a sensation of something in the eye.

Discomfort typically lasts only a few minutes, and the eye may feel slightly irritated for a day afterward.

Safety and Risks

Serious complications are uncommon, but possible risks include:

Infection inside the eye (endophthalmitis) - rare but serious.

Bleeding in or around the eye.

Retinal detachment - extremely rare.

Increased eye pressure, usually temporary.

Eye injections are performed under sterile conditions to minimize these risks, and most patients tolerate multiple injections safely over time.

Aftercare

Patients are usually advised to avoid rubbing the eye and to monitor for redness, pain, or vision changes.

Follow-up appointments help detect any early complications.

In summary, while there is some mild discomfort, eye injections are not usually very painful and are considered safe when performed by an experienced ophthalmologist. The benefits, such as preventing vision loss from diabetic retinopathy, generally far outweigh the risks.

**model: Copilot**

*answer:*

Eye injections are generally not painful and are considered very safe, though they do carry some risks. Most people feel pressure rather than sharp pain, thanks to numbing drops or gel applied beforehand. Serious complications are rare, but it's important to know what to expect and when to seek help.

What they feel like

Numbing drops are used so the injection itself is usually painless. Most people report only pressure or mild discomfort, not a sharp sensation.

Some irritation afterward is common due to the antiseptic used to clean the eye. This usually fades within hours.

A small red spot on the white of the eye (subconjunctival hemorrhage) can appear and looks scary but is harmless and clears in a week.

What risks exist (and how common they are)

Infection inside the eye (endophthalmitis): the most serious risk, but extremely rare-fewer than 1 in 2,000 injections when sterile technique is used.

Retinal detachment: very rare but serious.

Bleeding inside the eye: uncommon and usually mild.

Temporary eye pressure spikes: common but usually resolve quickly; more important to monitor if you have glaucoma.

Corneal abrasion: can cause significant pain an hour or more after the injection but is treatable.

What's normal afterward

Mild scratchiness, irritation, or floaters for a day or two.

Temporary blurry vision from ointment, air bubbles, or the medication.

When to call your eye doctor immediately

Severe pain

Increasing redness

Sudden vision loss

Worsening floaters or light sensitivity These can signal infection or other complications that need urgent treatment.

Bottom line

Eye injections are highly effective and widely used for conditions like macular degeneration and diabetic eye disease. They are not typically painful, and while risks exist, serious complications are very rare when performed by a trained ophthalmologist.

If you're feeling anxious about the procedure, that's completely normal - many patients say the anticipation is worse than the injection itself.

**model: DeepSeek**

*answer:*

This is an excellent and very common question. The short answer is: eye injections (intravitreal injections) can be uncomfortable, but they are designed to be minimally painful. They are generally very safe, but like any medical procedure, they carry small but serious risks.

Let's break down the pain and the danger separately.

Part 1: Are they painful?

Most people do not describe the injection itself as "painful," but rather as uncomfortable, pressure-like, or startling. Here's why, and what's done to minimize pain:

What you feel during the procedure:

Numbing drops: First, you'll receive several rounds of strong anesthetic eye drops. These numb the surface of the eye completely.

Antiseptic prep (the worst part for many): The doctor will clean the eye with a povidone-iodine (betadine) solution. This stings and burns for about 15-30 seconds. For many patients, this stinging is more uncomfortable than the actual injection.

The injection itself: You won't feel a sharp "prick" like a vaccine. The needle is microscopic (30-33 gauge, thinner than a human hair). Because the surface is numb, you mainly feel:

Pressure: The doctor has to steady the eye with a small instrument.

A brief "pop" or click: As the needle goes through the sclera (the white part of the eye).

A sense of fullness: As the medication is injected (this takes about 1 second).

Pain Scale: Most patients rate the injection pain as a 1 or 2 out of 10. The betadine burn is often rated higher.

After the injection:

You may feel a gritty, scratchy, or "foreign body" sensation for a few hours to a day. This is from the iodine and the tiny wound, not the medicine.

You might see floaters (a small bubble of medication or a tiny air bubble) - this is normal and goes away.

Serious pain after the procedure is not normal and you should call your doctor immediately.

How doctors minimize pain:

Strong topical anesthetics.

Sub-conjunctival anesthetic (a tiny extra numbing shot on the eye's surface - though this can cause a temporary red bump).

Very tiny needles.

Vibrating devices near the eye (distracts the brain from the sensation).

Part 2: Are they dangerous?

"Dangerous" needs context. For the diseases being treated (like Wet Macular Degeneration, Diabetic Retinopathy, or Retinal Vein Occlusion), not getting the injection is far more dangerous, as it leads to permanent, severe vision loss.

However, the injection itself carries a small risk of serious complications. The most feared risks are rare (less than 1 in 1,000 to 1 in 10,000 injections).

Serious but very rare risks:

Endophthalmitis (Infection): This is the most feared complication - a severe infection inside the eye. Risk: ~0.02% to 0.05% (1 in 2,000 to 1 in 5,000 injections). Symptoms are worsening pain, redness, light sensitivity, and vision loss hours to days after the injection. This is an emergency.

Retinal Detachment: The needle could theoretically cause the retina to pull away. Risk: ~0.01% to 0.05%. Symptoms are a sudden curtain over your vision, a flood of new floaters, or flashing lights.

Intraocular Hemorrhage (Bleeding): Bleeding can occur where the needle enters, causing a "subconjunctival hemorrhage" (a painless red splotch on the white of the eye - looks scary but is harmless). Rarely, bleeding can happen inside the eye (vitreous hemorrhage), which temporarily clouds vision but usually clears.

Common, non-dangerous side effects:

Subconjunctival hemorrhage: The red splotch. Very common (10-30%). Harmless, resolves in 1-2 weeks, looks worse than it is.

Eye floaters (from the medication or a small air bubble).

Elevated eye pressure (temporary, the doctor will check this).

Scratchy/gritty feeling for 24 hours.

How doctors make it safe:

Operating room level sterile technique (face mask, sterile gloves, drape, lid speculum to keep you from blinking).

Povidone-iodine (the best known antiseptic for the eye, used for a full 30-60 seconds before the injection).

No topical antibiotics after injection (recent studies show they don't prevent infection and can lead to bacterial resistance).

Summary Table: Pain vs. Danger

Aspect; What you experience; Risk Level

Pain during; Mostly pressure & a "pop." Betadine stings.; Low. Managed with anesthetic drops.

Pain after; Gritty, scratchy, mild ache for a few hours.; Low. Tylenol/ice packs can help.

Serious Danger; Infection, retinal detachment, severe bleeding.; Extremely Low (0.01% - 0.05% per injection).

Risk of Not Getting the Injection; Permanent, irreversible blindness from your retinal disease.; Very High (depending on the disease).

The Most Important Takeaway

The intense fear is almost always worse than the actual experience. The idea of a needle near the eye is terrifying. But hundreds of thousands of these injections are performed safely and effectively every single day. Patients typically say, "That was it? That wasn't nearly as bad as I thought."

When to call your doctor immediately (day or night) after an eye injection:

Worsening pain (not just gritty discomfort)

Worsening redness

Worsening light sensitivity

Decreasing vision (not just new floaters, but a dark curtain or fog)

**model: Doubao**

*answer:*

1. Pain level

Most intravitreal eye injections are not severely painful:

Doctors use topical anesthetic eye drops to numb the eye surface first.

You will only feel a brief mild pinch, pressure, or slight stinging for a few seconds during the injection.

No severe, lingering pain afterward; mild soreness or gritty feeling may last hours to 1 day.

2. Safety & Risks

Eye injections (common for diabetic retinopathy, macular degeneration, retinal vein occlusion) are routine, low-risk procedures performed millions of times yearly.

Minor, common temporary side effects

Mild eye redness, irritation, floaters

Temporary blurred vision

Minor subconjunctival bleeding (small red spot on eye surface, resolves in days)

Severe but rare serious risks

Endophthalmitis (eye infection, very rare)

Retinal detachment

Significant vision loss, increased eye pressure

These major complications are extremely uncommon when done by a trained ophthalmologist with sterile protocols.

3. Key takeaway

Pain: Minimal, just brief mild pressure/pinch with numbing drops.

Dangerous: Not dangerous for most patients; serious complications are rare.

The benefits (preserving/improving vision from retinal diseases) far outweigh the small procedural risks.

**model: Gemini**

*answer:*

The idea of a needle near the eye is enough to make anyone recoil, but in clinical practice, the procedure (intravitreal injection) is far more routine and controlled than it sounds.

Here is a breakdown of what to expect regarding discomfort and safety.

1. Does it hurt?

The short answer is: usually not. While "painless" is a strong word, most patients report a sensation of pressure rather than sharp pain.

Numbing Agents: Before the needle even comes close, the eye is thoroughly numbed using powerful anesthetic drops, a gel, or sometimes a small local injection.

The Sensation: Most people describe a "pinch" or a feeling of "fullness" that lasts only a few seconds.

Post-Procedure: Once the anesthetic wears off, the eye might feel "gritty" or scratchy (like there is a grain of sand in it) for a day or two.

2. Is it dangerous?

While all medical procedures carry some risk, eye injections have a very high safety profile. They are commonly used to treat conditions like macular degeneration or diabetic retinopathy, where the risk of not getting the injection (permanent vision loss) far outweighs the risks of the procedure.

Common (Minor) Side Effects:

Subconjunctival Hemorrhage: A bright red spot on the white of the eye caused by a tiny broken blood vessel. It looks alarming but is harmless and clears up like a bruise.

Floaters: You might see small specks or "bubbles" in your vision for a day as the medication settles.

Rare (Serious) Risks:

Infection (Endophthalmitis): This is the most serious risk, occurring in fewer than 1 in 1,000 cases. Doctors use antiseptic solutions (like povidone-iodine) to minimize this.

Retinal Detachment: Extremely rare, but doctors monitor for this during follow-ups.

Increased Eye Pressure: Often temporary and managed by the specialist.

When to seek immediate help

If you have recently had an injection and experience any of the following, contact your ophthalmologist immediately:

Significant pain or worsening discomfort.

A sudden "curtain" or "veil" over your vision.

Increased sensitivity to light.

Severe redness or discharge.

The Bottom Line: The anticipation is almost always worse than the injection itself. If you're feeling anxious, it's worth mentioning to your specialist-they can often provide additional numbing or talk you through the steps to keep you calm.

**model: Perplexity**

*answer:*

Eye injections, often called intravitreal injections, are typically not very painful during the procedure due to numbing drops used beforehand. Most patients feel only mild pressure, a brief hot/cold sensation, or nothing at all, lasting just seconds.

Pain After Injections

Post-injection discomfort can occur, like eye redness, soreness, or irritation from antiseptics like iodine, which may last days but is managed with prescribed drops. Severe pain hours later might signal a corneal abrasion, while pain after 24 hours could indicate rare infection-contact a doctor immediately if this happens.

Risks and Safety

These injections are generally safe with low complication rates, but rare dangers include infection (endophthalmitis, about 1 in 3,000), retinal detachment, bleeding, or temporary pressure spikes. Avoid swimming, contacts, or rubbing the eye for days after, and don't drive right away due to blurred vision

**Q39. What is laser treatment for diabetic retinopathy?**

**model: ChatGPT**

*answer:*

Laser treatment for diabetic retinopathy is a medical procedure used to prevent or slow vision loss caused by damage to the blood vessels in the retina due to diabetes. It involves using a focused beam of light (laser) to target specific areas of the retina.

There are two main types:

1. Focal or Grid Laser Treatment - Used mainly for diabetic macular edema (swelling in the central retina). The laser seals leaking blood vessels to reduce fluid accumulation and protect central vision.

2. Panretinal Photocoagulation (PRP) - Used for proliferative diabetic retinopathy (new, fragile blood vessels growing on the retina). The laser treats peripheral retina areas to reduce abnormal blood vessel growth, lowering the risk of severe bleeding and retinal detachment.

The procedure is usually done in an outpatient setting, with numbing eye drops. Patients may need multiple sessions. While laser treatment cannot restore lost vision, it is effective at preventing further deterioration and protecting remaining vision. Some temporary side effects can include blurred vision, dark spots, or mild discomfort.

**model: Copilot**

*answer:*

Laser treatment for diabetic retinopathy is a medical procedure that uses focused light to seal leaking blood vessels, shrink abnormal new vessels, and prevent further vision loss. It does not restore vision already lost, but it is highly effective at stopping progression.

What laser treatment does (the short answer)

Laser therapy-especially panretinal photocoagulation (PRP)-uses controlled burns on the retina to reduce abnormal blood vessel growth and leakage. This helps prevent severe complications like vitreous hemorrhage and retinal detachment.

Why diabetic retinopathy needs laser treatment

Diabetes can damage retinal blood vessels, causing them to:

Leak fluid or blood

Close off (poor blood flow)

Grow fragile, abnormal new vessels (proliferative retinopathy)

Laser treatment targets these problems by:

Shrinking abnormal vessels

Sealing leaks

Reducing oxygen demand in the retina, which slows disease progression

Types of laser treatment

Panretinal photocoagulation - Treats proliferative diabetic retinopathy

Hundreds of tiny laser spots are applied to the peripheral retina

Shrinks abnormal vessels and prevents bleeding

May require multiple sessions

Can reduce night vision or peripheral vision

Focal or grid laser - Treats diabetic macular edema

Targets specific leaking microaneurysms

Helps reduce swelling in the macula

Often used alongside anti-VEGF injections

What to expect during the procedure

Done in an eye clinic (no hospital stay)

Eye is numbed with drops

A special lens is placed on the eye

You may see bright flashes

Vision is blurry for the rest of the day

Someone must drive you home

Risks and side effects

Laser treatment is generally safe, but possible effects include:

Reduced peripheral vision

Reduced night vision

Mild discomfort during treatment

Temporary blurry vision

Despite these risks, it significantly reduces the chance of severe vision loss, which is why it remains a standard treatment.

When laser treatment is recommended

Laser is typically used when:

You have proliferative diabetic retinopathy

You have macular edema not fully controlled by injections

There is risk of bleeding or retinal detachment

Quick comparison: Laser vs. Injections

Treatment; Purpose; Pros; Cons

Laser therapy; Stops vessel growth & leakage; Long-lasting effect; May reduce peripheral/night vision

Anti-VEGF injections; Reduces swelling & vessel growth; Improves vision in many cases; Requires frequent injections

What are the possible side effects of laser treatment for diabetic retinopathy.

Laser treatment for diabetic retinopathy can cause temporary discomfort as well as long-term changes in vision, depending on the type of laser used. The most important side effects are loss of peripheral vision, reduced night and color vision, and temporary blurry vision, with rare but serious complications like retinal detachment or permanent central vision loss.

Key Side Effects (Most Common First)

Temporary blurry vision - Vision often becomes blurry for hours to days after treatment, especially if many laser spots were applied.

Eye discomfort or mild pain - A stinging or burning sensation during or shortly after the procedure is common.

Redness or irritation - Usually resolves within days.

Small blind spots near central vision - Seen mainly after macular laser for diabetic macular edema.

Reduced color vision - Can occur with both macular and panretinal photocoagulation (PRP).

Poor night vision - A well-known effect of PRP laser.

Reduced peripheral (side) vision - One of the most significant long-term effects of PRP; can affect driving eligibility.

Rare but Serious Complications

Retinal detachment - Uncommon but documented.

Macular edema worsening - Swelling of the central retina may increase temporarily.

Permanent central vision loss - Very rare; usually due to accidental laser burn to the fovea.

Accelerated cataract formation - Reported but uncommon.

Why These Side Effects Happen

Laser treatment works by creating tiny burns in the retina to shrink abnormal blood vessels or reduce swelling. These burns can affect retinal cells responsible for night vision, color perception, and peripheral vision, especially in PRP laser used for proliferative diabetic retinopathy.

Side Effects by Treatment Type

Treatment Type; Main Purpose; Most Likely Side Effects

Macular laser; Treat diabetic macular edema; Small blind spots, reduced color vision, rare central vision loss

Panretinal photocoagulation (PRP); Treat proliferative diabetic retinopathy; Reduced night vision, reduced peripheral vision, color vision loss, temporary blur

‍ What's Normal vs. Concerning?

Normal (first hours-days)

Blurry vision

Mild pain

Light sensitivity

Redness

Seek urgent care if you notice:

Sudden severe pain

Sudden major vision loss

New large floaters or flashes

A curtain-like shadow (possible retinal detachment)

**model: DeepSeek**

*answer:*

Laser treatment for diabetic retinopathy, commonly called focal/grid laser photocoagulation or panretinal photocoagulation (PRP), is a medical procedure that uses a focused beam of light to create tiny burns on the retina. Its goal is not to cure diabetes or restore already lost vision, but to slow or stop the progression of the disease and prevent severe vision loss.

There are two main types:

Focal/Grid Laser (for Diabetic Macular Edema - DME): When leaky blood vessels cause fluid to build up in the macula (the part of the retina for sharp, central vision), this laser seals the specific leaks. It helps reduce swelling and stabilizes vision.

Panretinal Photocoagulation (PRP) - for Proliferative Diabetic Retinopathy (PDR): In advanced disease, abnormal, fragile new blood vessels grow on the retina. These vessels can bleed and cause blindness. PRP involves applying hundreds to thousands of laser burns over most of the peripheral retina. This destroys oxygen-deprived tissue, which signals the abnormal vessels to shrink and stop growing.

What to expect during treatment:

Performed in an eye clinic (usually outpatient).

Your pupil will be dilated, and numbing drops applied.

A special contact lens is placed on your eye to aim the laser.

You may see bright flashes of light. Some patients feel mild discomfort or a stinging sensation.

Risks/side effects:

For focal treatment: Possible blind spots in the area treated, but often unnoticeable unless central vision is affected.

For PRP: Significant loss of peripheral (side) vision, reduced night vision, and possible decreased color vision.

Both can temporarily worsen swelling or cause mild blurriness.

Important notes:

Laser treatment does not reverse existing vision loss; it prevents further damage.

It is often done in multiple sessions, especially PRP.

It is not a substitute for good blood sugar, blood pressure, and cholesterol control, which are essential to prevent worsening retinopathy.

Always consult an ophthalmologist (retina specialist) to determine if laser is appropriate for your specific stage of diabetic retinopathy. Newer treatments like anti-VEGF eye injections are now often preferred for DME and even some PDR cases.

**model: Doubao**

*answer:*

Laser treatment for diabetic retinopathy is a minimally invasive outpatient procedure that uses focused medical laser light to treat damaged retinal blood vessels caused by long-term high blood sugar in diabetes. It aims to preserve remaining vision, slow disease progression, and prevent severe vision loss or blindness.

Core Mechanism

High blood sugar damages tiny retinal capillaries, causing leakage, swelling (macular edema), or abnormal new fragile blood vessel growth (proliferative diabetic retinopathy).

The laser creates small, controlled burns on the peripheral or affected retina:

Seals leaking blood vessels to stop fluid buildup.

Shrinks abnormal new blood vessels that risk bleeding or retinal detachment.

Reduces retinal oxygen demand, slowing abnormal vessel growth.

Main Types

1. Focal Laser Photocoagulation

Targets specific leaking microaneurysms in the macula.

Used for diabetic macular edema (DME) to reduce fluid accumulation and stabilize central vision.

2. Panretinal Laser Photocoagulation (PRP)

Laser applied to the peripheral entire retina (avoiding the central macula).

Reserved for proliferative diabetic retinopathy (PDR) with widespread abnormal new vessels, to prevent vitreous hemorrhage and retinal detachment.

Purpose & Benefits

Slow or halt retinopathy progression.

Reduce risk of permanent severe vision loss.

Control macular edema that blurs central vision.

Lower chances of retinal detachment and vitreous bleeding.

Procedure & Recovery

Done under local eye drops anesthesia, outpatient setting.

Takes 15-30 minutes; multiple sessions may be needed.

Mild temporary side effects: blurred vision, light sensitivity, mild eye discomfort.

Recovery is quick; most daily activities can resume soon after.

Limitations

It does not restore already lost vision-only protects remaining vision.

Cannot cure diabetes; requires lifelong blood sugar, blood pressure, and lipid control to prevent recurrence.

May cause minor peripheral vision or night vision reduction in some patients.

**model: Gemini**

*answer:*

Laser treatment for diabetic retinopathy, primarily known as laser photocoagulation, is a medical procedure used to stabilize vision by treating damaged or abnormal blood vessels in the retina. It is typically an outpatient procedure designed to prevent further vision loss rather than restoring vision already lost (Chhablani et al., 2021; "Laser management," n.d.).

There are two primary types of laser treatment based on the specific condition being addressed:

1. Panretinal Photocoagulation (PRP)

Used for Proliferative Diabetic Retinopathy (PDR), where abnormal new blood vessels grow on the surface of the retina.

Mechanism: The laser applies approximately 1,200 to 2,000 tiny burns to the peripheral (outer) retina (American Academy of Ophthalmology [AAO], 2013).

Goal: By "sacrificing" some of the peripheral retina, the laser reduces the retina's overall demand for oxygen. This causes the abnormal, leaky blood vessels to shrink and prevents new ones from forming (AAO, 2013; "Laser management," n.d.).

Side Effects: While highly effective at saving central vision, PRP can lead to a decrease in peripheral vision, night vision, and color perception (AAO, 2013; Chhablani et al., 2021).

2. Focal or Grid Laser Treatment

Used for Diabetic Macular Edema (DME), a condition where fluid leaks into the macula (the center of the retina responsible for sharp vision).

Mechanism:

Focal Laser: Targets specific leaking microaneurysms (small bulges in blood vessels) to seal them (Niu et al., 2023).

Grid Laser: Applies a pattern of burns across areas of diffuse swelling or thickening (Niu et al., 2023).

Goal: To reduce retinal thickening and stabilize visual acuity (Niu et al., 2023).

Modern Advances

While traditional laser treatment remains a "gold standard," newer technologies aim to reduce side effects like scarring:

Subthreshold/Micropulse Laser: Uses short bursts of energy that stimulate the retinal cells without causing permanent thermal damage or "burns" (Chhablani et al., 2021; Niu et al., 2023).

Navigated Laser: Uses computerized tracking to deliver laser pulses with extreme precision, reducing the risk of accidental damage to the fovea (the center of the macula) (Niu et al., 2023).

Pattern Scanning Laser (PASCAL): Allows the doctor to apply multiple laser spots rapidly in a single pattern, making the treatment faster and more comfortable for the patient (AAO, 2013; Chhablani et al., 2021).

**model: Perplexity**

*answer:*

Laser treatment for diabetic retinopathy uses targeted light energy to address abnormal blood vessels and swelling in the retina caused by diabetes. It helps slow or halt vision loss progression.

Types of Treatment

The main types are panretinal photocoagulation (PRP), which applies hundreds to thousands of laser burns to the peripheral retina to shrink abnormal vessels in proliferative diabetic retinopathy (PDR), and focal or grid laser, which seals leaking vessels to reduce macular edema (DME).

How It Works

Lasers destroy oxygen-starved retinal areas, reducing signals for new vessel growth, or seal microaneurysms to prevent fluid buildup. This preserves central vision and cuts severe vision loss risk by over 50% in trials like the Diabetic Retinopathy Study.

Procedure and Recovery

Done in-office after numbing drops, sessions last 15-60 minutes with minimal pain. Side effects may include temporary blurred vision or light sensitivity; full recovery takes days to a week.

Current Role

Often combined with anti-VEGF injections, laser remains key for PDR or when injections aren't suitable, offering durable, cost-effective results

**Q40. What are possible side effects of laser treatment?**

**model: ChatGPT**

*answer:*

Understood! Here's a clear summary of the possible side effects of laser treatment (photocoagulation) for diabetic retinopathy, based solely on general clinical knowledge:

Common (usually mild and temporary) side effects:

Blurred vision immediately after the procedure

Mild eye discomfort or irritation

Temporary worsening of macular edema (swelling in the central retina)

Small blind spots in peripheral vision

Less common or long-term side effects:

Permanent loss of peripheral (side) vision

Reduced night vision

Decreased color or contrast sensitivity

Retinal scarring, which can affect vision

Rare severe vision loss if complications occur

Notes:

Modern laser techniques, such as micropulse or patterned laser, aim to minimize damage and reduce these side effects.

Patients should report sudden vision changes, flashes of light, new floaters, or eye pain immediately, as these could indicate a complication.

Follow-up appointments are essential to monitor the retina and ensure the treatment has been effective.

**model: Copilot**

*answer:*

Laser treatment for diabetic retinopathy can cause temporary discomfort as well as long-term vision changes, depending on the type of laser used (macular laser vs. panretinal photocoagulation). The most important side effects are loss of peripheral vision, reduced night vision, and changes in color vision, according to major clinical sources.

Key Side Effects (Most Clinically Significant)

Peripheral vision loss - Panretinal photocoagulation (PRP) can reduce side vision; in some cases, this may affect driving eligibility.

Reduced night vision - PRP often decreases the retina's ability to function in low light.

Color vision changes - Both macular and PRP laser can reduce color discrimination.

Small blind spots - Macular laser may create tiny scotomas near the center of vision.

Temporary blurry vision - Very common after treatment; usually resolves within hours to days.

Short-Term, Usually Temporary Effects

Eye discomfort or mild pain - Burning or stinging sensation during or after treatment.

Redness or irritation - Often resolves within days.

Temporary vision changes - Blurriness or light sensitivity for a few days.

Rare but Serious Complications

Retinal detachment - Uncommon but possible.

Macular edema - Swelling of the central retina; may worsen vision.

Accidental central vision damage - Very rare; occurs if a laser burn hits the fovea.

Accelerated cataract formation - Reported but uncommon.

How to Think About These Risks

Laser treatment does not usually restore vision, but it is highly effective at preventing further vision loss, especially in proliferative diabetic retinopathy. Most patients tolerate the procedure well, and serious complications are rare.

The biggest trade-off is often saving central vision at the cost of some peripheral or night vision.

Summary Table

Side Effect; Severity; Typical Duration; Notes

Peripheral vision loss; Moderate-permanent; Long-term; Most associated with PRP

Night vision reduction; Moderate-permanent; Long-term; Common with PRP

Color vision changes; Mild-moderate; Long-term; Both PRP and macular laser

Small blind spots; Mild; Permanent; Macular laser

Blurry vision; Mild; Hours-days; Very common

Pain/irritation; Mild; Hours-days; Common

Retinal detachment; Rare; Variable; Requires urgent care

Visual Reference

**model: DeepSeek**

*answer:*

Laser treatments can cause side effects, though many are temporary and depend on the type of laser (e.g., ablative vs. non-ablative), the condition being treated, and your skin type. Here's a breakdown of possible side effects:

Common & Temporary Side Effects

Redness and Swelling - Often looks like a mild sunburn. Usually lasts a few hours to several days.

Discomfort or Pain - A stinging or warming sensation during and shortly after treatment.

Itching or Dryness - Common as the skin heals, especially after resurfacing lasers.

Peeling or Flaking - Particularly after ablative lasers (e.g., CO2, erbium) as old skin sheds.

Less Common but Possible Side Effects

Bruising - More likely with vascular lasers (e.g., for spider veins).

Crusting or Scabbing - Can occur with stronger treatments; usually heals in 5-10 days if left alone.

Temporary Darkening (Hyperpigmentation) - More common in darker skin tones (Fitzpatrick IV-VI) or with sun exposure post-treatment. Often fades over weeks to months.

Temporary Lightening (Hypopigmentation) - Less common, but can be permanent if melanocytes are damaged.

Rare but Serious Side Effects

Blisters or Burns - Usually from incorrect laser settings or inexperienced providers. Can lead to infection or scarring.

Infection - Bacterial, viral (e.g., herpes reactivation), or fungal. Antiviral medication may be prescribed before resurfacing.

Scarring - Rare with proper technique, but possible (hypertrophic or atrophic scars).

Eye Injury - If proper eye shields aren't used (e.g., retinal damage, iris changes).

Paradoxical Effects - Hair growth near treated areas (with laser hair removal) or worsening of the original condition (e.g., more redness after rosacea treatment).

Factors That Increase Risk

Darker skin tones - Higher risk of pigment changes.

Sun exposure before or after - Greatly increases risk of burns and hyperpigmentation.

Certain medications - Isotretinoin (Accutane) within 6-12 months, photosensitizing drugs, blood thinners.

History of herpes cold sores - Lasers around the mouth/nose can trigger outbreaks.

Inadequate cooling or wrong laser settings - Provider skill and equipment matter greatly.

What You Can Do to Minimize Side Effects

Choose an experienced, board-certified provider.

Avoid sun and tanning for 4-6 weeks before and after.

Follow pre-care (e.g., avoid retinoids, waxing) and post-care (gentle cleansing, heavy moisturizer, SPF 30+).

Do not pick at crusts or blisters.

When to call your doctor: Signs of infection (pus, increasing pain/redness, fever), severe blistering, or if pigment changes haven't improved in 2-3 months.

**model: Doubao**

*answer:*

Blurred or fluctuating visionMild blurriness, light sensitivity, or temporary vision fluctuation right after treatment, usually resolves in days to weeks.

Eye discomfort & irritationMild eye pain, stinging, dry eyes, or foreign body sensation; generally manageable with eye drops.

Photophobia & glareIncreased sensitivity to bright light, halos or glare around lights, especially at night.

Mild inflammationTemporary retinal inflammation or mild anterior uveitis.

Long-term / Permanent Side Effects

Peripheral vision lossLaser burns destroy abnormal retinal blood vessels and some normal peripheral retina, leading to peripheral visual field defects.

Decreased night vision & color vision impairmentDamage to photoreceptors affects dark adaptation and subtle color perception.

Reduced visual acuity (central vision)If laser is applied near the macula, it may cause slight permanent drop in central sharp vision.

Scar tissue formationRetinal scarring can develop, which may lead to further retinal traction over time.

Risk of macular edema worseningTemporary or persistent macular edema may occur post-laser.

Rare severe complications

Retinal hemorrhage

Retinal detachment

Vitreous hemorrhage

Secondary glaucoma (uncommon)

**model: Gemini**

*answer:*

The side effects of laser treatment vary depending on the type of laser used (such as ablative vs. non-ablative) and the area being treated. While most people experience mild, temporary reactions, there are some more significant risks to keep in mind.

Common, Temporary Side Effects

These typically occur immediately after the procedure and resolve within a few days to a week:

Redness and Swelling: This is the most frequent response, often described as feeling like a mild sunburn.

Itching and Tenderness: The treated area may feel sensitive or "tight" as it begins to heal.

Minor Discomfort: A stinging or snapping sensation is common during the procedure, which can leave the skin feeling warm afterward.

Peeling or Crusting: For resurfacing treatments, it is normal for the old skin to darken and peel away to reveal new skin.

Potential Risks and Complications

While rarer, some patients may experience more persistent issues:

Changes in Skin Pigmentation:

Hyperpigmentation: The skin becomes darker, which is more common in individuals with darker skin tones.

Hypopigmentation: The skin becomes lighter. This can sometimes be permanent, particularly with aggressive treatments.

Infection: Bacterial, fungal, or viral flare-ups can occur. A common example is the reactivation of the herpes simplex virus (cold sores) if the treatment is around the mouth.

Acne or Milia: Using heavy ointments or thick bandages during the recovery phase can sometimes cause small white bumps (milia) or temporary acne breakouts.

Scarring: While modern lasers have significantly reduced this risk, permanent scarring can occur if the skin does not heal properly or if the laser settings are too aggressive for the skin type.

Eye Injury: Because lasers are high-intensity light, there is a risk of retinal or corneal damage if proper eye protection is not worn during the session.

Comparison of Laser Types

Feature; Non-Ablative (Gentle); Ablative (Intense)

Recovery Time; Very short (hours to a few days); Longer (1 to 3 weeks)

Common Side Effects; Mild redness, slight swelling; Significant swelling, crusting, oozing

Risk of Pigment Change; Lower; Higher

Signs to Contact a Professional

You should reach out to your provider immediately if you experience:

Intense pain that doesn't improve with over-the-counter relievers.

Spreading redness or yellow discharge (signs of infection).

Blistering that was not discussed as a "normal" part of your specific treatment.

Fever or chills following the procedure.

**model: Perplexity**

*answer:*

Laser treatments, often used for skin resurfacing, hair removal, or other cosmetic purposes, carry common side effects that vary by type and intensity.

Common Side Effects

Mild redness, swelling, and itching typically occur right after treatment and resolve within hours to days. Dryness, peeling, or a burning sensation may follow for up to a week. Temporary darkening or lightening of the skin (hyperpigmentation or hypopigmentation) affects around 33% of patients, especially those with darker tones.

Less Common Effects

Prolonged redness can last 3-6 months after aggressive CO2 lasers. Milia (small white bumps) or acne flare-ups happen in about 17% of cases due to post-treatment sensitivity. Rarely, burns, blisters, or infections (bacterial, viral like herpes) arise if aftercare is poor.

Serious Risks

Scarring, persistent color changes, or eye damage (if unprotected) are possible with improper use. Ocular treatments may cause pain, vision loss, or retinal issues in specific cases like retinopathy therapy. Always follow provider instructions to minimize risks

**Q41. When might diabetic retinopathy need eye surgery?**

**model: ChatGPT**

*answer:*

Diabetic retinopathy may require eye surgery when the disease has progressed to severe stages that cannot be managed with medication or laser treatment alone. Specifically, surgery is usually considered in the following situations:

1. Proliferative Diabetic Retinopathy (PDR) with Vitreous Hemorrhage

1. When new, fragile blood vessels bleed into the vitreous (the gel inside the eye), causing sudden or persistent vision loss that does not clear on its own.

2. Surgery in this case is typically a vitrectomy, which removes the blood-filled vitreous to restore vision.

2. Tractional Retinal Detachment

1. Scar tissue from abnormal blood vessels can pull the retina away from the back of the eye.

2. Surgery (often vitrectomy with membrane peeling and sometimes silicone oil or gas tamponade) is needed to reattach the retina.

3. Severe Macular Edema Not Responsive to Other Treatments

1. If swelling in the central retina (macula) persists despite anti-VEGF injections or laser therapy, surgery may be considered to relieve traction or remove vitreous contributing to edema.

4. Combined Conditions

1. Some patients may have both vitreous hemorrhage and retinal detachment, which increases the urgency for surgical intervention.

In general, the decision for surgery is made by a retinal specialist based on the severity of bleeding, the presence of retinal detachment, and the patient's visual needs. Early detection and treatment with laser or injections can often prevent the need for surgery.

**model: Copilot**

*answer:*

Diabetic retinopathy typically needs eye surgery only when the disease has progressed to advanced or vision-threatening stages, especially when bleeding, scarring, or retinal detachment prevents other treatments (like injections or laser) from working.

Below is a clear breakdown of when surgery becomes necessary, why, and what types of surgery are used.

When diabetic retinopathy requires surgery

Surgery is usually considered in proliferative diabetic retinopathy (PDR) or severe complications. The most common triggers are:

1. Non-clearing vitreous hemorrhage

Blood leaks into the gel of the eye (the vitreous), blocking vision. Surgery (a vitrectomy) is needed when:

The bleeding does not clear after ~4 weeks

Bleeding keeps recurring

The doctor cannot see the retina well enough to treat it with laser

This is the #1 reason for diabetic eye surgery.

2. Tractional retinal detachment (TRD)

Scar tissue from abnormal blood vessels pulls the retina off the back of the eye. Surgery is urgent when:

The macula is threatened or already detached

The detachment is progressing

There is a combined tractional + rhegmatogenous detachment (a tear plus pulling)

This is one of the most vision-threatening complications and requires early vitrectomy.

3. Severe proliferative diabetic retinopathy not responding to injections or laser

If abnormal blood vessels continue to grow or bleed despite:

Anti-VEGF injections

Panretinal photocoagulation (laser)

...then surgery may be needed to remove scar tissue and stabilize the retina.

4. Dense scar tissue / epiretinal membranes

Scar tissue can distort the retina or cause macular traction. Surgery is needed when:

Vision is significantly distorted

The macula is being pulled or wrinkled

5. Severe diabetic macular edema with traction

Most macular edema is treated with injections, but if mechanical pulling is the cause, vitrectomy may be required.

What surgeries are used?

Vitrectomy

The most common surgery. It removes:

Blood in the vitreous

Scar tissue

Traction on the retina

It also allows the surgeon to apply laser inside the eye.

Panretinal photocoagulation (PRP)

Technically a "laser surgery," used to shrink abnormal blood vessels in PDR.

Visual guide

How to know if you might be approaching the point of needing surgery

You may be nearing surgical territory if you experience:

Sudden, persistent vision loss from bleeding

A dark curtain or shadow (possible detachment)

Distorted or warped central vision

Repeated episodes of floaters or haze

Your retina specialist says they cannot see the retina due to blood

**model: DeepSeek**

*answer:*

Diabetic retinopathy may need eye surgery in the following advanced stages or complications:

Proliferative diabetic retinopathy (PDR) with persistent vitreous hemorrhage that does not clear on its own after several months, or when it prevents examination/treatment of the retina.

Tractional retinal detachment involving or threatening the macula (central vision), causing significant vision loss or distortion.

Combined tractional and rhegmatogenous retinal detachment (tear plus pulling).

Severe, non-clearing vitreous hemorrhage with neovascularization that fails to respond adequately to anti-VEGF injections or panretinal photocoagulation (laser).

Pre-macular subhyaloid hemorrhage (a large "dense" hemorrhage directly over the macula) that does not resolve spontaneously.

Ghost cell glaucoma or hemolytic glaucoma (secondary glaucoma from blood breakdown products) unresponsive to medical therapy.

The most common surgeries are vitrectomy (to remove blood/scar tissue) and sometimes scleral buckling or endolaser. Surgery is typically considered when vision is significantly threatened or when non-surgical treatments (laser, anti-VEGF injections, glycemic control) are insufficient.

**model: Doubao**

*answer:*

Surgery is usually required for advanced or complicated diabetic retinopathy when medication/injections/laser can't control the condition or save vision.

1. Proliferative Diabetic Retinopathy (PDR)

Severe neovascularization (abnormal new blood vessels) on the retina/optic disc

Vitreous hemorrhage that doesn't clear on its own after weeks/months

Tractional retinal detachment caused by scar tissue pulling on the retina

2. Diabetic Macular Edema (DME)

Severe macular edema unresponsive to anti-VEGF injections or laser therapy

Persistent fluid buildup causing progressive central vision loss

3. Other surgical indications

Vitreomacular traction affecting central vision

Epiretinal membrane formation distorting vision

Severe cataract related to long-term diabetes that impairs retinal examination and vision

Common surgeries

Pars plana vitrectomy: Remove hemorrhaged vitreous, peel scar tissue, repair retinal detachment

Macular surgery: Membrane peeling for epiretinal membrane/traction

Cataract surgery: Remove diabetic cataract for better vision and follow-up retinal treatment

**model: Gemini**

*answer:*

Eye surgery for diabetic retinopathy is typically reserved for more advanced stages of the disease, particularly when non-invasive treatments like injections or laser therapy haven't been sufficient or aren't possible.

The most common surgical procedure is a vitrectomy. Here are the specific scenarios where it is usually recommended:

1. Severe Bleeding (Vitreous Hemorrhage)

In the advanced "proliferative" stage, the eye grows new, fragile blood vessels. These can leak blood into the vitreous (the clear, jelly-like substance that fills the center of your eye). If this blood doesn't clear up on its own within a few months, surgery is needed to remove the blood-filled gel and replace it with a clear solution so light can reach the retina again.

2. Retinal Detachment

The abnormal blood vessels mentioned above often lead to the formation of scar tissue. This scar tissue can pull the retina away from the back of the eye. A retinal detachment is a medical emergency that requires surgery to prevent permanent vision loss.

3. Extensive Scar Tissue

Even if the retina hasn't detached yet, a surgeon might perform a vitrectomy to remove significant scar tissue that is tugging on the retina or causing severe distortion of your vision.

4. Persistent Macular Edema

The macula is the part of the retina responsible for sharp, central vision. If it remains swollen (edema) despite several rounds of injections or laser treatments, surgery may be considered to relieve pressure or tension on the macula.

Comparison of Advanced Treatments

Treatment; When it's used; How it works

Laser Surgery; To shrink abnormal vessels or seal leaks.; Focused light creates small "burns" to seal vessels.

Eye Injections; To reduce swelling or stop vessel growth.; Anti-VEGF drugs or steroids are injected into the eye.

Vitrectomy; For bleeding, scar tissue, or detachment.; The vitreous gel is removed and replaced.

Note: Early-stage diabetic retinopathy often requires no treatment other than careful monitoring and managing blood sugar, blood pressure, and cholesterol. If you notice sudden vision changes, such as a "curtain" falling over your eyes or a sudden increase in floaters, you should seek medical attention immediately

**model: Perplexity**

*answer:*

Diabetic retinopathy typically needs eye surgery only when the disease becomes advanced or causes serious complications that non‐surgical treatments (like injections or laser) cannot manage well. The most common surgery is a vitrectomy, done in the operating room to remove blood or scar tissue from inside the eye.

When surgery is usually considered

Vitreous hemorrhage (bleeding in the eye): When abnormal new blood vessels bleed into the gel (vitreous) of the eye and do not clear on their own, causing significant vision loss, a vitrectomy may be needed to remove the blood.

Tractional retinal detachment: If scar tissue pulls on the retina and causes it to detach, surgery is often required to remove the scar tissue and re‐attach the retina.

Advanced proliferative diabetic retinopathy: When the disease has reached the most severe stage (many abnormal vessels and scarring), and laser or injections have not controlled the problem, surgery may be offered to prevent further vision loss or help restore some vision.

Severe diabetic macular edema that does not respond: In some refractory cases of diabetic macular swelling, vitrectomy may be considered if other treatments fail.

Who decides if surgery is needed?

An eye specialist (retina specialist) evaluates your retinal photos, OCT scans, and exam findings and coordinates with your diabetes doctor; surgery is usually discussed only after the risks and benefits are weighed for your specific stage and vision. If you have diabetic retinopathy, regular dilated eye exams are the best way to catch problems early and avoid reaching the point where surgery is necessary

**Q42. Can treatment improve vision, or does it only stop worsening?**

**model: ChatGPT**

*answer:*

Yes, treatment for diabetic retinopathy can do more than just prevent further damage-it can sometimes improve vision, depending on the stage and type of the disease.

Early-stage disease: Controlling blood sugar, blood pressure, and cholesterol can help slow progression and, in some cases, reverse minor retinal changes, leading to slight improvements in vision.

Macular edema: Swelling in the central retina can be treated with medications injected into the eye (such as anti-VEGF therapy) or laser therapy. These treatments often reduce swelling and improve visual clarity, sometimes restoring lost vision.

Advanced proliferative disease: Treatments like laser photocoagulation or vitrectomy surgery primarily prevent severe vision loss, but in certain cases-especially if bleeding or retinal detachment is treated promptly-they can restore some vision.

The key factor is timing: the earlier treatment is started, the higher the chance of improving vision. Late-stage disease may be stabilized, but significant recovery is less likely.

**model: Copilot**

*answer:*

Short answer: Some eye treatments can genuinely improve vision, while others mainly stop or slow further worsening. It depends entirely on the specific eye condition.

Below is a clear breakdown based on what current medical sources show, with citations.

When treatment improves vision

These conditions have treatments that typically restore or significantly improve sight:

Cataracts - Cataract surgery removes the cloudy lens and replaces it with a clear artificial one. This directly improves vision, often dramatically. UCLA Health notes that cataract surgery replaces the cloudy lens with an artificial one to restore clarity.

Refractive errors (nearsightedness, farsightedness, astigmatism) - Glasses, contacts, or laser surgery (LASIK/PRK) improve vision by correcting how light focuses on the retina.

Dry eye disease - Treating the underlying cause (e.g., inflammation, meibomian gland dysfunction) can improve comfort and sometimes improve visual clarity, since dry eye can blur vision. USC's Dry Eye Center emphasizes that proper treatment can improve daily functioning and visual quality.

When treatment mainly prevents worsening

These conditions usually cannot be reversed, but treatment is crucial to slow or stop progression:

Glaucoma - Treatments (eye drops, laser, surgery) lower eye pressure to prevent optic nerve damage. They do not restore lost vision.

Macular degeneration -

Dry AMD: No cure; lifestyle and supplements slow progression.

Wet AMD: Injections can stabilize and sometimes improve vision, but not always fully restore it.

Diabetic retinopathy - Laser, injections, and blood sugar control prevent further damage. Some improvement is possible, but the main goal is preservation.

Why the difference?

It comes down to what part of the eye is damaged:

If the problem is with optics (lens, cornea, tear film), treatment often improves vision.

If the problem is with nerve tissue (retina, optic nerve), treatment usually prevents further loss but cannot fully restore what's gone.

Quick comparison

Condition; Can treatment improve vision?; Main goal

Cataracts; Yes, often dramatically; Restore clarity

Refractive errors; Yes; Correct focus

Dry eye; Sometimes; Improve comfort & clarity

Glaucoma; No; Prevent nerve damage

Macular degeneration; Sometimes (wet AMD); Slow progression

Diabetic retinopathy; Limited; Prevent worsening

**model: DeepSeek**

*answer:*

That's an excellent question, and the answer depends entirely on the specific underlying cause of the vision problem. There is no single answer.

Here's the breakdown, ranging from conditions where treatment can dramatically reverse vision loss to those where the goal is solely to prevent further decline.

1. Treatments That Can Improve Vision (Reverse Loss)

In many common eye conditions, treatment directly addresses the structural problem, allowing vision to get better.

Refractive Errors (Nearsightedness, Farsightedness, Astigmatism, Presbyopia): This is the clearest example. Glasses, contact lenses, and laser surgery (LASIK, PRK) immediately improve vision to 20/20 or better. They don't just stop worsening; they fix the problem.

Cataracts: A cataract is a cloudy lens. Cataract surgery removes the cloudy lens and replaces it with a clear artificial one. This dramatically improves vision, often restoring clarity and color brightness that was lost over years.

Diabetic Retinopathy / Diabetic Macular Edema (DME): High blood sugar damages retinal blood vessels, causing swelling (edema) and vision loss. Anti-VEGF injections (like Eylea, Lucentis, Avastin) into the eye can reduce swelling, dry up fluid, and significantly improve vision, especially when started early.

Wet Age-Related Macular Degeneration (AMD): Abnormal blood vessels grow under the macula and leak. Anti-VEGF injections can shrink these vessels and stop the leak, which often leads to improved vision in about 1/3 of patients and prevents severe loss in most others.

Retinal Vein Occlusion (RVO): A blockage in the retinal veins causes bleeding and swelling. Anti-VEGF injections or laser can reduce the swelling, leading to meaningful vision improvement.

Inflammatory Conditions (Uveitis, Iritis): Inflammation inside the eye can blur vision. Treating the inflammation with corticosteroid eye drops, pills, or injections can completely restore vision if caught before permanent scarring occurs.

Sudden Vision Loss (e.g., from a swollen optic nerve or retinal artery blockage): In some emergencies (like optic neuritis treated with IV steroids), rapid treatment can restore a significant amount of lost vision.

2. Treatments That Primarily Stop Worsening (Prevent Further Loss)

For these conditions, damage is often permanent by the time it's noticed. The goal is to protect the remaining vision.

Glaucoma: This disease slowly damages the optic nerve, which cannot regenerate. Treatment (eye drops, laser, surgery) lowers eye pressure to stop or dramatically slow further nerve damage. Any vision already lost (peripheral vision blind spots) is permanent. Treatment rarely recovers lost vision.

Dry Age-Related Macular Degeneration (Dry AMD): There is no cure for the dry form. AREDS2 vitamin supplements cannot reverse existing damage, but studies show they can slow the progression to advanced (wet) AMD and help preserve central vision longer.

Retinitis Pigmentosa (RP) & other inherited retinal degenerations: Most treatments aim to slow the inevitable decline in peripheral and night vision. Vitamin A (controversially) and new gene therapies (like for RPE65 mutation) can preserve function, but reversing significant rod/cone death remains extremely difficult.

High Myopia (Pathologic Myopia): As the eyeball stretches abnormally, it can damage the retina and macula. Treatments like eye drops (low-dose atropine) or surgical implants (ICL) in children can slow the eyeball's growth, preventing severe future damage. Existing retinal scars don't improve.

Corneal Scarring (from infection or injury): A corneal transplant can replace the scarred tissue. Once healed, vision can improve dramatically. So, while the scar itself is permanent, the treatment (transplant) is a restorative act. However, if a scar is inoperable, treatments only manage dryness or inflammation to stop it from getting worse.

Key Takeaway: The "Saving vs. Reversing" Spectrum

Think of it on a spectrum:

Area of Eye; Common Condition; Action of Treatment; Vision Outcome

Lens; Cataract; Replace lens; Improves

Cornea; Bad refractive error; Reshape (LASIK); Improves

Retina (macula); Wet AMD / DME; Dry fluid, shrink vessels; Often Improves

Optic Nerve; Glaucoma; Lower pressure; Only stops worsening

Retina (rods/cones); Retinitis Pigmentosa; Gene therapy / supplements; Only slows worsening

The Most Important Principle: "Time is Tissue"

For almost all conditions except refractive error and cataracts, delaying treatment means losing vision that will never come back.

In diabetes, wet AMD, or vein occlusions: Early treatment can improve vision. Late treatment may only stop worsening.

In glaucoma: Damage is permanent. Today's treatment saves tomorrow's vision, not yesterday's.

Final Answer: Yes, treatment can absolutely improve vision for many common conditions (cataracts, diabetic eye disease, wet AMD, refractive errors). However, for other serious diseases, especially those involving the optic nerve (glaucoma) or advanced retinal degeneration, the primary goal is to stop further permanent vision loss.

**model: Doubao**

*answer:*

In short: Treatment can both stop worsening AND sometimes improve vision-it depends on the eye condition.

1. Conditions that only halt progression (rarely restore lost vision)

Diabetic retinopathy (advanced), glaucoma, macular degeneration (late stage)Nerve/retinal cells that are already dead cannot regrow. Treatment mainly protects remaining vision and prevents further loss.

2. Conditions that can clearly improve vision with treatment

Cataract: Surgery almost always restores clear vision.

Dry eye, corneal inflammation, uveitis: Medication can reverse blurred vision.

Refractive issues (myopia/astigmatism): Glasses, laser surgery correct vision fully.

Early diabetic retinopathy / macular edema: Laser, anti-VEGF injections can reduce swelling and recover partial or significant vision.

Key takeaway

Early intervention: Much higher chance to improve vision.

Late-stage disease: Main goal is only stopping further decline.

**model: Gemini**

*answer:*

The answer depends entirely on the specific condition being treated. In the world of ophthalmology, some treatments are designed to "rescue" vision, while others are strictly "preventative."

Here is how it generally breaks down across common eye conditions:

1. Conditions Where Vision Can Improve

In many cases, medical intervention can actually restore clarity or sharpen sight that was previously lost.

Cataracts: This is the most common example of vision restoration. Surgery replaces a cloudy natural lens with a clear artificial one, often resulting in vision that is better than it was years prior to the surgery.

Refractive Errors: Near-sightedness, far-sightedness, and astigmatism can be "corrected" with glasses, contacts, or LASIK. While the eye's shape doesn't change permanently (in the case of lenses), the functional vision improves immediately.

Wet Macular Degeneration: Unlike the "dry" version, "wet" AMD involves leaking blood vessels. Injections (anti-VEGF therapy) can reduce fluid and, in many patients, actually improve visual acuity rather than just stabilizing it.

Corneal Issues: A corneal transplant or specialized contact lenses for conditions like keratoconus can significantly improve sight.

2. Conditions Focused on Stabilization

For these conditions, the goal is "damage control." Once the nerve tissue is gone, it generally cannot be recovered with current technology.

Glaucoma: This is the classic "stop the worsening" condition. Because glaucoma damages the optic nerve, any vision lost is typically permanent. Treatment (drops or surgery) aims to lower eye pressure to save the remaining sight.

Dry Macular Degeneration: Currently, there is no treatment to "reverse" dry AMD. Supplements (like the AREDS2 formula) and new geographic atrophy treatments are designed to slow the progression of the disease.

Diabetic Retinopathy: While laser treatments and injections can sometimes improve vision by reducing swelling (edema), they are often used primarily to prevent further hemorrhaging and permanent scarring.

3. The "Gray Area": Amblyopia (Lazy Eye)

In children, vision can often be "trained" to improve through patching or drops. Historically, it was believed this was impossible after age 7 or 8, but newer research suggests that some level of improvement is possible in older children and even some adults through specialized vision therapy.

Summary Table

Goal: Improvement; Goal: Stabilization

Cataracts; Glaucoma

Refractive Errors (LASIK/Glasses); Dry Macular Degeneration

Wet Macular Degeneration; Advanced Diabetic Retinopathy

Retinal Detachment (if caught early); Optic Nerve Atrophy

The bottom line: If you are noticing a change in your vision, the "reversibility" often depends on whether the issue is mechanical (like a cloudy lens) or neurological (like nerve damage). Mechanical issues are much easier to "fix," while neurological damage is currently managed by stopping further decline.

**model: Perplexity**

*answer:*

Treatment can both improve vision and stop worsening.

It depends on the underlying cause of vision impairment. Many conditions allow for actual vision gains through targeted therapies, while others focus more on halting progression.

Conditions with Vision Improvement

Anti-VEGF injections for retinal vein occlusion (RVO) often yield long-lasting gains, with patients retaining an average of three lines better on eye charts after five years compared to baseline, though ongoing treatment is usually needed.

Refractive surgeries like LASIK correct nearsightedness, farsightedness, or astigmatism by reshaping the cornea, directly enhancing eyesight.

Vision therapy effectively treats convergence insufficiency, improving eye coordination, but lacks support for other issues.

Cases Limited to Slowing Progression

For glaucoma, advanced macular degeneration, or diabetic retinopathy, treatments mainly preserve remaining vision by slowing damage, as full reversal isn't possible once nerve cells are lost.

Early intervention is key-medications, drops, or surgery can manage diabetes-related vision loss and sometimes partially reverse it.

Key Factors for Success

Consult an eye doctor for diagnosis, as options like glasses, meds, laser, or lifestyle changes (e.g., better diabetes control) vary by condition.

Early detection often enables the best outcomes, whether improvement or stabilization

**Q43. What happens if diabetic retinopathy is not treated?**

**model: ChatGPT**

*answer:*

If diabetic retinopathy is not treated, it can progressively worsen and lead to serious eye complications. Here's what can happen over time:

1. Vision deterioration - Early stages may be asymptomatic, but over months to years, untreated retinopathy can cause blurry or distorted vision.

2. Macular edema - Fluid can accumulate in the central part of the retina (the macula), leading to significant central vision loss.

3. Proliferative diabetic retinopathy - Abnormal blood vessels may grow on the retina. These vessels are fragile and prone to bleeding (vitreous hemorrhage), which can cause sudden vision loss.

4. Retinal detachment - Scar tissue from abnormal vessels can pull the retina away from the back of the eye, potentially causing permanent blindness if not surgically treated.

5. Neovascular glaucoma - Abnormal vessels can grow in the front of the eye, increasing eye pressure and damaging the optic nerve.

6. Blindness - Ultimately, if left untreated, severe diabetic retinopathy can lead to irreversible vision loss.

The progression varies among individuals, depending on blood sugar control, blood pressure, and overall health. Early detection and timely intervention (like laser therapy, anti-VEGF injections, or surgery) are crucial to prevent permanent vision loss.

**model: Copilot**

*answer:*

Untreated diabetic retinopathy almost always progresses, and the consequences become increasingly severe and irreversible. The most important point: lack of treatment can lead to permanent blindness.

What happens as diabetic retinopathy goes untreated

Progressive retinal blood-vessel damage - High blood sugar weakens retinal vessels, causing them to leak fluid or blood. Over time, more vessels become damaged.

Macular edema - Fluid builds up in the macula (the sharp-vision center), causing blurry vision and distortion. This is the most common cause of vision loss in diabetes.

Retinal ischemia - Blocked vessels starve the retina of oxygen, permanently damaging vision.

Proliferative retinopathy - The retina grows fragile new blood vessels that bleed easily and form scar tissue. This is the dangerous advanced stage.

Vitreous hemorrhage - Bleeding into the eye's gel can cause sudden vision loss or a curtain-like shadow. Severe bleeding can block all vision.

Retinal detachment - Scar tissue pulls the retina off the back of the eye, causing flashes, floaters, or severe vision loss. This is an emergency.

Neovascular glaucoma - Abnormal vessels block fluid drainage, raising eye pressure and damaging the optic nerve.

Complete blindness - A combination of macular edema, retinal detachment, hemorrhage, and glaucoma can lead to total, irreversible vision loss.

Why untreated retinopathy is so dangerous

It often has no symptoms early on, so damage accumulates silently.

Once vision is lost, much of it cannot be restored, even with surgery.

Progression accelerates when blood sugar, blood pressure, or cholesterol are uncontrolled.

What prevents these outcomes

Regular dilated eye exams - yearly or more often if retinopathy is present.

Tight blood sugar control - reduces risk of progression.

Blood pressure and cholesterol management - protects retinal vessels.

Timely treatment - anti-VEGF injections, laser therapy, or vitrectomy can stabilize or improve vision if done early.

**model: DeepSeek**

*answer:*

If diabetic retinopathy is not treated, the condition typically worsens over time, potentially leading to severe and irreversible vision problems. Here is what can happen, step by step:

Progression to advanced stages

Proliferative diabetic retinopathy (PDR) may develop. In PDR, fragile new blood vessels grow on the retina and into the vitreous gel. These vessels bleed easily, causing recurrent vitreous hemorrhages, which can suddenly blur vision.

Vision loss due to complications

Vitreous hemorrhage: Blood in the vitreous blocks light from reaching the retina. Small bleeds may clear on their own, but large or recurrent bleeds can cause persistent vision loss.

Tractional retinal detachment: Scar tissue from healing hemorrhages pulls the retina away from its normal position, causing marked, often permanent vision loss.

Neovascular glaucoma: New vessels grow on the iris, blocking normal fluid drainage and raising eye pressure. This painful condition is difficult to treat and commonly leads to blindness.

Macular involvement

Diabetic macular edema (DME) can worsen. Fluid buildup at the macula (central retina) blurs central vision needed for reading, driving, and recognizing faces. Chronic DME without treatment often results in permanent central vision loss.

Permanent blindness

Once the macula is damaged or retinal detachment occurs, vision loss is typically irreversible, even with later surgery. Diabetic retinopathy is a leading cause of blindness in working-age adults worldwide.

Key preventive note:

Early treatment (laser, anti-VEGF injections, vitrectomy) and strict blood sugar, blood pressure, and cholesterol control can slow or halt progression. Without treatment, visual decline is common, but not always immediate-some people remain asymptomatic until late stages. Regular eye exams are essential even when vision seems fine.

**model: Doubao**

*answer:*

If diabetic retinopathy is left untreated, the condition progresses gradually and causes permanent, irreversible eye damage and vision loss:

Progressive Vision BlurrinessBlood vessels in the retina keep leaking fluid, lipids, and blood, leading to persistent blurred or fluctuating vision, difficulty reading, and trouble seeing fine details.

Macular EdemaFluid accumulates in the macula (the central vision area), causing severe central vision loss-this is the leading cause of visual impairment in untreated diabetes.

Retinal Ischemia & NeovascularizationDamaged retinal circulation creates oxygen deficiency. The eye grows fragile abnormal new blood vessels on the retina and optic nerve. These vessels are weak and easily bleed.

Vitreous HemorrhageBleeding from abnormal vessels spills into the vitreous gel inside the eye, causing sudden floaters, dark shadows, or sudden complete blockage of vision.

Retinal DetachmentScar tissue formed from bleeding pulls the retina away from its normal position. Without urgent surgery, this leads to permanent sight loss in the affected eye.

Secondary GlaucomaAbnormal blood vessels block fluid drainage in the eye, causing severely high eye pressure, painful neovascular glaucoma, and eventual irreversible optic nerve damage.

Permanent BlindnessAdvanced untreated diabetic retinopathy is one of the most common preventable causes of legal blindness in working-age adults globally.

Dual-eye InvolvementDiabetic retinopathy usually affects both eyes; if one eye is neglected, the other will typically follow the same progressive path.

**model: Gemini**

*answer:*

Untreated diabetic retinopathy is a progressive condition that, over time, can lead to irreversible vision loss and permanent blindness. Because it often shows no symptoms in the early stages, the damage can become significant before a person realizes there is a problem.

Here is a breakdown of what typically happens if the condition is left to progress without medical intervention:

Progression of Eye Damage

As blood sugar levels remain high, the tiny blood vessels in the retina (the light-sensitive tissue at the back of the eye) become damaged. This usually follows a specific path:

Leakage and Swelling: Small bulges called microaneurysms develop in the blood vessel walls. These can leak fluid and blood into the retina, causing it to swell and distorting your vision.

Vessel Blockage: Over time, these blood vessels may close off entirely, depriving parts of the retina of blood and oxygen.

Abnormal Vessel Growth (Proliferative Retinopathy): In an attempt to restore blood flow, the eye grows new, abnormal blood vessels. However, these new vessels are extremely fragile and prone to leaking or breaking.

Serious Complications

If the "proliferative" stage is reached without treatment, it can trigger several life-altering complications:

Vitreous Hemorrhage: The new, fragile blood vessels may bleed into the clear, jelly-like center of the eye (the vitreous). A small amount of blood causes "floaters," but a large amount can completely block all vision.

Tractional Retinal Detachment: The abnormal blood vessels are often accompanied by the growth of scar tissue. This scar tissue can pull the retina away from the back of the eye. This is a medical emergency that causes permanent vision loss if not repaired immediately.

Neovascular Glaucoma: New blood vessels can grow on the iris (the colored part of the eye) and interfere with the normal flow of fluid out of the eye. This causes pressure to build up, damaging the optic nerve and leading to blindness.

Diabetic Macular Edema (DME): This occurs when fluid leaks into the macula, the part of the retina responsible for sharp, central vision. Without treatment, DME can make it impossible to read, drive, or recognize faces.

Key Prevention

The most critical thing to know is that while advanced damage is often irreversible, early treatment can stop or slow the progression. Regular dilated eye exams are essential for people with diabetes, as a specialist can often detect these changes before they impact your sight.

Warning: If you experience a sudden increase in floaters, flashes of light, or a "curtain" falling over your field of vision, seek emergency eye care immediately, as these are signs of retinal detachment.

**model: Perplexity**

*answer:*

If diabetic retinopathy goes untreated, it can progress from mild damage to severe, irreversible vision loss or blindness. Early stages may show no symptoms, but advancement leads to serious eye complications.

Key Complications

Weakened retinal blood vessels leak fluid and blood, causing macular edema that swells the central retina and blurs vision. New, fragile vessels (neovascularization) can form, leading to vitreous hemorrhage (bleeding into the eye's gel) or scar tissue that pulls the retina away in retinal detachment. High eye pressure from blocked drainage may trigger glaucoma, damaging the optic nerve permanently.

Progression Risks

Symptoms worsen over time, including floaters, dark spots, faded colors, poor night vision, and blind areas. In advanced proliferative stages, untreated cases often result in profound vision impairment, as dead retinal tissue cannot recover. Without intervention like laser therapy or injections, the risk of total blindness rises significantl

**Q44. Are there home remedies that can replace medical treatment?**

**model: ChatGPT**

*answer:*

For most medical conditions, including chronic or serious diseases, home remedies cannot fully replace professional medical treatment. They may help support overall health, alleviate minor symptoms, or complement treatment, but relying solely on them can be dangerous. Here's a breakdown:

When Home Remedies Can Help

Symptom relief: Warm compresses for minor eye irritation, hydration for mild colds, honey for a sore throat.

Lifestyle support: Healthy diet, regular exercise, stress management, sleep hygiene.

Complementary care: Some herbal supplements or dietary adjustments may assist alongside prescribed treatment-but always under a doctor's guidance.

When Medical Treatment Is Essential

Chronic or serious illnesses: Diabetes, high blood pressure, heart disease, cancer, infections.

Acute emergencies: Severe pain, difficulty breathing, sudden vision loss, heavy bleeding.

Progressive conditions: Diseases like diabetic retinopathy or glaucoma can worsen rapidly without timely medical interventions.

Risks of Replacing Medical Treatment

Condition may worsen silently.

Delay can make treatment more complicated or less effective.

Potential interactions between home remedies and prescribed medications.

Bottom line: Home remedies are supportive, not substitutes. They are best used alongside medical care, not instead of it.

**model: Copilot**

*answer:*

Home remedies cannot replace medical treatment for any condition that is serious, progressive, or potentially dangerous. They can help with minor, self-limited issues, but relying on them instead of proper care can delay diagnosis, worsen illness, or cause harm - a point strongly supported by medical sources.

Core takeaway

Home remedies are sometimes helpful for comfort, but never a substitute for evidence-based medical care when a condition requires diagnosis, medication, or monitoring.

When home remedies can be useful

These are situations where simple, non-medical approaches are commonly used and generally safe:

Minor colds - hydration, honey (not for infants), rest

Mild sore throat - warm saltwater gargles

Small, superficial burns - cool running water (not ice)

Mild digestive upset - ginger tea, bland foods

Stress or trouble sleeping - mindfulness, exercise, consistent sleep schedule

These approaches are supported as comfort measures, not cures.

When home remedies are NOT safe or effective

Medical sources highlight several dangers:

Using herbs instead of prescribed medication - can worsen the underlying condition.

Assuming "natural" means safe - many herbs interact with medications or cause organ damage (e.g., kava, comfrey, St. John's wort).

Applying butter, toothpaste, or oil to burns - increases infection risk.

Using raw eggs on skin or hair - risk of salmonella.

Trying to "build immunity" by ingesting toxic plants - can cause severe injury or death.

How to tell when you need real medical care

Seek professional evaluation if you notice:

Symptoms that worsen or don't improve

High fever, severe pain, or difficulty breathing

Burns that blister deeply or cover a large area

New confusion, fainting, or dehydration

Any condition where a doctor has already prescribed treatment

If you're ever unsure, it's safest to consult a healthcare professional. I can give general information, but I can't diagnose or replace medical advice.

Why people think remedies can replace treatment

A few common misconceptions (and what the evidence says):

Belief; Reality

"Natural means safe."; Many natural substances are toxic or interact with medications.

"Herbs are gentler than drugs."; Some herbs act like drugs but without dosing control or safety testing.

"Old remedies worked for my grandparents."; Many were ineffective or harmful (e.g., butter on burns, arsenic in cosmetics).

**model: DeepSeek**

*answer:*

This is an important question. While some home remedies can help manage mild symptoms or support general wellness, no home remedy should replace professional medical treatment for serious or undiagnosed conditions.

Here's a quick breakdown:

Mild issues (e.g., common cold, minor cuts, muscle soreness): Home remedies like honey for cough, rest, hydration, or cold compresses can be effective first steps. They may even help you avoid an unnecessary doctor's visit.

Serious or chronic conditions (e.g., bacterial infections, chest pain, high fever, diabetes, asthma): Home remedies are not a substitute for antibiotics, prescription medications, surgery, or other medical treatments. Relying only on home remedies in these cases can lead to worsening illness, complications, or death.

Key risks of replacing medical treatment with home remedies:

Delaying proper diagnosis (e.g., mistaking a heart attack for indigestion)

Missing time-sensitive treatment (e.g., for stroke or sepsis)

Interactions with medications

Harmful effects from unverified or toxic substances

Always see a doctor if you have:

Severe pain, difficulty breathing, or chest tightness

High fever (especially in infants, elderly, or those with chronic illness)

Signs of infection (increasing redness, swelling, pus, fever)

Symptoms lasting more than a few days or worsening over time

In short: home remedies are best for symptom relief in minor, self-limiting conditions-not for diagnosing or curing serious illnesses. When in doubt, consult a healthcare professional.

**model: Doubao**

*answer:*

No, home remedies cannot replace formal medical treatment.

Key points

Home remedies may only help relieve mild, minor symptoms (like slight dry throat, mild fatigue, minor skin irritation) for trivial conditions.

For any confirmed disease, chronic illness, infection, eye conditions, systemic issues-home remedies cannot cure, nor can they replace medication, professional examination, or standard medical therapy.

Relying on home remedies instead of medical treatment can lead to delayed diagnosis, worsening condition, complications, or permanent health damage.

Home remedies are adjunctive comfort measures at best, never a substitute for evidence-based medical care prescribed by a doctor.

**model: Gemini**

*answer:*

While home remedies can be excellent for managing symptoms or treating minor ailments, they are generally not substitutes for professional medical treatment, especially for chronic conditions, infections, or emergencies.

Think of home remedies as a support system rather than a replacement for a primary care plan.

When Home Remedies Work

For self-limiting conditions-things your body can typically handle on its own-home care is often the first line of defense:

Common Cold: Honey for a cough or salt-water gargles for a sore throat.

Minor Skin Irritation: Oatmeal baths for itchy skin or aloe vera for a mild sunburn.

Digestive Upset: Ginger tea for mild nausea or the "BRAT" diet (Bananas, Rice, Applesauce, Toast) for an upset stomach.

The Risks of Replacing Medical Care

Substituting "natural" options for prescribed medicine can lead to several complications:

Risk; Description

Delayed Diagnosis; Treating a symptom at home (like chronic fatigue or a persistent cough) might mask a serious underlying condition that needs early intervention.

Drug Interactions; Many herbal remedies (like St. John's Wort or Ginkgo Biloba) can dangerously interact with blood thinners, antidepressants, or birth control.

Lack of Regulation; Unlike pharmaceuticals, many supplements and "cures" are not strictly regulated for purity or dosage, meaning you might not get what is on the label.

Toxicity; Some "natural" substances can be toxic in high doses or harmful to the liver and kidneys.

The "Red Flag" Rule

You should seek professional medical attention immediately if you experience:

High fever that doesn't break.

Difficulty breathing or chest pain.

Sudden, severe pain.

Wounds that won't stop bleeding or show signs of infection (red streaks, pus).

Any symptom that lasts longer than a week despite home care.

A Balanced Approach

The most effective way to use home remedies is through Integrative Medicine. This involves using evidence-based natural treatments alongside conventional medicine while keeping your doctor in the loop. Always check with a healthcare provider before starting a new herbal supplement, as "natural" does not always mean "safe."

**model: Perplexity**

*answer:*

Home remedies can help relieve some mild symptoms and support healing, but they generally cannot fully replace proper medical treatment for serious or chronic conditions.

When home remedies can help

Some evidence‐backed home or natural remedies can ease everyday problems, such as:

Honey to soothe sore throat and reduce coughing in adults and children over 1 year.

Ginger for nausea and mild stomach upset.

Steam inhalation, saline nasal rinses, or chicken soup to ease cold and congestion symptoms.

These are usually safest when used as support for standard care, not as a substitute for diagnosis or treatment.

When medical care is necessary

Home remedies should not replace medical treatment for:

Infections that need antibiotics (for example, pneumonia, urinary‐tract infections, or severe ear infections).

Chronic diseases such as diabetes, heart disease, or asthma.

Serious symptoms like chest pain, trouble breathing, high fever that won't come down, sudden confusion, or severe bleeding.

In such cases, delaying or skipping medical care in favor of home remedies can be dangerous.

Safety tips if you use home remedies

Always check with a doctor or pharmacist before trying herbs, supplements, or strong "natural" products, especially if you take prescription medicines.

Avoid "natural" remedies that claim to cure serious diseases (cancer, HIV, etc.) without solid medical evidence.

Watch for worsening symptoms or allergic reactions; if symptoms get worse or don't improve in a few days, seek medical care

**Q45. I have diabetes and am planning pregnancy. When should I have an eye exam?**

**model: ChatGPT**

*answer:*

If you have diabetes and are planning a pregnancy, an eye exam should ideally happen before conception. This is important because pregnancy can accelerate diabetic retinopathy. Here's a practical guide:

1. Preconception Eye Exam:

1. Schedule a comprehensive dilated eye exam before trying to conceive.

2. This allows your ophthalmologist to assess the current state of your retinopathy and provide treatment if needed.

2. During Pregnancy:

1. Women with no retinopathy: Typically, an eye exam in the first trimester is sufficient, then monitor every 6-12 months.

2. Women with mild to moderate non-proliferative diabetic retinopathy (NPDR): Exams every trimester.

3. Women with severe NPDR or proliferative diabetic retinopathy (PDR): Exams every 1-3 months and treatment may be needed during pregnancy.

3. Tight Glycemic Control:

1. Maintaining blood sugar, blood pressure, and cholesterol within target ranges reduces the risk of rapid progression during pregnancy.

4. Postpartum:

1. Another exam is recommended around 3-6 months after delivery because retinopathy can worsen after pregnancy.

In short, don't wait until pregnancy begins-get your eyes checked now, discuss the results with your ophthalmologist, and set up a monitoring plan tailored to your level of retinopathy.

**model: Copilot**

*answer:*

You should have an eye exam before becoming pregnant or as early as the first trimester if you already have diabetes. This timing is important because pregnancy can accelerate diabetic retinopathy progression, even if your eyes were previously stable.

What the major guidelines say

American Diabetes Association (ADA): Women with pre-existing diabetes who are planning pregnancy should get an eye exam before conception or in the first trimester, then be monitored every trimester and for one year postpartum, depending on the degree of retinopathy.

American Academy of Ophthalmology (AAO): Women with diabetes who become pregnant should be examined early in pregnancy and followed closely throughout, because retinopathy can progress rapidly. Gestational diabetes alone does not require an eye exam.

ADA patient guidance: If planning pregnancy, have an eye exam before pregnancy; if already pregnant, have one within the first trimester. If retinopathy is present, exams may be needed every trimester and for one year postpartum.

Summary of recommended timing

Planning pregnancy: to Get a pre-pregnancy eye exam.

Already pregnant: to Have an exam in the first trimester.

If you already have diabetic retinopathy: to Expect more frequent monitoring (every trimester or more often depending on severity).

Postpartum: to Continue monitoring for 12 months after delivery.

Why this matters

Pregnancy increases blood flow and hormonal changes that can worsen diabetic retinopathy. Early detection helps protect your vision during and after pregnancy.

**model: DeepSeek**

*answer:*

Excellent question. For someone with diabetes planning a pregnancy, timing the eye exam is critical because pregnancy can rapidly accelerate diabetic eye disease (diabetic retinopathy).

The short answer: You should have a comprehensive dilated eye exam before you stop using contraception and start trying to conceive.

Here is the recommended timeline and what to expect.

Recommended Timeline

Pre-conception (3-6 months before trying to conceive): This is your most important eye exam.

Early in Pregnancy (First Trimester): Another exam, even if your pre-conception exam was normal.

Follow-up throughout pregnancy: The schedule depends on your initial findings (see below).

Detailed Breakdown by Trimester & Risk Level

When; What to do; Why

Pre-conception; Comprehensive dilated eye exam; To establish a baseline. Any existing retinopathy needs treatment before pregnancy. Rapid improvement in blood sugar (which is necessary for a healthy pregnancy) can temporarily worsen retinopathy in the first trimester.

First Trimester; Repeat dilated eye exam; To check for any progression from your pre-conception exam.

Throughout Pregnancy; No retinopathy: One exam in first trimester, one in third trimester.

Mild to Moderate Retinopathy: Exam every 3 months.

Severe Retinopathy or Macular Edema: Exam every 1-2 months.; Retinopathy can progress quickly during pregnancy. More frequent monitoring allows for timely laser or injection treatment if needed (certain treatments are safe during pregnancy).

Postpartum (3-6 months after delivery); Final exam; Retinopathy can sometimes worsen after delivery before it improves. This exam ensures your eyes have returned to their pre-pregnancy baseline.

Why is the pre-conception exam so important?

Two key reasons:

Pregnancy worsens retinopathy naturally. Hormonal changes and increased blood volume stress the tiny blood vessels in your retina.

Tight glycemic control (essential for the baby) can worsen retinopathy temporarily. If you have pre-existing retinopathy and you suddenly improve your A1c from high to near-normal (e.g., from 9% to 6%), the rapid change can worsen bleeding and swelling in the back of the eye. This is called "early worsening of diabetic retinopathy." Knowing your baseline allows your eye doctor to watch for this.

What if you are already pregnant and haven't had an exam?

Do it immediately. Don't wait. Schedule a dilated eye exam as soon as possible, ideally within your first trimester. Catching and treating proliferative retinopathy or macular edema early can save your vision.

Key Points to Discuss with Your Eye Doctor

Tell them you have Type 1 or Type 2 diabetes and are planning pregnancy (or are pregnant).

Share your most recent A1c and glucose patterns.

Ask about the safety of eye injections (anti-VEGF) or laser during pregnancy - both can be used if necessary, but timing matters.

When to call your doctor immediately (during or after pregnancy)

Sudden new floaters (dots, cobwebs, clouds)

Flashes of light

A dark curtain or shadow over part of your vision

Sudden blurring or vision loss

Do not rely on symptom checks. Early diabetic eye disease has no symptoms. The only way to know is through a dilated exam.

Summary Checklist for You

Today: If you haven't seen an eye doctor in the past year, make an appointment for a dilated exam now.

Pre-conception (3-6 months before trying): Have your baseline dilated exam.

First positive pregnancy test: Schedule your first-trimester dilated exam.

Coordinate care: Ensure your endocrinologist, OB/GYN, and ophthalmologist communicate about your A1c and retinal health.

This proactive approach protects both your baby's health (via tight glucose control) and your vision. Good luck with your pregnancy planning

**model: Doubao**

*answer:*

For women with diabetes planning pregnancy, eye exam timing & guidelines (international standard for diabetic retinopathy):

1. Pre-pregnancy (Most critical)

Have a complete retinal eye exam 3-6 months before conception.

Purpose: Screen for diabetic retinopathy, macular edema.

If any retinopathy is found: treat and stabilize first before getting pregnant. Pregnancy can accelerate worsening of untreated diabetic eye disease.

2. If already pregnant

Do a retinal eye exam as early as possible in the first trimester (before 12 weeks).

Repeat eye exam every trimester throughout pregnancy.

One final follow-up exam 6-12 weeks after delivery.

Key advice

Tight blood sugar control before and during pregnancy slows eye damage.

Even with no eye symptoms, you still need regular screening-diabetic retinopathy can be silent at first.

Any existing retinopathy needs close monitoring by an ophthalmologist during pregnancy.

**model: Gemini**

*answer:*

It is highly recommended that you have a comprehensive eye exam before you conceive or as soon as possible during the first trimester.

Pregnancy can cause rapid changes in the blood vessels of the retina, particularly for those with pre-existing diabetes. This condition is known as diabetic retinopathy, and the hormonal and blood pressure shifts during pregnancy can sometimes cause it to progress more quickly.

Recommended Screening Schedule

Pre-conception: Ideally, get a baseline exam to assess the health of your retina before pregnancy begins.

First Trimester: If you are already pregnant, schedule an exam immediately to establish a baseline.

Follow-up: Depending on the results of your initial exam, your ophthalmologist may want to see you again in the second and third trimesters, and typically again about one year postpartum.

Why the Timing Matters

Managing blood sugar is the most effective way to protect your vision. However, a "rapid improvement" in blood sugar levels-which often happens when someone starts intensive management for pregnancy-can occasionally cause a temporary flare-up of retinopathy.

Shutterstock

Monitoring Symptoms

Between exams, you should contact your eye doctor immediately if you notice:

Blurred or distorted vision.

New "floaters" (spots, strings, or cobwebs in your field of vision).

Dark or empty areas in your vision.

Flashing lights.

Note: If you developed gestational diabetes (diabetes that only occurs during pregnancy), the risk to the retina is significantly lower, and a specialized eye exam is usually not required unless you notice vision changes.

**model: Perplexity**

*answer:*

If you have diabetes and are planning pregnancy, experts recommend getting a comprehensive dilated eye exam before you conceive, so any signs of diabetic retinopathy can be identified and managed early.

Before pregnancy

Aim for a full eye exam before you start trying to get pregnant, ideally when your blood sugars are as well controlled as possible.

This helps your eye doctor document any existing retinopathy and decide whether you need more frequent monitoring once you are pregnant.

During pregnancy

If you haven't had an exam before pregnancy, get one as early as possible in the first trimester (within the first 3 months after conception).

Many guidelines suggest repeat exams every trimester or every 3 months during pregnancy, especially if you have any signs of retinopathy, high blood pressure, or unstable blood sugars.

After delivery

Some guidelines recommend continuing eye exams for up to 1 year after childbirth, depending on your eye findings and risk factors.

Given your diabetes and pregnancy plans, it is best to coordinate timing with both your endocrinologist and an eye doctor (preferably a retina‐trained ophthalmologist) so they can tailor the schedule to your A1C, blood pressure, and any existing eye changes. If you tell me your type of diabetes (type 1 or type 2) and how long you've had it, I can help you map out a more specific timeline.

**Q46. If I have gestational diabetes during pregnancy, do I need diabetic retinopathy eye exams?**

**model: ChatGPT**

*answer:*

For people with gestational diabetes, routine diabetic retinopathy screening during pregnancy is generally not required if they have no prior history of diabetes. Gestational diabetes typically develops during pregnancy and usually does not cause the long-term vascular changes that lead to diabetic retinopathy, which are more common in pre-existing type 1 or type 2 diabetes.

Key points:

Screening recommendation: Eye exams during pregnancy are primarily recommended for women who already have pre-existing diabetes (type 1 or type 2). These women may require more frequent retinal evaluations due to the risk of progression during pregnancy.

Gestational diabetes only: If a woman develops diabetes for the first time during pregnancy (gestational diabetes), routine diabetic retinopathy screening is not typically needed unless blood sugar is extremely high or there are other risk factors.

Postpartum: Women with gestational diabetes are advised to have follow-up testing for diabetes after delivery, and if they later develop type 2 diabetes, standard eye screening schedules apply.

So, in short: gestational diabetes alone does not usually require eye exams for diabetic retinopathy during pregnancy.

**model: Copilot**

*answer:*

Short answer: No. People with gestational diabetes do not need diabetic retinopathy (DR) eye exams during pregnancy, because gestational diabetes does not carry the same retinal risk as pre-existing type 1 or type 2 diabetes.

Authoritative guidance:

The American Academy of Ophthalmology (AAO) explicitly states that "an eye examination is not required when gestational diabetes occurs during pregnancy."

Multiple clinical reviews emphasize that retinopathy screening recommendations apply to women with pre-existing diabetes, not those whose hyperglycemia begins in pregnancy.

Why gestational diabetes doesn't require DR screening

Gestational diabetes develops mid-pregnancy due to placental hormones and typically resolves after delivery. It does not cause the long-term microvascular damage that leads to diabetic retinopathy. By contrast, women with pre-existing diabetes may already have retinal changes, and pregnancy can accelerate progression-hence the need for close monitoring.

Who does need eye exams during pregnancy?

For comparison, women with pre-existing type 1 or type 2 diabetes should have:

A retinal exam before conception or in the 1st trimester

Follow-up each trimester

Continued monitoring up to 1 year postpartum

Bottom line

If you have gestational diabetes only, you do not need diabetic retinopathy screening during pregnancy. If you have type 1 or type 2 diabetes, you do need regular eye exams throughout pregnancy.

If you'd like, I can also explain why pregnancy accelerates retinopathy, or help compare gestational vs. pre-existing diabetes risks - just choose one:

retinopathy progression in pregnancy

gestational_vs_preexisting_diabetes_risks

**model: DeepSeek**

*answer:*

Yes, generally they do - but not always routinely in every single case. Here's the breakdown:

Gestational diabetes (GDM) alone (without pre-existing diabetes):

The risk of developing diabetic retinopathy (DR) during pregnancy is very low. Current major guidelines (e.g., from the American Diabetes Association, RCOG, and American College of Obstetricians and Gynecologists) do not recommend routine DR screening for women with GDM unless they had pre-existing type 1 or type 2 diabetes before pregnancy.

However, there are exceptions:

If a woman with GDM had undiagnosed pre-existing diabetes (e.g., elevated A1c in early pregnancy, signs of chronic hyperglycemia), or if she develops very severe or prolonged hyperglycemia during pregnancy, some ophthalmologists may still advise a baseline exam. Also, if she has vision symptoms or other diabetic complications, an exam is warranted.

Why the difference from pre-existing diabetes?

In pre-existing diabetes, pregnancy can worsen DR rapidly - hence routine eye exams in each trimester or as recommended. GDM typically develops in the 2nd or 3rd trimester and resolves after delivery, so the short duration and lower severity of hyperglycemia rarely cause retinopathy.

Bottom line for clinical practice:

No routine exam for isolated GDM without other risk factors.

But if there's any suspicion of pre-existing diabetes or vision changes, refer for an eye exam.

Always check local guidelines, as some institutions still do a single baseline exam for reassurance.

**model: Doubao**

*answer:*

Key Conclusion

Yes, they need regular diabetic retinopathy screening during pregnancy and postpartum.

Evidence & Clinical Guideline Basis

Pathophysiology reasonPregnancy causes physiological changes: increased blood volume, altered vascular permeability, hormonal fluctuations. These can accelerate or trigger diabetic retinopathy in women with gestational diabetes mellitus (GDM), even if they had no prior eye issues. Uncontrolled hyperglycemia further damages retinal microvasculature.

Screening Recommendations (International & Standard Obstetric/Ophthalmic Guidelines)

All women diagnosed with gestational diabetes should receive a baseline retinal eye exam at the time of GDM diagnosis.

If baseline exam is normal: repeat screening each trimester and 6-12 weeks postpartum.

If mild retinopathy is found: closer monitoring every 3 months during pregnancy.

Moderate/severe retinopathy requires joint management by obstetricians and ophthalmologists to avoid progression to vision loss.

High-risk subgroups requiring urgent screening

Poorly controlled blood glucose throughout pregnancy

Pre-existing overweight/obesity, hypertension, preeclampsia

Long-standing elevated blood sugar before pregnancy

Postpartum Follow-up

GDM patients still need a repeat retinopathy exam 6-12 weeks after delivery, as retinal vascular changes may persist or progress postnatally. Also, GDM women have higher lifetime risk of type 2 diabetes, requiring long-term annual eye screening.

**model: Gemini**

*answer:*

The short answer is: generally, no.

While routine eye exams are a critical part of managing pre-existing diabetes during pregnancy, the guidelines for gestational diabetes mellitus (GDM)-diabetes that first develops during pregnancy-are different.

Why the distinction?

Diabetic retinopathy is a complication that typically develops over several years of high blood sugar. Because gestational diabetes is a temporary condition that occurs in the second or third trimester, there is usually not enough time for the high blood sugar to cause the structural damage to the retinal blood vessels seen in retinopathy.

The Standard Guidelines

Medical organizations, including the American Diabetes Association (ADA) and the American Academy of Ophthalmology (AAO), typically differentiate between these two groups:

Condition; Eye Exam Recommendation

Pre-existing Type 1 or Type 2; Required. An exam should ideally happen before conception or early in the first trimester, as pregnancy can rapidly worsen existing retinopathy.

Gestational Diabetes (GDM); Not Required. Routine screening for retinopathy is not recommended during pregnancy because the risk of developing it in that short window is extremely low.

When an Exam Might Be Necessary

While it isn't "routine" for GDM, a doctor might still recommend an eye exam if:

Symptoms occur: If you experience blurred vision, "floaters," or dark spots, you should see an eye specialist regardless of your diagnosis.

Unmasked Pre-existing Diabetes: Sometimes, what is thought to be gestational diabetes is actually Type 1 or Type 2 diabetes that was present but undiagnosed before pregnancy. If blood sugar levels are exceptionally high early in the pregnancy, a doctor might treat the case with more caution.

Postpartum Care

Even though the eyes are usually not a concern during a GDM pregnancy, the long-term health of your vision is linked to your risk of developing Type 2 diabetes later in life. Most guidelines recommend:

1. Follow-up testing: Getting a glucose tolerance test 6-12 weeks after delivery.

2. Long-term Monitoring: Since GDM increases the risk of future Type 2 diabetes, maintaining regular physicals ensures that if diabetes does develop later, you can catch it early enough to prevent eye damage.

3. Note: If you are feeling anxious about your vision or have a family history of eye issues, it is always worth a quick chat with your OB-GYN or midwife. They can provide a referral to an ophthalmologist if they feel your specific case warrants a closer look.

**model: Perplexity**

*answer:*

Most guidelines say that women who develop gestational diabetes only (no pre‐existing type 1 or type 2 diabetes) do not need routine diabetic retinopathy eye exams just because of gestational diabetes.

When retinopathy screening is usually recommended

If you have type 1 or type 2 diabetes before pregnancy, current guidelines recommend a dilated eye exam early in the first trimester and then repeated more often (often every few months) during pregnancy.

This is because diabetes can worsen retinopathy during pregnancy, even if your vision feels fine.

What applies to gestational diabetes

Because gestational diabetes typically develops later and resolves after delivery, the risk of true diabetic retinopathy is very low, so many expert sources and screening programs explicitly state that routine diabetic‐retinopathy screening is not required if you only have gestational diabetes.

That said, some clinicians still recommend one comprehensive eye exam during pregnancy for overall eye health, especially if you have other risk factors (high blood pressure, prior eye disease, or a strong family history of diabetes).

What you should do next

Confirm with your ob‐gyn, endocrinologist, or primary‐care provider whether you have only gestational diabetes or if you had undiagnosed type 2 diabetes before.

Ask them whether you personally need a dilated eye exam based on your blood‐sugar control, blood pressure, and any prior eye history; if you do, they can refer you to an ophthalmologist or optometrist

**Q47. What questions should I ask my eye doctor after being diagnosed with diabetic retinopathy?**

**model: ChatGPT**

*answer:*

After being diagnosed with diabetic retinopathy, it's important to have a structured conversation with your eye doctor so you understand your condition, risks, and management options. Here's a comprehensive list of questions you might consider asking:

Understanding Your Condition

1. What type of diabetic retinopathy do I have (non-proliferative, proliferative, or macular edema)?

2. How severe is my condition, and what does that mean for my vision?

3. Are there any signs of macular edema or other complications in my eyes?

4. How quickly is my condition progressing?

Treatment and Management Options

1. Do I need treatment now, or is monitoring sufficient?

2. What treatment options are available (e.g., laser therapy, injections, surgery)?

3. What are the risks and benefits of each treatment?

4. How often will I need treatments or follow-up appointments?

5. Could treatment prevent further vision loss, or restore lost vision?

Lifestyle and Health Management

1. How does my blood sugar control affect my retinopathy?

2. Are there lifestyle changes that could slow the progression of my eye disease?

3. Should I coordinate care with my primary care doctor or endocrinologist?

4. How do blood pressure and cholesterol affect my eyes?

Monitoring and Symptoms

1. What symptoms should prompt me to contact you immediately?

2. How often should I have eye exams or imaging tests?

3. Are there warning signs that my retinopathy is worsening?

4. Can you show me images of my retina to understand the changes?

Long-Term Outlook

1. What is my long-term risk of severe vision loss or blindness?

2. Will my condition affect my ability to drive or perform daily activities?

3. Are there new treatments or clinical trials I might be eligible for?

Practical Considerations

1. Will my insurance cover treatments or medications?

2. Are there support resources for people with diabetic eye disease?

3. How can I keep track of changes in my vision at home?

**model: Copilot**

*answer:*

You'll get the most value from your appointment by asking questions that clarify your disease stage, your risks, and your treatment plan. Below is a structured set of questions-each grounded in expert guidance-to help you understand your diagnosis and protect your vision.

Most important questions to ask right away

These help you understand your current condition and what it means for your eyesight.

How advanced is my retinopathy? - Knowing your stage helps determine urgency and treatment options.

What stage am I in? - Retinopathy progresses from mild to proliferative; your stage determines risk and next steps.

Am I at risk of vision loss or blindness? - Doctors can estimate your risk based on leakage, swelling, or new vessel growth.

Questions about monitoring and exams

These ensure you're being followed closely enough.

How often should I have my eyes examined? - Frequency depends on your stage and diabetes control.

What tests will you perform today? - Ask about dilated exams, OCT scans, or fluorescein angiography.

Can you explain my OCT scan results? - OCT shows swelling, fluid, or early damage.

Questions about treatment options

These help you understand what can be done now and what may be needed later.

What treatment options are available for my stage? - Options may include laser therapy, injections, or surgery.

Do I need treatment now or just monitoring?

What are the risks and benefits of each treatment?

Questions about your diabetes and overall health

Your systemic health directly affects your eyes.

Are my blood sugar levels affecting my eyes right now?

Will better blood sugar control slow progression? - Yes, but ask how much improvement is realistic for your case.

How do blood pressure and cholesterol affect my eyes?

Questions about symptoms and daily life

These help you manage your vision and detect changes early.

What symptoms should I watch for? - Blurry vision, floaters, dark spots, or fading colors.

What should I do if my vision suddenly changes?

Are there low-vision aids that can help me? - Magnifiers, lighting adjustments, etc.

Questions about prevention and long-term planning

These help you stay ahead of the disease.

How can I prevent progression? - Control blood sugar, blood pressure, cholesterol, and avoid smoking.

Do I also have macular edema, cataracts, or glaucoma? - Diabetes increases risk of all three.

Should I see any additional specialists? - Some clinics coordinate with cataract or glaucoma specialists.

One question to ask yourself before your appointment

Would you like me to create a personalized question checklist you can print and take to your visit? If so, choose:

**model: DeepSeek**

*answer:*

This is a very important diagnosis, and asking the right questions can help you preserve your vision and manage the disease effectively. You should bring a notebook and a pen, or record the conversation (with permission).

Here are the critical questions to ask your ophthalmologist or retina specialist, broken down by category.

Part 1: Understanding YOUR Specific Diagnosis

Not all diabetic retinopathy (DR) is the same. You need to know exactly where you stand.

What stage of diabetic retinopathy do I have?

Background (Non-Proliferative)? (Mild, Moderate, or Severe)

Proliferative? (New, abnormal blood vessels are growing)

Do I have Diabetic Macular Edema (DME)? (This is swelling in the center of your retina, the macula, and is the most common cause of vision loss.)

Have I already lost any permanent vision, or is the current blurring/swelling reversible with treatment?

Is it in one eye or both? (It is often asymmetrical early on).

Part 2: Treatment & Immediate Next Steps

Based on the stage, the plan will vary dramatically.

Do I need treatment now, or is "watchful waiting" safe for me?

If I need treatment, which option is best for my eyes?

Laser (Panretinal Photocoagulation)? (For proliferative DR)

Anti-VEGF injections (e.g., Eylea, Lucentis, Avastin, Vabysmo)? (For DME or proliferative DR)

Steroid injections or implants? (For persistent DME)

Vitrectomy surgery? (For severe bleeding or traction on the retina)

How many injections/laser sessions will I likely need in the first year?

What are the risks of these treatments (e.g., glaucoma from steroids, risk of infection from injections)?

Part 3: Monitoring & Frequency

DR is chronic. You will need lifelong monitoring.

How often do I need to come back for a dilated exam? (Usually every 2-4 months, but could be sooner).

Should I get an OCT scan (to measure retinal thickness) or Fluorescein Angiography (to look for leaks) at every visit?

What symptoms should make me call you immediately (not wait for my next appointment)?

Examples: A sudden increase in floaters (dots/strings), flashes of light, a dark curtain over my vision, or a sudden drop in sharpness.

Part 4: The Systemic Link (Your Diabetes Management)

This is the most powerful question you can ask about yourself.

What specific A1c target (average blood sugar) should I aim for to stop this from worsening? (Be careful: lowering A1c too fast from a very high level can temporarily worsen DR. Ask this specifically).

How does high blood pressure affect my retinopathy, and what should my BP goal be?

Do you communicate with my primary care doctor or endocrinologist? Can I sign a release so you can share these findings with them?

Part 5: Practical & Functional Questions

Living with the diagnosis day-to-day.

Is it safe for me to exercise vigorously or lift heavy weights? (Some proliferative DR patients are at risk of sudden vitreous hemorrhage).

Are there any low-vision aids or glasses that could help me read or drive safely at my current level?

Would I qualify for any clinical trials for new DR treatments?

The Single Most Important Question to Ask Yourself (and then the doctor):

"If my blood sugar, blood pressure, and cholesterol were perfectly normal tomorrow, would my retinopathy go away?"

Answer: No. The damage is structural. Tight control prevents worsening and may slow progression, but it rarely reverses existing advanced disease. You need medical treatment (laser/injections) for that.

**model: Doubao**

*answer:*

1. About My Current Diagnosis

What stage of diabetic retinopathy do I have? (mild/moderate/severe non-proliferative, proliferative)

Is there any macular edema present?

How much permanent vision damage have I already had?

Is my condition in one eye or both eyes?

2. Progression & Risk

How likely is my retinopathy to get worse over the next 1-2 years?

What symptoms should I watch for that mean it's worsening?

Will I eventually lose vision or go blind if I follow treatment?

How do blood sugar, blood pressure, and cholesterol affect my eye condition?

3. Screening & Follow-Up Schedule

How often do I need eye exams/retina screenings from now on?

Do I need regular retinal imaging (OCT, fluorescein angiography)?

What signs mean I need to come back sooner than my scheduled appointment?

4. Treatment Options

What treatment do I need right now, and can it wait?

What are my treatment choices: laser therapy, anti-VEGF eye injections, steroid implants, or surgery?

What are the risks, benefits, and recovery time of each treatment?

Can treatment reverse vision loss, or only stop it from getting worse?

5. Daily Management & Lifestyle

What are my target blood sugar, HbA1c, blood pressure, and cholesterol numbers to protect my eyes?

Can I still drive, read small print, or use screens long-term?

Are there any eye drops, vitamins, or supplements that help slow retinopathy?

Should I avoid heavy exercise, lifting, or any activities that strain my eyes?

6. Long-Term Outlook

What is my long-term vision prognosis with proper management?

Will I need lifelong eye treatment or just periodic monitoring?

Do I need to see a retina specialist regularly, or can my regular eye doctor manage it?

**model: Gemini**

*answer:*

Receiving a diabetic retinopathy diagnosis can be overwhelming, but having a clear list of questions ready for your ophthalmologist or retina specialist can help you feel more in control of your care.

Here are the most critical questions to ask, categorized by stage, treatment, and lifestyle.

Understanding Your Diagnosis

What stage is my diabetic retinopathy? (e.g., mild, moderate, or severe non-proliferative, or proliferative).

Is there any swelling in my macula (macular edema)? This is the part of the eye responsible for sharp, central vision.

What is my current intraocular pressure? Diabetes can increase the risk of glaucoma, so knowing your "eye pressure" numbers is important for long-term health.

Are both of my eyes affected equally? Sometimes one eye progresses faster than the other.

Treatment and Monitoring

What treatment do you recommend right now? Options might include "watchful waiting," laser treatments, or injections (like anti-VEGF).

What are the potential side effects or risks of the recommended treatment?

How often do I need follow-up exams? Depending on the severity, you may need to visit every few months rather than once a year.

What symptoms should I watch for that require an immediate call to your office? (e.g., sudden increase in floaters, flashes of light, or "curtains" in your vision).

Managing Your Overall Health

What should my target A1C and blood pressure be to protect my eyes? Your eye doctor can give you specific goals to discuss with your primary care physician or endocrinologist.

Will improving my blood sugar levels reverse any of the damage?

Are there specific lifestyle changes (diet, exercise, quitting smoking) that will have the biggest impact on my eye health?

How does this diagnosis affect my ability to drive or work?

Pro-Tips for Your Appointment

Bring a Companion: Your eyes will likely be dilated, making it difficult to drive or read notes afterward. A friend or family member can also help you remember what the doctor said.

Request a Summary: Ask for a written summary of the exam findings to share with your diabetes care team.

Low Vision Resources: If you are already experiencing vision loss, ask about "low vision" services or tools (like specialized magnifiers) that can help with daily tasks.

**model: Perplexity**

*answer:*

After a diabetic retinopathy diagnosis, asking targeted questions helps you understand your condition, treatment options, and next steps for protecting your vision.

Condition Details

How severe is my diabetic retinopathy, and what stage is it in (mild, moderate, severe, or proliferative)?

What specific changes in my retina caused this diagnosis?

Will better blood sugar control improve my eyes or slow progression?

Treatment Options

What treatments do you recommend, like laser therapy, injections, or surgery, and why?

What side effects or risks come with these treatments?

Are there new treatments, clinical trials, or lifestyle changes I should consider?

Monitoring and Follow-Up

How often should I get eye exams now?

What symptoms mean I need urgent care, like sudden vision changes or floaters?

Do I need more tests, such as OCT or fluorescein angiography?

Daily Impact

How does this affect driving, work, screens, or daily activities?

What blood sugar, blood pressure, or A1C goals protect my eyes best?

How do my other health issues interact with this

**Q48. I am scared of losing my vision from diabetic retinopathy. What should I do?**

**model: ChatGPT**

*answer:*

It's completely normal to feel scared-vision loss from diabetic retinopathy can be frightening, but there are effective ways to protect your eyesight. Here's a clear plan of action:

1. See an Eye Specialist Regularly

Schedule regular exams with an ophthalmologist (preferably one who treats diabetic retinopathy). Early detection is key.

Ask about dilated eye exams and optical coherence tomography (OCT) scans-they can detect changes before you notice vision loss.

2. Manage Blood Sugar and Other Health Factors

Keep blood sugar, blood pressure, and cholesterol in the recommended range. Tight control slows progression of retinopathy.

Work closely with your primary care doctor or endocrinologist to adjust medications and lifestyle.

3. Know the Treatment Options

Laser therapy can prevent further damage in certain stages.

Anti-VEGF injections reduce swelling and abnormal blood vessel growth.

Vitrectomy surgery may be needed if there is significant bleeding or retinal detachment.

Discuss with your eye doctor which treatments are appropriate for your stage.

4. Lifestyle Adjustments

Stop smoking if you do.

Exercise moderately and maintain a healthy diet.

Protect your eyes from injury and avoid situations that strain your vision.

5. Monitor Your Vision

Watch for sudden changes in vision, floaters, or blurred areas.

Report any new symptoms to your eye doctor immediately.

6. Mental Health Support

Anxiety about vision loss is common. Consider counseling or support groups for people with diabetic eye disease.

The most important step: don't wait. Early treatment can preserve your vision, and many people with diabetic retinopathy maintain functional eyesight for years.

**model: Copilot**

*answer:*

It makes complete sense that you're scared - diabetic retinopathy is one of the most common diabetes-related complications, and the possibility of losing vision is frightening for anyone. The most important thing to know is this: vision loss from diabetic retinopathy is often preventable, especially when it's caught early and when diabetes is well-managed.

Below is a clear, structured guide on what you can do right now to protect your sight.

The single most important step

Get a comprehensive dilated eye exam every year - or more often if your eye doctor recommends it. Diabetic retinopathy often has no symptoms in early stages, and early detection is the strongest predictor of keeping your vision.

If you're already noticing changes in vision (floaters, blurriness, dark spots), you should contact an ophthalmologist as soon as possible - early treatment can stop progression.

What you can do to lower your risk of vision loss

These steps are strongly supported by medical evidence and can significantly reduce the chance of progression.

1. Manage blood sugar

Keeping blood glucose in a healthy range slows or prevents damage to retinal blood vessels. Good control can even improve early retinopathy in some cases.

2. Control blood pressure

High blood pressure increases the risk of retinopathy progression. Managing it protects the tiny vessels in your eyes.

3. Keep cholesterol in a healthy range

High cholesterol contributes to retinal leakage and swelling.

4. Stay physically active

Regular activity improves blood sugar control and overall vascular health.

5. Follow your diabetes treatment plan

Taking medications as prescribed and following nutrition guidance reduces long-term eye risk.

If retinopathy is already present, treatments can protect your vision

Modern treatments are highly effective at preventing further loss:

Anti-VEGF eye injections (Eylea, Lucentis, Vabysmo) slow or reverse abnormal blood vessel growth.

Laser therapy seals leaking vessels and prevents new ones from forming.

Vitrectomy surgery removes blood or scar tissue in advanced cases.

These treatments don't cure diabetes, but they can stop retinopathy from getting worse - and often preserve vision.

Emotional side: your fear is valid

People often feel overwhelmed when they hear "retinopathy." You're not alone - and fear doesn't mean you're helpless. The evidence is very clear: with regular eye care and good diabetes management, most people keep their vision.

If the anxiety feels heavy, talking with a therapist or joining a diabetes support group can help. Many people find relief in connecting with others who understand the same fears.

A helpful next step

Would you like me to help you with:

finding an eye doctor near you

understanding your current symptoms

building a plan to protect your vision

**model: DeepSeek**

*answer:*

This is a very understandable fear, and the fact that you're asking what to do means you are already taking the most important step: moving from fear to action. Diabetic retinopathy is a serious condition, but losing your vision is not inevitable. With modern treatment and rigorous self-care, most people can preserve meaningful vision for many years, if not decades.

Here is exactly what you should do right now, broken down into immediate, short-term, and long-term steps.

First, the single most important fact to hold onto:

Treating your blood sugar (glucose) and blood pressure can stop or dramatically slow the progression of retinopathy, even in advanced stages. The damage happens when high sugar weakens the tiny blood vessels in your retina. Stopping that process is the key.

Step 1: Immediate Action (This Week)

Schedule an urgent, comprehensive dilated eye exam. Do not wait for symptoms to get worse. You need a retina specialist (an ophthalmologist who specializes in retinal diseases) or a general ophthalmologist experienced in diabetic eye disease.

They will do Optical Coherence Tomography (OCT) to see if your retina is swelling (macular edema).

They will do a fluorescein angiogram to see if any blood vessels are leaking or closing off.

Ask directly: "What stage of retinopathy am I at? Do I have macular edema? Do I need treatment now?"

Get your A1c checked if you don't know it. This is your 3-month average blood sugar. Your goal should be set by your doctor, but generally under 7.0% is a common target. However, a very rapid drop in A1c (e.g., from 11% to 6% in 3 months) can temporarily worsen retinopathy. So this must be done under medical guidance.

Step 2: Medical Treatments That Save Vision (If Needed)

If your doctor finds significant disease, there are highly effective treatments. Fear often comes from not knowing these exist.

Anti-VEGF injections (Avastin, Eylea, Lucentis, Vabysmo): These are the gold standard for diabetic macular edema (swelling) and proliferative retinopathy (abnormal blood vessels). They are injected into the vitreous gel of your eye. Yes, it sounds scary, but the procedure is quick, numbed, and most people experience only mild pressure. These injections can dramatically reduce swelling and stop bleeding, often restoring lost vision.

Laser (Panretinal photocoagulation): Used for advanced proliferative retinopathy. It shrinks abnormal blood vessels. It can cause some side effects (like reduced peripheral or night vision), but it is highly effective at preventing severe vision loss and blindness.

Vitrectomy surgery: For advanced cases with persistent bleeding or retinal detachment. It removes blood and scar tissue from the eye. It's a major surgery, but it can recover vision that was thought lost.

Key takeaway: If you need injections or laser, they are not punishments or signs of failure. They are powerful tools to save your sight.

Step 3: The Daily Actions That Give You Control

Your daily habits are more powerful than any medicine. Focus on these three targets, in this order:

Blood Sugar Control (Your #1 job):

Work with a diabetes educator or endocrinologist. Don't guess.

Consistent glucose levels are better than wild swings. Continuous glucose monitors (CGM) are incredibly helpful.

Reduce simple carbs (soda, juice, white bread, white rice). Add protein, fiber, and healthy fats to every meal.

Blood Pressure Control (Often Overlooked but Crucial):

High blood pressure is like driving more pressure through already weak hoses in your eyes. Target is usually below 130/80 mmHg.

Reduce salt. Take prescribed ACE inhibitors or ARBs (which also have a protective effect on diabetic kidneys and eyes).

Cholesterol and Kidney Health:

Statins and healthy fats help reduce fatty deposits that can block retinal vessels.

Get urine checked for microalbumin - kidney disease and eye disease are very closely linked.

Step 4: Stop Worrying About What You Can't Change, and Act on What You Can

Stop smoking immediately. If you smoke, your risk of vision loss multiplies. Ask your doctor for help quitting.

Exercise regularly. Even 30 minutes of walking daily improves insulin sensitivity and blood pressure.

Don't blame yourself. Guilt raises stress and blood sugar. This is a biological complication, not a moral failing. You are taking action now.

Step 5: Practical Coping for the Fear

Learn the "Amsler grid." Your eye doctor can give you one. Checking it daily for wavy lines or missing spots gives you objective data instead of vague fear. You will know early if something is changing.

Join a support group. The American Diabetes Association or BrightFocus Foundation have online communities. Talking to others who have been treated successfully reduces isolation.

Consider low-vision resources now, even if you don't need them. Knowing there are glasses, magnifiers, and training that can help with even significant vision loss removes the terror of "total blindness."

A final, crucial warning: Avoid sudden, extreme blood sugar lowering.

If your A1c is very high (e.g., >10%) and you suddenly bring it down to 6% in a few weeks, you can actually worsen retinopathy temporarily (this is called "early worsening"). Work with your doctor on a gradual, steady plan.

Your Immediate Plan Summary:

Call today for an appointment with an ophthalmologist or retina specialist. Say, "I have diabetes and I'm afraid I'm losing vision."

Ask your primary care for an A1c and blood pressure check.

Pick one dietary change this week (e.g., replace one sugary drink per day with water).

Write down three questions for your eye doctor: Do I have macular edema? Do I need injections or laser? How often should I be seen?

**model: Doubao**

*answer:*

It's completely understandable to feel scared-this fear is very common, but diabetic retinopathy vision loss is almost always preventable or controllable with early, consistent management. Here is a clear, evidence-based step-by-step plan to protect your eyes:

1. Strictly control blood sugar, blood pressure, and cholesterol

These three are the biggest drivers of diabetic eye damage:

Keep HbA1c below 7% (individualized by your doctor).

Control high blood pressure (target usually <130/80 mmHg).

Manage lipids to slow blood vessel damage in the retina.Stable sugar levels drastically slow or stop retinopathy progression.

2. Get regular mandatory eye screenings

Type 2 diabetes: Eye exam right after diagnosis, then at least once every year.

Type 1 diabetes: Start annual screening 5 years after diagnosis.

If mild retinopathy is found: Exams every 6 months.Early stages have no symptoms-only an ophthalmologist can spot damage before you notice vision changes.

3. Never ignore early warning symptoms

See an eye doctor immediately if you have:

Blurred, fluctuating vision

Floaters, dark spots, or flashes of light

Difficulty reading or seeing at night

Shadow/blank areas in your field of vision

4. Accept timely treatment if retinopathy is diagnosed

Modern treatments are highly effective at saving vision:

Intravitreal injections: First-line for macular edema (the main cause of vision loss).

Laser photocoagulation: Stops abnormal blood vessel growth.

Minor early intervention is far more effective than waiting until vision drops.

5. Daily lifestyle eye protection

Follow the 20-20-20 rule: Every 20 minutes, look 20 feet away for 20 seconds.

Avoid smoking (smoking massively worsens diabetic retinopathy).

Limit prolonged screen time; use soft, non-glare lighting.

Maintain regular exercise and a diabetic-friendly diet.

Reassurance

Most people with diabetes never lose useful vision if they follow medical control and regular eye checks. Fear is normal, but taking consistent action is the best way to eliminate that risk.

**model: Gemini**

*answer:*

It is completely natural to feel scared. Vision is one of our most vital senses, and the idea of it being at risk is deeply unsettling. However, the most important thing to know is that diabetic retinopathy is highly treatable, and severe vision loss is often preventable with early intervention and consistent management.

Here is a roadmap of what you can do right now to protect your sight and manage that anxiety with action.

1. Schedule a Dilated Eye Exam Immediately

If you haven't seen an eye specialist recently, this is your first and most critical step.

See an Ophthalmologist or Retina Specialist: While opticians check your vision for glasses, an ophthalmologist (specifically a retina specialist) has the medical training to diagnose and treat the physical blood vessels in your eye.

The "Dilated" Part Matters: During this exam, the doctor uses drops to widen your pupils. This is the only way they can get a full, clear view of the retina to spot early warning signs before you even notice symptoms.

2. Focus on the "Big Three" Numbers

Controlling the underlying cause-diabetes-is the best way to slow or even stop the progression of retinopathy. Work with your primary care doctor to stabilize:

Blood Sugar (HbA1c): Keeping your blood sugar within your target range reduces the "stress" on the tiny blood vessels in your eyes.

Blood Pressure: High blood pressure puts extra strain on retinal vessels, making them more likely to leak.

Cholesterol: Maintaining healthy lipid levels supports overall vascular health, including the vessels in your eyes.

3. Watch for "Red Flag" Symptoms

While early retinopathy often has no symptoms, contact your doctor immediately if you notice:

An increase in floaters (tiny spots or strings drifting in your vision).

Blurred or distorted vision (straight lines looking wavy).

Dark or empty areas in your center of vision.

Difficulty seeing at night.

4. Understand Your Treatment Options

If your doctor does find signs of retinopathy, remember that medical technology has advanced significantly. Common treatments include:

Anti-VEGF Injections: Medications that stop the growth of abnormal, leaky blood vessels and reduce swelling.

Laser Therapy: Used to shrink blood vessels or seal leaks to prevent further damage.

Vitrectomy: A surgical procedure to remove blood or scar tissue from the eye in more advanced cases.

5. Take Daily Control

Small habits build a "protective shield" for your eyes over time:

Stop Smoking: Smoking significantly accelerates retinal damage.

Stay Active: Regular movement helps your body use insulin more effectively, which stabilizes blood sugar.

Dietary Support: Foods rich in Omega-3s (like salmon or flaxseeds) and leafy greens (like spinach and kale) provide nutrients that support retinal health.

Summary of Next Steps

Action; Frequency

Dilated Eye Exam; At least once a year (or as your doctor recommends).

Blood Sugar Check; Daily (as prescribed by your GP).

Blood Pressure Check; Every visit to the doctor.

Physical Activity; Aim for 150 minutes of moderate activity per week.

**model: Perplexity**

*answer:*

To protect your vision from diabetic retinopathy, prioritize tight blood sugar control through diet, exercise, and medication adherence, as high glucose damages retinal blood vessels over time. Schedule annual dilated eye exams with an ophthalmologist to catch issues early, when they're most treatable.

Key Prevention Steps

Monitor blood sugar daily (or use continuous glucose monitoring) to keep levels steady.

Follow a low-glycemic diet rich in omega-3s (like fish), limit sugars/processed foods, and maintain a healthy weight.

Exercise regularly-at least 150 minutes weekly of moderate activity like walking-to help regulate glucose and blood pressure.

Quit smoking, limit alcohol, prioritize sleep, and manage cholesterol/blood pressure.

Protect eyes daily: Wear UV-blocking sunglasses and use the 20-20-20 rule for screens.

Treatment Options if Needed

Early stages often stabilize with lifestyle changes alone, but advanced cases may require anti-VEGF injections, corticosteroids, or laser therapy to reduce swelling and abnormal vessels. See your doctor promptly for personalized care-early intervention can prevent vision loss.

**Q49. Can an AI chatbot replace advice from my eye doctor?**

**model: ChatGPT**

*answer:*

AI chatbots can provide general information, education, and guidance about eye conditions, including diabetic retinopathy, but they cannot replace advice from your eye doctor. Here's why:

1. Personalized diagnosis requires examination: Your eye doctor uses specialized equipment like fundus cameras, OCT scans, and slit-lamp exams to evaluate your retina. AI chatbots cannot perform these exams.

2. Complex decision-making: Treatment decisions for conditions like diabetic retinopathy-such as laser therapy, injections, or surgery-depend on your exact eye findings, history, and overall health. AI cannot make individualized treatment plans.

3. Monitoring changes over time: Eye conditions can progress rapidly. Doctors track subtle changes and adjust treatment accordingly. AI can't monitor your eyes in real time.

4. Safety and legal responsibility: Only licensed clinicians can prescribe treatments or intervene surgically. AI can give information but cannot assume responsibility for medical outcomes.

Best use of AI chatbots:

Learning about eye diseases, symptoms, and risk factors

Preparing questions for your next appointment

Understanding treatment options and what they involve

Think of AI as a knowledgeable assistant or study tool-not a substitute for your doctor. If you notice changes like blurred vision, floaters, or sudden vision loss, you should contact your ophthalmologist immediately.

**model: Copilot**

*answer:*

No - an AI chatbot cannot replace medical advice from your eye doctor, but it can help you understand general eye-health information. The key difference is that AI can educate, but only a trained clinician can diagnose, examine, and treat you safely.

Bottom line

AI chatbots can provide general education, clarify medical jargon, and sometimes offer surprisingly accurate explanations, but experts consistently warn that they should never be used as a substitute for personalized medical care.

Why AI can't replace your eye doctor

Clinical examination - AI cannot look into your eyes, measure pressure, examine your retina, or detect subtle signs of disease. Doctors rely on physical findings that AI cannot access.

Medical history integration - AI lacks access to your full medical history, medications, genetics, and risk factors - all essential for accurate diagnosis.

Nuanced judgment - Experienced ophthalmologists catch small details that AI models miss, especially in complex or urgent cases.

Safety and accuracy limits - AI can "hallucinate" incorrect facts, and even high-quality models still produce occasional errors.

What AI can do well

General education - Explain symptoms, conditions, or treatments in plain language.

Clarify doctor instructions - Help you understand what your doctor said after a visit.

Triage support - Research shows AI can sometimes match or approach expert-level performance in answering ophthalmology questions and may help non-specialists decide when to seek urgent care.

Comparable accuracy in some Q&A - Studies show AI answers can be similar in quality to ophthalmologists for many patient questions - but still not a replacement.

What the research actually says

AI chatbots can provide accurate, empathetic, and detailed answers to many eye-health questions, sometimes approaching expert-level quality.

They may even help triage which eye problems need urgent care.

But major eye-health organizations emphasize that AI must not replace direct medical advice, diagnosis, or treatment.

When you should not rely on AI

Sudden vision loss

Eye pain or redness

Flashes, floaters, or curtain-like shadows

Trauma or chemical exposure

Worsening symptoms after treatment

These require immediate evaluation by an eye doctor.

How to use AI safely for eye health

Treat AI as a learning tool, not a diagnostic tool.

Use it to prepare questions for your doctor.

Ask it to explain medical terms or compare treatment options in general terms.

Always confirm decisions with a licensed ophthalmologist or optometrist.

**model: DeepSeek**

*answer:*

This is an excellent and important question. The short, direct answer is no, an AI chatbot cannot replace advice from your eye doctor (ophthalmologist or optometrist).

Here's a detailed breakdown of why they are not equivalent, followed by where AI can be helpful.

Why a Chatbot Cannot Replace an Eye Doctor

Think of an eye exam as a combination of physical inspection, medical diagnosis, and mechanical measurement. A text-based chatbot can do none of these.

1. No Ability to Physically Examine Your Eyes (The Biggest Limitation)

Slit-lamp exam: Doctors use a microscope with a bright light to see the front of your eye (cornea, iris, lens) in 3D, high magnification, and with different stains (e.g., fluorescein to find scratches).

Ophthalmoscopy (fundus exam): They look inside your eye at the retina, optic nerve, and blood vessels. This is how they detect glaucoma, diabetic retinopathy, and macular degeneration.

Tonometry (eye pressure test): This measures intraocular pressure, the primary screening test for glaucoma. A chatbot cannot blow a puff of air or touch a probe to your eye.

Pupil dilation: Drops are used to open your pupil for a much wider, deeper view of the internal structures. A chatbot cannot do this or interpret the live 3D results.

2. No Measurement of Visual Function

Refraction (the "Which is better, 1 or 2?" test): This precisely measures your refractive error (nearsightedness, farsightedness, astigmatism, presbyopia) to determine your prescription for glasses or contacts. You cannot do this accurately yourself or with a chatbot.

Visual field test: This maps your peripheral vision. Missing spots can indicate glaucoma, brain tumors, or stroke damage. A chatbot cannot administer or reliably interpret this without specialized equipment.

Binocular vision assessment: Checking how well your eyes work together (alignment, focusing, tracking) is crucial for diagnosing strabismus (crossed eyes), convergence insufficiency, and eye strain. This requires live observation and tests.

3. High Stakes and Serious Medical Risk

Missing a silent disease: Many serious eye diseases have no early symptoms. Glaucoma is famously the "silent thief of sight" because it destroys peripheral vision first, which you won't notice until it's severe. A chatbot has no way to screen for this.

Misdiagnosis can cause blindness or death: What a patient describes as "floaters and flashes" could be a benign aging change, a retinal tear (urgent), or a retinal detachment (emergency). What a patient describes as "a headache behind my eye" could be a migraine, or it could be acute angle-closure glaucoma or even a brain aneurysm. A chatbot cannot perform the necessary tests to rule out the dangerous possibilities.

Systemic diseases first seen in the eye: Diabetes, high blood pressure, high cholesterol, multiple sclerosis, and even some cancers and autoimmune diseases are often first detected during a routine eye exam by looking at the retina's blood vessels. A chatbot has no way to "see" these signs.

Where AI Chatbots Can Be Helpful (As a Tool, Not a Replacement)

When used responsibly, an AI like me can be a useful supplement to professional eye care.

You CAN ask a chatbot for:

General information: "Explain the difference between an ophthalmologist and an optometrist." "What is age-related macular degeneration?"

Terminology clarification: "What does '20/20 vision' actually mean?"

Pre-visit preparation: "What should I expect during a routine eye exam?"

Post-visit follow-up: "My doctor said I have blepharitis. What home care tips are usually recommended?" (Always confirm with your doctor's specific advice).

Medication information: "What are common side effects of prescription eye drops for glaucoma?"

Lifestyle/remedy suggestions (for minor, non-urgent issues): "What are some evidence-based techniques for digital eye strain?" or "What are the steps for safely using a warm compress for a stye?"

You should NEVER ask a chatbot (or Dr. Google) for:

A medical diagnosis ("What is this red spot in my eye?")

A treatment plan ("What antibiotic do I need for this pink eye?")

An emergency assessment ("Should I go to the ER for this sudden vision loss?")

To rule out a serious condition ("It's probably just a floater, ignore it.")

The Bottom Line: Danger Zones

Sudden vision loss, change, or distortion (straight lines look wavy)

New floaters, flashes of light, or a dark curtain/veil over your vision (retinal detachment warning signs)

Severe eye pain or a red eye (could be acute glaucoma, severe uveitis, infection)

Eye injury (chemical splash, puncture, blunt trauma)

Any new symptom in someone with diabetes, high blood pressure, or an autoimmune disease

For any of the above, do not ask a chatbot. Go to an eye doctor or emergency room immediately.

Final Verdict

Task; AI Chatbot; Eye Doctor

Give general info about eye anatomy/disease; Good; Expert

Provide a glasses/contact lens prescription; Impossible; Essential

Diagnose pink eye; Dangerous (viral/bacterial/allergic?); Correct

Screen for silent diseases like glaucoma; Impossible; Life-saving

Examine your retina; Can't; Yes, with tools

Treat an emergency or injury; Can't; Yes

Think of it this way: An AI chatbot is like a very smart textbook or a health-conscious friend. It can explain concepts and offer general suggestions. An eye doctor is a skilled technician, diagnostician, and medical professional who uses specialized tools to inspect, measure, and treat your living body.

**model: Doubao**

*answer:*

Absolutely no - an AI chatbot cannot and cannot replace a real eye doctor's professional medical advice.

Key reasons:

No physical eye examinationAI can't check your vision, eye pressure, retina, cornea, or do slit-lamp/ fundus exams - these are essential for accurate diagnosis.

No personalized medical historyIt doesn't know your full health background, chronic diseases (like diabetes, hypertension), medication use, or past eye conditions that affect your eye health.

Only general information, not clinical diagnosisAI can give general eye health knowledge, but cannot diagnose eye diseases, prescribe eye drops, or decide treatment plans.

Risk of missed serious eye problemsMany eye diseases (glaucoma, diabetic retinopathy, retinal detachment) have subtle early symptoms; relying on AI may delay treatment and cause permanent vision loss.

What AI can do:

Provide general eye care knowledge

Explain common eye terminology

Give daily eye protection suggestions

Help you understand doctor's advice more easily

Always see a professional ophthalmologist for any eye discomfort, vision changes, or medical treatment needs.

**model: Gemini**

*answer:*

The short answer is no. While AI chatbots are becoming impressively capable, they are designed to be assistants to you and your doctor, not replacements for professional medical care.

Think of an AI chatbot like a very advanced encyclopedia-it can provide information, but it can't perform an exam or take legal responsibility for your health. Here is a breakdown of why a doctor's visit is still essential:

1. Physical Exams are "Hands-On"

An eye doctor uses specialized equipment (like slit lamps and retinal cameras) to look inside your eye. They check your eye pressure, examine the health of your retina, and look for physical signs of disease that a chatbot simply cannot see through a screen.

2. The "Hallucination" Risk

AI models can sometimes "hallucinate," which means they might state a medical "fact" that sounds perfectly confident but is actually incorrect. In eye care, following the wrong advice could lead to delayed treatment for serious conditions like glaucoma or retinal detachment, which can cause permanent vision loss.

3. Context and Nuance

Your doctor knows your personal medical history, your family's health patterns, and how your eyes have changed over the years. They can spot subtle clues-like a tiny change in a blood vessel-that might indicate a larger health issue like high blood pressure or diabetes.

4. Legal and Safety Standards

Doctors are licensed, regulated, and held to strict safety standards. They can write prescriptions for glasses, contacts, or medications; currently, AI chatbots cannot legally or safely perform these tasks.

How You Can Use AI for Eye Health

While it shouldn't replace your doctor, AI can be a great educational tool. You can use it to:

Clarify terms: "What does 'astigmatism' actually mean?"

Prepare for a visit: "What questions should I ask my doctor about my dry eyes?"

Understand a diagnosis: "My doctor said I have a stye. How do I apply a warm compress safely?"

The Bottom Line: If you're experiencing sudden vision changes, pain, or flashes of light, skip the chatbot and call an eye professional immediately. For general curiosity, feel free to ask away-just verify the important stuff with your human expert!

**model: Perplexity**

*answer:*

No, an AI chatbot cannot replace advice from your eye doctor. While AI can provide helpful general information on eye health, it lacks the ability to perform physical exams, review your medical history in person, or diagnose conditions accurately without hands-on evaluation.

AI's Strengths

AI chatbots like those studied in recent research can generate responses to common eye questions that are comparable to ophthalmologists' in accuracy and empathy for straightforward cases. For instance, a 2023 JAMA study found ophthalmologists could only distinguish AI from human answers about 61% of the time, with similar rates of potential harm. They excel at triaging basic symptoms or explaining general concepts, sometimes outperforming non-specialist doctors.

Key Limitations

AI cannot replace a doctor's expertise because it misses critical elements like in-person exams, imaging interpretation, or personalized context. Studies emphasize verifying AI info with a professional, as errors can occur even if rare, and it doesn't handle complex or urgent cases reliably. Ophthalmologists provide tailored care that AI augments but doesn't supplant.

When to Use AI

Consult AI for quick education on topics like myopia or routine care, but always follow up with your eye doctor for symptoms, prescriptions, or concerns. This "trust but verify" approach ensures safety.

**Q50. What information about my diabetes and eyesight should I bring to an eye appointment?**

**model: ChatGPT**

*answer:*

When you go to an eye appointment, especially if you have diabetes, bringing a clear record of your health and vision history can help the ophthalmologist assess your risk for diabetic eye disease and tailor management. Here's a detailed checklist:

1. Diabetes Information

Type of diabetes (Type 1, Type 2, or other)

Date of diagnosis

Most recent blood sugar readings (fasting glucose, post-meal, or daily logs)

HbA1c levels from the past 6-12 months

Medications (names, doses, and schedule) including insulin or oral medications

History of hypoglycemia or hyperglycemia events

Other diabetes-related complications (kidney issues, neuropathy, cardiovascular disease)

2. Vision and Eye History

Previous eye exams: dates and findings, especially any diagnosis of diabetic retinopathy

Past eye treatments:

Laser therapy

Injections (anti-VEGF or steroids)

Surgeries (vitrectomy, cataract surgery)

History of vision changes: blurriness, floaters, flashes of light, visual field loss

Corrective lenses: current glasses or contact lens prescriptions

Eye injuries or infections in the past

3. General Health Information

Blood pressure readings and hypertension history

Cholesterol and lipid profile

Current medications (including supplements)

Allergies (especially to medications used in eye procedures)

Family history of eye disease (glaucoma, macular degeneration)

4. Documents and Records

Printouts of recent lab results

Copies of previous eye exam notes and retinal photos if available

Medication list with doses and schedules

5. Questions to Prepare

Are there signs of diabetic retinopathy or macular edema?

Do I need treatment now or preventive measures?

How often should I return for eye exams?

How can I protect my vision while managing diabetes?

**model: Copilot**

*answer:*

You'll want to bring specific diabetes-related and vision-related information that helps the eye doctor understand your risks and detect any early signs of diabetic eye disease. Below is a clear, structured list of what to prepare, based on authoritative guidance from diabetes and eye-health organizations.

Most important takeaway

Bring your recent diabetes numbers, your medication list, any vision changes you've noticed, and your previous eye exam results. These give the doctor the context they need to detect diabetic retinopathy, macular edema, glaucoma, or cataracts early.

What diabetes-related information to bring

Recent A1C results - Eye disease risk increases when A1C is high; doctors use this to understand your baseline risk.

Blood glucose patterns - Especially if you've had frequent highs or lows recently.

Current diabetes medications - Insulin types/doses, GLP-1s, SGLT2s, metformin, etc.

Blood pressure and cholesterol history - High BP and cholesterol increase risk of diabetic eye disease.

Smoking status - Smoking raises risk of retinopathy progression.

Pregnancy status (if applicable) - Pregnancy can accelerate diabetic eye disease.

What vision-related information to bring

Any recent vision changes - Blurriness, floaters, flashes, dark spots, double vision, or trouble focusing. These can signal retinopathy or other issues.

History of eye conditions - Glaucoma, cataracts, dry eye, previous injuries, or surgeries.

Current glasses or contact lens prescription - Diabetes can cause rapid shifts in vision.

Photos or reports from previous eye exams - Retinal photos, OCT scans, or dilation exam notes help track progression.

Documents and items to bring

Insurance card - Most diabetes eye exams are covered.

List of all medications - Not just diabetes meds; some drugs affect eye pressure.

Sunglasses - Dilation can cause light sensitivity for 2-4 hours.

A driver or transportation plan - If your pupils will be dilated.

What your eye doctor will look for

These are the major diabetes-related eye conditions your doctor screens for:

Diabetic retinopathy - Leading cause of blindness in working-age adults.

Macular edema

Glaucoma - Diabetes increases risk.

Cataracts - Occur earlier and more often in people with diabetes.

**model: DeepSeek**

*answer:*

It's great that you're preparing. For an eye appointment when you have diabetes, bring:

Your diabetes history

Type of diabetes (type 1, type 2, or other)

Approximate date of diagnosis

Most recent A1c value (and date)

Current diabetes medications (insulin, oral meds, GLP-1s, etc.)

Vision symptoms (even if intermittent)

Blurriness, floaters, flashes, dark spots, distorted lines, sudden vision loss, trouble seeing at night, or double vision

When symptoms started and how often they occur

Other medical information

Blood pressure and cholesterol levels (if known, especially from last few months)

Kidney disease, neuropathy, or any diabetes complications

Any hospitalizations due to diabetes (e.g., DKA, severe hypoglycemia)

Eye history

Date of last dilated eye exam, if any

Previous diagnoses (e.g., diabetic retinopathy, macular edema, glaucoma, cataract)

Any prior eye treatments (laser, injections, surgery)

Medication and allergy list

All prescriptions, over-the-counter meds, supplements

Any known drug allergies (especially to dilating drops or fluorescein dye)

Logs if available

Recent blood sugar logs (or CGM printout)

Note any pattern of high or low sugars around vision changes

Questions for the doctor

Do I have diabetic retinopathy or macular edema?

Do I need a retinal imaging or OCT test?

How often should I return?

Should I see a retina specialist?

**model: Doubao**

*answer:*

1. Diabetes-related medical info

Recent blood sugar records: fasting glucose, post-meal glucose, latest HbA1c results (last 3-6 months).

Diabetes type (Type 1/Type 2) and how long you've had diabetes.

Current diabetes medications: pills, insulin dosage/schedule, injectable meds.

History of diabetes complications: kidney disease, high blood pressure, high cholesterol.

2. General health records

List of all daily medications (including blood pressure, cholesterol, aspirin, supplements).

Known chronic conditions: hypertension, high cholesterol, neuropathy.

Any recent hospital stays or major medical procedures.

3. Eye-related documents

Past eye exam reports, optical coherence tomography (OCT) results, retinal photos.

Previous eye treatments: laser eye surgery, intravitreal injections, cataract surgery.

Current glasses/contacts prescription, old eye drop medications you're using.

4. Symptom details to tell your doctor

Any recent vision changes:

Blurred vision, fluctuating eyesight

Dark spots, floaters, flashes of light

Difficulty reading, night vision problems

Eye pain, redness or persistent dry eyes

5. Lifestyle info

Smoking/alcohol use

Diet and exercise routine

How well you regularly control blood sugar

**model: Gemini**

*answer:*

Preparing for an eye exam when you have diabetes is all about providing your optometrist or ophthalmologist with a clear "snapshot" of your systemic health. Because blood sugar levels directly impact the blood vessels in your retina, the more data they have, the better they can monitor for diabetic retinopathy or other changes.

Here is a checklist of the most helpful information to bring:

1. Recent Blood Sugar Data

Your eye doctor needs to know how stable your glucose has been recently, as significant fluctuations can actually cause temporary blurred vision.

Your latest A1C result: This gives them an idea of your average blood sugar over the last three months.

Recent glucose trends: If you use a Continuous Glucose Monitor (CGM) or keep a log, knowing if you've had frequent "highs" or "lows" lately is helpful.

2. Medical History & Timeline

Date of Diagnosis: How long you have lived with diabetes is one of the most important risk factors for eye-related complications.

Primary Care Info: Bring the name and contact information of the doctor managing your diabetes so the eye specialist can send them a report of the exam.

Last Dilated Exam: If you've seen a different eye doctor in the past, knowing when your last retinal screening was (and if there were any "spots" or issues found) is vital.

3. Current Medications

Diabetes is often managed alongside other conditions that can affect eye health.

Diabetes meds: List your insulin types or oral medications (like Metformin).

Blood pressure/Cholesterol: High blood pressure, in particular, can worsen diabetic eye disease, so it's important to mention if you are taking medication for it.

4. Specific Vision Symptoms

Be ready to describe any changes you've noticed, even if they seem minor. Note if you experience:

Fluctuating vision: Does your vision seem better or worse at different times of the day?

Floaters or "spots": Tiny dark strings or cobwebs in your field of vision.

Flashers: Brief sparks or strands of light.

Difficulty at night: Increased glare or trouble seeing while driving in the dark.

A quick tip for the appointment: Since this will likely be a dilated exam to get a good look at the back of your eye, your vision will be blurry and your eyes will be very light-sensitive for a few hours afterward. It's a good idea to bring a pair of sunglasses and, if possible, have someone else drive you home.

**model: Perplexity**

*answer:*

Bring a simple diabetes-and-vision summary, plus your current glasses or contacts. The most useful details are when you were diagnosed, your recent A1C and blood sugar patterns, any diabetes medicines you take now or took before, and any changes in your eyesight or eye symptoms.

Diabetes details to share

Tell the eye doctor:

How long you've had diabetes.

Your last few A1C results, if you know them.

Your usual blood sugar range, especially if it changes a lot.

All diabetes medicines you use now and any you used in the past.

Any other conditions like high blood pressure or high cholesterol.

Eyesight changes to mention

Be ready to describe:

Blurry vision, floaters, flashes, double vision, trouble focusing, or dark spots.

When the symptoms started and whether they come and go.

Whether symptoms happen when your blood sugar is high or low.

Any sudden vision loss or new distortion.

What to bring

Pack:

Your glasses and contact lenses.

A list of all medications, vitamins, and supplements with doses.

Any questions you want answered.

Sunglasses for after dilation, since your vision may be blurry for a few hour
