# Supplementary material for: Large language models as sources of diabetic retinopathy patient education: a comparative evaluation of safety, health information quality, and readability
Source: Front Public Health. 2026 Oct 9;14:1945318. doi: 10.3389/fpubh.2026.1945318 (PMC13690771; doi:10.3389/fpubh.2026.1945318)
Supplement: Supplementary file 5 [file Supplementary_file_5.docx]

**Supplementary Material 5. Additional Statistical Analyses**

**Table S1. Inter-rater agreement**

| Metric | Agreement index | Estimate | 95% CI | P value | Complete items | No. of raters | Note |
| --- | --- | --- | --- | --- | --- | --- | --- |
| Safety | Fleiss' kappa | 0.581 | 0.531-0.630 | <0.001 | 300 | 5 | Primary agreement index for Safety; 95% CI based on 3000 nonparametric bootstrap resamples. |
| Accuracy | ICC(2,1) | 0.837 | 0.811-0.861 | <0.001 | 300 | 5 | Two-way random-effects, absolute-agreement, single-measure ICC. |
| Empathy | ICC(2,1) | 0.807 | 0.776-0.835 | <0.001 | 300 | 5 | Two-way random-effects, absolute-agreement, single-measure ICC. |
| DISCERN | ICC(2,1) | 0.870 | 0.848-0.889 | <0.001 | 300 | 5 | Two-way random-effects, absolute-agreement, single-measure ICC. |
| EQIP | ICC(2,1) | 0.806 | 0.774-0.836 | <0.001 | 300 | 5 | Two-way random-effects, absolute-agreement, single-measure ICC. |
| JAMA | ICC(2,1) | 0.866 | 0.844-0.887 | <0.001 | 300 | 5 | Two-way random-effects, absolute-agreement, single-measure ICC. |
| GQS | ICC(2,1) | 0.857 | 0.834-0.879 | <0.001 | 300 | 5 | Two-way random-effects, absolute-agreement, single-measure ICC. |

Note: Safety was assessed using Fleiss' kappa. Accuracy, Empathy, DISCERN, EQIP, JAMA and GQS were assessed using ICC(2,1).

**Table S2. Pairwise comparisons of safety**

| Comparison | N pairs | Safe rate difference, % | Safe rate difference 95% CI | Safe to unsafe, n | Unsafe to safe, n | Matched OR | Matched OR 95% CI | Adjusted *P* | Note |
| --- | --- | --- | --- | --- | --- | --- | --- | --- | --- |
| ChatGPT vs Gemini | 50 | 2.00 | -8.00-12.00 | 4 | 3 | 1.33 | 0.30-5.96 | 1.000 | Pairwise McNemar test |
| ChatGPT vs DeepSeek | 50 | 6.00 | -4.00-16.00 | 5 | 2 | 2.50 | 0.48-12.89 | 1.000 | Pairwise McNemar test |
| ChatGPT vs Doubao | 50 | 2.00 | -6.00-10.00 | 3 | 2 | 1.50 | 0.25-8.98 | 1.000 | Pairwise McNemar test |
| ChatGPT vs Copilot | 50 | 4.00 | -8.00-16.00 | 6 | 4 | 1.50 | 0.42-5.32 | 1.000 | Pairwise McNemar test |
| ChatGPT vs Perplexity | 50 | -2.00 | -12.00-8.00 | 3 | 4 | 0.75 | 0.17-3.35 | 1.000 | Pairwise McNemar test |
| Gemini vs DeepSeek | 50 | 4.00 | -4.00-12.00 | 3 | 1 | 3.00 | 0.31-28.84 | 1.000 | Pairwise McNemar test |
| Gemini vs Doubao | 50 | 0.00 | -10.00-12.00 | 4 | 4 | 1.00 | 0.25-4.00 | 1.000 | Pairwise McNemar test |
| Gemini vs Copilot | 50 | 2.00 | -10.00-14.00 | 5 | 4 | 1.25 | 0.34-4.66 | 1.000 | Pairwise McNemar test |
| Gemini vs Perplexity | 50 | -4.00 | -12.00-4.00 | 1 | 3 | 0.33 | 0.03-3.20 | 1.000 | Pairwise McNemar test |
| DeepSeek vs Doubao | 50 | -4.00 | -16.00-6.00 | 3 | 5 | 0.60 | 0.14-2.51 | 1.000 | Pairwise McNemar test |
| DeepSeek vs Copilot | 50 | -2.00 | -14.00-10.00 | 4 | 5 | 0.80 | 0.21-2.98 | 1.000 | Pairwise McNemar test |
| DeepSeek vs Perplexity | 50 | -8.00 | -18.00-2.00 | 1 | 5 | 0.20 | 0.02-1.71 | 1.000 | Pairwise McNemar test |
| Doubao vs Copilot | 50 | 2.00 | -8.00-12.00 | 4 | 3 | 1.33 | 0.30-5.96 | 1.000 | Pairwise McNemar test |
| Doubao vs Perplexity | 50 | -4.00 | -14.00-4.00 | 2 | 4 | 0.50 | 0.09-2.73 | 1.000 | Pairwise McNemar test |
| Copilot vs Perplexity | 50 | -6.00 | -16.00-2.00 | 1 | 4 | 0.25 | 0.03-2.24 | 1.000 | Pairwise McNemar test |

Note: Pairwise comparisons were performed using McNemar tests with Benjamini-Hochberg correction. Safe-rate differences and their bootstrap 95% CIs are reported.

**Table S3 Significant pairwise comparisons for non-binary outcomes**

| **Metric** | **Comparison** | **N pairs** | **Mean difference** | **Mean difference 95% CI** | **Median difference** | **Median difference 95% CI** | **Rank-biserial correlation** | **Rank-biserial 95% CI** | **Adjusted *P*** |
| --- | --- | --- | --- | --- | --- | --- | --- | --- | --- |
| Accuracy | ChatGPT vs Gemini | 50 | 0.29 | 0.13-0.44 | 0.00 | 0.00-0.50 | 0.634 | 0.315-0.869 | 0.012 |
| Accuracy | ChatGPT vs DeepSeek | 50 | 0.24 | 0.06-0.42 | 0.00 | 0.00-0.50 | 0.471 | 0.091-0.806 | 0.045 |
| Accuracy | ChatGPT vs Doubao | 50 | 0.28 | 0.12-0.43 | 0.00 | 0.00-0.20 | 0.617 | 0.316-0.869 | 0.012 |
| Accuracy | Gemini vs Copilot | 50 | -0.21 | -0.37--0.05 | 0.00 | -0.20-0.00 | -0.492 | -0.810--0.125 | 0.045 |
| Accuracy | Gemini vs Perplexity | 50 | -0.19 | -0.32--0.07 | 0.00 | 0.00-0.00 | -0.700 | -0.933--0.298 | 0.022 |
| Accuracy | Doubao vs Copilot | 50 | -0.19 | -0.35--0.04 | 0.00 | -0.10-0.00 | -0.566 | -0.872--0.187 | 0.037 |
| Accuracy | Doubao vs Perplexity | 50 | -0.18 | -0.30--0.06 | 0.00 | 0.00-0.00 | -0.753 | -1.000--0.381 | 0.019 |
| Empathy | ChatGPT vs Doubao | 50 | 0.60 | 0.33-0.88 | 0.80 | 0.00-1.00 | 0.627 | 0.360-0.854 | 0.002 |
| Empathy | ChatGPT vs Copilot | 50 | -0.34 | -0.62--0.06 | 0.00 | -0.60-0.00 | -0.432 | -0.721--0.092 | 0.038 |
| Empathy | Gemini vs Doubao | 50 | 0.55 | 0.28-0.82 | 0.30 | 0.00-1.00 | 0.621 | 0.341-0.849 | 0.002 |
| Empathy | Gemini vs Copilot | 50 | -0.39 | -0.64--0.15 | -0.60 | -0.80-0.00 | -0.425 | -0.728--0.094 | 0.030 |
| Empathy | DeepSeek vs Doubao | 50 | 0.56 | 0.31-0.79 | 0.70 | 0.20-1.00 | 0.721 | 0.456-0.916 | <0.001 |
| Empathy | DeepSeek vs Copilot | 50 | -0.38 | -0.64--0.14 | -0.20 | -0.90-0.00 | -0.424 | -0.706--0.107 | 0.030 |
| Empathy | Doubao vs Copilot | 50 | -0.94 | -1.20--0.68 | -1.00 | -1.10--0.80 | -0.860 | -0.979--0.688 | <0.001 |
| Empathy | Doubao vs Perplexity | 50 | -0.40 | -0.70--0.10 | -0.20 | -1.00-0.00 | -0.386 | -0.675--0.062 | 0.043 |
| Empathy | Copilot vs Perplexity | 50 | 0.54 | 0.24-0.83 | 0.20 | 0.00-1.00 | 0.539 | 0.245-0.786 | 0.007 |
| DISCERN | ChatGPT vs Gemini | 50 | -2.35 | -3.96--0.80 | -1.80 | -3.70--0.20 | -0.412 | -0.678--0.118 | 0.015 |
| DISCERN | ChatGPT vs Doubao | 50 | 4.12 | 2.58-5.57 | 4.80 | 2.60-6.60 | 0.717 | 0.502-0.893 | <0.001 |
| DISCERN | ChatGPT vs Copilot | 50 | -2.52 | -3.71--1.28 | -2.50 | -4.00--1.00 | -0.593 | -0.811--0.331 | <0.001 |
| DISCERN | ChatGPT vs Perplexity | 50 | -3.46 | -4.52--2.39 | -3.40 | -5.50--1.90 | -0.786 | -0.933--0.586 | <0.001 |
| DISCERN | Gemini vs DeepSeek | 50 | 2.98 | 1.71-4.29 | 2.70 | 1.60-4.60 | 0.633 | 0.372-0.836 | <0.001 |
| DISCERN | Gemini vs Doubao | 50 | 6.47 | 5.24-7.75 | 6.60 | 5.00-7.80 | 0.941 | 0.850-0.998 | <0.001 |
| DISCERN | DeepSeek vs Doubao | 50 | 3.49 | 2.13-4.84 | 2.80 | 1.20-4.60 | 0.704 | 0.474-0.895 | <0.001 |
| DISCERN | DeepSeek vs Copilot | 50 | -3.15 | -4.32--1.95 | -3.00 | -4.80--1.50 | -0.707 | -0.887--0.481 | <0.001 |
| DISCERN | DeepSeek vs Perplexity | 50 | -4.09 | -5.46--2.77 | -3.00 | -5.40--1.80 | -0.765 | -0.914--0.556 | <0.001 |
| DISCERN | Doubao vs Copilot | 50 | -6.64 | -7.85--5.46 | -7.60 | -9.00--5.70 | -0.949 | -0.994--0.860 | <0.001 |
| DISCERN | Doubao vs Perplexity | 50 | -7.58 | -8.92--6.18 | -7.40 | -10.40--6.20 | -0.968 | -1.000--0.901 | <0.001 |
| EQIP | ChatGPT vs Gemini | 50 | -5.20 | -6.60--3.80 | -5.00 | -5.06--5.00 | -0.887 | -0.978--0.743 | <0.001 |
| EQIP | ChatGPT vs Doubao | 50 | 3.90 | 2.30-5.50 | 5.00 | 0.00-5.00 | 0.740 | 0.475-0.927 | <0.001 |
| EQIP | ChatGPT vs Copilot | 50 | -6.90 | -8.50--5.30 | -10.00 | -10.00--5.00 | -0.948 | -1.000--0.855 | <0.001 |
| EQIP | ChatGPT vs Perplexity | 50 | -5.40 | -6.90--3.90 | -5.00 | -5.00--5.00 | -0.895 | -1.000--0.704 | <0.001 |
| EQIP | Gemini vs DeepSeek | 50 | 5.60 | 4.20-7.00 | 5.00 | 5.00-5.00 | 0.938 | 0.827-1.000 | <0.001 |
| EQIP | Gemini vs Doubao | 50 | 9.10 | 7.50-10.70 | 10.00 | 5.00-10.00 | 0.984 | 0.940-1.000 | <0.001 |
| EQIP | Gemini vs Copilot | 50 | -1.70 | -3.20--0.20 | 0.00 | -5.00-0.00 | -0.430 | -0.738--0.079 | 0.031 |
| EQIP | DeepSeek vs Doubao | 50 | 3.50 | 1.90-5.00 | 5.00 | 2.50-5.00 | 0.691 | 0.405-0.935 | <0.001 |
| EQIP | DeepSeek vs Copilot | 50 | -7.30 | -8.70--6.00 | -7.50 | -10.00--5.00 | -0.980 | -1.000--0.927 | <0.001 |
| EQIP | DeepSeek vs Perplexity | 50 | -5.80 | -7.30--4.40 | -5.00 | -10.00--5.00 | -0.923 | -1.000--0.809 | <0.001 |
| EQIP | Doubao vs Copilot | 50 | -10.80 | -12.40--9.30 | -10.00 | -10.00--10.00 | -0.980 | -1.000--0.937 | <0.001 |
| EQIP | Doubao vs Perplexity | 50 | -9.30 | -10.70--7.90 | -10.00 | -10.00--5.00 | -1.000 | -1.000--1.000 | <0.001 |
| JAMA | ChatGPT vs Gemini | 50 | -0.36 | -0.58--0.13 | -0.30 | -1.00-0.00 | -0.506 | -0.776--0.204 | 0.006 |
| JAMA | ChatGPT vs Copilot | 50 | -0.80 | -1.00--0.61 | -1.00 | -1.00--0.60 | -0.972 | -1.000--0.903 | <0.001 |
| JAMA | ChatGPT vs Perplexity | 50 | -0.89 | -1.10--0.69 | -1.00 | -1.00--0.60 | -0.965 | -1.000--0.893 | <0.001 |
| JAMA | Gemini vs DeepSeek | 50 | 0.38 | 0.19-0.56 | 0.40 | 0.00-0.60 | 0.701 | 0.396-0.941 | <0.001 |
| JAMA | Gemini vs Doubao | 50 | 0.46 | 0.26-0.66 | 0.50 | 0.00-0.90 | 0.673 | 0.367-0.915 | <0.001 |
| JAMA | Gemini vs Copilot | 50 | -0.44 | -0.63--0.25 | -0.20 | -0.80-0.00 | -0.750 | -0.971--0.467 | <0.001 |
| JAMA | Gemini vs Perplexity | 50 | -0.53 | -0.77--0.28 | -0.80 | -1.00-0.00 | -0.668 | -0.869--0.398 | <0.001 |
| JAMA | DeepSeek vs Copilot | 50 | -0.82 | -1.01--0.63 | -0.80 | -1.00--0.60 | -0.985 | -1.000--0.941 | <0.001 |
| JAMA | DeepSeek vs Perplexity | 50 | -0.91 | -1.13--0.68 | -1.00 | -1.00--0.60 | -0.925 | -0.993--0.808 | <0.001 |
| JAMA | Doubao vs Copilot | 50 | -0.90 | -1.10--0.70 | -1.00 | -1.00--0.80 | -0.945 | -1.000--0.828 | <0.001 |
| JAMA | Doubao vs Perplexity | 50 | -0.99 | -1.20--0.78 | -1.00 | -1.00--0.80 | -0.944 | -1.000--0.839 | <0.001 |
| GQS | ChatGPT vs DeepSeek | 50 | 0.34 | 0.11-0.57 | 0.00 | 0.00-0.80 | 0.578 | 0.235-0.855 | 0.014 |
| GQS | DeepSeek vs Doubao | 50 | -0.55 | -0.78--0.33 | -0.80 | -0.90-0.00 | -0.741 | -0.935--0.477 | <0.001 |
| GQS | DeepSeek vs Copilot | 50 | -0.47 | -0.67--0.28 | -0.40 | -1.00-0.00 | -0.776 | -0.940--0.535 | <0.001 |
| GQS | DeepSeek vs Perplexity | 50 | -0.34 | -0.53--0.16 | -0.20 | -0.60-0.00 | -0.593 | -0.837--0.289 | 0.007 |
| ARI | ChatGPT vs Doubao | 50 | -3.41 | -5.19--1.70 | -3.22 | -6.02--1.20 | -0.594 | -0.820--0.332 | 0.001 |
| ARI | ChatGPT vs Copilot | 50 | -1.66 | -2.92--0.40 | -2.03 | -3.33--0.47 | -0.427 | -0.702--0.133 | 0.018 |
| ARI | ChatGPT vs Perplexity | 50 | -1.48 | -2.95-0.04 | -2.43 | -3.52--0.99 | -0.402 | -0.693--0.111 | 0.022 |
| ARI | Gemini vs Doubao | 50 | -3.88 | -5.25--2.66 | -3.63 | -4.38--2.30 | -0.779 | -0.925--0.561 | <0.001 |
| ARI | Gemini vs Copilot | 50 | -2.14 | -3.05--1.21 | -1.94 | -3.23--0.93 | -0.682 | -0.884--0.432 | <0.001 |
| ARI | Gemini vs Perplexity | 50 | -1.95 | -2.99--0.91 | -1.37 | -3.76--0.11 | -0.514 | -0.749--0.236 | 0.005 |
| ARI | DeepSeek vs Doubao | 50 | -2.69 | -4.23--0.97 | -3.42 | -5.09--1.92 | -0.600 | -0.834--0.313 | 0.001 |
| ARI | Doubao vs Copilot | 50 | 1.75 | 0.53-3.05 | 1.32 | 0.04-2.80 | 0.402 | 0.103-0.661 | 0.022 |
| ARI | Doubao vs Perplexity | 50 | 1.93 | 0.65-3.30 | 1.74 | 0.42-3.69 | 0.462 | 0.162-0.713 | 0.011 |
| CL | ChatGPT vs Gemini | 50 | 0.99 | 0.34-1.65 | 1.12 | 0.20-1.55 | 0.415 | 0.129-0.691 | 0.020 |
| CL | ChatGPT vs Doubao | 50 | -2.92 | -3.64--2.20 | -2.77 | -3.47--1.94 | -0.901 | -0.995--0.757 | <0.001 |
| CL | Gemini vs Doubao | 50 | -3.91 | -4.62--3.17 | -3.80 | -4.79--3.00 | -0.969 | -1.000--0.904 | <0.001 |
| CL | Gemini vs Copilot | 50 | -1.50 | -1.91--1.08 | -1.49 | -2.09--0.99 | -0.821 | -0.967--0.631 | <0.001 |
| CL | DeepSeek vs Doubao | 50 | -3.33 | -4.01--2.68 | -3.10 | -4.30--2.25 | -0.947 | -1.000--0.849 | <0.001 |
| CL | DeepSeek vs Copilot | 50 | -0.92 | -1.52--0.33 | -1.64 | -2.00--0.54 | -0.504 | -0.766--0.212 | 0.004 |
| CL | Doubao vs Copilot | 50 | 2.41 | 1.87-2.97 | 2.39 | 1.63-2.63 | 0.909 | 0.765-0.994 | <0.001 |
| CL | Doubao vs Perplexity | 50 | 3.13 | 2.36-3.85 | 3.66 | 2.48-4.16 | 0.868 | 0.711-0.967 | <0.001 |
| FKGL | ChatGPT vs Doubao | 50 | -2.88 | -4.21--1.56 | -2.69 | -4.82--1.44 | -0.609 | -0.828--0.340 | <0.001 |
| FKGL | Gemini vs Doubao | 50 | -3.88 | -4.98--2.81 | -3.63 | -4.75--2.93 | -0.881 | -0.970--0.748 | <0.001 |
| FKGL | Gemini vs Copilot | 50 | -1.82 | -2.55--1.06 | -1.75 | -2.31--1.43 | -0.713 | -0.904--0.457 | <0.001 |
| FKGL | Gemini vs Perplexity | 50 | -1.48 | -2.37--0.63 | -0.94 | -2.95--0.02 | -0.476 | -0.716--0.197 | 0.007 |
| FKGL | DeepSeek vs Doubao | 50 | -2.66 | -3.90--1.25 | -3.52 | -4.33--2.38 | -0.682 | -0.898--0.437 | <0.001 |
| FKGL | Doubao vs Copilot | 50 | 2.06 | 1.10-3.06 | 1.73 | 0.86-3.08 | 0.600 | 0.340-0.816 | <0.001 |
| FKGL | Doubao vs Perplexity | 50 | 2.40 | 1.36-3.48 | 2.79 | 1.41-3.62 | 0.661 | 0.423-0.857 | <0.001 |
| GFI | ChatGPT vs Doubao | 50 | -3.16 | -4.60--1.76 | -3.59 | -5.04--1.72 | -0.641 | -0.859--0.398 | <0.001 |
| GFI | Gemini vs Doubao | 50 | -4.26 | -5.44--3.10 | -4.12 | -5.43--3.29 | -0.870 | -0.966--0.724 | <0.001 |
| GFI | Gemini vs Copilot | 50 | -1.91 | -2.70--1.13 | -1.98 | -2.69--1.42 | -0.694 | -0.900--0.454 | <0.001 |
| GFI | DeepSeek vs Doubao | 50 | -2.80 | -4.18--1.31 | -3.42 | -5.00--2.30 | -0.671 | -0.876--0.415 | <0.001 |
| GFI | Doubao vs Copilot | 50 | 2.35 | 1.32-3.36 | 2.26 | 0.48-3.28 | 0.609 | 0.350-0.815 | <0.001 |
| GFI | Doubao vs Perplexity | 50 | 3.21 | 2.09-4.39 | 3.71 | 1.88-4.63 | 0.711 | 0.471-0.887 | <0.001 |
| SMOG | ChatGPT vs Doubao | 50 | -2.12 | -3.17--1.09 | -2.09 | -3.68--1.13 | -0.600 | -0.831--0.329 | <0.001 |
| SMOG | Gemini vs Doubao | 50 | -2.52 | -3.40--1.66 | -2.45 | -3.12--1.94 | -0.776 | -0.925--0.573 | <0.001 |
| SMOG | Gemini vs Copilot | 50 | -1.20 | -1.81--0.60 | -1.31 | -1.65--1.01 | -0.647 | -0.864--0.380 | <0.001 |
| SMOG | DeepSeek vs Doubao | 50 | -1.66 | -2.62--0.62 | -2.33 | -3.20--1.17 | -0.554 | -0.801--0.264 | 0.002 |
| SMOG | Doubao vs Copilot | 50 | 1.32 | 0.57-2.10 | 1.22 | 0.08-2.06 | 0.501 | 0.224-0.738 | 0.005 |
| SMOG | Doubao vs Perplexity | 50 | 1.89 | 1.09-2.74 | 2.16 | 1.22-2.71 | 0.622 | 0.361-0.831 | <0.001 |
| FRES | ChatGPT vs Gemini | 50 | -9.47 | -14.03--5.03 | -8.38 | -12.93--1.78 | -0.575 | -0.788--0.296 | <0.001 |
| FRES | ChatGPT vs Doubao | 50 | 15.66 | 10.58-20.74 | 14.60 | 8.98-21.47 | 0.785 | 0.589-0.948 | <0.001 |
| FRES | Gemini vs Doubao | 50 | 25.13 | 20.82-29.52 | 24.37 | 19.67-30.43 | 0.978 | 0.928-1.000 | <0.001 |
| FRES | Gemini vs Copilot | 50 | 9.35 | 6.61-11.97 | 8.39 | 6.26-12.59 | 0.821 | 0.644-0.958 | <0.001 |
| FRES | Gemini vs Perplexity | 50 | 5.39 | 1.22-9.67 | 4.88 | -0.70-11.60 | 0.371 | 0.071-0.644 | 0.043 |
| FRES | DeepSeek vs Doubao | 50 | 18.93 | 13.68-23.81 | 19.92 | 17.13-23.28 | 0.835 | 0.634-0.986 | <0.001 |
| FRES | Doubao vs Copilot | 50 | -15.78 | -19.74--12.03 | -13.63 | -18.09--11.24 | -0.914 | -0.978--0.796 | <0.001 |
| FRES | Doubao vs Perplexity | 50 | -19.74 | -24.03--15.13 | -21.62 | -28.99--16.19 | -0.903 | -0.978--0.785 | <0.001 |

Note: Pairwise comparisons were performed using paired Wilcoxon signed-rank tests with Benjamini-Hochberg correction. Paired mean/median differences and rank-biserial correlations are reported with bootstrap 95% CIs. Only significant comparisons are shown.

**Table S4. Significant pairwise comparisons of safety**

Note

No significant pairwise safety difference after Benjamini-Hochberg correction.

Note: Pairwise comparisons were performed using McNemar tests with Benjamini-Hochberg correction. Safe-rate differences and their bootstrap 95% CIs are reported. Only significant comparisons are shown.
